# Precision T cell correction platform for inborn errors of immunity

**DOI:** 10.1016/j.ymthe.2025.08.018

**Published:** 2025-08-12

**Authors:** Katariina Mamia, Solrun Kolbeinsdottir, Kornel Labun, Zhuokun Li, Anna Komisarczuk, Salla Keskitalo, Ganna Reint, Frida Loe Haugen, Britt Olaug Lindestad, Siv Skundberg Jensen, Thea Johanne Gjerdingen, Antti Tuhkala, Carolina Wieczorek Ervik, Pavel Kopcil, Nail Fatkhutdinov, Karen Helene Bronken Martinsen, Hans Christian Erichsen, Monika Szymanska, Eero Tölö, Virpi Glumoff, Janna Saarela, Trond Melbye Michelsen, Camilla Schalin-Jäntti, Johanna Olweus, Eira Leinonen, Markku Varjosalo, Eivind Valen, Timo Hautala, Martin Enge, Timi Martelius, Shiva Dahal-Koirala, Emma Haapaniemi

**Affiliations:** 1 Centre for Molecular Medicine Norway, University of Oslo, 0318 Oslo, Norway; 2 Department of Pediatrics, Oslo University Hospital, 0372 Oslo, Norway; 3 Precision Immunotherapy Alliance, University of Oslo, 0379 Oslo, Norway; 4 Department of Oncology-Pathology, Karolinska Institutet, 17177 Stockholm, Sweden; 5 Computational Biology Unit, Department of Informatics, University of Bergen, 5008 Bergen, Norway; 6 Systems Biology/Pathology Research Group, University of Helsinki, 00014 Helsinki, Finland; 7 Institute of Biotechnology, HiLIFE, University of Helsinki, 00014 Helsinki, Finland; 8 Department of Cancer Immunology, Institute for Cancer Research, Oslo University Hospital Radiumhospitalet, 0310 Oslo, Norway; 9 Division of Pediatric and Adolescent Medicine, Oslo University Hospital and Institute of Clinical Medicine, University of Oslo, 0424 Oslo, Norway; 10 Faculty of Medicine, Institute of Clinical Medicine, University of Oslo, 0318 Oslo, Norway; 11 Ministry of Finance, 0030 Oslo, Norway; 12 Research Unit of Internal Medicine and Biomedicine, University of Oulu, 90014 Oulu, Finland; 13 ERN-RITA Core Center Member, RITAFIN Consortium, Infectious Diseases Clinic, Oulu University Hospital, 90220 Oulu, Finland; 14 Institute for Molecular Medicine Finland, HiLIFE, 00290 Helsinki, Finland; 15 Department of Medical Genetics, Oslo University Hospital, 0450 Oslo, Norway; 16 Department of Obstetrics, Division of Obstetrics and Gynecology, Oslo University Hospital, 0424 Oslo, Norway; 17 Endocrinology, Abdominal Center, Helsinki University Hospital, 00029 Helsinki, Finland; 18 University of Helsinki, ENDO-ERN (European Reference Network on Rare Endocrine Conditions), 00290 Helsinki, Finland; 19 Folkhälsan Institute of Genetics, and Stem Cells and Metabolism Research Program, University of Helsinki, 00014 Helsinki, Finland; 20 Department of Biosciences, University of Oslo, 0371 Oslo, Norway; 21 Inflammation Center, Department of Infectious Disease, Helsinki University Hospital and University of Helsinki, 00029 Helsinki, Finland

**Keywords:** gene therapy, inborn errors of immunity, CRISPR-Cas9 gene correction, single-nucleotide variant correction, autologous T cell therapy, platform technology, non-viral genome editing, homology-directed repair, primary T cell editing, *ex vivo* gene editing

## Abstract

CRISPR-Cas9 gene editing is a promising tool to correct pathogenic variants for autologous cell therapies targeting inborn errors of immunity (IEI). Current strategies, such as gene knockout or cDNA knockin, address many single-gene defects but can disrupt gene expression, highlighting the need for precise correction platforms. While transplanting corrected autologous hematopoietic stem cells is a curative approach, it is unsuitable for patients with advanced disease, inflammation, or acute infections. As correcting T cells is an alternative therapeutic strategy for lymphoid IEIs, we present an efficient T cell single-nucleotide variant (SNV) correction platform based on homology-directed repair (HDR). By using STAT1 gain-of-function, cartilage hair hypoplasia, deficiency of ADA2, and autoimmune polyendocrinopathy-candidiasis-ectodermal dystrophy as IEI models, we demonstrate that our platform achieves up to 80% correction, with resultant functional correction of the disease phenotype in the selected models. Furthermore, we performed safety profiling using GUIDE-seq, single-cell RNA sequencing, long-read genome sequencing, and proteomics analysis and detected no genomic, transcriptomic, or proteomic aberrations. This study establishes HDR-based SNV editing as a portable method for developing clinical autologous T cell therapies and represents a promising step toward a broad-spectrum gene correction platform for treating diverse monogenic immune disorders.

## Graphical abstract

**Figure:**
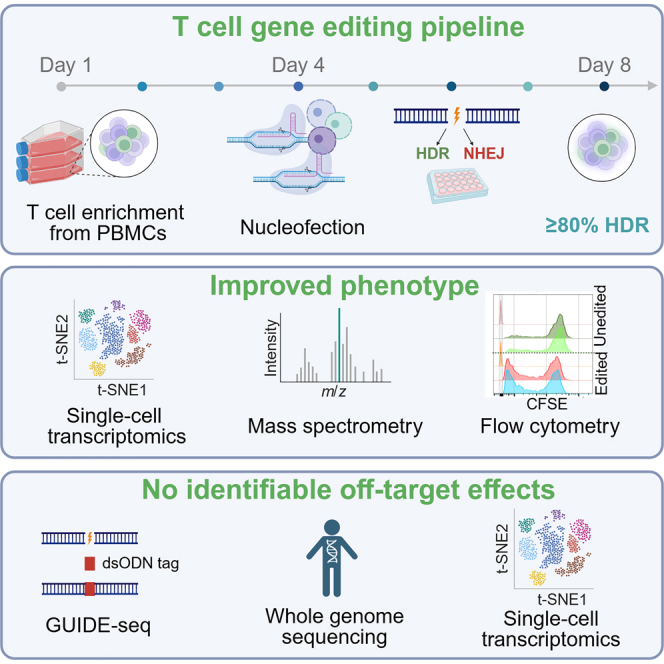

## Teaser

Mamia and colleagues present a non-viral HDR-based T cell SNV correction platform for inborn errors of immunity, achieving up to 80% correction and functional improvement in selected models. Furthermore, they performed safety profiling using GUIDE-seq, single-cell RNA-seq, long-read genome sequencing, and proteomics analysis, detecting no genomic, transcriptomic, or proteomic aberrations.

## Introduction

Inborn errors of immunity (IEI) encompass ∼555 single-gene defects that affect multiple cell types of the immune system, leading to diverse clinical presentations, including infection susceptibility, autoimmunity and inflammation, cancer predisposition, and allergies.^1^^,^^2^ IEIs are popular targets for CRISPR-Cas9 gene correction as routine clinical protocols exist for immune cell transplantation. While hematopoietic stem and progenitor cells (HSPCs) are considered the prime target for full IEI correction,^3^^,^^4^ the strategy is not always suitable due to severe clinical status, acute infections, or ongoing inflammation.^5^^,^^6^

Therapeutic benefit can also come from correcting patient T cells in IEIs that affect the T cell lymphoid compartment, such as CTLA-4 insufficiency.^7^^,^^8^^,^^9^^,^^10^^,^^11^^,^^12^^,^^13^ The corrected cells can be infused to the patient as an adoptive T cell therapy to control infections, inflammation, and other pathology that stems from faulty T cell functions.^4^ Autologous T cell transplantation offers advantages over hematopoietic stem cell (HSC) transplantation, including easier protocols for cell collection and reduced toxicity from lymphodepletion compared to the intensive chemotherapy required for HSC engraftment.^14^ Furthermore, T cell editing does not pose the same safety concerns as editing of HSCs as they are terminally differentiated and carry a lower risk of insertional mutagenesis.^15^^,^^16^ The efficacy of T cell editing for IEIs using viral delivery has previously been demonstrated for selected diseases both *in vitro* and *in vivo*,^7^^,^^10^ highlighting the translational potential in targeting T cells for gene therapy (Table S1).

For some IEIs, knockout of the disease gene can restore normal cell function.^17^^,^^18^ However, the main CRISPR-Cas9 correction strategy is to knockin the therapeutic cDNA under endogenous promoter of the diseased gene.^19^^,^^20^ Although this strategy can treat most defects caused by a single gene, it may result in suboptimal expression of the cDNA construct due to a lack of endogenous regulatory sequences.^21^^,^^22^ Furthermore, this strategy is slow to adapt to large genes, novel disease gene discoveries, or ultra-rare IEIs, which might feature only <10 patients globally. To overcome these issues, precise gene correction of the pathogenic variant is a therapeutic alternative.

Precise correction of monogenic mutations is typically attributed to base^23^^,^^24^ and prime editing^24^^,^^25^ as these methods do not induce double-stranded DNA (dsDNA) breaks and are thus considered safer alternatives.^26^^,^^27^ However, identifying safe and efficient guides are a limiting factor for both. Large screens are necessary to identify an effective prime editing guide RNA (gRNA),^28^^,^^29^^,^^30^ and guide options are limited for base editing, with a risk for bystander editing of the nearby coding bases.^31^^,^^32^ CRISPR reagent design is better defined for “standard” CRISPR-Cas9, where availability of protospacer adjacent motifs (PAMs) define the number of available gRNAs per target site. Together with a repair template, homology-directed repair (HDR) occurs at the dsDNA break, which can be used to correct virtually all single-nucleotide variants (SNVs) and small indels in the human genome.

In this study, we have developed a T cell editing platform that utilizes CRISPR-Cas9-mediated HDR and can correct SNV mutations in diverse IEIs with up to 80% efficiency. We have used the following model IEIs for this proof-of-concept and platform development study: STAT-1 gain-of function (STAT1-GOF) (*STAT1*, c.1163A>G, NM_007315.3, p.K388R), ADA2 deficiency (*ADA2, c.506G>A, NM_001282225.2,* p.R169Q), autoimmune polyendocrinopathy-candidiasis-ectodermal dystrophy (APECED) (*AIRE, c.769C>T, NM_000383.4,* p.R257X), and cartilage hair hypoplasia (CHH) (*RMRP, NR_003051.3,* c.A71G). During platform development, we investigated several strategies for HDR enhancement to obtain high mutation correction levels and functional improvement in the disease phenotype.

## Results

### gRNA design and repair strategy

**Figure 1:**
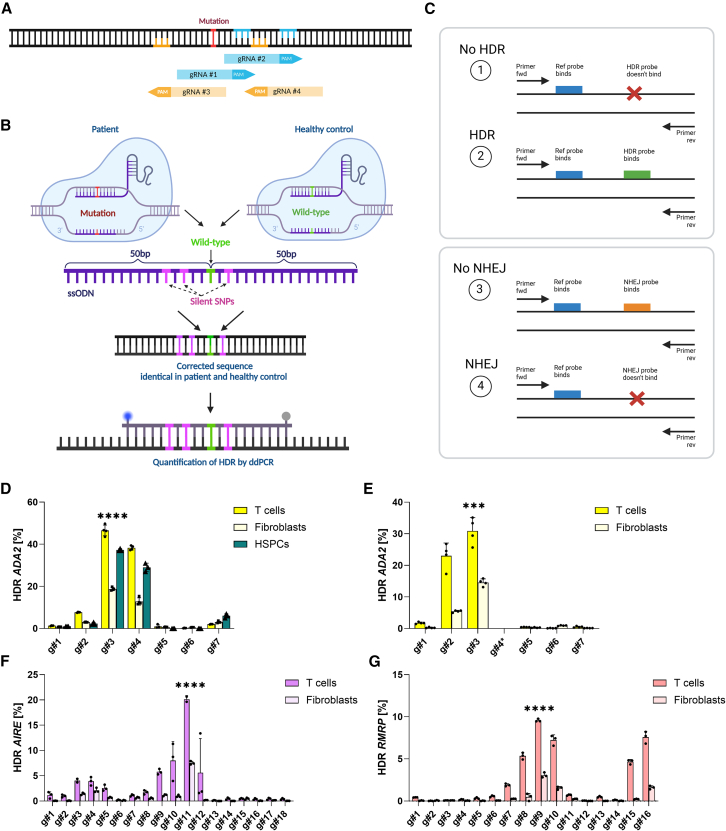
Repair strategy and gRNA screening in patient cells (A) Schematic representation of the gRNA screening strategy, where multiple gRNAs were assessed based on available PAM sites within the 100-bp ssODN area. Forward gRNAs and their PAMs are marked in blue and reverse in yellow. (B) Schematic representation of the repair strategy used in the study, where 100-bp ssODNs with ±50-bp homology arms from the mutation site (red) were used. ssODN design includes correction of the mutation (green) and 3–4 silent SNVs (pink), enabling identical editing strategy in patients and healthy controls and HDR detection by ddPCR. (C) Schematic representation of the ddPCR assay design for HDR and NHEJ detection. (D) ADA2 gRNA screening in HD T cells, fibroblasts and CD34^+^ HSPCs, assessed by ddPCR (*n* = 4 technical replicates for T cells and fibroblasts, *n* = 3 for HSPCs). (E) ADA2 gRNA screening in DADA2 patient T cells and fibroblasts, assessed by ddPCR (*n* = 3 technical replicates). ADA2 gRNA number 4 (asterisk) was not tested in patients due to PAM loss caused by the mutation. (F) AIRE gRNA screening in APECED patient T cells and fibroblasts, assessed by ddPCR (*n* = 3 technical replicates). (G) RMRP gRNA screening in CHH patient T cells and fibroblasts, assessed by ddPCR (*n* = 3 technical replicates). One independent experiment was performed for all sets of data. Statistical significance of best-performing gRNAs was assessed by one-way ANOVA with Fisher’s least significant difference (LSD) test, where ∗∗∗*p* < 0.0002 and ∗∗∗∗*p* < 0.0001. Bar denotes mean value, error bars represent ±SD.

gRNA design is crucial for the success of CRISPR experiments.^33^ As *ADA2* p.R169Q has no available base editing guides, and AIRE and RMRP guides can induce bystander base editing (Figures S1A–S1C), we designed “standard” CRISPR guides for these model loci. We included guides with cut sites located within the 100-bp repair template (7–18 guides per locus) (Figure 1A). To prevent CRISPR re-cutting^34^^,^^35^ and enable identical repair templates for healthy and patient cells, we designed single-stranded oligodeoxynucleotide (ssODN) repair templates, where 3–4 silent SNPs were added close to the mutation site (Figures 1B and S1D–S1F). This repair strategy also enabled rapid HDR detection in the edited samples by droplet digital PCR (ddPCR), with no differences between using an internal or external reference probe (Figures 1C, S2A, and S2B). Since *RMRP* encodes a non-coding RNA, we could not design silent SNPs to the locus and thus knocked in two variants of unknown function during the early optimization experiments (Figures S1F and S1G). In later studies, we corrected only the pathogenic variant (Figure S1H).

We first tested the correction strategy in the *ADA2* locus in healthy control T cells, fibroblasts, and CD34^+^ HSPCs isolated from umbilical cord blood and compared the results to similar screens in deficiency of ADA2 (DADA2) patient T cells and fibroblasts (Figures 1D and 1E; all patients are homozygous for the *ADA2* p.R169Q mutation). We identified gRNA number 3 as the best guide for *ADA2* correction across cell types, with ∼30% maximum HDR efficiency (Figure 1E). We then screened guides for *AIRE* and *RMRP* loci in homozygous patient T cells and fibroblasts and identified AIRE gRNA number 11 and RMRP gRNA number 9 as the best guides (Figures 1F and 1G, 10%–20% HDR). We assessed HDR in the samples also by deep amplicon sequencing with near-identical results (Figures S2C–S2E), confirming ddPCR as a reliable method for rapid HDR assessment. We did not observe a clear correlation between the HDR frequency and guide cutting distance from the mutation site (Figures S2F–S2H), possibly due to the sequence context and structural or thermodynamic properties of the tested gRNAs.^33^^,^^36^ *In silico* gRNA design tools showed poor accuracy with this correction strategy, likely as they are built on datasets adapted for non-homologous end joining (NHEJ)-based gene knockout (Figures S2I–S2K).

### Optimized T cell culture for editing enhancement

**Figure 2:**
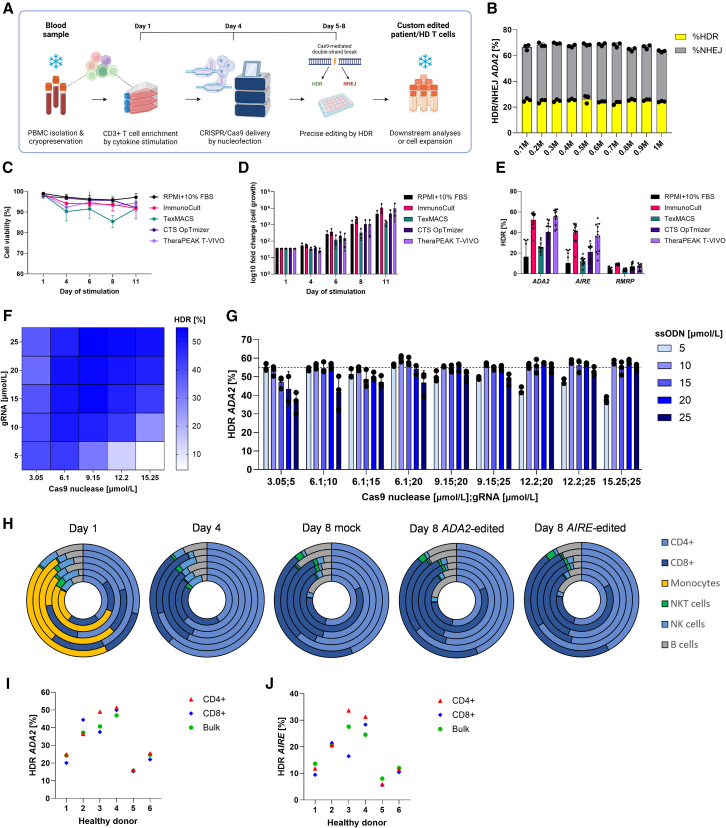
Establishment and assessment of CRISPR-Cas9 T cell editing platform (A) Schematic representation of the CRISPR-Cas9 T cell editing platform. PBMCs from patient and HD blood samples are first isolated and cryopreserved. PBMCs are thawed on day 1 and stimulated for 3 days with interleukins: IL-2 (120 U/mL), IL-7 (3 ng/μL), and IL-15 (3 ng/μL) and soluble CD3/CD28 (15 μL/mL), which activate and induce expansion of CD3^+^ T cells. Cells are nucleofected on day 4 with custom CRISPR reagents (gRNA, Cas9 nuclease, ssODN). Afterward, cells are cultured for 4 days in IL-2 (250 U/mL), during which Cas9-mediated double-stranded breaks are repaired by HDR/NHEJ. On day 8, cells are harvested for downstream assays, expanded further, or cryopreserved. (B) *ADA2* HDR and NHEJ editing in HD T cells with 0.1–1 M nucleofected cells/sample, measured by ddPCR (*n* = 3 technical replicates). Comparison of different T cell culture media during 11-day cytokine stimulation, assessed by (C) T cell viability (dots represent mean of *n* = 3 biological replicates), (D) T cell fold change (*n* = 3 biological replicates), and (E) *ADA2*, *AIRE,* and *RMRP* HDR editing on day 8 (*n* = 3 technical ddPCR replicates from *n* = 3 biological replicates). (F) *ADA2* HDR editing in HD T cells with Cas9 nuclease at 3.05–15.25 μmol/L/sample, gRNA at 5–25 μmol/L/sample, and ssODN at 5 μmol/L/sample/sample, measured by ddPCR (*n* = 3 technical replicates). (G) *ADA2* HDR editing in HD T cells with selected Cas9-gRNA concentrations and ssODN at 5–25 μmol/L/sample, measured by ddPCR (*n* = 3 technical replicates). Dashed line indicates mean of Cas9 nuclease at 3.05 μmol/L/sample, gRNA at 5 μmol/L/sample, and ssODN at 5 μmol/L/sample. (H) Frequency of immune cells (CD4^+^, CD8^+^, monocytes, NKT cells, NK cells, B cells) in six HDs on days 1, 4, and 8 (mock, *ADA2* edited, or *AIRE* edited) of the platform, assessed by flow cytometry. Each ring of the doughnut plot represents one HD. HDR editing levels in CD4^+^ and CD8^+^ and the bulk of cells for *ADA2* (I) and *AIRE* (J) on day 8, measured by ddPCR (*n* = 1 technical replicate). One independent experiment was performed for all sets of data except for (C)−(E), where data from three donors are shown in the graphs, and (F) and (G), where one out of three representative experiments is shown. Bar denotes mean value, error bars represent ±SD.

HDR-dependent correction happens in the S/G2 phases of the cell cycle.^37^ Therefore, optimal T cell expansion and viability can further increase gene correction.^38^ As a baseline, we used common T cell editing protocols and our previous work,^39^^,^^40^^,^^41^ where peripheral blood mononuclear cells (PBMCs) are first stimulated for 3 days, then nucleofected with CRISPR-Cas9 ribonucleoprotein complexes (RNPs), and collected for DNA extraction 3–5 days post-nucleofection (Figure 2A). We noted ∼15%–30% *ADA2* and ∼5%–8% *AIRE* HDR editing in healthy controls, with editing levels plateauing 3 (*ADA2*) and 2 (*AIRE*) days after nucleofection and staying consistent for up to 14 days after nucleofection (Figures S3A and S3B). The number of cells used for nucleofection had no effect on the final editing level, allowing us to work with less material when necessary (Figure 2B). We thus settled for 0.5–1 million cells per nucleofection and standardized sample collection on day 4 post-nucleofection.

To improve T cell proliferation, viability, and HDR, we compared several GMP-compatible T cell media while editing *ADA2*, *AIRE*, and *RMRP* loci (Figures 2C–2E). Based on the results across tested donors, Immunocult and TheraPEAK T-VIVO performed similarly. Since Immunocult supported cell proliferation earlier, we selected it as the basal medium and supplemented it with 120 U/mL interleukin-2 (IL-2), 3 ng/μL IL-7, 3 ng/μL IL-15, and 15 μL/mL soluble CD3/CD28 T cell activator to obtain the T cell stimulation cocktail for selective CD3^+^ T cell expansion from PBMCs. We also titrated the concentrations of Cas9 nuclease, gRNA, and repair template, with the goal of reaching optimal reagent concentration in the nucleus without excessive toxicity (Figures 2F, 2G, S3C, and S3D). Based on the results, we standardized Cas9 nuclease at 3.05, gRNA at 5, and ssODN at 5 μmol/L per nucleofected sample.

To verify selective CD3^+^ T cell expansion from PBMCs, we quantified the immune cell populations on culture days 1, 4, and 8 from 6 healthy controls by flow cytometry (Figure 2H). While PBMC population diversity is considerable on day 1, it gradually disappears during cytokine stimulation. By day 8, CD4^+^ and CD8^+^ T cells make up ∼80% of all cells. On day 8, it is possible to sort, cryopreserve, or further expand the T cells. Although we noted interindividual and locus-specific variation in editing efficiency, also described by others,^38^^,^^41^^,^^42^^,^^43^ HDR editing levels in CD4^+^ and CD8^+^ subsets were similar within each donor (Figures 2I and 2J).

### Refined repair template design for HDR enhancement

**Figure 3:**
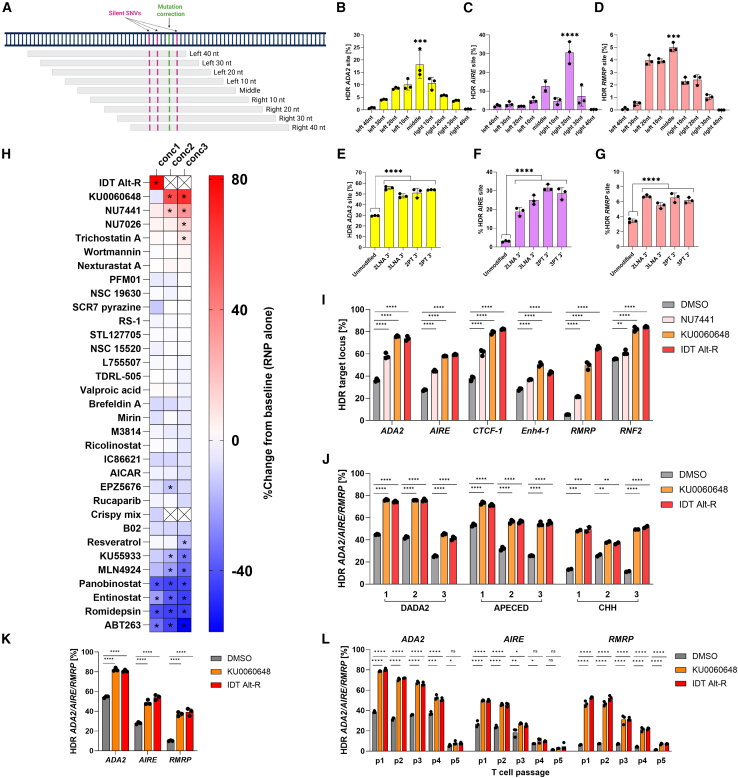
HDR enhancement in healthy control and patient T cells (A) Schematic representation of asymmetric ssODN designs with 10- to 90-bp homology arms on either side from target site. HDR editing with asymmetric ssODNs in HD T cells for (B) *ADA2*, (C) *AIRE*, and (D) *RMRP*, measured by ddPCR (*n* = 3 technical replicates). HDR editing with 3′ LNA- or 3′ PT-modified, position-optimized ssODNs in HD T cells for (E) *ADA2*, (F) *AIRE*, and (G) *RMRP*, measured by ddPCR (*n* = 3 technical replicates). (H) Validation of HDR-enhancing compounds at three concentrations in increasing order (conc1–3) for *ADA2* editing in HD T cells, assessed by ddPCR (*n* = 2 technical replicates per condition). Compounds were assessed in three HDs, where mean of all donors per condition were compared to the mean of edited DMSO-treated baseline. Statistical significance was assessed by ANOVA. For the heatmap, percentage of HDR fold change from baseline was calculated for each compound concentration. Statistically significant concentrations are indicated with black asterisks. Conc2–3 are marked with a cross for those compounds that were assessed at one concentration. (I) HDR editing for *ADA2*, *AIRE*, *CTCF1*, *Enh4-1*, *RMRP*, and *RNF2*, measured by ddPCR (*n* = 3 technical replicates) with selected HDR-enhancing compounds (4 μM NU7441, 0.5 μM KU0060648, and 1 μM IDT Alt-R enhancer V2) or DMSO in HD T cells. (J) HDR in DADA2, APECED, and CHH patient T cells with concentration-optimized HDR enhancing compounds (0.5 μM KU0060648 and 0.6 μM IDT Alt-R enhancer V2), where *ADA2*, *AIRE*, and *RMRP* loci, respectively, were corrected. HDR levels were assessed by ddPCR for *ADA2* and *AIRE* (*n* = 3 technical replicates) and by amplicon sequencing for *RMRP* (*n* = 2 technical replicates). (K) *ADA2*, *AIRE*, and *RMRP* HDR editing in HD CD34^+^ HSPCs, measured by ddPCR (*n* = 3 technical replicates) with concentration-optimized HDR enhancing compounds (0.5 μM KU0060648 and 0.6 μM IDT Alt-R enhancer V2) or DMSO. (L) HDR editing in *ADA2*, *AIRE*, and *RMRP* in HD T cells at different cell passages (p1–p5), measured by ddPCR (*n* = 3 technical replicates) with concentration-optimized HDR enhancing compounds (0.5 μM KU0060648 and 0.6 μM IDT Alt-R enhancer V2) or DMSO. Three independent experiments were performed for all sets of data where representative experiment is shown, except for (H) where average measurements from three HDs is shown and (J) where all patients are shown in the graph. Bar denotes mean value, error bars represent ±SD. Statistical significance for all sets of data, except (H), was assessed by one-way ANOVA with Fisher’s LSD test, where ∗*p* < 0.01, ∗∗*p* < 0.001, ∗∗∗*p* < 0.0002, and ∗∗∗∗*p* < 0.0001.

Positioning and format of the repair template affects HDR editing.^35^^,^^44^^,^^45^^,^^46^ For optimal repair template positioning, we designed asymmetric 100-bp templates for *ADA2*, *AIRE*, and *RMRP* loci, with 10- to 90-bp homology arms on either side (9 templates per locus, all reverse complementary to the guide; Figure 3A).^44^ The symmetric templates with 50-bp homology arms proved best for *ADA2* and *RMRP*; however, *AIRE* locus edited most optimally with an asymmetric template (30 bp left homology arm, 70 bp right homology arm, Figures 3B–3D).

Coupling the repair template to Cas9 improves HDR editing, presumably by enhancing nuclear import and template positioning at the cut site.^47^^,^^48^ To test this, we synthesized 5′ benzylguanine (BG)-coupled repair templates that can bind covalently to Cas9-SNAP fusion protein.^48^^,^^49^ BG templates led to 2-fold *ADA2* HDR enhancement in fibroblasts and T cells with both Cas9-wild type (WT) and Cas9-SNAP RNPs (Figures S4A–S4C), suggesting alternative HDR enhancing mechanisms independent of the Cas9-SNAP coupling. The commercially available 3′ phosphorothioate (PT) and locked nucleic acid (LNA) modifications led to similar HDR improvements in T cells, fibroblasts, and CD34^+^ HSPCs (Figures 3E–3G and S4D–S4F). 3′ PT and LNA modifications likely protect the ssODN from 3′ endonucleases such as TREX1,^50^ resulting in increased stability of the oligo and enhanced HDR. We chose 2PT 3′ modified repair templates for further experiments due to their universal effectiveness and ease of synthesis (Figures S4G–S4I).

### Inhibition of DNA-PKcs further improves HDR

A substantial number of HDR-enhancing chemicals have been published. We reviewed 33 compounds convincingly reported as HDR enhancers (Table S12) and tested them in the *ADA2* locus in healthy control T cells at three concentrations based on previously reported effective concentrations in cell lines and primary cells. Most compounds decreased HDR, likely due to cell toxicity. Three compounds led up to 80% efficiencies in screening conditions (Figure 3H): the DNA-dependent protein kinase catalytic subunit (DNA-PKcs) inhibitors NU7441^51^ and KU0060648^51^ and Integrated DNA Technologies (IDT) Alt-R enhancer V2 (hereafter referred to as IDT Alt-R; compound identity undisclosed). We validated these three compounds in six endogenous loci (*ADA2*, *AIRE*, *CTCF-1*, *Enh4-1*, *RMRP*, and *RNF2*), optimized their concentrations in healthy controls, and tested them further in DADA2, APECED, and CHH patient T cells, consistently achieving minimal toxicity, ∼2-fold improvement, and up to 80% mutation correction, depending on the target locus and individual (Figures 3I, 3J, S4J, and S4K). The compounds improved editing even in CD34^+^ HSPCs derived from healthy donor umbilical cord blood (Figure 3K). High HDR levels were maintained when nucleofection was performed at passages 1–3, decreasing in later passages (Figure 3L). Increased HDR levels were also observed in patient and healthy control samples analyzed by amplicon sequencing (Figures S5 and S6).

As HDR is dependent on the S/G2 phases of the cell cycle,^37^ we also tested a set of cell-cycle inhibitors (Table S13) for their ability to synchronize editing to S/G2 phases and consequently increase HDR. Hydroxyurea^52^ emerged as an unexpected HDR enhancer when applied 24 h before nucleofection, but because the effect was suboptimal in comparison to IDT Alt-R, we did not explore the strategy further (Figures S4L and S4M).

### Adapted GUIDE-seq off-target profiling for patient and healthy control T cells

Genome-wide, unbiased identification of double-strand breaks enabled by sequencing (GUIDE-seq) finds CRISPR off-target cuts by transfecting cells with modified dsDNA oligos (dsODNs) along the CRISPR RNP complex, and then selectively amplifying and sequencing the oligo integration sites.^53^ The existing GUIDE-seq data mainly come from cell lines.^53^ There are reports for adaptations to T cells,^39^^,^^54^ but since dsODNs can be particularly toxic to patient T cells, we started the off-target profiling by optimizing the dsODN concentration for improved cell viability. Experiments in healthy control T cells for guides targeting the *ADA2* and *HEK-site 4* loci (positive control guide with multiple off-targets^53^) showed acceptable cell viabilities and optimal dsODN integration with dsODN at 1–5 μmol/L per nucleofected sample (Figures S7A–S7F). Subsequent deep sequencing detected no off-targets for ADA2 guide but recovered several integrants for HEK-site 4, validating the sensitivity of the method (Figures S7C and S7F).

**Figure 4:**
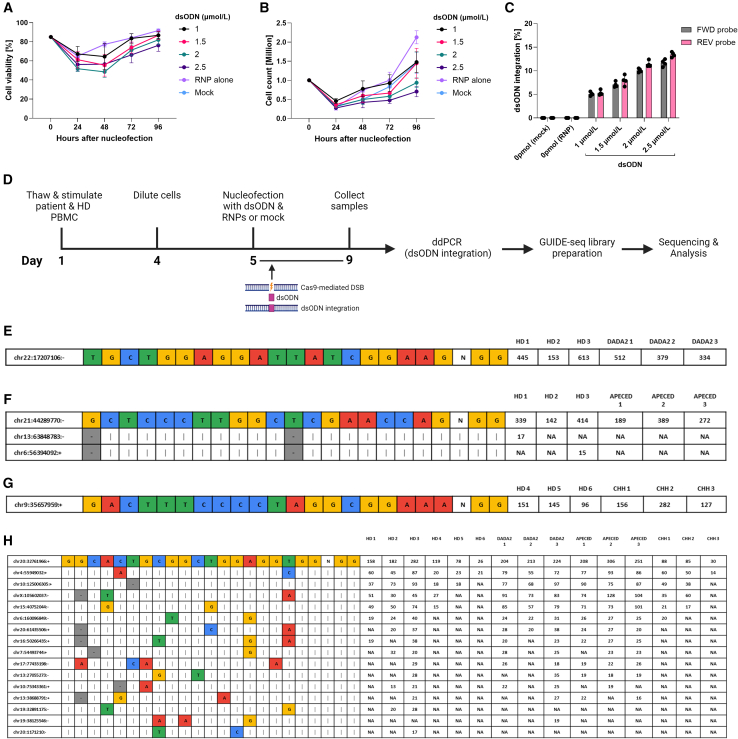
gRNA off-target profiling by GUIDE-seq in patient and healthy control T cells DADA2 patient T cell (A) viability (*n* = 4 technical replicates) and (B) count (*n* = 4 technical replicates) 24–96 h after nucleofection with 0–2.5 μmol/L/sample dsODN and ADA2 RNPs. (C) dsODN integration 96 h after nucleofection in DADA2 patient T cells, with 0–2.5 μmol/L/sample dsODN and ADA2 RNPs, assessed by ddPCR (*n* = 3 technical replicates) using forward (gray) and reverse (pink) dsODN probes for detection. (D) Schematic representation of the GUIDE-seq experiment. DADA2, APECED, and CHH patient and HD PBMCs were thawed and stimulated with IL-2 (120 U/mL), IL-7 (3 ng/μL), IL-15 (3 ng/μL), and soluble CD3/CD28 (15 μL/mL) on day 1, diluted on day 4, and nucleofected on day 5 with 1.5 μmol/L/sample dsODN and selected RNPs or mock. Cells were cultured in IL-2 (250 U/mL) until sample collection on day 9, followed by genomic DNA (gDNA) extraction, ddPCR for dsODN integration, and GUIDE-seq library preparation. GUIDE-seq in patient and HD T cells for (E) ADA2 gRNA number 3, (F) AIRE gRNA number 11, (G) RMRP gRNA number 9, and (H) HEK-site 4 gRNA, targeting the endogenous human embryonic kidney *HEK-site 4*. GUIDE-seq results are shown as mismatch plots, where the on-target sequence is depicted at the first line of the table with sequencing read counts per individual (right). The most abundant off-targets, if applicable, are listed under the target with their corresponding locations in the genome (left) and sequencing read counts (right). One independent experiment was performed for all sets of data. Bar denotes mean value, error bars represent ±SD.

To account for increased dsODN toxicity in patient T cells, we refined the dsODN concentration further in DADA2 patient T cells, settling on dsODN at 1.5 μmol/L per nucleofected sample based on cell viability, dsODN integration, and cell yield (Figures 4A–4C). Finally, we performed GUIDE-seq in three patients and three healthy controls for each locus and confirmed the safety of ADA2 gRNA number 3, AIRE gRNA number 11, and RMRP gRNA number 9 with no off-targets, contrasting with multiple off-targets for *HEK-site 4* (Figures 4D–4H). To summarize, we present a refined GUIDE-seq protocol for T cell CRISPR-Cas9 off-target profiling and recommend lower dsODN concentrations for IEI patient samples to reach optimal cell viability and reliable sequencing results.

### Long-read sequencing and single-cell transcriptomics reveal no aberrant changes in the karyotype, transcriptome, and T cell receptor repertoire of edited T cells

CRISPR-Cas9 can cause various chromosomal aberrations,^55^^,^^56^ which increase the risk for malignant transformation and complicate clinical translation of genome editing. To evaluate the translational potential of our HDR enhancement strategies, we performed a comprehensive safety assessment using state-of-art technologies for genomic and transcriptomic analysis of the edited T cells.

We first performed PacBio long-read sequencing to map unintended edits in DADA2 and healthy control T cells. We edited the cells with or without KU0060648 and harvested cells 6 days post-nucleofection (Figures S8A and S8B; detailed visualization of the cut site is available in Figure S8C). We quantified a mean coverage of 25× across the genome, with 4/31 (∼47%, RNP only) and 12/18 (∼67%, RNP^+^ KU0060648) reads containing the desired edit, respectively. We noted additional on-target indels between ∼3 and 300 bp, and a ∼1.2-kb on-target deletion in one HiFi read in the KU0060648-treated sample. Only one read per edited sample contained no on-target alterations, demonstrating that virtually all cells had been exposed to editing reagents. We did not find any chromosomal translocations or integrated repair template concatemers at the intended cut site or elsewhere in the genome (Figure S8C); however, increased sequencing depth might uncover additional low-frequency events. SNVs, small insertions or deletions outside the cut site, were shared between the experimental conditions and were not suggestive of aberrant mutational signatures or indicative of cancer (Figures S8D and S9A).^57^

**Figure 5:**
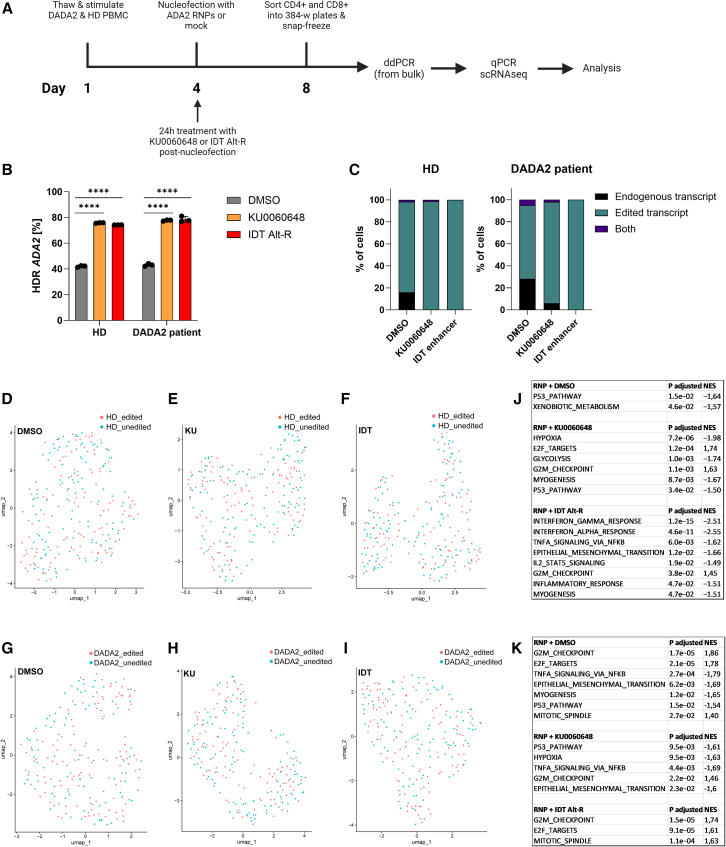
scRNA-seq assessment of CRISPR-Cas9 and HDR-enhancing compounds in DADA2 patient and HD T cells (A) Outline of the experiment. HD and DADA2 patient PBMCs were thawed and stimulated with IL-2 (120 U/mL), IL-7 (3 ng/μL), IL-15 (3 ng/μL), and soluble CD3/CD28 (15 μL/mL) on day 1 and nucleofected on day 4 with ADA2 CRISPR RNPs or mock. Cells were cultured in IL-2 (250 U/mL) and HDR enhancers (0.5 μM KU0060648, 0.6 μM IDT Alt-R enhancer V2) or DMSO for 24 h after nucleofection and IL-2 alone afterward. On day 8, 64 CD4^+^ and 64 CD8^+^ T cells per condition (128 cells in total per condition) were sorted into 384-well plates, and gDNA was extracted from the bulk for ddPCR. Sorted cells were further analyzed with RT-qPCR and scRNA-seq. (B) *ADA2* HDR editing in HD and DADA2 patients on day 8, assessed by ddPCR (*n* = 3 technical replicates). (C) ADA2 editing in HD and DADA2 patients, assessed by RT-qPCR of the scRNA-seq libraries with probes to the corrected and uncorrected nucleotide sequence. For HD, 56, 75, and 77 cells were analyzed for DMSO, KU0060648, and IDT Alt-R enhancer V2-treated cells, respectively. For DADA2 patients, 39, 50, and 50 cells were analyzed for DMSO, KU0060648, and IDT Alt-R enhancer V2-treated cells, respectively. Uniform manifold approximation and projection (UMAP) plots generated from scRNA-seq for *ADA2*-edited HD treated with (D) DMSO, (E) KU0060648, and (F) IDT Alt-R enhancer V2, compared to unedited HD (DMSO). UMAP plots of corrected DADA2 patient treated with (G) DMSO, (H) KU0060648 and (I) IDT Alt-R enhancer V2, compared to uncorrected DADA2 patient (DMSO). (J) Hallmark gene set enrichment results for *ADA2*-edited HD (DMSO, KU0060648, and IDT Alt-R enhancer V2) compared to unedited HD (DMSO). (K) Hallmark gene set enrichment results for corrected DADA2 patient (DMSO, KU0060648, and IDT Alt-R enhancer V2) compared to uncorrected DADA2 patient. One independent experiment was performed for all sets of data. Bar denotes mean value, error bars represent ±SD. Statistical significance for HDR editing in (B) was assessed by one-way ANOVA with Fisher’s LSD test, where ∗∗∗∗*p* < 0.0001. NES, normalized enrichment score.

Next, we performed single-cell RNA sequencing (scRNA-seq) of full-length mRNA to search for karyotypic and transcriptomic changes. DADA2 patient and matched healthy control T cells were nucleofected with or without ADA2 RNPs and treated with HDR enhancers (KU0060648, IDT-Alt-R) or DMSO (total of six treatment groups; Figures 5A and 5B). Four days post-nucleofection, we sorted equal amounts of single CD4^+^ and CD8^+^ cells in plates for library preparation (Figure 5A). Cultures exposed to HDR enhancers had a slight underrepresentation of CD4^+^ cells and an overrepresentation of CD8^+^ cells (Figures S10A and S10B). scRNA quantitative reverse transcription-PCR (RT-qPCR) detected the presence of the corrected RNA transcript in ∼80% of the RNP-treated and >98% of the HDR enhancer-treated cells, indicating that nearly all cells had been exposed to editing reagents and harbored at least one corrected allele (Figures 5C, S11A, and S11B).

The scRNA-seq data showed minimal effect of editing on the general transcriptomic profile as the edited cells clustered with unedited cells in both the control and DADA2 patient (Figures 5D–5K). The samples edited with the presence of DMSO and KU0060648 showed a slight downregulation of the p53 response, likely as an adaptation to the transient p53 upregulation^41^ when the *ADA2* gene was cut. KU0060648 also affected metabolism slightly, likely due to the compound’s bystander effect on phosphatidylinositol 3-kinase.^58^ IDT Alt-R showed a downregulation of immune response pathways in the healthy control (Figure 5J). In addition, all samples recovered low-frequency non-recurring novel fusion transcripts (Table S18). Fusion transcripts that mapped to genes in chromosome 22 (where *ADA2* resides) were not found in >1 cell per condition. scRNA-seq data showed no loss of heterozygosity, indicative of no identifiable loss of chromosomal material. In addition, the T cell repertoire was polyclonal, and editing did not diminish the T cell receptor diversity (Figures S12A and S12B).

To conclude, long-read sequencing and single-cell transcriptomics demonstrate that genome-edited healthy control and DADA2 patient T cells cultured with or without NHEJ inhibitors do not display identifiable structural variations, transcriptome, and T cell receptor repertoire.

### Functional consequences of *ADA2* correction on the transcriptome and proteome

DADA2 is a complex autoinflammatory disease with multiple affected immune subsets, including T cells.^59^ The disease hallmark is enhanced interferon-γ (IFN-γ) and tumor necrosis factor α (TNF-α) signaling. We compared T cell transcriptomes in unedited DADA2 patients and healthy controls. We found that they clustered separately and noted enhanced TNF-α signaling in the DADA2 patient (Figures S13A–S13D), suggesting that T cells can, with limitations, be used to model the disease pathology. Somewhat unexpectedly, patient T cell transcriptomes also indicated downregulation of IFN-α and IFN-γ responses.

As expected, 4 days after *ADA2* correction, we saw downregulation of TNF-α signaling in samples corrected in the presence of DMSO or KU0060648. The effects were not visible in cells corrected with IDT Alt-R, possibly due to the compound interfering with immune signaling pathways as seen in the healthy control (Figures 5J and 5K). The corrected DADA2 T cell transcriptomes continued to cluster with uncorrected cells. The corrected cells will likely need longer culture and re-stimulation with appropriate cytokines to show a noticeable shift toward a “healthy” T cell state.

**Figure 6:**
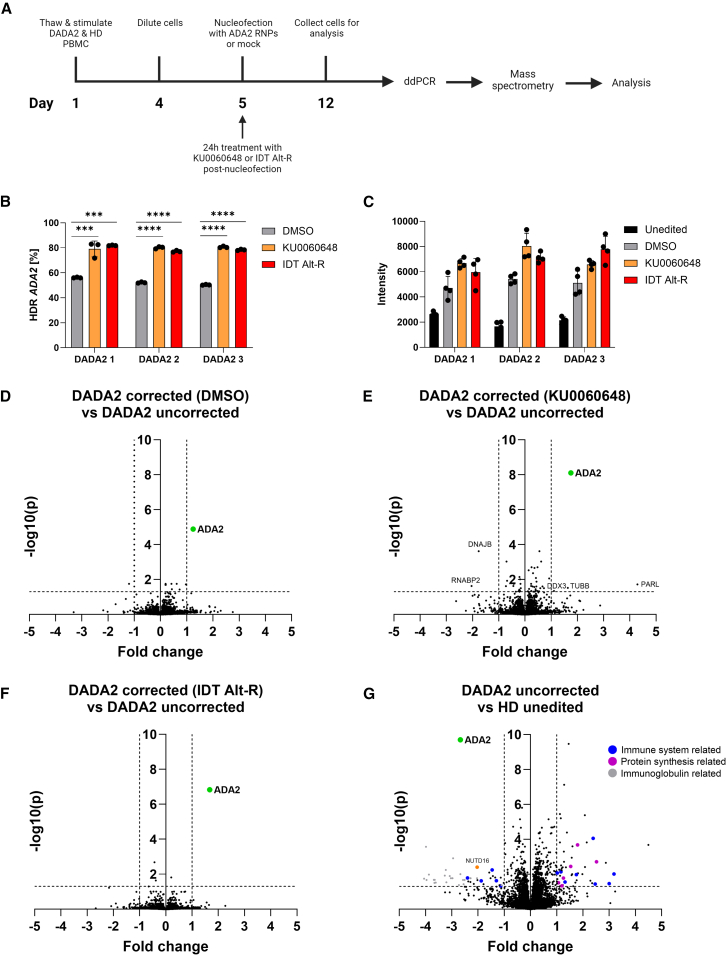
Mass spectrometry analysis of corrected and uncorrected DADA2 patient T cells (A) Outline of the experiment. DADA2 patient and HD PBMCs were thawed and stimulated with IL-2 (120 U/mL), IL-7 (3 ng/μL), IL-15 (3 ng/μL), and soluble CD3/CD28 (15 μL/mL) on day 1 and diluted on day 4 for further expansion. Cells were nucleofected with ADA2 CRISPR RNPs or mock on day 5 and cultured in IL-2 (250 U/mL) and HDR enhancers (0.5 μM KU0060648 and 0.6 μM IDT Alt-R enhancer V2) or DMSO for 24 h. Afterward, cells were cultured in IL-2 (250 U/mL) until sample collection on day 12. (B) *ADA2* HDR editing in three DADA2 patients (DADA2 1–3) treated with HDR enhancers or DMSO, assessed by ddPCR (*n* = 3 technical replicates). (C) Abundance of ADA2 protein in DADA2 patients, reported as intensities (*n* = 4 technical replicates). Comparison of protein expression levels in (D) *ADA2*-corrected (DMSO) DADA2 patients to uncorrected DADA2 patients, (E) *ADA2*-corrected (KU0060648) DADA2 patients to uncorrected DADA2 patients, (F) *ADA2*-corrected (IDT Alt-R enhancer V2) DADA2 patients to uncorrected DADA2 patients, and (G) uncorrected DADA2 patients to unedited HDs, assessed by mass spectrometry. For (D)–(G), volcano plots were created by reporting protein expression fold change from mean of three DADA2 patients and three HDs on the *x* axis and –log10 *p* value on the *y* axis. One independent experiment was performed for all sets of data. Statistical significance was assessed by one-way ANOVA with Fisher’s LSD test, where ∗*p* < 0.05, ∗∗*p* < 0.01, ∗∗∗*p* < 0.0002, and∗∗∗∗*p* < 0.0001. Bar denotes mean value, error bars represent ±SD. DDX3, ATP-dependent RNA helicase; DNAJB, DnaJ homolog subfamily B; NUDT, U8 snoRNA-decapping enzyme; PARL, presenilin-associated rhomboid-like protein; RNABP2, E3 SUMO-protein ligase RanBP2; TUBB, tubulin beta.

To finalize safety and functional profiling, we analyzed the proteomes of edited and unedited DADA2 and healthy control T cells by mass spectrometry (MS) (Tables S18, S19, and S20). Cells were collected 7 days post-nucleofection, and genomic editing was confirmed with ddPCR (Figures 6A, 6B, and S14A). We saw low but detectable ADA2 expression in patients when all MS data-independent acquisition (DIA) runs were searched together (Figure 6C; Table S19); however, when searched alone, no ADA2 was detected, suggesting very low to no ADA2 expression in the patients. Editing increased ADA2 expression up to 2-fold in corrected DADA2 T cells (Figures 6C–6F). In healthy controls, ADA2 expression generally decreased upon editing, either due to on-target NHEJ deletions or the addition of silent SNVs (Figure S14B).

Other than the changes in ADA2 expression, we found no significant proteomic alterations in samples edited without enhancers (Figures 6D and S14C). In samples edited with HDR enhancers, gene set enrichment analysis^60^^,^^61^ identified minor alterations without clear clustering to pathways (Figures 6E, 6F, S14D, and S14E). IDT Alt-R-treated, *ADA2*-edited healthy control cells showed more altered proteins (Figure S14E; Table S19), which we did not investigate further as the identity of the compound is undisclosed.

When comparing unedited DADA2 patients and healthy controls, DADA2 patients showed downregulation of several proteins implicated in inflammatory response, as well as decreased expression of the mRNA decapping enzyme NUTD16 (Figure 6G; Tables S20 and S21). Consequently, the proteins of the translational machinery were upregulated, along with several adaptive immune response proteins (Table S21). We also detected cytoplasmic immunoglobulins, which we attribute to residual B cells in the samples, as we saw no immunoglobulin transcripts in the scRNA-seq data where T cells were pre-sorted using flow cytometry.

To conclude, we observed ADA2 protein expression and downregulation of TNF-α signaling in corrected DADA2 patient proteomes and transcriptomes, with minimal persisting interference from the KU0060648 compound.

### Gene correction improves T cell proliferation in CHH

Mutations in *RMRP* cause CHH, a syndromic immunodeficiency with defective T cell proliferation.^62^ We thus evaluated the patient T cell proliferative capacity in response to mutation correction. We further hypothesized that corrected patient T cells would outgrow their uncorrected counterparts, and consequently the frequency of corrected alleles would increase in DNA samples taken during prolonged CHH T cell culture.

**Figure 7:**
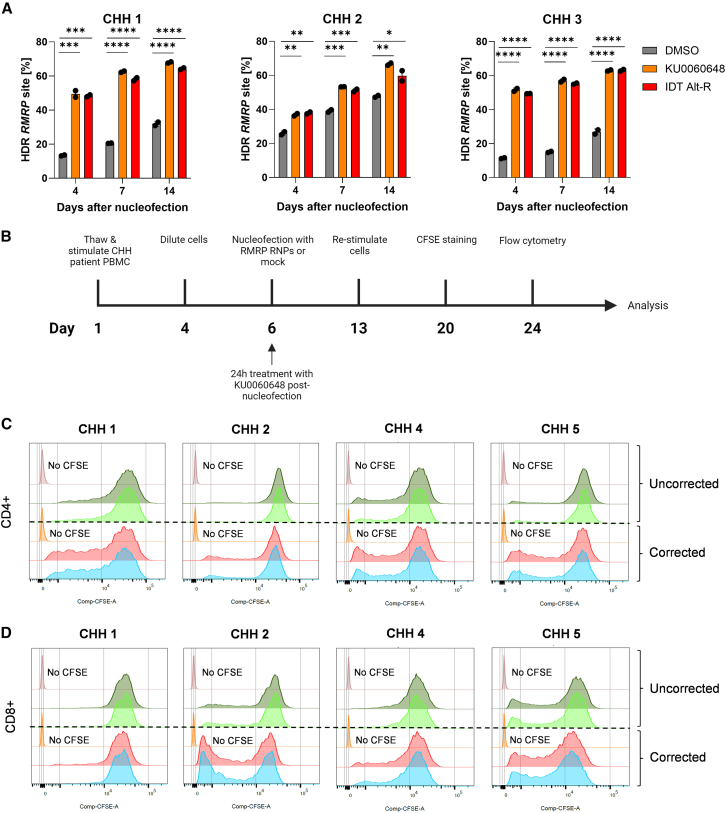
T cell proliferation assay in cartilage-hair hypoplasia patients (A) *RMRP* HDR editing in three cartilage-hair hypoplasia (CHH) patients (CHH 1–3) 4, 7, and 14 days after nucleofection with concentration-optimized HDR enhancing compounds (0.5 μM KU0060648 and 0.6 μM IDT Alt-R enhancer V2) or DMSO. HDR was assessed by amplicon sequencing (*n* = 2 technical replicates). (B) Outline of the CFSE-based T cell proliferation experiment. CHH patient PBMCs were thawed and stimulated with IL-2 (120 U/mL), IL-7 (3 ng/μL), IL-15 (3 ng/μL), and soluble CD3/CD28 (15 μL/mL) on day 1 and diluted on day 4 for further expansion. Cells were nucleofected with CRISPR RNPs for RMRP correction or mock on day 6 and cultured in IL-2 (250 U/mL) and 0.5 μM KU0060648 for 24 h after nucleofection. Afterward, cells were cultured in IL-2 (250 U/mL) until re-stimulation on day 13 with the same setup as on day 1. Cells were stained with CFSE on day 20 and cultured in IL-2 (250 U/mL) for 4 days. On day 24, cells were stained for flow cytometry. T cell proliferation in corrected and uncorrected CHH patients for (C) CD4^+^ and (D) CD8^+^ T cells, assessed by flow cytometry. One independent experiment was performed for all sets of data. The patient number corresponds to patient information in Table S15. Bar denotes mean value, error bars represent ±SD. Statistical significance was assessed by one-way ANOVA with Fisher’s LSD test, where ∗*p* < 0.05, ∗∗*p* < 0.01, ∗∗∗*p* < 0.0002, and ∗∗∗∗*p* < 0.0001.

To test this, we first corrected *RMRP* in T cells from three CHH patients and measured HDR correction levels at 4, 7, and 14 days post-nucleofection by amplicon sequencing (Figure 7A). We noted an up to 50% correction at day 4, which increased to 70% at 14 days post-nucleofection, with individual variation and diverse representation of small indels in the samples (Figures S15–S17). Consequently, we chose to assess T cell proliferative capacity 14 days post-nucleofection and enhance *RMRP* correction by treating cells with KU0060648 for the first 24 h after nucleofection, as we observed no increased toxicity from NHEJ inhibition (Figure S4K). We performed carboxyfluorescein succinimidyl ester (CFSE)-based T cell proliferation assay in four corrected and uncorrected CHH patients 14 days after nucleofection (day 20 in cell culture) (Figure 7B). We also assessed a healthy control CD4^+^ and CD8^+^ T cells 14 days after mock nucleofection from the same experimental pipeline as a technical positive control for the assay (Figures S18A and S18B). Unstimulated PBMCs were used as a technical negative control for CD4^+^ and CD8^+^ T cell proliferation (Figures S18C and S18D). We saw significant improvement in proliferation of corrected CD4^+^ T cells compared to that of uncorrected cells in all four patients (Figures 7C and S18E). Similarly, we saw significant improvement in CD8^+^ T cell proliferation upon mutation correction in all but one patient (Figures 7D and S18F). In conclusion, genomic correction of *RMRP* enhances T cell proliferation, leading to selective growth advantage for the corrected cells.

### Gene correction reduces STAT1 hyperphosphorylation in STAT1-GOF patients

**Figure 8:**
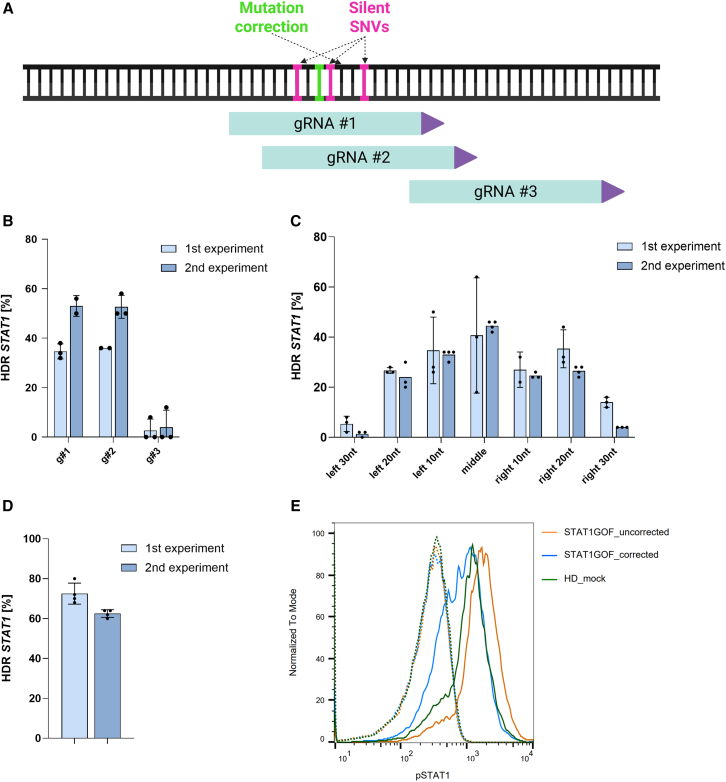
Assessment of STAT1 phosphorylation in corrected and uncorrected STAT1-GOF patients (A) Schematic representation STAT1 gRNA design and repair strategy. Correction of pathogenic mutation is marked with green and silent SNVs in pink. Three gRNAs were designed (green), where the PAM site is represented as an arrow (purple). (B) STAT1 gRNA screening in STAT1-GOF patient T cells assessed by measuring HDR editing using ddPCR (*n* = 3 technical replicates) in two independent experiments, indicated in light and dark blue. (C) 3′ PT modified asymmetric ssODNs screening with best-performing guide (g number 2) in STAT1-GOF patient T cells assessed by measuring HDR editing using ddPCR (*n* = 3 technical replicates) in two independent experiments, indicated in light and dark blue. (D) *STAT1* HDR editing in T cells from STAT1-GOF patient 7 days after nucleofection with optimized RNP was assessed by ddPCR (*n* = 2 technical replicates) in two independent experiments, indicated in light and dark blue. The ddPCR readouts in (B)–(D) are reported as twice the measured value as the mutation is heterozygous and uncorrected allele is present at the time of assessment. (E) The cells obtained on 7 days post-nucleofection from the second experiment in (D) were also stimulated with IFN-α, followed by assessment of phosphorylated STAT1 levels in CD3^+^ cells using flow cytometry. Mock electroporated patient T cells and healthy donor T cells that did not receive CRISPR RNPs were used as controls. The dotted line and the solid line show unstimulated and stimulated samples, respectively.

Dominant activating *STAT1* mutations cause a defect in T cell function, which presents as increased susceptibility to fungal and viral infections and autoimmunity.^63^^,^^64^^,^^65^ We thus hypothesized that correction of *STAT1* would reduce STAT1 hyperactivation in stimulated T cells. To correct an activating *STAT1* p.388R mutation, we first designed CRISPR reagents as described (Figures 1 and 3). Of the three available guides surrounding the mutation site (Figure 8A), we identified gRNA number 2 and symmetric repair template as the best combination because gRNA number 2 is mutation specific and does not cut the WT allele (Figures 8A–8C). When correcting patient cells with the optimized platform, we noted up to 40% total HDR, which translates to 80% diseased allele correction because the mutation is heterozygous (Figure 8D).

Activating STAT1 mutations lead to STAT1 hyperphosphorylation in stimulated cells.^66^^,^^67^ Consistently, we saw increased pSTAT1 in patient T cells that were stimulated with IFN-α. The phosphorylation decreased in the patient upon gene correction (Figure 8E). Without stimulation, STAT1 phosphorylation was not observed in uncorrected patient T cells, and consequently, correction did not affect resting pSTAT1 levels in our patient. We conclude that gene correction can reduce excessive STAT1 activation, and that our platform is effective in correcting heterozygous mutations.

## Discussion

In this study, we developed a CRISPR-Cas9-based T cell gene correction platform for monogenic IEIs. We demonstrate up to 80% mutation correction efficiency and functional improvement in the model IEIs. The platform is suitable for correcting diverse SNVs and small indels in multiple genes and is portable for clinical translation. Corrected autologous T cell transplants can further be developed into a salvage therapy for IEI patients with isolated T cell defects.^4^^,^^7^^,^^10^^,^^11^^,^^12^^,^^13^

In this study, we have optimized the T cell editing platform in six endogenous loci (*ADA2*, *AIRE*, *CTCF-1*, *Enh4-1*, *RMRP*, and *RNF2*) in healthy controls and further assessed the functional impact of mutation correction in DADA2, CHH, and STAT1-GOF patients where the peripheral T cells contribute to disease phenotype. While gene editing of peripheral T cells is not expected to offer therapeutic benefits for APECED, which primarily affects the thymic medullary epithelial cells,^68^ the T cells from these patients were used for method/platform development primarily due to practical considerations regarding access to sizable patient cohorts. In DADA2, we observed restored ADA2 protein expression and reduced TNF-α signaling in patient T cells following mutation correction. In CHH, the correction improved the proliferation defect observed in patient T cells. In STAT1-GOF patients, the correction reduced STAT1 hyperphosphorylation into a normal level. Successful SNV editing in six distinct loci along with observed functional impact of SNV correction in three IEIs highlights the versatility of this platform to serve as a universal approach for a wide range of monogenic T cell defects. However, thorough assessment of preclinical efficacy and safety of the strategies presented here are required before further clinical translation. Donor-to-donor variability among healthy controls limits threshold definition in this study. Future work will include larger cohorts to establish normal ranges and support clinical applications in diseases such as CHH and STAT1-GOF. Furthermore, we advise that each model be assessed separately for its potential clinical impact.

Our correction approach requires the presence of T cells that can proliferate, which excludes certain severe combined immunodeficiencies where T cells are absent or do not proliferate. In conditions where T cells exist but have little proliferation, cell-cycle-independent correction methods such as base and prime editing can be better alternatives. If poor proliferation is due to gene defects in cytokine signaling, then adjustments to the presented stimulation protocol can improve correction levels.

CRISPR-Cas9 cutting can lead to off-target cuts. We found no off-targets with GUIDE-seq profiling for the selected gRNAs. Alternative methods such as circularization for *in vitro* reporting of cleavage effects by sequencing, cellular indexing of transcriptomes and epitopes by sequencing, and circularization for high-throughput analysis of nuclease genome-wide effects by sequencing exist and all have their own advantages and limitations.^69^ In addition, CRISPR can induce structural chromosomal changes at the target site.^56^^,^^70^^,^^71^^,^^72^^,^^73^ The structural variants increase with rapid cell proliferation, and optimized culture conditions can decrease the events.^56^ The use of DNA-PKcs inhibition^74^^,^^75^ was recently reported to increase on-target chromosome loss.^76^ Although we did not find persisting genomic aberrations, we cannot exclude the possibility that low-frequency on- and off-target structural variants remain undetected due to technology constraints. The cells with larger abnormalities can also become arrested and disappear below detection limit by the assay time point.^56^^,^^73^ Time point experiments with extended cell culture, along with *in vivo* xenotransplant studies, can complement the safety assessment and help to evaluate the long-term T cell survival and malignant transformation risk.

Our correction strategy introduces 2–4 silent SNVs along with correction of the pathogenic variant. The strategy prevents CRISPR re-cutting after successful HDR repair and improves precise correction levels.^34^^,^^45^ Additionally, it allows accurate and rapid editing quantification by droplet digital PCR. To ensure minimal interference with gene function and regulation, we advise prioritizing SNVs that are part of normal human variation and located in evolutionarily less conserved regions. We also advise that the functional effect of the SNVs be assessed case by case, as certain silent SNVs can disrupt mRNA transcription and protein translation.^77^^,^^78^^,^^79^

In conclusion, we present a non-viral T cell SNV correction platform that has the potential to be scaled up to a translationally relevant platform to correct diverse pathogenic SNVs, small deletions, and insertions in IEIs.

## Materials and methods

The aim of this study was to develop a CRISPR-Cas9-based T cell platform for mutation correction in IEI patients. We used the following Finnish founder diseases as models: DADA2, APECED, and CHH. We obtained PB, cord blood, and skin biopsies from patients or healthy controls. Detailed information on the patients is provided in Table S15. The study was conducted in accordance with the principles of the Declaration of Helsinki and approved by the Helsinki University Central Hospital Ethics Committee and the Regional Committee for Medical and Health Research Ethics South-East Norway. Participants have signed written informed consent forms.

### Isolation, culture, and nucleofection of T cells, CD34^+^ HSPCs, and fibroblasts

PBMCs were isolated from PB using Ficoll gradient centrifugation and cryopreserved. Upon thawing, PBMCs were cultured in ImmunoCult-XF T Cell Expansion Medium supplemented with IL-2, IL-7, IL-15, and CD3/CD28 T cell activator. After 3 nights at 37°C/5% CO_2_, cells were nucleofected or further cultured without the CD3/CD28 activator. CD34^+^ HSPCs were isolated from cord blood using the CD34 MicroBead Kit and cryopreserved. Upon thawing, HSPCs were cultured in StemSpan SFEM II supplemented with GlutaMax, Flt3-L, thrombopoietin, stem cell factor, IL-6, StemRegenin-1, and UM729. After 3 nights at 37°C/5% CO_2_, cells were nucleofected or further cultured. Fibroblasts isolated from skin biopsies were expanded in DMEM with low glucose, pyruvate, and FBS and cryopreserved. Upon thawing, fibroblasts were cultured until confluent, passaged every 3–4 days with TrypLE Express Enzyme, and nucleofected by passage 10.

T cells, CD34^+^ HSPCs, and fibroblasts were nucleofected using a 4-D Nucleofector system and a 96-well unit (Lonza). gRNAs were prepared by annealing CRISPR RNA and *trans*-activating CRISPR RNA (IDT) and mixed with Cas9 nuclease and ssODN (IDT) to form RNPs. T cells (0.5 million or 1 million), HSPCs (0.3 million), and fibroblasts (1 million) were resuspended in 20 μL electroporation buffer and nucleofected using programs EO-115, DZ-100, and CA-137, respectively. Post-nucleofection, T cells, HSPCs and fibroblasts were incubated with their respective recovery media for 15 min, transferred to plates, and cultured until collection after 4–8 days. For details, see the supplemental methods.

### Design and screening of CRISPR-Cas9 reagents

From 7 to 18 gRNAs were designed based on available PAM (NGG) centering the mutation site. ssODNs of 100 bp were designed with ±50-bp homology arms from the mutation site. Synonymous, silent SNVs were added in repair templates for *ADA2* (four SNVs) and *AIRE* (three SNVs) to prevent CRISPR re-cutting and ensure identical editing in donors and patients. As *RMRP* is a non-coding gene, SNVs were used in early experiments, and only mutation correction later in functional assessments. Asymmetric ssODNs with 10- to 40-bp homology arms were tested to enhance HDR. Details about gRNA (Table S2) and ssODN design (Table S3), BG-coupled ssODNs, and Cas9-SNAP protein production can be found in the supplemental methods.

### On-target editing assessment

ddPCR assays were performed to assess HDR and NHEJ editing. Previously described oligos^41^ were used to edit *Enh4-1*, *CTCF1*, and *RNF2*, while new oligos for *ADA2*, *AIRE*, and *RMRP* were designed. ddPCR was performed using the QX200 system (Bio-Rad) and analyzed with QuantaSoft software (Bio-Rad). The oligos are listed in Table S8.

Amplicon sequencing libraries were prepared from gDNA samples using a two-step PCR method.^41^ Unique molecular identifiers were added to the primers to filter out PCR bias.^41^ Libraries were sequenced using the Illumina MiSeq version 2 platform. Data analysis was done using the ampliCan software package.^80^ Amplicon sequencing PCR and oligos are listed in Tables S9–S11.

### Assessment of *in silico* gRNA design tools

We assessed the predictive power of *in silico* gRNA design tools against *in vitro* gRNA screening data using the following tools: Atum, Benchling, CHOPCHOP, CRISPOR, DeepSpCas9, EuPaGDT, and the IDT gRNA design tool. Using 100-bp mutant-specific sequences with 50-bp homology arms as input, we selected the three highest predicted efficiency gRNAs from each tool. These were then compared against the three best *in vitro*-validated gRNAs from patient T cells. Details of the *in silico* tools are listed in the supplemental methods.

### Screening HDR enhancers and cell-cycle inhibitors in healthy control T cells

A total of 33 HDR enhancers and 10 cell-cycle inhibitors (Tables S12 and S13) were screened in healthy donor (HD) T cells at 3 concentrations against RNP-edited cells (DMSO). For HDR enhancers, 0.5 million T cells per sample were nucleofected and incubated, with the compounds in T cell recovery medium for 24 h, then split 1:1 in recovery medium without compounds 24 and 72 h after nucleofection. The toxicity of HDR enhancers was assessed using the CellTiter-Glo assay (Promega) according to the manufacturer’s instructions (see details in the supplemental methods). For cell-cycle inhibitors, cells were either pre-treated with the compounds for 24 h before or 24 h after nucleofection. In both cases, 0.5 million cells per sample were nucleofected and split 1:1 in recovery medium without compounds 24 and 72 h after nucleofection. Samples for both screens were collected 96 h after nucleofection for gDNA extraction and ddPCR.

### Off-target editing assessment

The previously published GUIDE-seq method^53^ was used to assess off-target editing (see details in the supplemental methods). In brief, 1 million T cells per sample were nucleofected on day 5 with RNPs (5 μmol/L/sample gRNA, 3.05 μmol/L/sample Cas9 nuclease, 1.5 μmol/L/sample dsODN). Samples were collected for library preparation, sequencing, and ddPCR 4 days later. Data analysis was performed following the GUIDE-seq analysis pipeline from Zhu et al.,^81^ but adjusted for allowing bulges between single-guide RNA (sgRNA) and off-target sites with editing distance of 4 with the use of CHOPOFF.^82^ Final off-targets were normalized against control data (transfected with dsODN only). We used custom scripts available at https://git.app.uib.no/valenlab/t_cell_editing_pipeline/.

### PacBio sequencing of CRISPR-edited healthy control T cells

Healthy control T cells were edited as described above and treated with 0.5 μM KU0060648 or DMSO. Six days post-editing, DNA was extracted from 5 million cells per sample using Qiagen kits. DNA quality was assessed using NanoDrop, Qubit, and agarose gel electrophoresis. Libraries for PacBio HiFi sequencing were prepared using the Revio HiFi Prep Kit and Sequencing Chemistry version 2.0. Sequencing data were demultiplexed with SMRT Link, and circular consensus sequence reads were generated and further demultiplexed using barcoded primers, with HiFi reads indexed by barcode IDs. The HiFi sequencing reads were aligned with pbmm2 version 1.13.0. Structural variants were called with pbsv version 2.9.0 and small variants with deepVariant version 1.6.0. All possible mismatches, deletions, and insertions were extracted from aligned reads using custom scripts (https://git.app.uib.no/valenlab/t_cell_editing_pipeline/-/tree/main/katariina_pacbio). We normalized data using two control samples and focused on sites that were potential sgRNA off-target within distance of 4, allowing for bulges.

### Immunophenotyping by flow cytometry

PBMC samples from days 1, 4, and 8 of the platform were assessed using flow cytometry. Cells (0.5 million per sample) were washed with flow cytometry buffer, blocked with 10% human serum, and stained with an antibody cocktail (Table S4) to identify CD4 T cells, CD8 T cells, B cells, natural killer (NK) cells, monocytes, and dendritic cells. After washing, cells were resuspended in 250 μL flow cytometry buffer and stored at 4°C. Flow cytometry was done on LSRII and data analysis was done using FlowJo. For details, see the supplemental methods.

### T cell proliferation assay in CHH patients

T cells from CHH patients from day 20 of the platform were collected, washed with PBS, and resuspended at 2 million cells/mL. Cells were stained with 1 μM CFSE and incubated in the dark at 37°C for 5 min. Cold human serum was added to quench the reaction. Cells were then washed and resuspended in Immunocult medium supplemented with 250 U/mL IL-2 at 0.2 million cells per well in a 96-well U-bottom plate. After 4 days, cells were stained with an antibody cocktail (Table S6) and analyzed by flow cytometry as described previously. For details, see the supplemental methods.

### Assessment of STAT1 phosphorylation in STAT1-GOF patients

T cells from corrected and uncorrected STAT1-GOF patients were collected 4 days after nucleofection, washed with PBS, and resuspended at 1 million cells/sample. Cells were stained in the dark at 4°C for 30 min with Live/Dead dye and FcR Blocking Reagent, after which cells were stimulated with 250 μL of the 2 × 10^3^ U/mL of the IFN-α in Immunocult medium and the cocktail of cell surface antibodies (Table S7). The unstimulated controls received only 250 μL medium and the same antibody cocktail. Cells were incubated at 37°C for 30 min in the dark, with shaking every 5 min. Immediately after, 2 mL freshly prepared 1:5 Phosflow Lyse/Fix Buffer was added to the samples, which were shortly vortexed before incubating at 37°C for 10 min, with shaking every 3 min. After incubation, samples were washed and centrifuged, and 500 μL cold Phosflow PermBuffer III was added, followed by incubation on ice for 30 min in the dark. Cells were washed with flow buffer and stained with 1:10 dilution of pSTAT1 antibody, followed by 30 min incubation at room temperature in the dark. Afterward, cells were washed two times with flow buffer, resuspended in flow buffer, and stored in a refrigerator overnight for flow cytometry analysis the day after. For details, see the supplemental methods.

### DNRT scRNA-seq and RT-qPCR of control and DADA2 patient T cells

A previously published Smart-Seq2-based direct nuclear tagmentation and RNA-seq (DNRT) protocol was used.^83^ For details, see the supplemental methods. In brief, on day 8, nucleofected HD and DADA2 patient T cells were collected, washed, and stained with Live/Dead dye and Fc blocking reagent. After washing, cells were stained with antibody cocktail (Table S5), washed and resuspended in flow buffer. Live CD4+ and CD8+ T cells were sorted into 384-well plates with lysis buffer. After sorting, plates were centrifuged, snap-frozen, and stored at −80°C. Using the Smart-Seq2 protocol,^83^ cells were thawed, reverse transcribed, and cDNA pre-amplified, with cleanup using SPRI beads and concentration measured with the Qubit DNA HS kit (Table S14). Tagmentation of diluted cDNA was followed by SDS reaction stop, barcoding, and PCR. Libraries were cleaned with SPRI beads and sequenced on a Novaseq 6000.

For data analysis, the reads were trimmed with Cutadapt^84^ and aligned to hg38 with STAR.^85^ Picard^86^ removed duplicates, and HTSeq^87^ summarized counts. Cells with <20,000 reads, <500 features, or low ACTB expression were filtered out. Seurat^88^ version 5.0.1 log-normalized data identified 2,000 variable features and scaled data per condition. FindMarkers in Seurat identified markers between conditions, and fgsea^89^ performed gene set enrichment analysis. Fusion gene detection was performed with STAR-Fusion. Loss of heterozygosity calculations were performed as described in the supplemental methods. For quantitative analysis of different alleles in single cells, 1 μL diluted cDNA was amplified with specific probes for WT and edited alleles (see details in the supplemental methods). RT-qPCR analysis used Bio-Rad software with a 200 relative fluorescence units as a threshold for determining which allele was being expressed.

### MS

For details, see the supplemental methods. In brief, T cells from three DADA2 patients and HDs were cultured with 1 million cells per sample and nucleofected on day 5. Mock-nucleofected cells were treated with DMSO, and edited cells with 0.5 μM KU0060648, 0.6 μM IDT Alt-R enhancer V2, or DMSO for 24 h. Cells were collected on day 12, washed, pelleted, and snap-frozen on liquid nitrogen.

For MS, trypsin/LysC digested samples were diluted 1:60 in 0.1% formic acid in water, and 20 μL was loaded into an Evotip. Samples were analyzed using the Evosep One system with the Bruker timsTOF Pro mass spectrometer. Peptide separation used an 8 cm × 150 μm column with a 21-min gradient. Data were processed with DIA-NN version 1.8.1^90^^,^^91^ using the UniProt human proteome spectral library, with fixed and variable modifications. Pre-processing involved log2 transformation, median-normalization, and QRILC imputation (https://cran.r-project.org/web/packages/imputeLCMD/imputeLCMD.pdf). Statistical analysis used Student’s t test^92^ and the Benjamini-Hochberg method^93^ for *p* value adjustment. The volcano plots were generated using bioinfokit.

## Data availability

The data can be found in Tables S1–S14, Figures S1–S18, and Tables S15, S16, S17, S18, S19, S20, and S21. Raw GUIDE-seq, scRNA-seq, MS and PacBio whole-genome sequencing data will be deposited in a secure repository after publication.

## Acknowledgments

We thank all patients and families for their participation in the study. We thank Karolinska Institute Protein Science Facility for manufacturing the Cas9 protein and Riitta Lehtinen for her expert technical assistance. The 10.13039/501100005416Research Council of Norway, Health South-East Region, the Swedish Childhood Cancer Society (Barncancerfonden), and the 10.13039/100008730Norwegian Cancer Society supported this work. This work was partially supported by the 10.13039/501100005416Research Council of Norway through its Centers of Excellence scheme (project number 332727).

## Author contributions

K.M. performed most of the experiments and wrote the manuscript. S. Kolbeinsdottir and M.E. performed the scRNA-seq experiments. Z.L. designed the CRISPR reagents and performed the GUIDE-seq optimization. K.L., E.T., and E.V. performed the bioinformatics and data analysis. A.K. performed the GUIDE-seq and PacBio experiments. S. Keskitalo, A.T., and M.V. performed the MS experiments and data analysis. G.R. designed the CRISPR reagents and the amplicon sequencing panel and performed the experiments. F.H.H. performed the gRNA screening and BG-coupled ssODN experiments. B.O.L. performed the CRISPR optimization experiments in T cells. S.S.J. performed gRNA screening and the STAT1-GOF functional assessment. T.J.G. performed flow cytometry experiments. C.W.E. and P.K. performed gRNA screening in CD34^+^ HSPCs. N.F. and M.S. performed library preparation for amplicon sequencing. T.M.M. obtained cord blood for HSPC isolation. J.S. and J.O. supervised the experiments. V.G., E.L., C.S.-J., T.H., K.H.B.M., H.C.E., and T.M. provided clinical care for the patients and obtained samples. S.D.-K. designed the scRNA-seq, flow cytometry and cell sorting experiments; performed and supervised the research; and wrote the manuscript. E.H. supervised the study and wrote the manuscript. All authors read and approved the manuscript.

## Declaration of interests

Authors declare no competing interests.

## Supplemental information

Document S1. Figures S1–S18, Tables S1–S14, and supplemental methods

Table S15. Patient table for IEI patients used in the study

Table S16. CHOPOFF off-target predictions for selected gRNAs

Table S17. Barcodes used for scRNA-seq

Table S18. scRNA-seq fusion detection

Table S19. Mass spectrometry comparing all samples to unedited healthy controls

Table S20. Mass spectrometry comparing all samples to unedited DADA2 patients

Table S21. Mass spectrometry hits in DADA2 patient

Document S2. Article plus supplemental information
