# Supplementary material for: Precision T cell correction platform for inborn errors of immunity
Source: Mol Ther. 2025 Aug 12;33(11):5624–43. doi: 10.1016/j.ymthe.2025.08.018 (PMC12628183; doi:10.1016/j.ymthe.2025.08.018)
Supplement: Document S1. Figures S1–S18, Tables S1–S14, and supplemental methods [file mmc1.pdf]

YMTHE, Volume *33*

Supplemental Information

Precision T cell correction platform

for inborn errors of immunity

Katariina Mamia, Solrun Kolbeinsdottir, Kornel Labun, Zhuokun Li, Anna Komisarczuk, Salla Keskitalo, Ganna Reint, Frida Loe Haugen, Britt Olaug Lindestad, Siv Skundberg Jensen, Thea Johanne Gjerdingen, Antti Tuhkala, Carolina Wieczorek Ervik, Pavel Kopcil, Nail Fatkhutdinov, Karen Helene Bronken Martinsen, Hans Christian Erichsen, Monika Szymanska, Eero Tölö, Virpi Glumoff, Janna Saarela, Trond Melbye Michelsen, Camilla Schalin-Jäntti, Johanna Olweus, Eira Leinonen, Markku Varjosalo, Eivind Valen, Timo Hautala, Martin Enge, Timi Martelius, Shiva Dahal-Koirala, and Emma Haapaniemi

SUPPLEMENTAL FIGURES

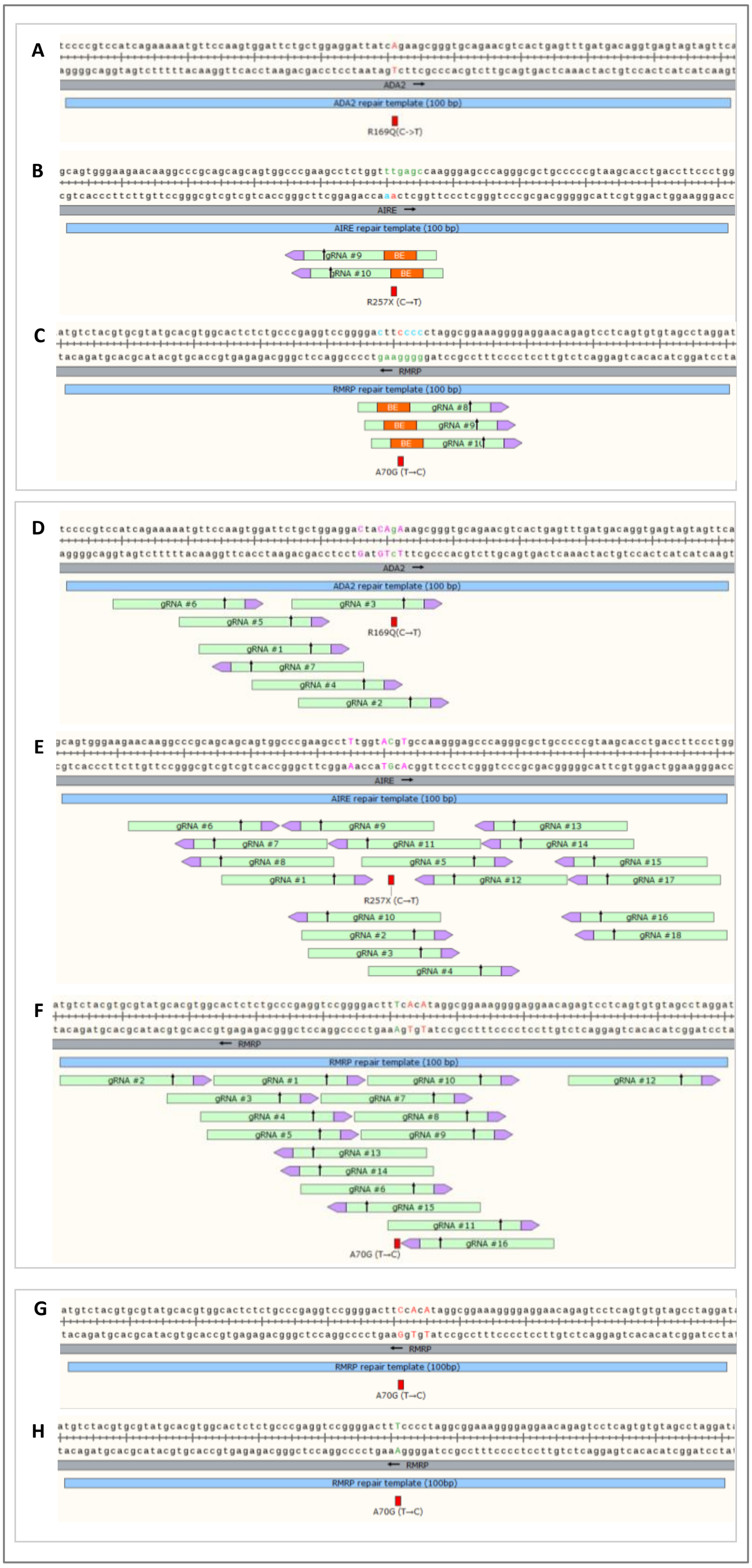

Figure S1. Repair template and gRNA design for *ADA2*, *AIRE* and *RMRP*

(A) Schematic representation of *ADA2* mutant site, marked in red, with no possible base editing gRNAs. (B) Schematic representation of *AIRE* mutant site, where the edited pathogenic mutation nucleotide is marked with red, showing possible A→G base editing gRNAs. The nucleotide positions which fall within the editing window span of BE guides are shown in green and the bystander edits are shown in blue. (C) Schematic representation of *RMRP* mutant site, where the edited pathogenic mutation nucleotide is marked with red, showing possible A→G base editing gRNAs. The nucleotide positions which fall within the editing window span of BE guides are shown in green and the bystander edits are shown in blue. (D) Schematic representation ADA2 gRNA design

and repair strategy. Correction of pathogenic mutation (red) is marked with green and silent SNVs as pink uppercase letters. (E) Schematic representation AIRE gRNA design and repair strategy, as explained in (d). (F) Schematic representation RMRP gRNA design and repair strategy, as explained in (d). As *RMRP* is noncoding, non-silent SNVs (red) were added in the repair strategy for early experiments. (G) Schematic representation of RMRP SVP strategy for editing wild-type cells, where non-silent SNVs (red) were added in the repair strategy for early experiments. (H) Schematic representation of *RMRP* repair strategy for mutation (red) correction (green). Abbreviations: gRNA (guide-RNA), BE (base editing), SNV (singe nucleotide variant), gRNA (guide-RNA).

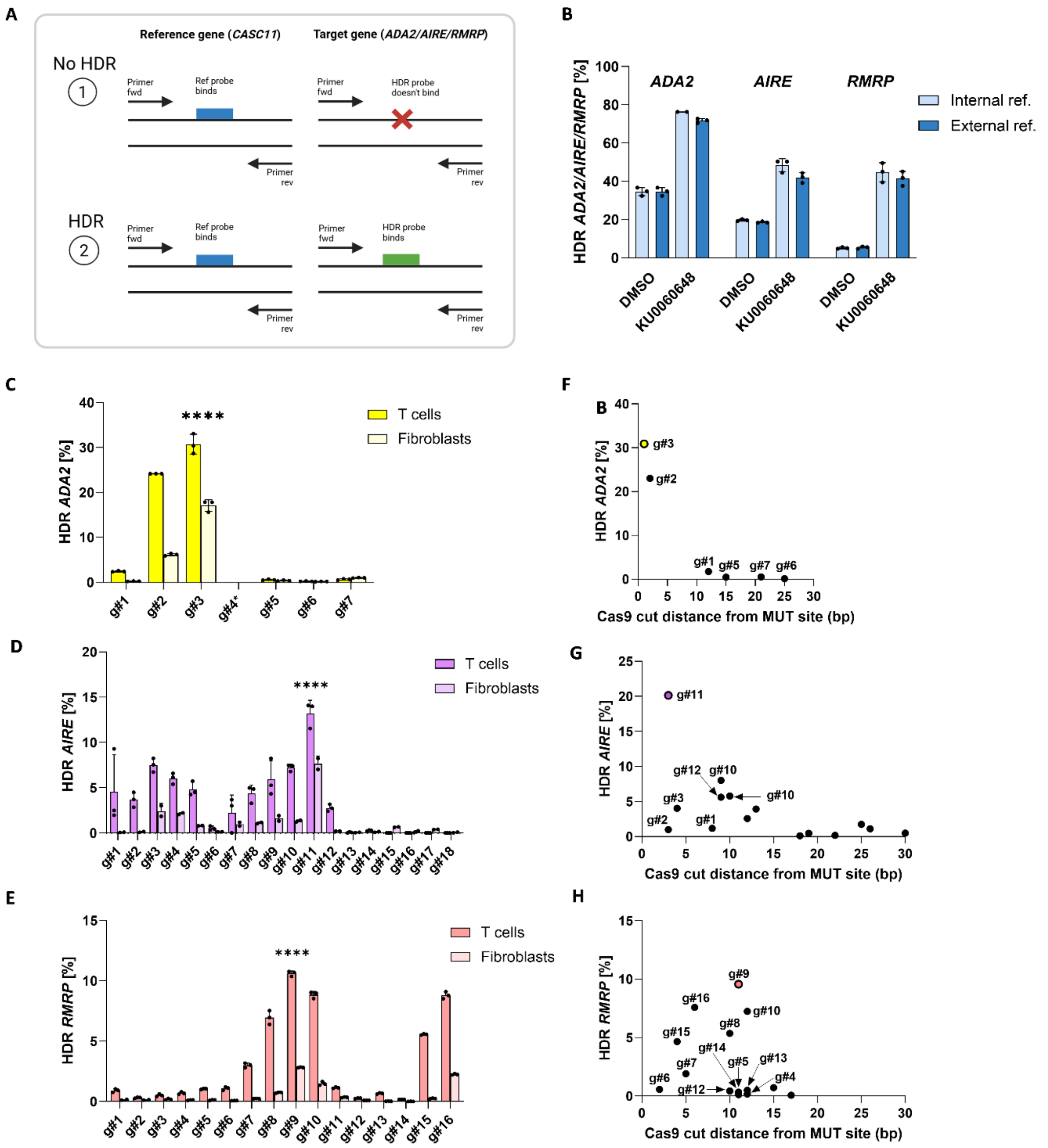

I

	Predicted #1	Predicted #2	Predicted #3
**Atum**	Not tested	g1	g5
**Benchling**	g6	Not tested	g2
**CHOPCHOP**	g6	g5	Not tested
**CRISPROR**	Not tested	Not tested	g5
**DeepSpCas9**	Not tested	g2	g5
**EuPaGDT**	g5	g2	Not tested
**IDT design tool**	Not tested	g5	g1

J

	Predicted #1	Predicted #2	Predicted #3
**Atum**	g15	g16	g13
**Benchling**	g11	g4	g9
**CHOPCHOP**	g11	g4	g2
**CRISPROR**	g15	g13	g16
**DeepSpCas9**	g4	g11	g5
**EuPaGDT**	g16	g17	g15
**IDT design tool**	g10	g4	g9

K

	Predicted #1	Predicted #2	Predicted #3
**Atum**	Not tested	Not tested	Not tested
**Benchling**	g7	g3	g11
**CHOPCHOP**	g7	g3	g11
**CRISPROR**	g2	Not tested	g16
**DeepSpCas9**	g11	g10	g1
**EuPaGDT**	g5	g10	g4
**IDT design tool**	g12	g3	g2

Figure S2. ddPCR assay design, gRNA screening results and *in silico* gRNA predictions for *ADA2*, *AIRE* and *RMRP*

(A) Schematic representation of an alternative ddPCR assay design for HDR detection, which uses an external reference probe binding to *CASC11* locus, while using an internal HDR probe that binds to the edited *ADA2*, *AIRE* or *RMRP* locus, respectively. Original ddPCR assay design schematic with an internal reference probe is shown in Fig. 1C. (B) Comparison of HDR frequencies reported by a ddPCR assay using an internal reference probe (light blue) and an external one (dark blue) in *ADA2*, *AIRE* and *RMRP* -edited HD T cells treated with 0.5 μM KU0060648 or DMSO. (C) ADA2 gRNA screening in DADA2 patient T cells and fibroblasts, HDR assessed by amplicon sequencing (n=3 technical replicates). (D) AIRE gRNA screening in APECED patient T cells and fibroblasts, HDR assessed by amplicon sequencing (n=3 technical replicates in T cells, n=2 in fibroblasts). (E) RMRP gRNA screening in CHH patient T cells and fibroblasts, HDR assessed by amplicon sequencing (n=3 technical replicates). Samples from (c-e) are the same samples assessed by ddPCR in Fig. 1E-G. Cas9 cut distance from mutation site plotted against HDR frequency for tested gRNAs in DADA2 (F), APECED (G) and CHH (H) patient T cells, where reported HDR frequencies are the ddPCR measurements reported in Fig. 1E-G. The best gRNA is shown as the coloured dot. (I) Comparison of three best ADA2 gRNAs identified by *in silico* gRNA design tools to *in vitro* validated gRNA screening results from DADA2 patient T cells, where accurate predictions are shown in green, incorrect predictions in red and gRNAs that were designed by the tools but not assessed *in vitro* in white. (J) Comparison of three best AIRE gRNAs identified by *in silico* gRNA design tools to *in vitro* validated gRNA screening results from APECED patient T cells, where accurate predictions are shown in green, incorrect predictions in red. (K) Comparison of three best RMRP gRNAs identified by *in silico* gRNA design tools to *in vitro* validated gRNA screening results from CHH patient T cells, where accurate predictions are shown in green, incorrect predictions in red and gRNAs that were designed by the tools but not assessed *in vitro* in white. One independent experiment was performed for all sets of data. Statistical

significance of highest HDR for a given gRNA was assessed by one-way ANOVA with Fisher’s LSD test, where ****p<0.0001. Bar denotes mean value, error bars represent ± SD. Abbreviations: ddPCR (droplet digital PCR), gRNA (guide-RNA), DADA2 (Deficiency of adenosine deaminase 2), HDR (homology-directed repair), APECED (Autoimmune polyendocrinopathy-candidiasis-ectodermal dystrophy), CHH (Cartilage hair hypoplasia).

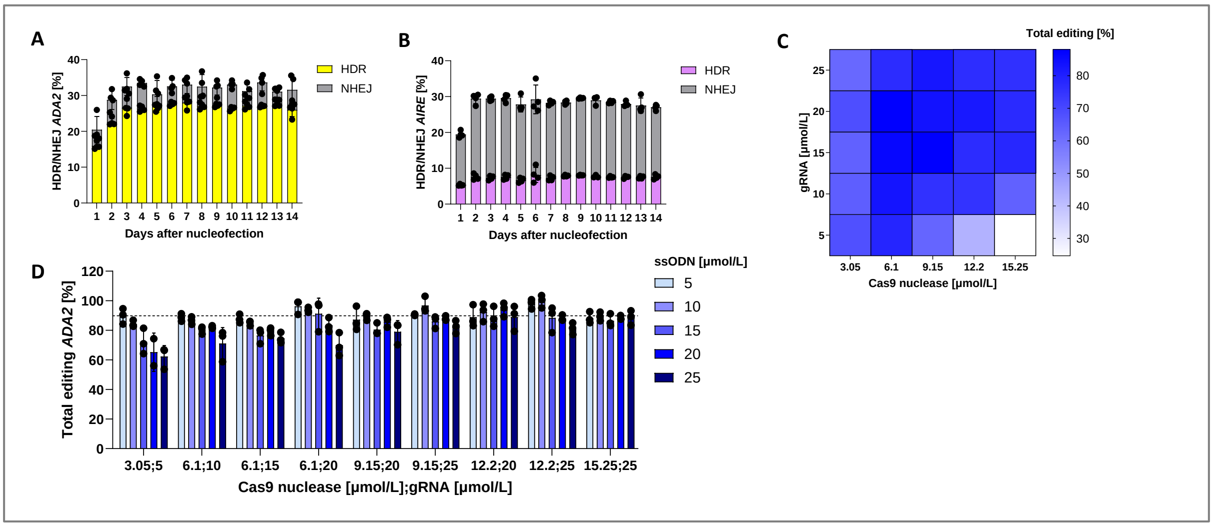

Figure S3. Optimization of CRISPR reagents and nucleofection in healthy control T cells

(A) *ADA2* HDR and NHEJ editing in HD T cells 1-14 days after nucleofection, measured by ddPCR (n=3 technical replicates). (B) *AIRE* HDR and NHEJ editing in HD T cells 1-14 days after nucleofection, measured by ddPCR (n=3 technical replicates). (C) *ADA2* total editing (reported as the sum of HDR and NHEJ) in HD T cells nucleofected with Cas9 nuclease at 3.05-15.25-, gRNA at 5-25- and ssODN at 5 µmol/L per nucleofected sample, measured by ddPCR (n=3 technical replicates). (D) *ADA2* total editing (reported as the sum of HDR and NHEJ) in HD T cells with selected combinations of RNPs with ssODN at 5-25 µmol/L per nucleofected sample, measured by ddPCR (n=3 technical replicates). Dashed line indicates mean value of Cas9 nuclease at 3.05 µmol/L-, gRNA at 5 µmol/L- and ssODN at 5 µmol/L per nucleofected sample. One independent experiment was performed for all sets of data except for (c)-(d) where one out of three representative experiments is shown. Bar denotes mean value,

error bars represent ± SD. Abbreviations: HDR (homology-directed repair), NHEJ (non-homologous end joining), HD (healthy donor), ddPCR (Droplet Digital PCR), gRNA (guide-RNA), ssODN (single-stranded oligodinucleotide), RNP (ribonucleoprotein).

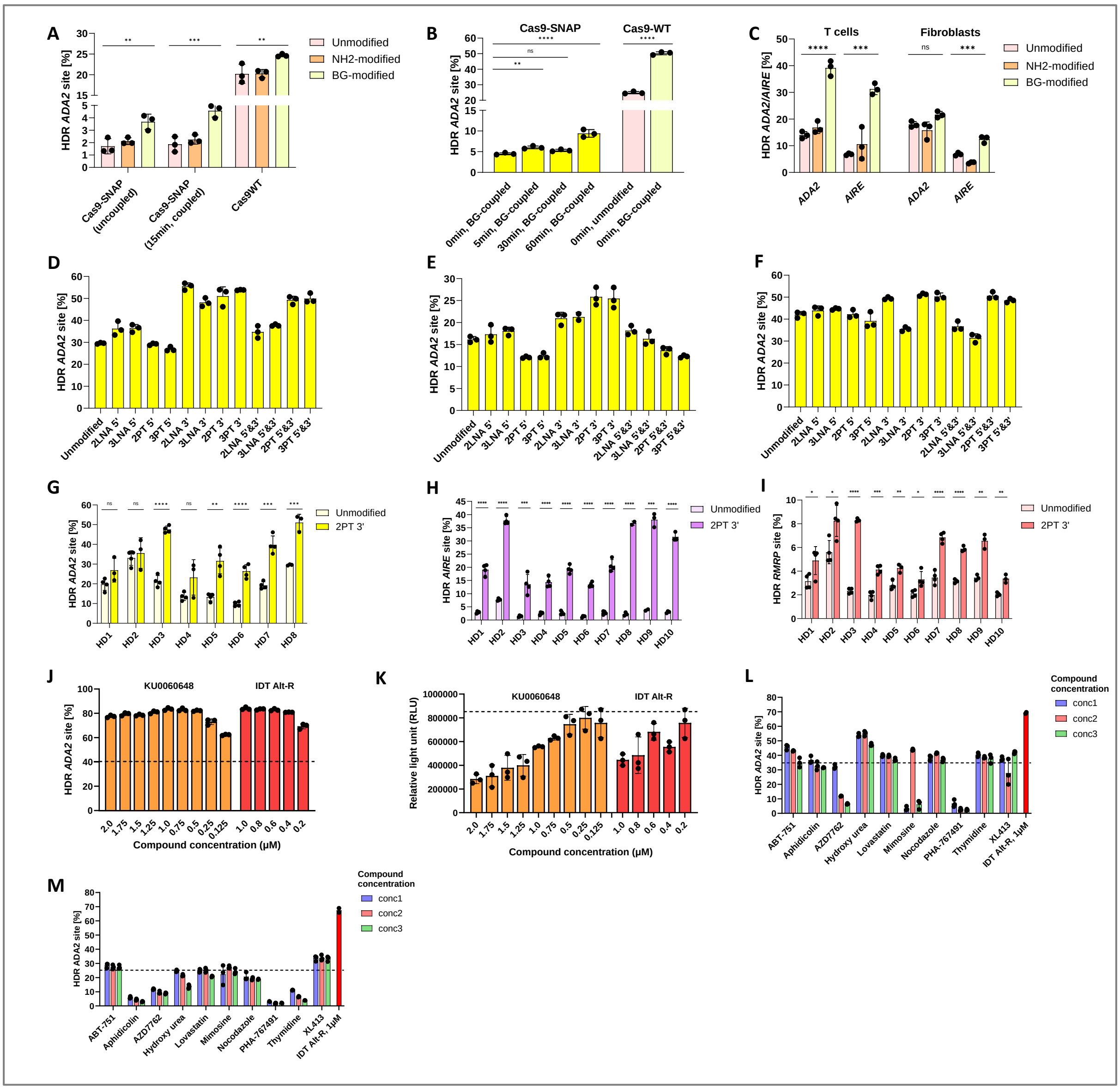

Figure S4. Assessing HDR improvement strategies in healthy control primary cells

(A) *ADA2* HDR editing in HD fibroblasts with unmodified, NH2- or BG-modified ssODNs with Cas9-SNAP (uncoupled or coupled with BG) or Cas9WT nuclease (uncoupled), measured by ddPCR (n=3 technical replicates). (B) *ADA2* HDR editing in HD T cells with unmodified (pink bar) or BG-modified (bright and pale-yellow bars) ssODNs with Cas9-SNAP or Cas9WT

nuclease, measured by ddPCR (n=3 technical replicates). (C) *ADA2* and *AIRE* HDR editing in HD T cells and fibroblasts with unmodified, NH2- or BG-modified ssODNs with Cas9WT nuclease, measured by ddPCR (n=3 technical replicates). *ADA2* HDR editing with LNA- and PT-modified ssODNs in HD (D) T cells (E), fibroblasts and (F) CD34+ HSPCs, measured by ddPCR (n=3 technical replicates). HDR editing in 8-10 healthy T cell donors with position-optimized ssODNs with unmodified or 2PT 3' modified ssODNs for (G) *ADA2*, (H) *AIRE* and (I) *RMRP*, measured by ddPCR (n=3-4 technical replicates depending on the donor). Effect of HDR enhancing compounds at selected concentrations (0.125-2 μM KU0060648, 0.2-1 μM IDT Alt-R enhancer V2) in HD T cells on (J) *ADA2* HDR editing, measured by ddPCR (n=3 technical replicates), where dashed line indicates the mean of RNP baseline (DMSO), and (K) cell viability 96h after nucleofection, measured by CellTiter-Glo (n=3 technical replicates). *ADA2* HDR editing in HD T cells with cell cycle inhibitors at three concentrations in increasing order (conc1-conc3) applied (L) 24h pre- and (M) 24h post nucleofection, measured by ddPCR (n=3 technical replicates). Dashed line indicates the mean value of RNP baseline (DMSO). A single experiment was performed for all sets of data except for (j) and (k) where three independent experiments were performed, and the representative experiment is shown. Statistical significance was assessed by one-way ANOVA with Fisher's LSD test, where ****p<0.0001, ***p<0.0002, **p<0.001 and *p<0.01. Bar denotes mean value, error bars represent ± SD. Abbreviations: HDR (homology-directed repair), HD (healthy donor), BG (benzylguanine), ssODN (single-stranded oligodinucleotide), ddPCR (Droplet Digital PCR), LNA (locked nucleic acid), PT (phosphorothioate), LNA (locked nucleic acid), HSPC (hematopoietic stem and progenitor cell), RNP (ribonucleoprotein).

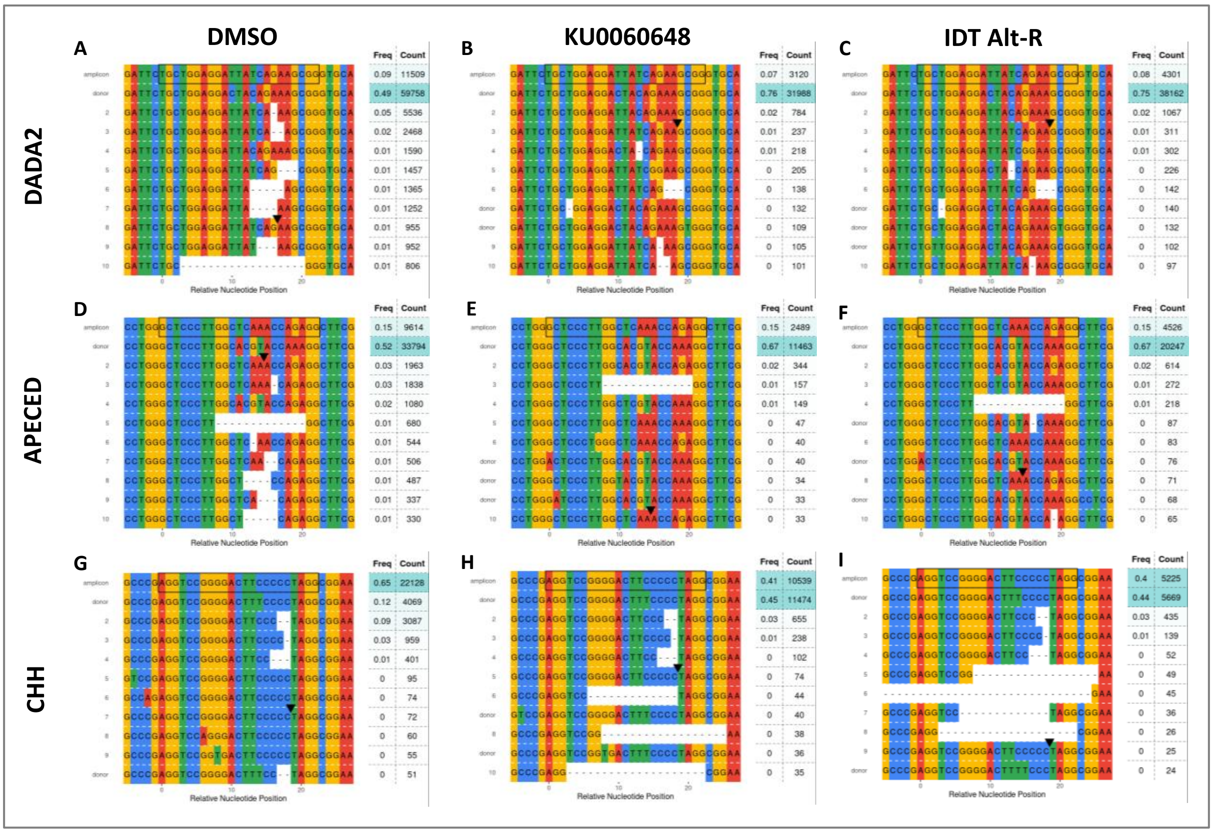

Figure S5. Amplicon sequencing variant plots in corrected DADA2, APECED and CHH patient T cells

(A-C) Amplicon sequencing variant plots in corrected DADA2 patient (DADA2 1) treated with HDR enhancing compounds (0.5 µM KU0060648 and 0.6 µM IDT Alt-R enhancer V2) or DMSO. (D-F) Amplicon sequencing variant plots in corrected APECED patient (APECED 1) treated with HDR enhancing compounds (0.5 µM KU0060648 and 0.6 µM IDT Alt-R enhancer V2) or DMSO. (G-I) Amplicon sequencing variant plots in corrected CHH patient (CHH 2) treated with HDR enhancing compounds (0.5 µM KU0060648 and 0.6 µM IDT Alt-R enhancer V2) or DMSO. For all patients, samples were collected four days after nucleofection. Edits are characterized on the left side of the plot, where "amplicon" is the unedited wild-type sequence, "donor" below the "amplicon" the perfect HDR and imperfect HDR or indels in the rows below "amplicon". Arrow indicates an insertion. Frequencies and counts are reported on the right side of the plots. HDR was assessed by amplicon sequencing, where one representative

measurement is shown (n=3 technical replicates). One independent experiment was performed for all sets of data. The patient number corresponds to patient information in Supplemental Table S15. Abbreviations: DADA2 (Deficiency of adenosine deaminase 2), APECED (Autoimmune polyendocrinopathy-candidiasis-ectodermal dystrophy), CHH (Cartilage Hair Hypoplasia).

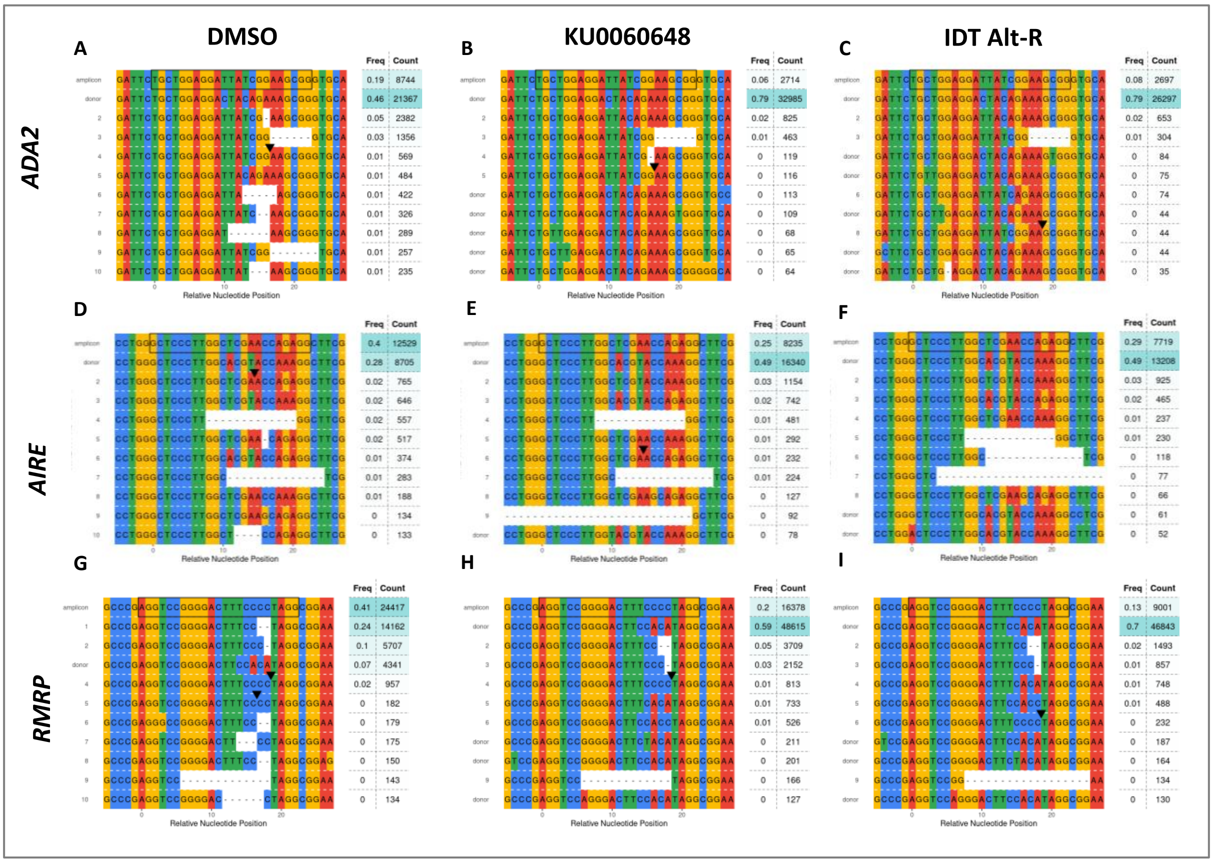

Figure S6. Amplicon sequencing variant plots in *ADA2*, *AIRE* and *RMRP* -edited healthy control T cells

(A-C) Amplicon sequencing variant plots in *ADA2-edited* HD treated with HDR enhancing compounds (0.5 µM KU0060648 and 0.6 µM IDT Alt-R enhancer V2) or DMSO. (D-F) Amplicon sequencing variant plots in *AIRE-edited* HD treated with HDR enhancing compounds (0.5 µM KU0060648 and 0.6 µM IDT Alt-R enhancer V2) or DMSO. (G-I) Amplicon sequencing variant plots in *RMRP*-edited HD treated with HDR enhancing compounds (0.5 µM KU0060648 and 0.6 µM IDT Alt-R enhancer V2) or DMSO. For all loci,

samples were collected four days after nucleofection. Edits are characterized on the left side of the plot, where "amplicon" is the unedited wild-type sequence, "donor" below the "amplicon" the perfect HDR and imperfect HDR or indels as the remaining rows below "amplicon". Arrow indicates an insertion. Frequencies and counts are reported on the right side of the plots. HDR was assessed by amplicon sequencing, where one representative measurement is shown (n=3 technical replicates). One independent experiment was performed for all sets of data. Abbreviations: HD (healthy donor).

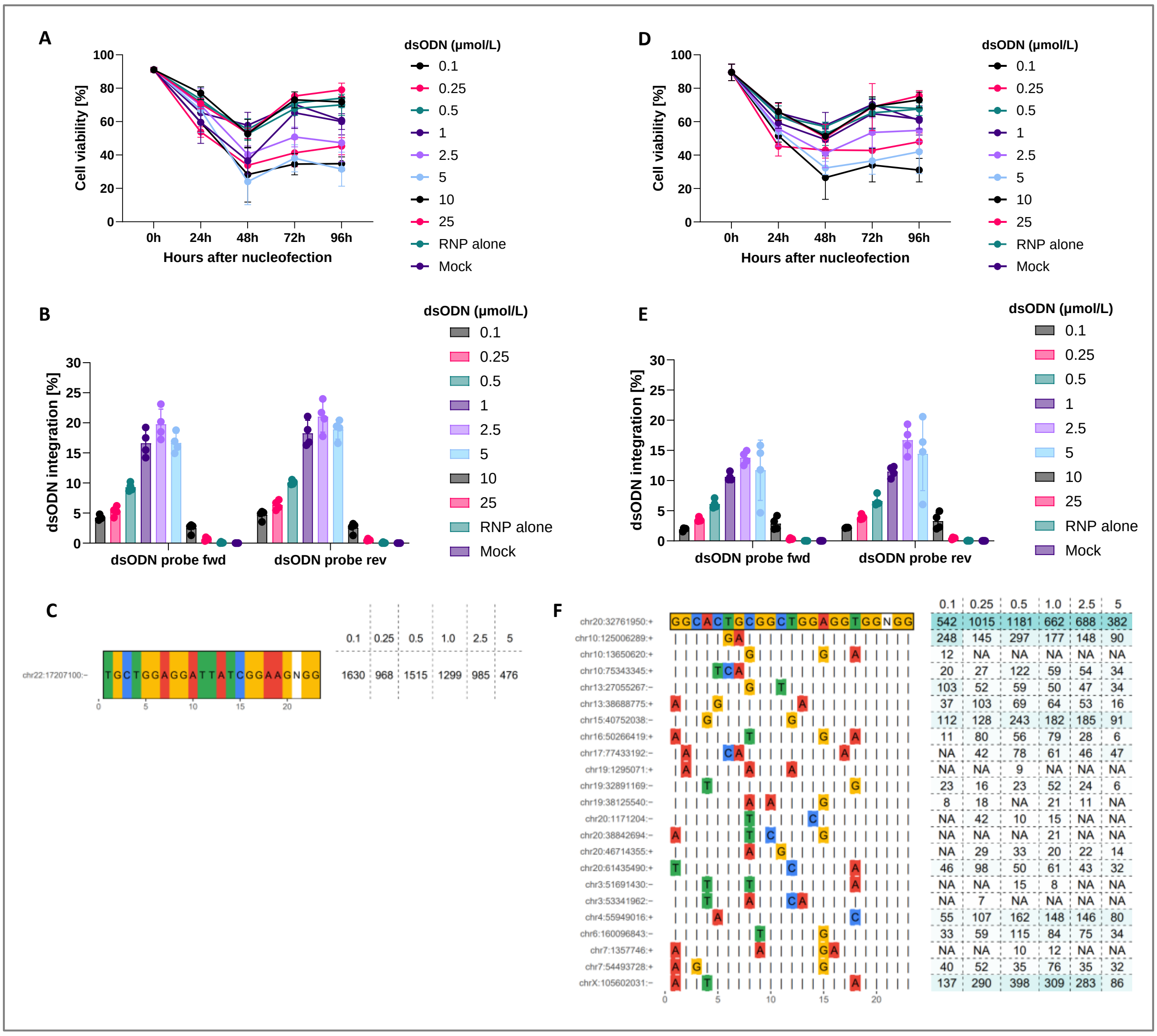

Figure S7. GUIDE-seq optimization in healthy control T cells

(A) HD T cell viability 24-96h after nucleofection with 0-25 µmol/L dsODN/nucleofected sample for *ADA2* locus (n=4 technical replicates). (B) dsODN integration in HD T cells with

0-25 μmol/L dsODN/nucleofected sample for *ADA2* locus, assessed by ddPCR (n=4 technical replicates). (C) GUIDE-seq mismatch plot for ADA2 gRNA #3 in HD T cells with dsODN at 0.1-5 μmol/L dsODN/nucleofected sample. On-target sequence is reported at the top of the table with sequencing reads for each dsODN concentration at the right. (D) HD T cell viability 24-96h after nucleofection with 0-25 μmol/L dsODN/nucleofected sample for *HEK-site4* locus (n=4 technical replicates). (E) dsODN integration in HD T cells with 0-25 μmol/L dsODN/nucleofected sample for *HEK-site4* locus, assessed by ddPCR (n=4 technical replicates). (F) GUIDE-seq mismatch plot for HEK-site4 gRNA, targeting the endogenous human embryonic kidney HEK site 4, in HD T cells with dsODN at 0.1-5 μmol/L dsODN/nucleofected sample. The most abundant off-targets are listed under the target site with their corresponding locations in the genome (left) and sequencing read counts (right). Coloured bases of off-targets indicate mismatches with the on-target site. One independent experiment was performed for all sets of data. Bar denotes mean value, error bars represent ± SD. Abbreviations: GUIDE-seq (Genome-wide, Unbiased Identification of DSBs Enabled by Sequencing), HD (healthy donor), dsODN (double-stranded oligodeoxynucleotide), ddPCR (Droplet Digital PCR), gRNA (guide-RNA), HDR (homology-directed repair), RNP (ribonucleoprotein).

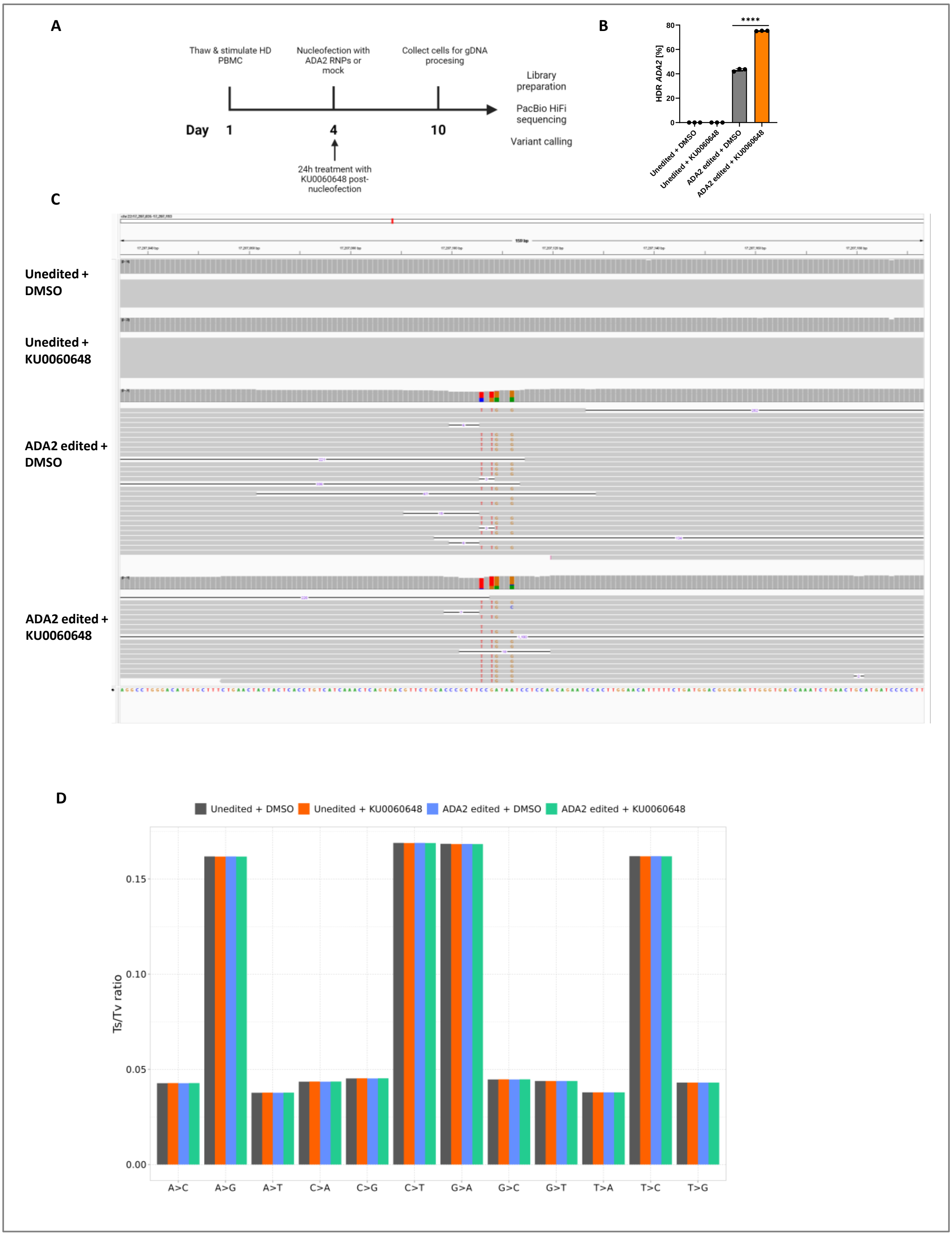

Figure S8. Whole genome sequencing of unedited and *ADA2*-edited healthy control T cells

(A) Outline of the WGS experiment, briefly discussed here: HD T cells were thawed and stimulated with IL-2 (120 U/mL), IL-7 (3 ng/µL), IL-15 (3 ng/µL) and soluble CD3/CD28 (15

μL/mL) on day 1 and nucleofected on day 4 with ADA2 RNPs or mock. Cells were cultured in IL-2 (250 U/mL) and 0.5 μM KU0060648 or DMSO for 24h after nucleofection and collected on day 10 of the platform. gDNA from samples was processed for ddPCR and PacBio sample preparation, followed by PacBio HiFi sequencing and analysis. (B) ADA2 HDR editing levels, assessed by ddPCR (measurements performed in triplicates. (C) IGV view of HiFi PacBio reads on the on-target ADA2 site. HDR reads contain four SNVs at the same time: C>T, G>T, A>G and A>G. No editing is present in the unedited samples. (D) Transition transversion ratio plot showing no difference between edited and unedited samples, showing no global CRISPR toxicity. One independent experiment was performed for all sets of data. Bar denotes mean value, error bars represent ± SD. Statistical significance was assessed by one-way ANOVA with Fisher's LSD test, where ****p<0.0001. Abbreviations: WGS (whole genome sequencing), HD (healthy donor), IL (interleukin), RNP (ribonucleoprotein), DMSO (dimethyl sulfoxide), gDNA (genomic DNA), ddPCR (Droplet Digital PCR), IGV (Integrative Genomics Viewer), HDR (homology-directed repair).

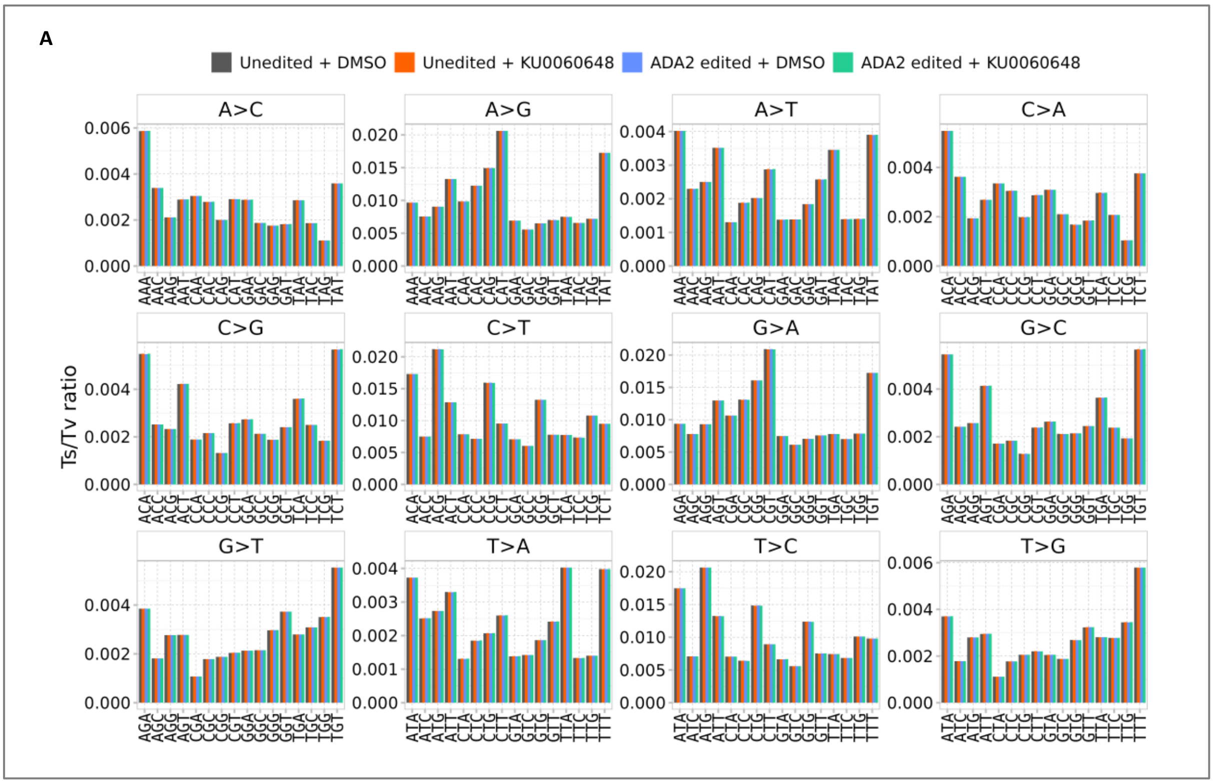

Figure S9. Whole genome sequencing of unedited and ADA2-edited healthy control T cells

(A) Mutational signature by codon shows no differences between edited and unedited samples. One independent experiment was performed for all sets of data. Abbreviations: WGS (whole genome sequencing), HD (healthy donor).

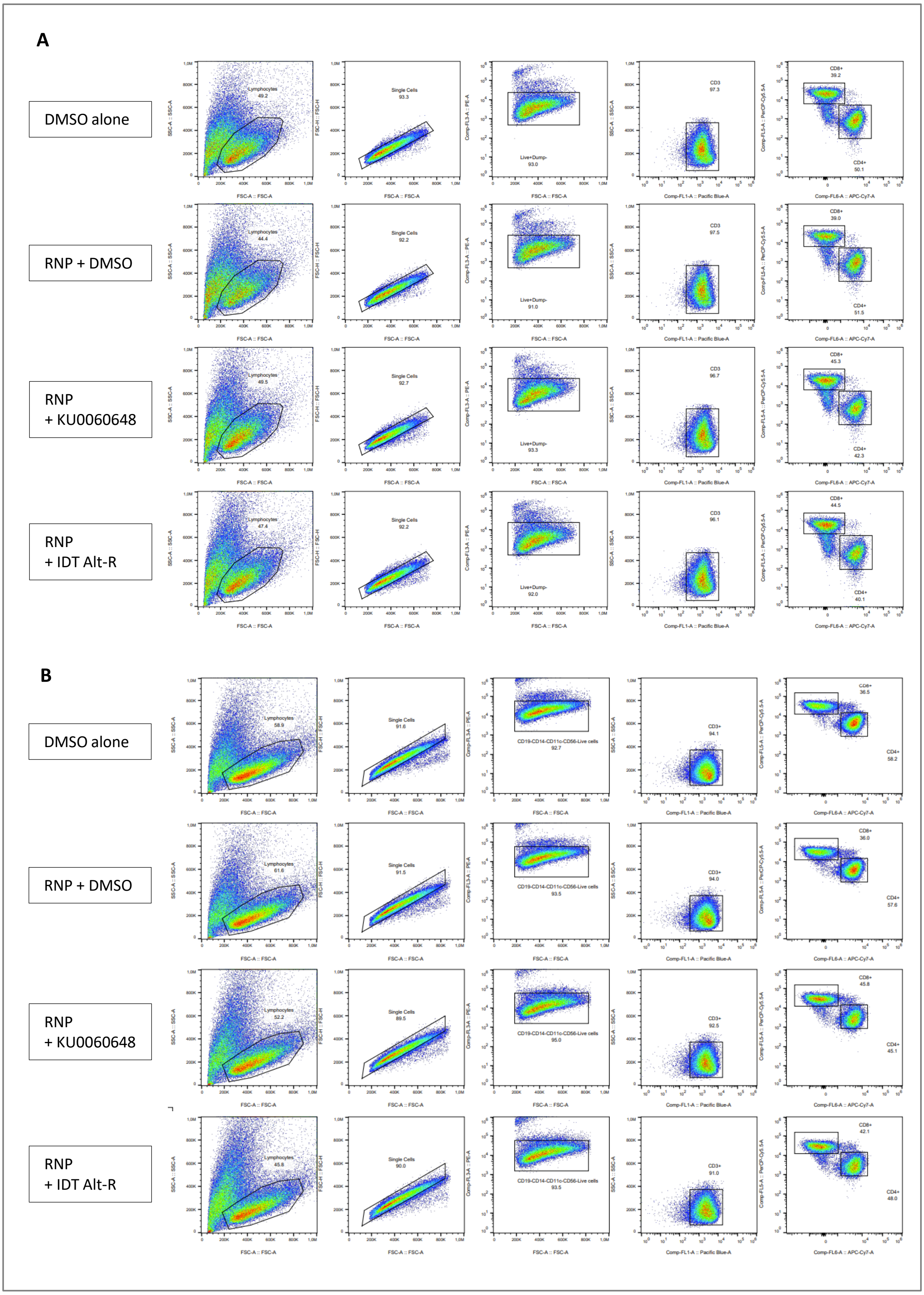

Figure S10. FACS sorting panel for scRNA-seq in DADA2 patient and healthy control

FACS gating strategy of unedited and ADA2-edited CD4+ and CD8+ T cells in (A) HD and (B) DADA2 patient. Cells were nucleofected on day 4 of the platform and treated with 0.5 μM KU0060648, 0.6 μM IDT Alt-R enhancer V2 or DMSO for 24h after nucleofection. Samples were collected for FACS on day 8 of the platform. One independent experiment was performed for all sets of data. Abbreviations: scRNA-seq (single-cell RNA sequencing), HD (healthy donor), RNP (ribonucleoprotein), DMSO (dimethyl sulfoxide), FACS (fluorescence-activated cell sorting), DADA2 (Deficiency of adenosine deaminase 2).

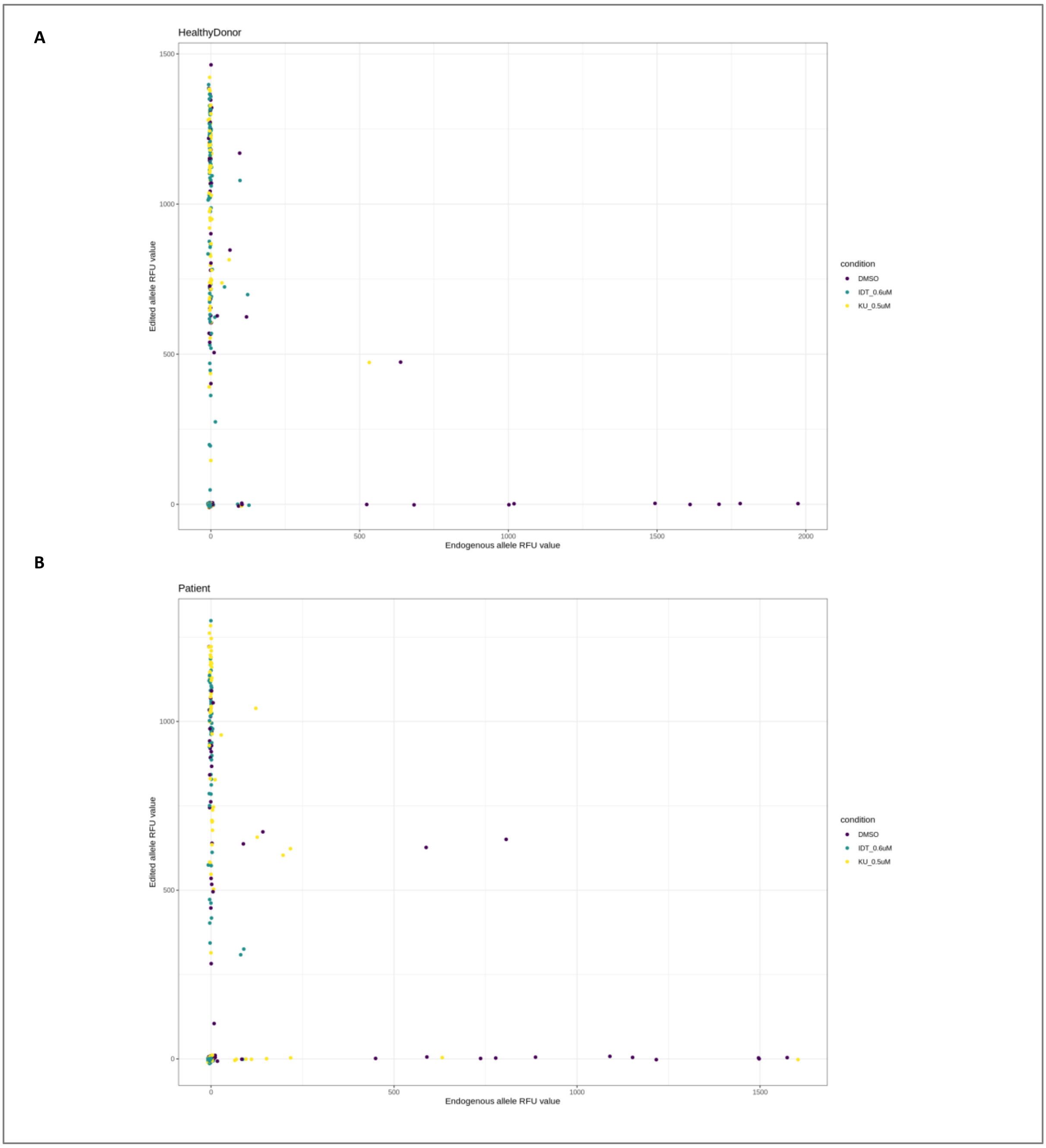

Figure S11. scRNA-seq RT-qPCR plots in healthy control and DADA2 patient

(A) RT-qPCR plots for edited HD and (B) DADA2 patient. Plots show RFU values of edited allele on y axis and endogenous allele on x axis. Each dot is measurement from a single cell. Dots are colored by which condition the cells underwent editing, DMSO in purple, 0.6 µM IDT Alt-R enhancer V2 in green and 0.5µM KU0060648 in yellow. Abbreviations: scRNA-seq (single-cell RNA sequencing), HD (healthy donor), DADA2 (Deficiency of adenosine deaminase 2), RT-qPCR (reverse transcription-qPCR), RFU (relative fluorescence units).

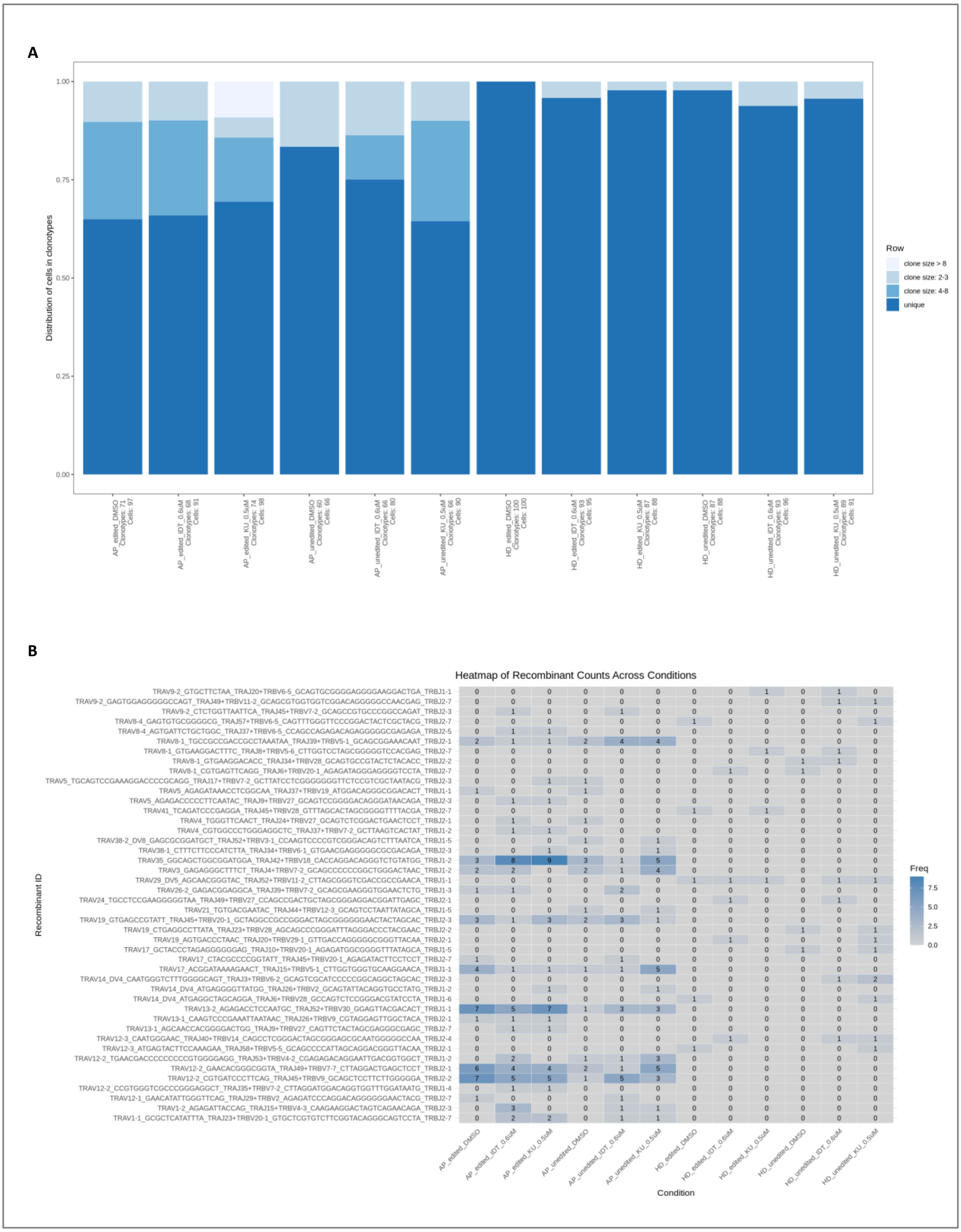

Figure S12. scRNA-seq TCR analysis in DADA2 patient and healthy control

(A) TCR repertoire analysis from scRNA-seq across experimental conditions for DADA2 patient ("AP") and healthy donor ("HD"). (B) Heatmap of recombinant counts across

conditions, reported as frequencies. One independent experiment was performed for all sets of data. Abbreviations: scRNA-seq (single-cell RNA sequencing), TCR (T cell receptor), HD (healthy donor), RNP (ribonucleoprotein), DMSO (dimethyl sulfoxide), DADA2 (Deficiency of adenosine deaminase 2).

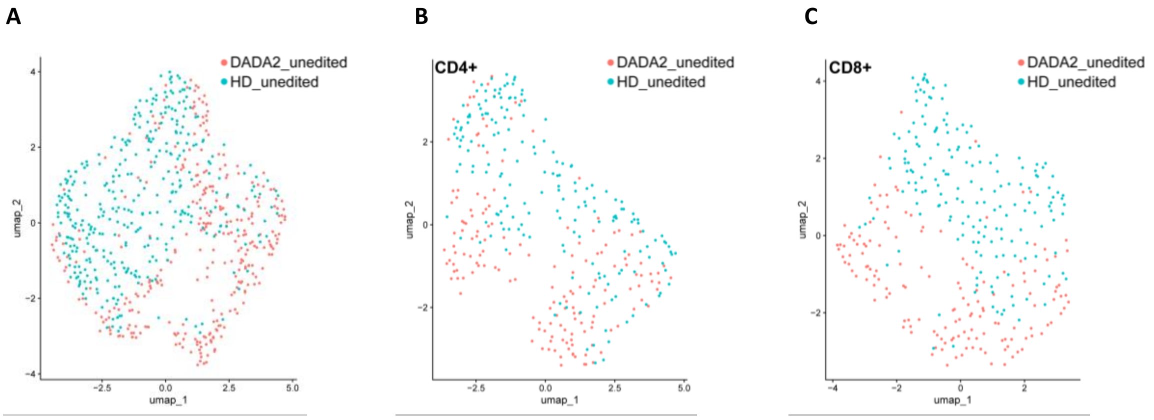

D

Total	P adjusted	NES
INTERFERON_ALPHA_RESPONSE	8.9e-06	-2,11
INTERFERON_GAMMA_RESPONSE	3.7e−03	-1,65
TNFA_SIGNALING_VIA_NFKB	3.7e−03	1,66
MITOTIC_SPINDLE	3.6e−02	-1,5
**CD4+**	**P adjusted**	**NES**
TNFA_SIGNALING_VIA_NFKB	2.1e−05	1,99
INTERFERON_ALPHA_RESPONSE	2.1e−05	-2,09
IL2_STAT5_SIGNALING	2.1e−03	1,70
ALLOGRAFT_REJECTION	2.1e−03	1,69
INTERFERON_GAMMA_RESPONSE	2.6e−03	-1,65
EPITHELIAL_MESENCHYMAL_TRANSITION	5.8e−03	1,7
COAGULATION	1.9e−02	1,66
ESTROGEN_RESPONSE_LATE	3.3e−02	1,51
ANGIOGENESIS	3.3e−02	1,75
KRAS_SIGNALING_UP	3.3e−02	1,49
**CD8+**	**P adjusted**	**NES**
INTERFERON_ALPHA_RESPONSE	4.0e−06	-2,12
INTERFERON_GAMMA_RESPONSE	1.7e−05	-1,86
KRAS_SIGNALING_UP	1.1e−04	-1,88
IL2_STAT5_SIGNALING	1.1e−04	-1,78
MYOGENESIS	1.4e−03	-1,76
MITOTIC_SPINDLE	3.6e−03	-1,59
XENOBIOTIC_METABOLISM	1.9e−02	-1,58
OXIDATIVE_PHOSPHORYLATION	2.5e−02	-1,45
UV_RESPONSE_DN	3.3e−02	1,48
COMPLEMENT	3.7e−02	-1,43

Figure S13. scRNA-seq UMAP plots comparing DADA2 patient to unedited healthy control

UMAP plots generated from scRNA-seq of unedited DADA2 patient for (A) total, (B) CD4+ and (C) CD8+ T cells, compared to unedited HD (DMSO). (D) Hallmark gene set enrichment results for unedited DADA2 patient (total, CD4+ and CD8+) compared to unedited HD

(DMSO). One independent experiment was performed for all sets of data. Abbreviations: scRNA-seq (single-cell RNA sequencing), HD (healthy donor), DADA2 (Deficiency of adenosine deaminase 2), UMAP (Uniform Manifold Approximation and Projection, NES (normalized enrichment score).

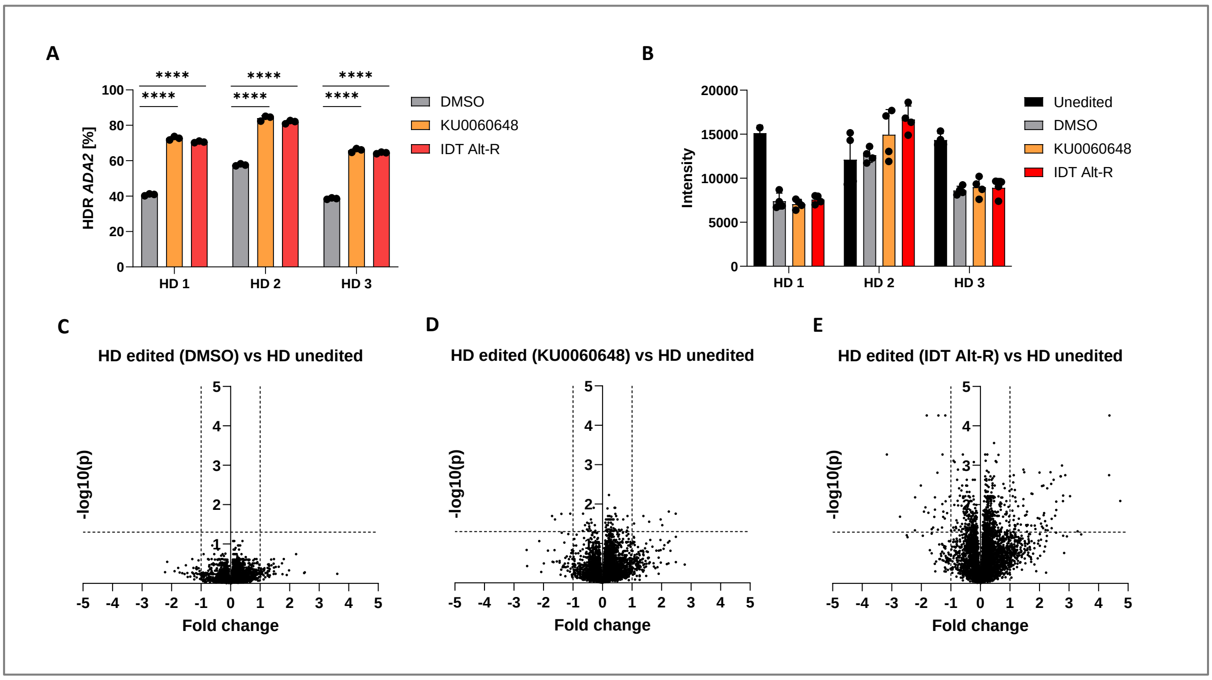

Figure S14. Mass spectrometry analysis of *ADA2*-edited and unedited healthy control T cells

(A) *ADA2* HDR editing in three HDs treated with HDR enhancers (0.5μM KU0060648, 0.6μM IDT Alt-R enhancer V2) or DMSO, assessed by ddPCR (n=3 technical replicates). (B) Abundance of ADA2 protein in three unedited and *ADA2*-edited HDs, reported as intensities (n=4 technical replicates). Comparison of protein expression levels in (C) *ADA2*-edited DMSO-treated HDs to unedited HDs, (D) *ADA2*-edited KU0060648-treated HDs to unedited HDs, (E) *ADA2*-edited IDT Alt-R enhancer V2 -treated HDs to unedited HDs, assessed by mass spectrometry. For (c)-(e), volcano plots were created by reporting protein expression fold change from mean of three HDs on the x axis and –log10 p value on the y axis. One independent experiment was performed for all sets of data. Statistical significance was assessed by one-way

ANOVA with Fisher's LSD test, where ****p<0.0001. Bar denotes mean value, error bars represent ± SD. Bar denotes mean value, error bars represent ± SD. Abbreviations: HDR (homology-directed repair), HD (healthy donor), DMSO (dimethyl sulfoxide), RNP (ribonucleoprotein), ddPCR (Droplet Digital PCR).

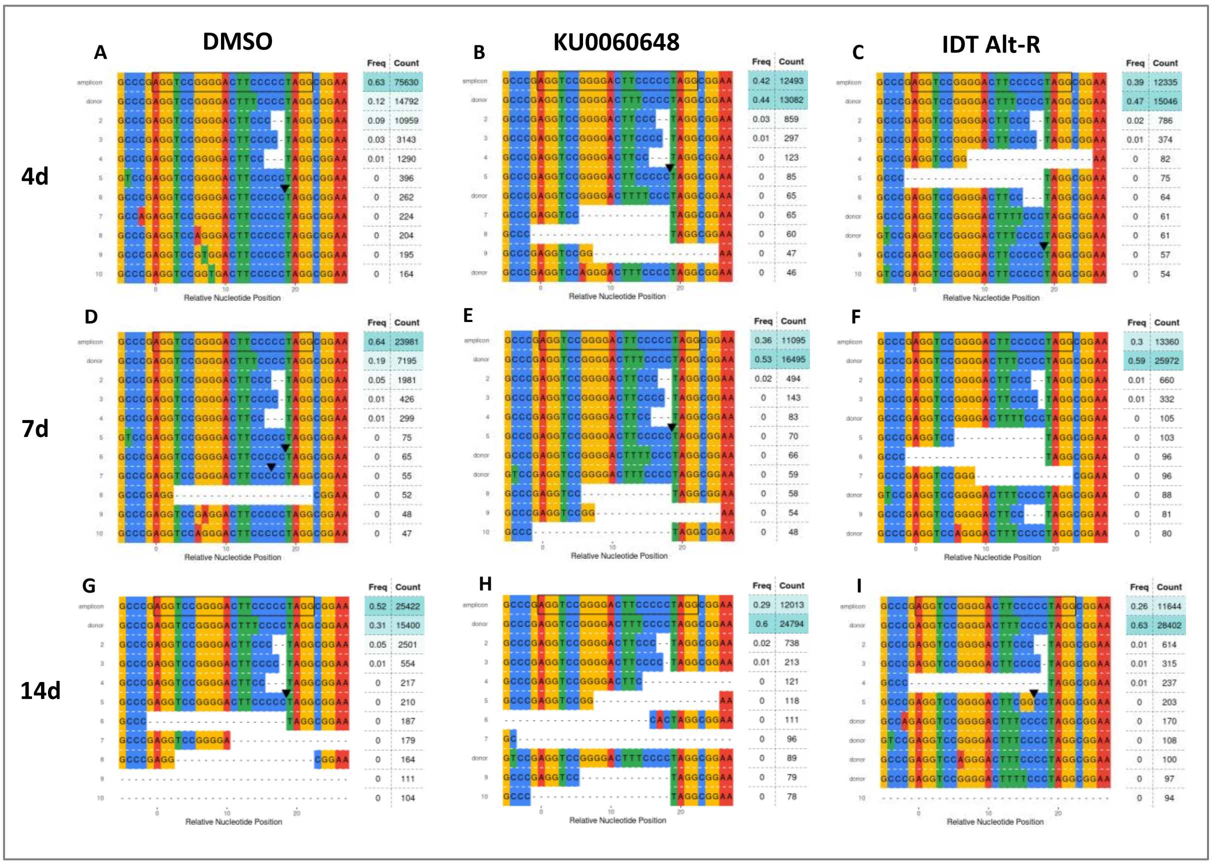

Figure S15. Amplicon sequencing variant plots in Cartilage hair hypoplasia patient CHH 1

Amplicon sequencing variant plots in *RMRP*-corrected CHH patient (CHH 1) treated with HDR enhancing compounds (0.5 µM KU0060648 and 0.6 µM IDT Alt-R enhancer V2) or DMSO, where samples were collected four (A-C), seven (D-F) and fourteen (G-I) days after nucleofection, shown in Fig. 7A. Edits are characterized on the left side of the plot, where "amplicon" is the unedited mutant sequence, "donor" below the "amplicon" is the perfect HDR (mutation correction) and imperfect HDR or indels in the rows below "amplicon". Arrow

indicates an insertion. Frequencies and sequencing counts are reported on the right side of the plot. HDR was assessed by amplicon sequencing, where one representative measurement is shown (n=2 technical replicates). One independent experiment was performed for all sets of data. The patient number corresponds to patient information in Supplemental Table S15. Abbreviations: CHH (Cartilage Hair Hypoplasia).

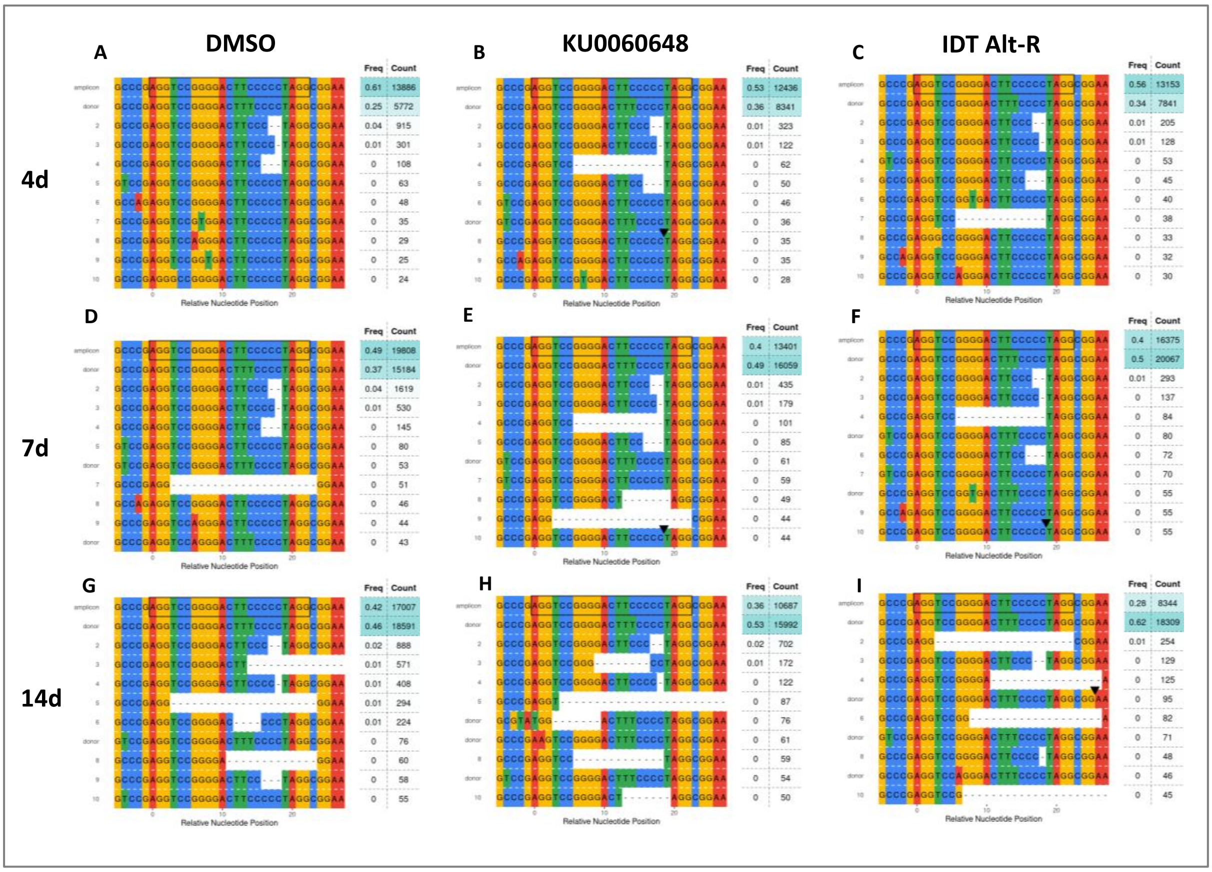

Figure S16. Amplicon sequencing variant plots in Cartilage hair hypoplasia patient CHH 2

Amplicon sequencing variant plots in *RMRP*-corrected CHH patient (CHH 2) treated with HDR enhancing compounds (0.5 µM KU0060648 and 0.6 µM IDT Alt-R enhancer V2) or DMSO, where samples were collected four (A-C), seven (D-F) and fourteen (G-I) days after nucleofection, shown in Fig. 7A. Edits are characterized on the left side of the plot, where "amplicon" is the unedited mutant sequence, "donor" below the "amplicon" is the perfect HDR

(mutation correction) and imperfect HDR or indels in the rows below “amplicon”. Arrow indicates an insertion. Frequencies and sequencing counts are reported on the right side of the plot. HDR was assessed by amplicon sequencing, where one representative measurement is shown (n=2 technical replicates). One independent experiment was performed for all sets of data. The patient number corresponds to patient information in Supplemental Table S15. Abbreviations: CHH (Cartilage Hair Hypoplasia).

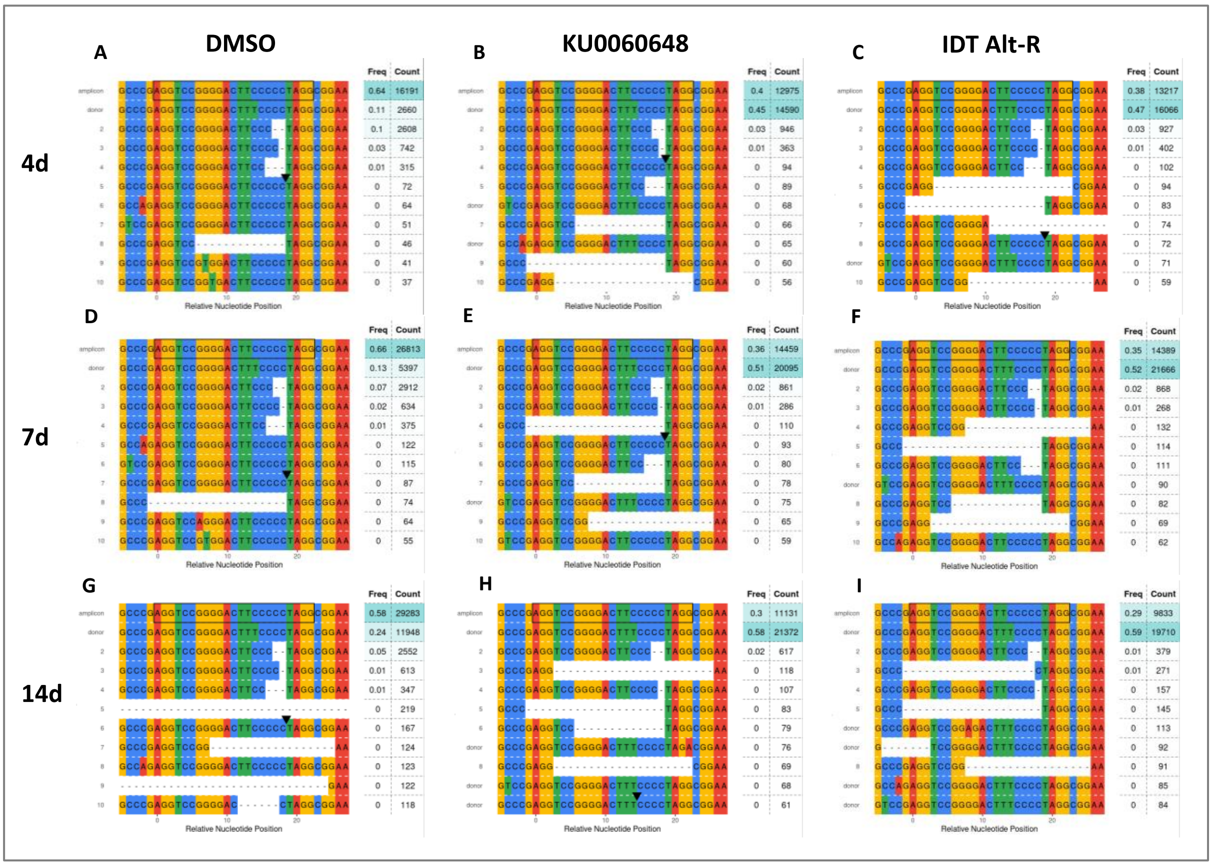

Figure S17. Amplicon sequencing variant plots in Cartilage hair hypoplasia patient CHH 3

Amplicon sequencing variant plots in *RMRP*-corrected CHH patient (CHH 3) treated with HDR enhancing compounds (0.5 µM KU0060648 and 0.6 µM IDT Alt-R enhancer V2) or DMSO, where samples were collected four (A-C), seven (D-F) and fourteen (G-I) days after nucleofection, shown in Fig. 7A. Edits are characterized on the left side of the plot, where

"amplicon" is the unedited mutant sequence, "donor" below the "amplicon" is the perfect HDR (mutation correction) and imperfect HDR or indels in the rows below "amplicon". Arrow indicates an insertion. Frequencies and sequencing counts are reported on the right side of the plot. HDR was assessed by amplicon sequencing, where one representative measurement is shown (n=2 technical replicates). One independent experiment was performed for all sets of data. The patient number corresponds to patient information in Supplemental Table S15. Abbreviations: CHH (Cartilage Hair Hypoplasia).

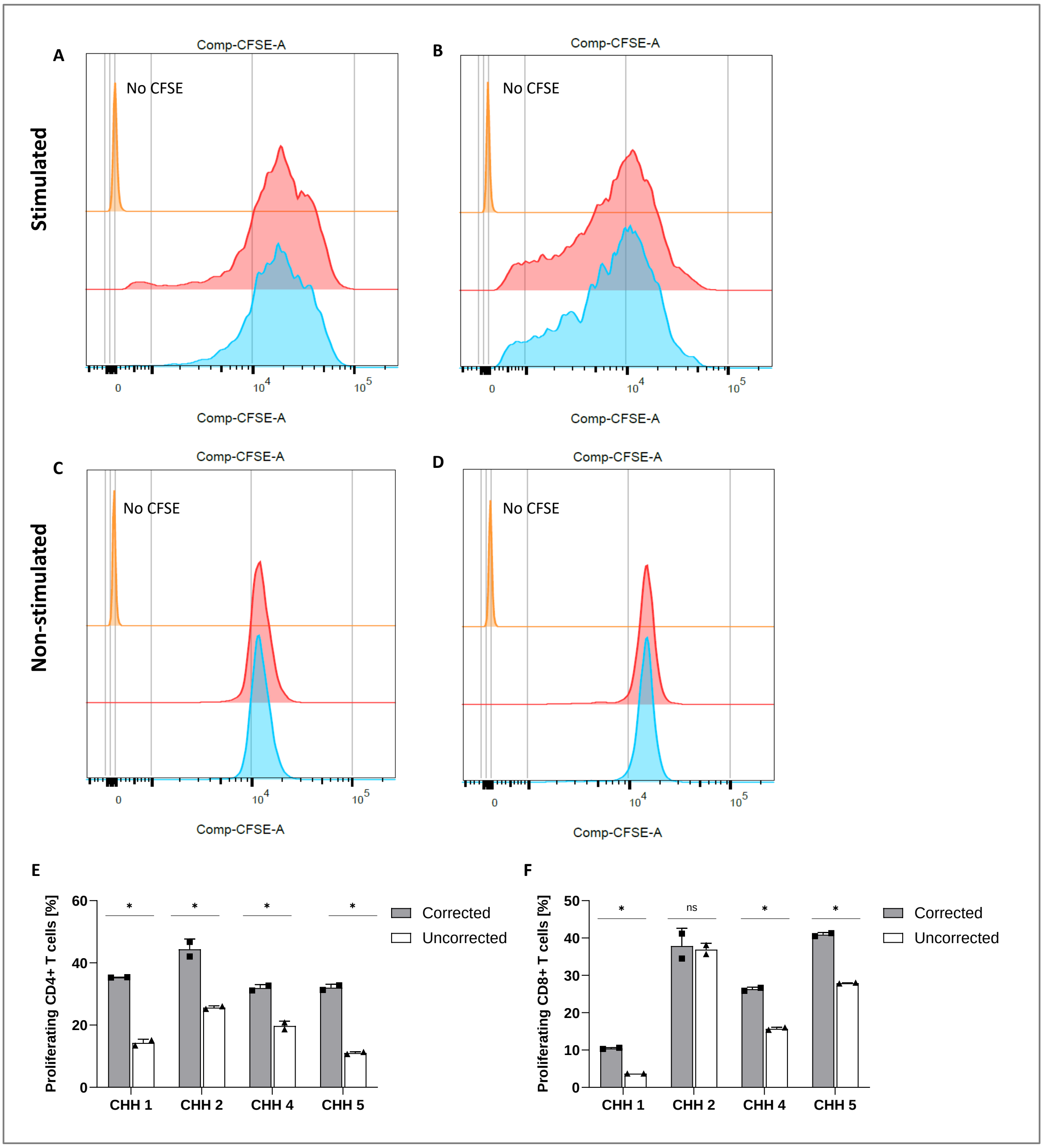

Figure S18. CFSE T cell proliferation assay in healthy control and Cartilage hair hypoplasia patient T cells

T cell proliferation assay in stimulated, mock electroporated (A) CD4+ and (B) CD8+ T cells, assessed by flow cytometry. T cell proliferation assay in unstimulated PBMCs in (C) CD4+ and (D) CD8+ T cells, assessed by flow cytometry. Frequency of proliferating (E) CD4+ and (F) CD8+ T cells in corrected and uncorrected CHH patients (CHH 1-5) shown in in Fig. 7C-

D were assessed by flow cytometry. CFSE signal in unstimulated PBMCs (c-d) were used as a gating control to assess frequency of proliferation in patients. The patient number corresponds to patient information in Supplemental Table S15. One independent experiment was performed for all sets of data. Bar denotes mean value, error bars represent ± SD. Statistical significance was assessed by performing unpaired t-test where * shows significance ($p<0.05$). Abbreviations: CHH (Cartilage hair hypoplasia).

SUPPLEMENTAL TABLES

Table S1: Preclinical evidence of T cell editing in IEI patients

IEI (gene)	Editing approach	Target cell type	Delivery strategy	Study highlights
CTLA4 Haploinsufficiency (*CTLA4*)[1]	cDNA knock-in to restore CTLA-4 expression	Patient CD4+ T cells and T_{regs}	Cas9 RNP + AAV6 HDR donor	Rescued CTLA-4 expression in CD4+ T cells; restoration of transendocytosis in T_{regs}. Engraftment and prevention of lymphoproliferation *in vivo* in a murine model of CTLA insufficiency.
Familial Hemophagocytic Lymphohistiocytosis (*PRF1, UNC13D*)[2]	HDR-mediated precise repair of cytotoxicity-related genes	Patient $CD8^+$ T cells	Cas9 RNP + AAV donor	Restored perforin or granule exocytosis; recovery of cytotoxic activity against target cells
IL-2Rα deficiency (*IL2RA/CD25*)[3]	Precise repair of pathogenic *IL2RA* variants	Patient CD4+ and CD8+ T cells	Cas9 RNP + ssDNA/dsDNA donor (non-viral)	Restored IL-2 expression; increased STAT5 phosphorylation
IPEX (*FOXP3*)[4]	cDNA knock-in to restore FOXP3 expression	Patient T_{eff} and T_{reg}	Cas9 RNP + rAAV6 HDR donor	Restored FOXP3 expression; regulation of T_{eff} proliferation; recovered suppressive T_{reg} function
X-linked Hyper-IgM Syndrome (*CD40LG*)[5]	cDNA knock-in to restore CD40LG expression	Patient $CD4^+$ T cells	Cas9 RNP + AAV6/IDLV donor	Restored CD40L expression; rescued B-cell help/class-switching
X-linked Hyper-IgM Syndrome (*CD40LG*)[6]	cDNA knock-in to restore CD40LG expression	Patient $CD4^+$ T cells	Cas9 RNP + IDLV donor	GMP-compatible & scalable manufacturing of edited T cells; restored CD40LG expression; engraftment upon xenotransplantation
X-linked Lymphoproliferative Disease (*SH2D1A*)[7]	cDNA knock-in to restore SAP expression	Patient $CD4^+$ and $CD8^+$ T cells	Cas9 RNP + AAV6 donor	Restored SAP expression; restored sensitivity to restimulation-induced

				cell death; restored T:B cell signalling; T_{FH} support; improved cytotoxic functions
X-linked Lymphoproliferative Disease (*SH2D1A*)[8]	Stable SAP expression using precise promoter-driven expression cassette	Patient $CD4^{+}$ and $CD8^{+}$ T cells	Lentiviral vectors	Restored SAP expression; restored sensitivity to restimulation-induced cell death

Table S2: gRNA sequences for *ADA2*, *AIRE*, *RMRP* and *STAT1*

Locus	gRNA	gRNA sequence 5'→3'	gRNA orientation	gRNA length (bp)
ADA2	g#1	ttccaagtggattctgctgg	FWD	20
ADA2	g#2 (WT)	gctggaggattatcggaagc	FWD	20
ADA2	g#2 (MUT)	gctggaggattatcagaagc	FWD	20
ADA2	g#3 (WT)	tgctggaggattatcggaag	FWD	20
ADA2	g#3 (MUT)	tgctggaggattatcagaag	FWD	20
ADA2	g#4	ggattctgctggaggattat	FWD	20
ADA2	g#5	atgttccaagtggattctgc	FWD	20
ADA2	g#6	catcagaaaaatgttccaag	FWD	20
ADA2	g#7	atcctccagcagaatccact	REV	20
AIRE	g#1	cagcagtggcccgaagcctc	FWD	20
AIRE	g#2 (MUT)	gaagcctctggtttgagcca	FWD	20
AIRE	g#3 (MUT)	aagcctctggtttgagccaa	FWD	20
AIRE	g#4 (MUT)	gtttgagccaagggagccca	FWD	20
AIRE	g#5 (MUT)	ggtttgagccaagggagccc	FWD	20
AIRE	g#6	aacaaggcccgcagcagcag	FWD	20
AIRE	g#7	cttcgggccactgctgctgc	REV	20
AIRE	g#8	gcttcgggccactgctgctg	REV	20
AIRE	g#9 (MUT)	tggctcaaaccagaggcttc	REV	20
AIRE	g#10 (MUT)	ttggctcaaaccagaggctt	REV	20
AIRE	g#11 (WT)	gctcccttggctcgaaccag	REV	20
AIRE	g#11 (MUT)	gctcccttggctcaaaccag	REV	20
AIRE	g#12 (MUT)	gctcccttggctcaaaccag	REV	20
AIRE	g#13	ggcagcgccctgggctccct	REV	20
AIRE	g#14	gcttacgggggcagcgccct	REV	20
AIRE	g#15	tgcttacgggggcagcgccc	REV	20
AIRE	g#16	ggaaggtcaggtgcttacgg	REV	20
AIRE	g#17	gggaaggtcaggtgcttacg	REV	20
AIRE	g#18	agggaaggtcaggtgcttac	REV	20

AIRE	g#19	cagggaaggtcaggtgctta	REV	20
RMRP	g#1	cactctctgcccgaggtccg	FWD	20
RMRP	g#2	tgtctacgtgcgtatgcacg	FWD	20
RMRP	g#3	cacgtggcactctctgcccg	FWD	20
RMRP	g#4	ggcactctctgcccgaggtc	FWD	20
RMRP	g#5	gcactctctgcccgaggtcc	FWD	20
RMRP	g#6 (MUT)	aggtccggggacttcccct	FWD	20
RMRP	g#7 (MUT)	tccggggacttcccccctagg	FWD	20
RMRP	g#8 (MUT)	ggacttcccccctaggcggaa	FWD	20
RMRP	g#9 (WT)	gactttcccctaggcggaaa	FWD	20
RMRP	g#9 (MUT)	gacttcccccctaggcggaaa	FWD	20
RMRP	g#10 (MUT)	acttcccccctaggcggaaag	FWD	20
RMRP	g#11 (MUT)	tcccccctaggcggaaagggg	FWD	20
RMRP	g#12	gagtcctcagtgtgtagcct	FWD	20
RMRP	g#13 (MUT)	gggggaagtccccggacctc	REV	20
RMRP	g#14 (MUT)	agggggaagtccccggacct	REV	20
RMRP	g#15 (MUT)	tccgcctagggggaagtccc	REV	20
RMRP	g#16	ttcctcccctttccgcctag	REV	20
STAT1	g#1 (MUT)	gcacacaagagtgatgaaca	FWD	20
STAT1	g#2 (MUT)	cacaagagtgatgaacatgg	FWD	20
STAT1	g#3	aacatggaggagtccaccaa	FWD	20

Table S3: ssODN sequences for *ADA2*, *AIRE*, *RMRP* and *STAT1*

Locus	Repair strategy	ssODN symmetry	ssODN direction (5'→3')	ssODN sequence (100 bp)
ADA2	WT/MUT →SNP	Left 40nt	fwd	cccaaggggggatcatgcagttcagatttgctcacccaactccccgtccatcagaaaaatgttccaagtggattctgctggaggactacagaaagcgggtg
ADA2	WT/MUT →SNP	Left 30nt	fwd	atcatgcagttcagatttgctcacccaactccccgtccatcagaaaaatgttccaagtggattctgctggaggactacagaaagcgggtgcagaacgtca
ADA2	WT/MUT →SNP	Left 20nt	fwd	tcagatttgctcacccaactccccgtccatcagaaaaatgttccaagtggattctgctggaggactacagaaagcgggtgcagaacgtcactgagtttga
ADA2	WT/MUT →SNP	Left 10nt	fwd	tcacccaactccccgtccatcagaaaaatgttccaagtggattctgctggaggactacagaaagcgggtgcagaacgtcactgagtttgatgacaggtga
ADA2	WT/MUT →SNP	Middle	fwd	ccccgtccatcagaaaaatgttccaagtggattctgctggaggactacagaaagcgggtgcagaacgtcactgagtttgatgacaggtgagtagtagttc
ADA2	WT/MUT →SNP	Right 10nt	fwd	cagaaaaatgttccaagtggattctgctggaggactacagaaagcgggtgcagaacgtcactgagtttgatgacaggtgagtagtagttcagaaagcaca

ADA2	WT/MUT →SNP	Right 20nt	fwd	ttccaagtggattctgctggaggactacagaaagcgggtgcagaacgtcactgagtttgatgacaggtgagtagtagttcagaaagcacatgtcccaggc
ADA2	WT/MUT →SNP	Right 30nt	fwd	ttctgctggaggactacagaaagcgggtgcagaacgtcactgagtttgatgacaggtgagtagtagttcagaaagcacatgtcccaggcctgtcatgggg
ADA2	WT/MUT →SNP	Right 40nt	fwd	aggactacagaaagcgggtgcagaacgtcactgagtttgatgacaggtgagtagtagttcagaaagcacatgtcccaggcctgtcatggggtggcagtgg
AIRE	WT/MUT →SNP	Left 40nt	rev	cccttggcacgtaccaaaggcttcgggccactgctgctgcgggccttgttcttcccactgccggagtcttcgaacttgctgggagtgtagaactccccgc
AIRE	WT/MUT →SNP	Left 30nt	rev	gccctgggctcccttggcacgtaccaaaggcttcgggccactgctgctgcgggccttgttcttcccactgccggagtcttcgaacttgctgggagtgtag
AIRE	WT/MUT →SNP	Left 20nt	rev	cgggggcagcgccctgggctcccttggcacgtaccaaaggcttcgggccactgctgctgcgggccttgttcttcccactgccggagtcttcgaacttgct
AIRE	WT/MUT →SNP	Left 10nt	rev	caggtgcttacgggggcagcgccctgggctcccttggcacgtaccaaaggcttcgggccactgctgctgcgggccttgttcttcccactgccggagtctt
AIRE	WT/MUT →SNP	Middle	rev	cagggaaggtcaggtgcttacgggggcagcgccctgggctcccttggcacgtaccaaaggcttcgggccactgctgctgcgggccttgttcttcccactg
AIRE	WT/MUT →SNP	Right 10nt	rev	ccaggctccccagggaaggtcaggtgcttacgggggcagcgccctgggctcccttggcacgtaccaaaggcttcgggccactgctgctgcgggccttgtt
AIRE	WT/MUT →SNP	Right 20nt	rev	gcatcaagagccaggctccccagggaaggtcaggtgcttacgggggcagcgccctgggctcccttggcacgtaccaaaggcttcgggccactgctgctgc
AIRE	WT/MUT →SNP	Right 30nt	rev	ggggcggggggcatcaagagccaggctccccagggaaggtcaggtgcttacgggggcagcgccctgggctcccttggcacgtaccaaaggcttcgggcca
AIRE	WT/MUT →SNP	Right 40nt	rev	cgctgttcctggggcggggggcatcaagagccaggctccccagggaaggtcaggtgcttacgggggcagcgccctgggctcccttggcacgtaccaaagg
RMRP	WT→SNP	Left 40nt	fwd	gatacgcttcttggcggactttggagtgggaagcggggaatgtctacgtgcgtatgcacgtggcactctctgcccgaggtccggggacttccacataggc
RMRP	WT→SNP	Left 30nt	fwd	ttggcggactttggagtgggaagcggggaatgtctacgtgcgtatgcacgtggcactctctgcccgaggtccggggacttccacataggcggaaagggga
RMRP	WT→SNP	Left 20nt	fwd	ttggagtgggaagcggggaatgtctacgtgcgtatgcacgtggcactctctgcccgaggtccggggacttccacataggcggaaaggggaggaacagagt
RMRP	WT→SNP	Left 10nt	fwd	aagcggggaatgtctacgtgcgtatgcacgtggcactctctgcccgaggtccggggacttccacataggcggaaaggggaggaacagagtcctcagtgtg
RMRP	WT→SNP	Middle	fwd	tgtctacgtgcgtatgcacgtggcactctctgcccgaggtccggggacttccacataggcggaaaggggaggaacagagtcctcagtgtgtagcctagga
RMRP	WT→SNP	Right 10nt	fwd	cgtatgcacgtggcactctctgcccgaggtccggggacttccacataggcggaaaggggaggaacagagtcctcagtgtgtagcctaggatacaggcctt
RMRP	WT→SNP	Right 20nt	fwd	tggcactctctgcccgaggtccggggacttccacataggcggaaaggggaggaacagagtcctcagtgtgtagcctaggatacaggccttcagcacgaac
RMRP	WT→SNP	Right 30nt	fwd	tgcccgaggtccggggacttccacataggcggaaaggggaggaacagagtcctcagtgtgtagcctaggatacaggccttcagcacgaaccacgtcctca
RMRP	WT→SNP	Right 40nt	fwd	ccggggacttccacataggcggaaaggggaggaacagagtcctcagtgtgtagcctaggatacaggccttcagcacgaaccacgtcctcagcttcacaga
RMRP	MUT→SNP	Middle	fwd	tgtctacgtgcgtatgcacgtggcactctctgcccgaggtccggggactttcacataggcggaaaggggaggaacagagtcctcagtgtgtagcctagga
RMRP	MUT→WT	Middle	fwd	tgtctacgtgcgtatgcacgtggcactctctgcccgaggtccggggactttcccctaggcggaaaggggaggaacagagtcctcagtgtgtagcctagga
STAT1	MUT→ SNP	Left 30nt	fwd	taatagttggaagacttttcagcatttctttcctatattgtatagatttaggaagttcaacattttgggcacgcacacgaaggttatgaacatggaggag

STAT1	MUT→ SNP	Left 20nt	fwd	aagacttttcagcatttctttcctatattgtatagatttaggaagttcaacattttgggcacgcacacgaaggttatgaacatggagg agtccaccaatg
STAT1	MUT→ SNP	Left 10nt	fwd	agcatttctttcctatattgtatagatttaggaagttcaacattttgggcacgcacacgaaggttatgaacatggaggagtccacca atggcagtctggc
STAT1	MUT→ SNP	Middle	fwd	tcctatattgtatagatttaggaagttcaacattttgggcacgcacacgaaggttatgaacatggaggagtccaccaatggcagtc tggcggctgaattt
STAT1	MUT→ SNP	Right 10nt	fwd	tatagatttaggaagttcaacattttgggcacgcacacgaaggttatgaacatggaggagtccaccaatggcagtctggcggct gaatttcggcacctgg
STAT1	MUT→ SNP	Right 20nt	fwd	ggaagttcaacattttgggcacgcacacgaaggttatgaacatggaggagtccaccaatggcagtctggcggctgaatttcgg cacctggtagggacatc
STAT1	MUT→ SNP	Right 30nt	fwd	cattttgggcacgcacacgaaggttatgaacatggaggagtccaccaatggcagtctggcggctgaatttcggcacctggtag ggacatcagtttcctct

Table S4: Markers used for immune cell characterization

**Marker**	**Color**	**Clone**	**Vendor**	**Catalog**	**Dilution**
CD14	PerCP-Cy5.5	61D3	eBioscoence	45-0149-42	1:200
CD15	FITC	3G8	Biolegend	302001	1:200
CD56	FITC	NCAM16.2	BD	664524	1:200
CD4	AlexaFluor700	RPA-T4	BD Pharmingen	557922	1:200
CD3	BV421	UCHT1	BD Horizon	562426	1:200
CD20	BV786	2H7	Biolegend	302356	1:100
CD8	PE	4B9	eBioscience	12-0087-42	1:100
LiveDead near IR	N/A	N/A	Thermo Fisher	L34992	1:1000

Table S5: Markers used for CD4+ and CD8+ T cell sorting panel

**Marker**	**Color**	**Clone**	**Vendor**	**Catalog**	**Dilution**
CD19	PE	HIB19	BioLegend	302207	1:100
CD14	PE	HCD14	BioLegend	325605	1:100
CD11c	PE	3.9	BioLegend	301605	1:100
CD56	PE	HCD56	BioLegend	318305	1:100
CD3	Pacific Blue	SK7	BioLegend	344823	1:50
CD4	APC-Cy7	OKT4	BioLegend	317417	1:50
CD8a	PerCP-Cy5.5	RPA-T8	BioLegend	301031	1:100
LIVE/DEAD Fixable Red	N/A	N/A	Invitrogen	L34971	1:500

Table S6: Antibodies and reagents used for CFSE T cell proliferation assay

Marker	Color	Clone	Vendor	Catalog	Dilution
CFSE	CFSE	N/A	Invitrogen	C34554A	1µM
CD19	Pacific Blue	HIB19	Biolegend	302232	1:200
CD14	Pacific Blue	M5E2	Biolegend	301828	1:200
CD56	Pacific Blue	MEM-188	Biolegend	304629	1:200
CD11c	V450	B-ly6	BD Biosciences	560369	1:200
Live/Dead Fixable Violet	N/A	N/A	Invitrogen	L34963	1:500
CD8a	Alexa Fluor 594	RPA-T8	Biolegend	301056	1:100
CD4	APC-Cy7	OKT4	BioLegend	317417	1:100

Table S7. Antibodies used for assessment of phosphorylated STAT1

Marker	Color	Clone	Vendor	Catalog	Dilution
CD3	Alexa Flour 488	UCHT1	Biolegend	300415	1:500
CD19	Pacific Blue	HIB19	Biolegend	302232	1:1000
CD14	Pacific Blue	M5E2	Biolegend	301828	1:1000
CD56	Pacific Blue	MEM-188	Biolegend	304629	1:1000
CD11c	V450	B-ly6	BD Biosciences	560369	1:1000
Live/Dead Fixable Violet	N/A	N/A	Invitrogen	L34963	1:500
pSTAT1 (Y701)	Alexa Fluor 647		BD Biosciences	612597	1:10

Table S8: ddPCR oligos for ADA2, AIRE, RMRP and STAT-1

Target gene	ddPCR primer fwd	ddPCR primer rev	ddPCR probe reference	ddPCR probe HDR	ddPCR probe NHEJ
ADA2	GGTGAGGAATGTCACCTACA	GTACCAAGGGAGACACCTAC	**WT&MUT:** /5'FAM/GCCACATCTGTTTCACCCCA/3'BHQ_1/	**WT/MUT→SNP:** /5'HEX/CTGGAGGACTACAGAAAGCGG/3'BHQ_1/	**WT→SNP:** /5'HEX/ATTATCGGAAGCGGGTGCAGA/3'BHQ_1/ **MUT→SNP:** /5'HEX/ATTATCAGAAGCGGGTGCAGA/3'BHQ_1/

AIRE	TCTACACTCCCAGCAAGTTC	GGAAGGTCAGGTGCTTACG	**WT&MUT:**<br><br>/5'FAM/TCCGGCAGTGGGAAGAACAA/3'BHQ_1/	**WT/MUT→SNP:**<br>/5'HEX/AAGCCTTTGGTACGTGCCAAG/3'BHQ_1/	**WT→SNP:**<br>/5'HEX/CGAAGCCTCTGGTTCGAGC/3'BHQ_1/<br><br>**MUT→SNP:**<br>/5'HEX/CGAAGCCTCTGGTTTGAGC/3'BHQ_1/
RMRP	GCTTCTTGGCGGACTTTG	ATACTACTCTGTGAAGCTGAGG	**WT&MUT:**<br><br>/5'FAM/TGGGAAGCGGGGAATGTCTA/3'BHQ_1/	**WT→SNP:**<br>/5'HEX/GGACTTCCACATAGGCGGAA/3'BHQ_1/<br><br>**MUT→SNP:**<br>/5'HEX/GGACTTTCACATAGGCGGAA/3'BHQ_1/	**WT→SNP:**<br>/5'HEX/ACTTTCCCCTAGGCGGAAAG/3'BHQ_1/<br><br>**MUT→SNP:**<br>/5'HEX/ACTTCCCCCTAGGCGGAAAG/3'BHQ_1/
STAT1	ACGTGACGTACTTTACGCTAT	GAAACTGATGTCCCTACCAGG	**WT & MUT:**<br>/5’FAM/AGTTGGAAGACTTTTCAGCATTTCT/3'BHQ_1/	**MUT→SNP:**<br>/5’HEX/ACGCACACGAAGGTTATGAACA/3'BHQ_1/	**MUT→SNP:**<br>/5’HEX/AAGAGTGATGAACATGGAGGAGT/3'BHQ_1/
CASC11 (external reference gene)	AGGTGGCTGGAAACTTGT	GGAGCAACCAATCGCTATG	**All edited loci:**<br>/5’FAM/CCTCGGACGCTCCTGCTCCT/3'BHQ_1/	N/A	N/A

Table S9: Amplicon-seq first PCR components

**Reagent**	**Vendor**	**Final concentration**
Nuclease-free water	Ambion	Add up to 20
5× Phusion GC Buffer	Thermo Fisher Scientific	1X
10 mM dNTPs	Thermo Fisher Scientific	200 µM
10 µM Primer fwd	IDT	0.5 µM
10 µM Primer rev	IDT	0.5 µM
Betaine	Sigma Aldrich	1 M
Phusion Hot Start II DNA Polymerase	Thermo Fisher Scientific	0.02 U/µl
Template DNA	N/A	100 ng

Table S10: Amplicon sequencing second PCR primers

i5-PCR Index 9	**AAT GAT ACG GCG ACC ACC GAG ATC TA**TTGCTTGC_AC ACT CTT TCC CTA CAC GAC GCT CTT CCG ATC* T_
i5-PCR Index 10	**AAT GAT ACG GCG ACC ACC GAG ATC TA**GAGAGGTT_AC ACT CTT TCC CTA CAC GAC GCT CTT CCG ATC* T_
i5-PCR Index 11	**AAT GAT ACG GCG ACC ACC GAG ATC TA**ACCTGGTT_AC ACT CTT TCC CTA CAC GAC GCT CTT CCG ATC* T_
i5-PCR Index 13	**AAT GAT ACG GCG ACC ACC GAG ATC TA**CGGAACAA_AC ACT CTT TCC CTA CAC GAC GCT CTT CCG ATC* T_
i5-PCR Index A505	**AAT GAT ACG GCG ACC ACC GAG ATC TA**CTAATCGA_AC ACT CTT TCC CTA CAC GAC GCT CTT CCG ATC* T_
i5-PCR Index A506	**AAT GAT ACG GCG ACC ACC GAG ATC TA**CTAGAACA_AC ACT CTT TCC CTA CAC GAC GCT CTT CCG ATC* T_
i5-PCR Index A507	**AAT GAT ACG GCG ACC ACC GAG ATC TA**TAAGTTCC_AC ACT CTT TCC CTA CAC GAC GCT CTT CCG ATC* T_
i5-PCR Index A508	**AAT GAT ACG GCG ACC ACC GAG ATC TA**TAGACCTA_AC ACT CTT TCC CTA CAC GAC GCT CTT CCG ATC* T_
i7-PCR Index 13	**CAA GCA GAA GAC GGC ATA CGA GAT**TTCCTCCT_G TGA CTG GAG TTC AGA CGT GTG CTC TTC CGA TC*T_
i7-PCR Index 14	**CAA GCA GAA GAC GGC ATA CGA GAT**TGCTTGCT_G TGA CTG GAG TTC AGA CGT GTG CTC TTC CGA TC*T_
i7-PCR Index 15	**CAA GCA GAA GAC GGC ATA CGA GAT**GGTGATGA_G TGA CTG GAG TTC AGA CGT GTG CTC TTC CGA TC*T_
i7-PCR Index 16	**CAA GCA GAA GAC GGC ATA CGA GAT**AACCTACG_G TGA CTG GAG TTC AGA CGT GTG CTC TTC CGA TC*T_
i7-PCR Index A705	**CAA GCA GAA GAC GGC ATA CGA GAT**ACCCAGCA_G TGA CTG GAG TTC AGA CGT GTG CTC TTC CGA TC*T_
i7-PCR Index A706	**CAA GCA GAA GAC GGC ATA CGA GAT**AACCCCTC_G TGA CTG GAG TTC AGA CGT GTG CTC TTC CGA TC*T_
i7-PCR Index A707	**CAA GCA GAA GAC GGC ATA CGA GAT**CCCAACCT_G TGA CTG GAG TTC AGA CGT GTG CTC TTC CGA TC*T_
i7-PCR Index A708	**CAA GCA GAA GAC GGC ATA CGA GAT**CACCACAC_G TGA CTG GAG TTC AGA CGT GTG CTC TTC CGA TC*T_
i7-PCR Index A709	**CAA GCA GAA GAC GGC ATA CGA GAT**GAAACCCA_G TGA CTG GAG TTC AGA CGT GTG CTC TTC CGA TC*T_
i7-PCR Index A710	**CAA GCA GAA GAC GGC ATA CGA GAT**TGTGACCA_G TGA CTG GAG TTC AGA CGT GTG CTC TTC CGA TC*T_
i7-PCR Index A711	**CAA GCA GAA GAC GGC ATA CGA GAT**AGGGTCAA_G TGA CTG GAG TTC AGA CGT GTG CTC TTC CGA TC*T_
i7-PCR Index A712	**CAA GCA GAA GAC GGC ATA CGA GAT**AGGAGTGG_G TGA CTG GAG TTC AGA CGT GTG CTC TTC CGA TC*T_

Table S11: Amplicon-seq second PCR components

Reagent	Vendor	Final concentration
Nuclease-free water	Ambion	Add up to 20

5× Phusion GC Buffer	Thermo Fisher Scientific	1X
10 mM dNTPs	Thermo Fisher Scientific	200 µM
10 µM Primer fwd	IDT	0.25 µM
10 µM Primer rev	IDT	0.25 µM
Betaine	Sigma Aldrich	1 M
Phusion Hot Start II DNA Polymerase	Thermo Fisher Scientific	0.02 U/µl
Template DNA	N/A	5 ng

Table S12: List of HDR enhancing compounds and tested concentrations

**Compound**	**Conc 1 (µM)**	**Conc 2 (µM)**	**Conc 3 (µM)**
ABT263 [9]	0,25	0,5	1
AICAR[10]	10	20	40
B02 [10]	10	20	40
Brefeldin A[11]	0,05	0,1	0,2
Entinostat[12]	2,5	5	10
EPZ5676[13]	0,05	0,1	0,2
IC86621[14]	100	200	400
KU0060648[15,16]	0,125	0,25	0,5
KU55933[17,18]	1,5	3	6
L755507[11]	2,5	5	10
Mirin[17]	1,5	3	6
MLN4924[10]	0,25	0,5	1
M3814[19]	1	2	4
Nexturastat A[20]	1,25	2,5	5
NSC 15520[10]	2,5	5	10
NSC 19630[10]	0,5	1	2
NU7026[10]	10	20	40
NU7441[16]	1	2	4
Panobinostat[12]	0,05	0,1	0,2

PFM01[17]	5	10	20
Resveratrol[21]	0,5	1	25
Ricolinostat[20]	1,25	2,5	5
Romidepsin[22,23]	0,01	0,025	0,1
RS-1[24]	5	10	20
Rucaparib[25,26]	2,5	5	10
SCR7 pyrazine	2,5	1	5
STL127705[10,27]	2,5	5	10
TDRL-505[17]	10	20	40
Trichostatin A[10,28]	0,005	0,01	0.1
Valproic acid[23,29]	5	10	20
Wortmannin[30]	0,01	0,02	0,04
Crispy mix[10]*	*	Not tested	Not tested
IDT ALT-R enhancer V2	1	Not tested	Not tested

*20 µM NU7026, 0.01 µM Trichostatin A, 0.5 µM MLN4924, 5uM NCS15520

Table S13: List of cell cycle inhibitors and tested concentrations

Compound	Conc 1 (µM)	Conc 2 (µM)	Conc 3 (µM)
ABT-751[31]	0,175*	0,35*	0,7*
Aphidicolin[32]	1*	2*	4*
AZD7762[33]	0,5	1	2
Hydroxy urea[32]	62,5	125	250
Lovastatin[32]	20	40	80
Mimosine[32]	100	200	400
Nocodazole[32]	0,1*	0,2*	0,4*
PHA-767491[34]	5	10	20
Thymidine[32]	1250	2500	5000
XL413[34]	5	10	20

***** Concentration reported as µg/mL instead of µM

Table S14: Reagents for scRNAseq

Reagent	Vendor

Maxima H Minus Reverse Transcriptase	Thermo Fisher
psfTn5	Addgene
KAPA HiFi HotStart ReadyMix	Roche
Lambda Exonuclease	BioNordika
Tween-20	Sigma Aldrich
10% SDS solution	Teknova
Magnesium Chloride (1 M)	Sigma Aldrich
Triton X-100	Sigma Aldrich
KAPA HiFi PCR kit with dNTPs	Roche
Betaine (5 M)	Sigma Aldrich
UltraPure DNase/RNase Free Distilled Water	Thermo Fisher
ERCC RNA Spike-In Mix	Thermo Fisher
USB Dithiothreitol (DTT, 0.1 M)	Thermo Fisher
RNase inhibitor	Takara Bio
dNTP Mix (dATP, dCTP, dGTP, and dTTP, each at 10 mM)	Thermo Fisher
SpeedBeads magnetic carboxylate modified particles	Merck
Peg8000	Sigma Aldrich
TAPS 0.2 M buffer soln., pH 8.5	Thermo Fisher
0.5 M EDTA, pH 8	Sigma Aldrich
Sodium Chloride solution 5 M	Invitrogen
Ultra Pure TrisHCl 1 M pH 8	Invitrogen
Qubit DNA HS	Thermo Fisher
Illumina compatible barcodes	IDT (see Table S16 for barcode sequences)

Table S15 (Excel file): Patient table for IEI patients used in the study

Table S16 (Excel file): CHOPOFF off-target predictions for selected gRNAs

Table S17 (Excel file): Barcodes used for scRNA-seq

Table S18 (Excel file): scRNA-seq fusion detection

Table S19 (Excel file): Mass spectrometry comparing all samples to unedited healthy controls

Table S20 (Excel file): Mass spectrometry comparing all samples to unedited DADA2 patients

Table S21 (Excel file): Mass spectrometry hits in DADA2 patients

SUPPLEMENTAL METHODS

Patient and healthy donor sample collection

We obtained peripheral blood, cord blood, and fibroblasts from human donors. The study was conducted per the principles of the Helsinki Declaration. It was approved by the Helsinki University Central Hospital Ethics Committee, and the Regional Committee for Medical and Health Research Ethics South-East Norway. The donors participating in the study have signed written informed consent. Information about patients used in the study is presented in Supplemental Table S15 (separate Excel file).

Isolation and culture of human primary T cells, CD34+ HSPCs and fibroblasts

To isolate human T cells, peripheral blood mononuclear cells (PBMCs) were isolated from human peripheral blood from healthy donors (HD) and patients using Ficoll (StemCell Technologies) gradient centrifugation. Isolated PBMCs were cryopreserved at -150°C. For experiments, PBMCs were thawed and cultured at 1 million cells/mL in ImmunoCult™-XF T Cell Expansion Medium (StemCell Technologies). The basal media was supplemented with 120 U/mL IL-2 (Peprotech), 3 ng/µL IL-7 (Peprotech), 3 ng/µL IL-15 (Peprotech) and 15 µL/mL ImmunoCult™ Human CD3/CD28 T Cell Activator (StemCell Technologies) to make T cell stimulation medium. After incubating cells at 37°C/5% CO_2 for three nights, cells were either nucleofected or diluted further with T cell stimulation medium without CD3/CD28 T Cell Activator.

CD34+ hematopoietic stem and progenitor cells (HSPCs) were isolated from cord blood collected during scheduled caesarean sections. Cells were isolated using CD34 MicroBead Kit UltraPure (Miltenyi), and cryopreserved at -150°C. For experiments, CD34+ HSPCs were thawed and cultured at 0.3 million cells/mL in StemSpan™ SFEM II (StemCell Technologies), supplemented with 1X GlutaMax (ThermoScientific), 100 ng/mL human recombinant Flt3-L (Peprotech), 20 ng/mL human recombinant TPO (Peprotech), 100 ng/mL human recombinant SCF (Peprotech), 20 ng/mL human recombinant IL-6 (Peprotech), 10 µM StemRegenin-1 (StemCell Technologies) and 50 µM UM729 (StemCell Technologies) to make HSPC stimulation medium. After incubating cells at 37°C/5% CO_2 for three nights, cells were either nucleofected or diluted further with HSPC stimulation medium.

Human fibroblasts were isolated from skin biopsies, expanded in DMEM medium supplemented with low glucose, 1 mM Puryvate and 10% FBS (Gibco) and cryopreserved at -150°C. For experiments, cells were thawed and cultured in same conditions until confluent.

Cells were passaged every 3-4 days by dissociating them with TrypLE™ Express Enzyme (Gibco), until cell expansion was complete and cells were nucleofected. All gene editing experiments with fibroblasts were carried out latest at passage 10.

CRISPR gRNA design for *ADA2, AIRE, RMRP* and *STAT1*

We designed 3-18 gRNAs per locus for *ADA2*, *AIRE*, *RMRP* and *STAT1* based on available PAM (NGG) sites within the 100 bp repair template region centering the mutation site. gRNA sequence information is presented in Table S2, where gRNAs overlapping the mutation sites are marked in red.

CRISPR repair template design for *ADA2, AIRE, RMRP* and *STAT1*

Single-stranded DNA repair templates (ssODN) of 100 bp length were designed for *ADA2*, *AIRE*, *RMRP* and *STAT1* with +/- 50 bp homology arms surrounding the mutation site. To prevent Cas9 from re-cutting the edited strand, silent SNPs were added in *ADA2*, *AIRE* and *STAT1* designs in addition to mutation correction (WT/MUT→SNP). Four silent SNPs were added for *ADA2* and three for *AIRE* and *STAT1*. As *RMRP* is non-coding, we used non-silent SNPs (WT→SNP, MUT→SNP) for early experiments and mutation correction (MUT→WT) later for functional assessments. ssODN sequence information is presented in Table S3. To further improve HDR, we designed asymmetric ssODNs for *ADA2*, *AIRE*, *RMRP* and *STAT1*, where we tested 10-40 nt homology arms surrounding the mutation sites. Sequences of asymmetric ssODNs and SNP strategy are presented in Table S3.

Nucleofection of human primary T cells, CD34+ HSPCs and fibroblasts

Human T cells, CD34+ HSPCs and fibroblasts were nucleofected using 4-D Nucleofector system and 96-well unit (Lonza). gRNAs were made by annealing crRNA (IDT) and tracrRNA (IDT) as according to the manufacturer's instructions. RNPs were prepared by mixing 61 pmol Alt-R™ S.p. Cas9 Nuclease V3 (IDT) with 100 pmol annealed gRNA per sample and incubating at 37°C for 15 min, after which 100 pmol ssODN (IDT) was added.

For nucleofection, 0.5 or 1 million T cells, 0.3 million HSPCs and 1 million fibroblasts per sample were resuspended in 20 uL Primary P3 electroporation buffer (Lonza) and mixed carefully with the RNPs, making the final concentrations of Cas9 nuclease at 3.05-, gRNA at 5- and ssODN at 5 μmol/L per nucleofected sample. Cells were nucleofected with the following programs: EO-115 (T cells), DZ-100 (HSPCs), CA-137 (fibroblasts).

After nucleofection of T cells, 85 µL T cell recovery medium (basal medium supplemented with 250U/mL IL-2) was added into the electroporation plate, followed by 15 min incubation at 37°C/5% CO_2. Afterwards, cells were transferred into 24-well (1 million cells/sample) or 48-well (0.5 million cells/sample) plates to grow and incubated at 37°C/5% CO_2. Cells were split 1:1 or as necessary with T cell recovery medium 24h and 72h after nucleofection. Cells were collected for downstream analyses 4 days, or alternatively 6-8 days, after nucleofection depending on the experiment.

After nucleofection of HSPCs, 85 µL HSPC stimulation medium (basal medium supplemented with aforementioned cytokines) was added into the electroporation plate, followed by 15 min incubation at 37°C/5% CO_2. Afterwards, cells were transferred into 48-well culture plates to grow and incubated at 37°C/5% CO_2. HSPC stimulation medium was added 24 and 72h after nucleofection if necessary. Cells were collected for downstream analyses 4 days after nucleofection.

After nucleofection of fibroblasts, 85 µL fibroblast culture medium (basal medium supplemented with aforementioned reagents) was added into the electroporation plate, followed by 15 min incubation at 37°C/5% CO_2. Afterwards, cells were transferred into 6-well culture plates to grow and incubated at 37°C/5% CO_2. Medium was changed 24h after nucleofection, and samples were trypsinized and collected for downstream analyses 4 days after nucleofection.

Flow cytometry

1. Characterization of immune cells in HD PBMCs

PBMC samples from day 1, 4 and 8 of the platform were prepared for flow cytometry analysis by washing 0.5 million cells per sample once with RT PBS, followed by blocking for 10 min at RT with 10% human serum in PBS. Cells were then stained in the dark for 30 min at 4°C by adding 50 µL antibody cocktail per sample, as presented in Table S4. After staining, cells were washed twice with 200 µL flow buffer (eBioscience) and resuspended in 250 µL flow buffer. Samples were stored in the dark at 4°C until flow cytometry. The flow analyses were performed on LSRII (BD Bioscience) at the Flow Cytometry Core Facility at Oslo University Hospital (Oslo, Norway). Data analysis was done with FlowJo software (FlowJo LLC, Ashland, OR).

2. CD4+ CD8+ T cell sorting panel for fluorescence-activated cell sorting

T cells from DADA2 patient and HD were collected on day 8 of the platform and prepared for flow cytometry analysis by collecting 2 million cells per sample and washing them once with ice-cold PBS. Cells were resuspended with 200 µL of 1:500 Live/Dead dye combined 1:10 FcR Blocking Reagent (Miltenyi) and samples were stained in the dark at 4°C for 30 min. Cells were then stained in the dark for 30 min at 4°C by adding 50 µL antibody cocktail per sample, as presented in Table S5. After staining, cells were washed once in cold flow buffer (eBioscience) and resuspended in cold flow buffer, followed by FACS (SONY SH800S) at Centre for Molecular Medicine Norway at University of Oslo, Norway. Data analysis was done with FlowJo software (FlowJo LLC, Ashland, OR).

3. T cell proliferation assay in CHH patients

CHH patient T cells from day 20 of the platform were collected and washed once with PBS. Cells were resuspended in PBS at 2 million cells/mL. CFSE working solution (2 µM) was prepared right before staining from CellTrace™ CFSE Cell Proliferation Kit (Invitrogen), where stock (5mM) was first diluted with PBS. To stain cells, equal volume of CFSE working solution and PBS were added to get a final concentration of 1 uM CFSE. Cells were immediately vortexed for 10 s, followed by incubation in the dark at 37°C, 5% CO2 for 5 min, including a brief vortexing step at 2.5 min of incubation. Immediately after incubation, equal volume of cold human serum (Sigma) was added on cells. Cells were centrifuged, washed twice with PBS and resuspended in Immunocult medium at 4 million cells/mL. Cell suspension (50 uL) was added per well on a 96-well U bottom plate (Thermo Fisher) containing 100 ul of Immunocult medium and IL2 to get 0.2 million cells per well with a final concentration of 250 U/mL IL-2 (Peprotech). The cells were then incubated for four days at 37°C, 5% CO_2. On day 24 of the platform, cells were stained for flow cytometry. Samples were washed once with PBS and resuspended in 50 uL Live/Dead staining with Fc blocking reagent (Miltenyi) per sample and incubated in the dark for 30 min at 4°C. Samples were centrifuged and resuspended in 50 uL antibody cocktail as presented in Table S6 and stained in the dark for 30 min at 4°C. Afterwards, cells were washed two times with flow buffer (eBioscience) and resuspended in flow buffer for flow cytometry analysis. The flow analyses were performed on LSRII (BD Bioscience) at the Flow Cytometry Core Facility at Oslo University Hospital (Oslo, Norway). Data analysis was done with FlowJo software (FlowJo LLC, Ashland, OR).

4. Assessment of phosphorylated STAT1 in STAT1-GOF patients

Patient T cells were harvested four days post electroporation. Cell suspension was washed twice in PBS by centrifuging at 400 g for 6 min and discarding the supernatant. One million cells per sample were used for each condition. Cells were resuspended with 100 µL of 1:500 Live/Dead dye combined 1:10 FcR Blocking Reagent (Miltenyi) and stained in the dark at 4°C for 30 min. After incubation, 5 mL ImmunoCult was added to the cells before centrifugation at 400 g for 6 min. The supernatant was discarded after centrifugation, and 250 µL of the 2 x10^3 U/ml of the IFNα in medium and the cocktail of cell surface antibodies (presented in Table S7) was added to the samples to be stimulated. The unstimulated controls received only 250 µL medium and the same antibody cocktail. The cells were incubated at 37 ºC for 30 min in the dark while being shaken every five minutes during incubation. Immediately after, 2 mL of freshly prepared 1:5 Phosflow Lyse/Fix Buffer was added to the sample tubes, which was then shortly vortexed before incubating in a 5 % CO2 incubator at 37 ºC for 10 min. The tubes were shaken every 3 minutes to ensure thorough cell lysis and fixation. After incubation, the tubes were centrifuged at 500 g for 8 min. The supernatant was discarded, and the cell pellet was shortly vortexed. 5 mL flow buffer was added to each tube and centrifuged at 500 g for 8 min. The supernatant was discarded, and the cell pellet was shortly vortexed. 500 µL of cold Phosflow PermBuffer III was added to each tube. The tubes were incubated on ice for 30 min in the dark. After incubation, 5 mL of eBioscience™ Flow Cytometry Staining Buffer was added to each tube, followed by centrifugation at 500 g for 8 min. Supernatant was discarded and cells were stained with 1:10 dilution of pSTAT1 antibody in a total staining volume of 50 µl. Cells were mixed and incubated for 30 min at RT in the dark. Afterwards, cells were washed two times with flow buffer, resuspended in flow buffer and stored in refrigerator overnight for flow cytometry analysis the day after.

Assessment of *in silico* gRNA design tools

To evaluate predictive power of available *in silico* gRNA design tools against *in vitro* gRNA screening data, we selected the following tools: Atum (https://www.atum.bio/eCommerce/cas9/input), Benchling (benchling.com), CHOPCHOP (chopchop.cbu.uib.no), CRISPOR (crispor.tefor.net/), DeepSpCas9 (deepcrispr.info/DeepSpCas9/), EuPaGDT (grna.ctegd.uga.edu/) and IDT gRNA design tool (eu.idtdna.com/site/order/designtool/index/CRISPR_SEQUENCE). We used 100 bp mutant-specific sequences with 50 bp homology arms from the mutation site as input (target sequence) for the tools. Three gRNAs with highest predicted efficiency were chosen from the tool output and compared against three best *in vitro* validated gRNAs from patient T cells.

On-target editing assessment by ddPCR

ddPCR assays were performed to assess HDR and NHEJ editing for *ADA2*, *AIRE*, *Enh4-1*, *CTCF1*, *RNF2*, *RMRP* and *STAT1*. We used previously published ddPCR oligos for Enh4-1, CTCF1, RNF2[35] and designed new ddPCR oligos for *ADA2*, *AIRE*, *RMRP* and *STAT1* (presented in Table S8). ddPCR was performed using the QX200 system (Bio-Rad) as previously described[35]. In short, 8 µl of DNA (concentration normalized to 8 ng/µl), primers (900 nM), reference probe (250 nM), and HDR or NHEJ probe (250 nM). The HDR and NHEJ detection occurred in two separate ddPCR reactions. Each reaction was then loaded into a sample well of an eight-well disposable cartridge (DG8; Bio-Rad Laboratories) along with 70 µl of droplet generation oil (Bio-Rad Laboratories). Droplets were formed using a QX200 Droplet Generator (Bio-Rad Laboratories). Droplets were transferred to a 96-well PCR plate, heat-sealed with foil, and amplified using a conventional thermal cycler. The thermocycling protocol was the following: (1) 95°C - 10 min, (2) 94°C – 30 s, 56°C – 3 min, step repeated 42 times (3) 98°C – 10 min, (4) 4°C – hold. The resulting PCR products were loaded on a QX200 Droplet Reader (Bio-Rad Laboratories), and the data was analyzed using QuantaSoft software (Bio-Rad Laboratories).

On-target editing assessment by amplicon sequencing

Amplicon sequencing libraries for assessing on-target editing for *ADA2*, *AIRE* and *RMRP* were prepared from gDNA samples as previously described.[35] In short, library preparation was performed using a two-step PCR method. For the first PCR, a pair of target-specific primers were designed to amplify a 150 bp area surrounding the cutting site. Each target primer additionally includes an extension at the 5' end: for forward primers, this contains the Illumina Read1 primer sequence (see below, nucleotides in bold) and an 8 bp UMI (nucleotides underlined), and for the reverse primers, this contains the Illumina Read2 primer sequence only (see below, nucleotides in bold):

ADA2 fwd 5'→3': **ACACTCTTTCCCTACACGACGCTCTTCCGATCT**NNNNNNNNTCATGCAGTTCAGATTTGCTCAC
ADA2 rev 5'→3': **GTGACTGGAGTTCAGACGTGTGCTCTTCCGATCT**GGCCTGGGACATGTGCTTTC

AIRE fwd 5'→3': **ACACTCTTTCCCTACACGACGCTCTTCCGATCT**NNNNNNNNactcccagcaagttcgaaga
AIRE rev 5'→3': **GTGACTGGAGTTCAGACGTGTGCTCTTCCGATCT**GGGGGCATCAAGAGCCAG

RMRP fwd 5'→3': **ACACTCTTTCCCTACACGACGCTCTTCCGATCT**NNNNNNNNgagtgggaagcggggaatg
RMRP rev 5'→3': **GTGACTGGAGTTCAGACGTGTGCTCTTCCGATCT**AGCTGAGGACGTGGTTCGT

First PCR was performed with reagents listed presented in Table S9. The thermocycling protocol was the following: (1) 98°C - 30 s, (2) 98°C – 10 s, 57°C (ADA2, RMRP)/58°C (AIRE) – 10 s, 72°C – 20 s, step repeated 30 times (3) 72°C – 5 min, (4) 4°C – hold.

For the second PCR, the amplified products were purified using AMPure XP magnetic (Beckman Coulter, #A63882) according to the manufacturer's instructions, pooled and annealed with i5 and i7 Illumina Index primers (presented in Table S10). Both primers contain flow-cell-binding region (highlighted in bold), index region (underlined) and Illumina Read1 or Read2 primer binding regions, correspondingly (italics).

Second PCR was performed with reagents listed in Table S10. The thermocycling protocol was the following: (1) 98°C - 30 s, (2) 98°C – 10 s, 58°C – 10 s, 72°C – 20 s, step repeated 10 times (3) 72°C – 5 min, (4) 4°C – hold.

PCR products were purified using AMPure XP magnetic beads (Beckman Coulter) according to the manufacturer's instructions and DNA concentrations were measured with Qubit HS kit (Thermo Fisher Scientific). Final sample libraries were sequenced using Illumine MiSeq v2 Micro flow cell, including 10% PhiX. Data analysis was performed using the ampliCan software package.[36]

Off-target assessment by GUIDE-seq

One million patient and healthy donor T cells/sample were nucleofected on day 5 of the platform, as previously described, with RNPs containing selected gRNAs 100 pmol, Cas9 nuclease at 61 pmol and dsODN at 30 pmol/nucleofected sample. Thus, final concentrations of CRISPR reagents were: Cas9 nuclease at 3.05-, gRNA at 5- and dsODN at 1.5 μmol/L per nucleofected sample.

Cells were transferred into 24w plates after nucleofection with 500 uL T cell recovery medium and split 1:1 with T cell recovery medium 24h and 72h after nucleofection. Samples were collected for GUIDE-seq sample processing and ddPCR 4 days after nucleofection.

The blunt-ended dsODN used in our GUIDE-seq experiments was the same as that was used in the original publication.[37] dsODN was prepared by annealing the two modified oligonucleotides of the following compositions:

5′- P-G*T**TTAATTGAGTTGTCATATGTTAATAACGGT**A*T -3′ and
5′- P-A*T**ACCGTTATTAACATATGACAACTCAATTAA**A*C -3′
P represents a 5′ phosphorylation and * indicates a phosphorothioate linkage.

The GUIDEseq protocol[37] was adapted from certain modifications described below. Briefly, gDNA was sheared with a Bioruptor® Pico Sonication System (Diagenode) to an average length of 500 bp. End-repair was done with Fast DNA End Repair Kit (Thermo Fisher Scientific), A-tailing with Taq DNA Polymerase, native (Thermo Fisher Scientific) and ligation of half-functional adapters, incorporating 8-nt random molecular index was done using T4 DNA Ligase (Thermo Fisher Scientific), all according to the manufacturer's instructions. Between each step, DNA was purified using AMPure XP SPRI beads (Backman Coulter) and eluted with TE buffer, pH 8.0 (Invitrogen). Two rounds of nested anchored PCR, with primers complementary to the oligo tag, were used for target enrichment. Before library pooling, the quality of the final products was tested using High Sensitivity DNA Kit (Agilent) on Bioanalyzer 2100 (Agilent) according to the manufacturer instructions and NanoDrop (Thermo Fisher Scientific). Based on the average size estimated from Bioanalyzer, equal number of particles from each sample were pulled to achieve the final volume 20µl containing $1{,}2 \times 10^{10}$.

Sample was delivered together with custom sequencing primer Index-1 and Read-2 and sequenced at The Department of Core Facilities, Oslo University Hospital, Norway. Denaturated library was loaded onto the Miseq according to Illumina's standard protocol for sequencing with an Illumina Miseq Reagent Kit V2 - 300 cycle (2 x 150 bp paired end).

Data analysis was performed following the GUIDE-Seq analysis pipeline[38] but adjusted for allowing bulges between sgRNA and off-target sites with editing distance of 4. We used custom scripts (https://git.app.uib.no/valenlab/t_cell_editing_pipeline/) with cutadapt v2.8 (TTGAGTTGTCATATGTTAATAACGGTAT and ACATATGACAACTCAATTAAAC). Afterward, the data was aligned to the human genome (hg38v34) using bwa v0.7.17-r1188. CHOPOFF (https://github.com/JokingHero/CHOPOFF.jl) was used to find all off-target sites with edit distance up to 4, allowing for mismatches, deletions and insertions. Final off-targets were normalized against control data (transfected with dsODN only). Control data was processed in the same pipeline as the modified Cas9 samples. Detailed analysis of predicted *in silico* off-targets can be found in Supplemental Table S16 (separate Excel file).

Cas9WT and Cas9-SNAP *in vitro* mRNA transcription

Cas9WT and Cas9-SNAP mRNA were prepared with HiScribe T7 ARCA mRNA Kit with tailing (NEB-Bionordika), according to the manufacturer's instructions. Total of 8000 ng stock plasmid was digested with 2 µl FastDigest MssI enzyme (Thermo Fisher Scientific) in the supplemented restriction-digestion buffer with a total reaction volume of 20 µl. Incubation was carried out at 37°C overnight. Length of the digested product was confirmed by gel electrophoresis. For the IVT reaction, 1000 ng of the linearized plasmid was mixed with 10 µl of 2xARCA/NTP mix and 2 µl of T7 RNA Polymerase mix, and the reaction was incubated for 30 min at 37°C. Sequentially, 2 µl of DNAse enzyme was added and the mixture was incubated at 37°C for 15 min. Poly(A) tailing step was performed by adding 20 µl of milliQ (RNAse free), 5 µl of 10× PolyA polymerase reaction buffer, and 5 µl of 10× PolyA polymerase directly to the IVT reaction, which was incubation at 37°C for 30 min. mRNA was purified using LiCl solution, as described in the manufacturer's protocol. Aliquots were frozen in -80°C for later use.

Synthesis of O^6-Benzylguanine coupled repair templates

BG-coupled repair template oligos for ADA2 and AIRE were prepared as previously described.[39,40] In short, a coupling reaction of BG-GLA-NHS (New England BioLabs) and NH2-oligo (IDT) in HEPES buffer pH 8.5 (Invitrogen) was performed. Following coupling reactions, the BG-oligos were purified with ethanol precipitation as and stored at -20°C until later use.

Cas9-SNAP nuclease production

To test the editing performance of BG-coupled repair oligo in combination with the Cas9-SNAP fusion protein, the Cas9-SNAP protein was produced as protein. The pTH24-Cas9-SNAP construct was transformed into *E. coli* BL21(DE3) T1R cells and cultivated in Terrific Broth (TB) medium. Protein expression was induced with isopropyl-D-1-thiogalactopyranoside, and protein purified by immobilized metal-ion chromatography, followed by size exclusion chromatography (SEC). The purified protein was stored in 20 mM HEPES supplemented with 300 mM NaCl, 10% glycerol and 2 mM TCEP to pH 7.5. Aliquots were flash-frozen in liquid nitrogen and stored at -80°C until experiments.

HDR enhancing compound screen in healthy donor T cells

We selected 33 previously published HDR enhancing compounds for the screen, described in Table S12. Compounds were dissolved in DMSO and each of them were assessed at three concentrations in HD T cells against DMSO vehicle control. As described previously, 0.5

million T cells/sample were nucleofected, transferred to 48-well cell culture plates and incubated in T cell recovery medium containing the compounds for 24h. Cells were split 1:1 24h and 72h after nucleofection in T cell recovery medium without the compounds. Samples were collected for gDNA extraction and ddPCR 96h after nucleofection.

Validating cell cycle inhibitors in healthy donor T cells

We selected 10 previously published HDR enhancing cell cycle inhibitors for validating in HD T cells and assessed at three concentrations against DMSO vehicle control, described in Table S13. Cells were either pre-treated with the compounds or vehicle for 24h before nucleofection, followed by nucleofection and incubation without compounds, or treated for 24h after nucleofection. For both conditions, 0.5 million cells per sample were nucleofected. For both groups, cells were split 1:1 in T cell recovery medium without compounds 24h and 72h after nucleofection. Samples were collected for gDNA extraction and ddPCR 96h after nucleofection.

PacBio sequencing and variant calling of CRISPR edited healthy donor T cells

T cells from a healthy donor with written consent for sequencing were cultured and edited as previously described. The cells were either unedited and treated with 0.5 μM KU0060648 or DMSO or *ADA2*-edited and treated with 0.5 μM KU0060648 or DMSO. Cells were collected six days after editing on day 10 of the platform and DNA was extracted from 5 million cells per sample using Blood & Cell Culture DNA Kits (Qiagen). All samples were extracted according to the manufacturer's instructions for Cell cultures described in "QIAGEN® Genomic DNA Handbook, June 2015". The concentration, purity and size of the DNA was estimated using both NanoDrop (Thermo Fisher Scientific) and a Qubit fluorometer (Invitrogen) and checked on the agarose gel (0.5%, 35V, 16-18 hours runtime) containing 500 ng of each sample, with an appropriate ladder as a reference standard: Quick-Load 1 kb Extend DNA Ladder (New England Biolabs).

Library preparations for PacBio HiFi sequencing were done by the Norwegian sequencing Centre on 8M SMRT cells using Revio HiFi prep kit and Sequencing chemistry v2.0. The sequencing data was demultiplexed with the Demultiplexing pipeline on SMRT Link v10.2.0.1333434. Circular consensus sequencing (CCS) reads were then generated for demultiplexed polymerase reads and further demultiplexed using the barcoded primer sequences. The HiFi sequencing reads were separated and indexed with the provided barcode ID.

The HiFi sequencing reads were aligned with pbmm2 v1.13.0 with options "--preset HIFI --bam-index BAI --sort". Structural variants were called with pbsv v2.9.0, small variants with deepVariant v1.6.0. All possible mismatches, deletions and insertions were extracted from aligned reads using custom scripts (https://git.app.uib.no/valenlab/t_cell_editing_pipeline/-/tree/main/katariina_pacbio). We normalized data using two control samples and focused on sites that were potential sgRNA off-target within distance of 4, allowing for bulges. Additionally, transversion ratio plot and codon signature analysis showed no global effects of CRISPR activity.

CellTiter-Glo cell viability assay for HDR enhancing compound toxicity assessment

HDR enhancing compound toxicity was assessed by CellTiter-Glo viability assay (Promega) according to manufacturer's instructions. In short, 50 µL of T cell suspension per sample was transferred into while opaque 96-w plates (Thermo Fisher), followed by adding 100 µL RT CellTiter-Glo assay buffer per sample. Plate was covered with aluminum foil and placed on a plate shaker at 500 rpm for 5 min. Afterwards, plate was incubated for 10 min at RT while still covered. After incubation, foil was removed and luminescence values from the plate were assessed by BioTek Synergy Neo2 Instrument (Agilent). To analyze results, background values from medium alone were subtracted from sample values and data was analyzed according to manufacturer's instructions.

scRNAseq in HD and DADA2 patient T cells

1. Cell culture and processing

Cells were cultured and nucleofected as previously described and sorted with FACS on day 8 of the platform into 384 well plates containing 2 uL of lysis buffer [H_2O: 1.31 uL, RNase Inhibitor 0.05 uL, ERCC (1:30000) 0.05 uL, 10% Triton (0.04 uL), 10 mM dNTP (0.5 uL) and 100 uM oligo dT (0.05 uL)]. After sorting, the plates were spun down at 2000g, 4°C for 5 min and then the plate was snap frozen on dry ice and kept at -80 until further processing. Reagents for sample processing are described in Table S14.

Oligos:

Oligo-dT:	IDT	AAGCAGTGGTATCAACGCAGAGTACTTT<br>TTTTTTTTTTTTTTTTTTTTTTTTTTT<br>(N1:34333300)(N2:25252525)
IS_PCR	IDT	5′-AAGCAGTGGTATCAACGCAGAGT-3′

TSO	IDT	5′-AAGCAGTGGTATCAACGCA GAGTACATrGrG+G-3′
ME-A	IDT	5′-TCGTCGGCAGCGTCAGATGTG TATAAGAGACAG-3′
ME-B	IDT	5′-GTCTCGTGGGCTCGGAGATG TGTATAAGAGACAG-3′
ME-Rev	IDT	5′-/5Phos/CTGTCTCTTATACACATCT-3′

ADA2_WT IDT /56-FAM/TGGAGGATT/ZEN/ATCGGAAGCGGGTG/3IABkFQ/

ADA2_Mut/WT_Fixed IDT /5HEX/TGGAGGACT/ZEN/ACAGAAAGCGGGTG/3IABkFQ/

ADA2 fwd: GGTGAGGAATGTCACCTACA

ADA2 rev: CATCAAACTCAGTGACGTTC

2. RNA preparation

Full length mRNA-sequencing is based on Smart-Seq2 protocol.[41,42] Lysis plates containing cells were thawed and primer annealing was performed for 3 min at 72°C. 3 uL of reverse transcription mix (5x Reverse Transcriptase buffer (1 uL), Maxima H minus Reverse Transcriptase (0.05 uL), RNase Inhibitor (0.125uL), 100mM DTT (0.25 uL), 5M Betaine (1 uL), 1M $MgCl_2$ (0.03 uL), 100uM TSO (0.05 uL), H_2O 0.495) was added to each well, and reaction occurred at 42°C for 90 min, then heat inactivation at 70°C for 5 min. Next cDNA pre-amplification was performed by adding 7 uL of mastermix [2X Kapa HiFi HotStart ReadyMix (6 uL), 10 uM IS_PCR primer (0.12 uL), Lambda exonuclease (0.05625 uL) and H_2O (0.8237 uL)]. PCR program was 37°C for 30 min, 95°C for 3 min, 22 cycles of 98°C for 20s, 67°C for 15s, 72°C for 4 min, then final elongation at 72°C for 5 min.

At this stage primers are removed by SPRI bead cleanup (prepared as here https://openwetware.org/wiki/SPRI_bead_mix#Ingredients_for_50_mL_2) at a ratio of 0.7:1. Concentration of independent wells is measured with Qubit DNA HS kit, and wells are diluted to 0.15 ng/uL.

3. Library preparation

Tagmentation was performed on the diluted cDNA, by adding 1 uL cDNA to 1.5 uL tagmentation mix (Tn5 (2.6mg/mL purified psfTn5-c006, Addgene plasmid #79107, loaded with standard Illumina Tn5 adapters (Meds A, MedsB, MedsRev))) (0.250 uL), 5X TAPS-PEG (Buffer is is 8% PEG, 5mM MgCl2, 10mM TAPS) 0.5 uL), H2O (0.750 uL)) and incubate for

10 min at 55°C. Then the reaction was stopped and transposome stripped of cDNA by adding 0.1% SDS (1 uL) and incubating for 10 min at 55°C. 7 uL barcoding mix was added (5x buffer (2.5 uL), 10mM dNTP (0.3 uL), 10% Tween (0.15 uL), Kapa HiFi (0.2 uL), H2O (3.85 uL) and 2 uL primer mix at 3.75 uM/primer. PCR program was as follows: 72°C for 3 min, 95°C for 30s, then 12 cycles of 95°C for 15s, 55°C for 30s, 72°C 45s, then final elongation at 72°C for 5 min.

Library was pooled and cleaned up 2x with 0.9:1 ratio of SPRI beads.

Libraries were sequenced on a Novaseq 6000.

4. RT-qPCR

For quantitative analysis of the two different alleles, 1uL of the diluted cDNA is amplified with Ada2 specific primers in the presence of WT or mutated and edited probes that are attached to different fluorophores. Reaction conditions were as follows: 2X Kapa HiFi HotStart ReadyMix (2.5 uL), 10uM forward and reverse primers (0.05 + 0.05 uL), 10 uM WT probe (0.05 uL), 10 uM Edited probe (0.05 uL), H2O (1.3 uL). PCR program was 95°C for 1 min, 35 cycles of 95°C for 15 s, 63°C for 45 s.

5. Analysis of scRNA-seq data

Cutadapt[43] was used to trim RNA sequence reads from adapters and low-quality bases. STAR[44] was used to align to hg38, with ERCC reads added. Picard[45] was used to remove duplicate reads. HTSeq[46] was used to summarize read counts. Cells with less than 20000 reads or 500 features were filtered out, as well as those with ACTB expression less than 0.01 quantile of the normal distribution. Seurat[47] was used to process the count data. Shortly, data was log normalized, 2000 variable features were found and the data were scaled; this was done separately per condition. Pathway analysis on scRNA-data was done as follows. FindMarkers function of Seurat was used to find markers between the two conditions of interest, with logfc.threshold=0. The resulting table was arranged based on avg_log2FC. The fgsea[48] package was used to read in the C7 and hallmark pathways from the Molecular Signature Database /Subramanian, Tamayo, et al. (2005, PNAS) and one or more of the following as appropriate: Liberzon, et al. (2011, Bioinformatics) and to perform gene set enrichment analysis based on the ordered avg_log2FC from FindMarkers and minSize=15 and maxSize=500. Information about barcodes used for sequencing can be found in Supplemental Table S17 (separate Excel file).

STAR-Fusion[49] was used to predict fusion transcripts from the single cell data. Pseudobulk samples were prepared by merging fastq files per condition (unedited, edited +/- NHEJ inhibition, per individual), and run with default parameters. The deconvolved abridged fusion predictions were filtered by removing all fusions that were categorised as neighbouring by STAR-Fusion annotation. Information about fusion transcripts can be found in Supplemental Table S18 (separate Excel file).

To detect possible chromosomal loss due to editing all bam files from two individuals, a patient and a healthy donor were merged to create pseudobulk bam files using samtools.[50] cellsnp-lite[51] was used to genotype the samples, based on the 1000G phase 1 SNPs (https://www.internationalgenome.org/category/variants/), with settings minMAPQ=20, minLEN=30, UMItag=None, p=20, -I P, countORPHAN, exclFLAG=UNMAP, SECONDARY, QCFAIL, DUP. The output was filtered for GT="het" & INFO/AD[0]>1 using bcftools.[52] The vcf files were uploaded to Michigan Imputation Server,[53] where the 1000G phase3 30x panel was chosen as a reference for imputation and Eagle2.4 was chosen for phasing. The imputed files were filtered with bcftools for R2>0.3 and TYPE="snp. Then cellsnp-lite was run on all individual cells against the imputed file for each sample, with the same parameters as before, except minCOUNT=10 and minMAF=0.2. The haplotype ratios were calculated and cells with only one haplotype on the q arm of chr22 were considered to have lost one chromosome.

6. Analysis of RT-qPCR data

RT-qPCR analysis was performed on the RFU values per allele. A cutoff of an RFU value 200 was determined to decide which allele (wild type, mutated or edited) was being expressed in each cell.

7. TCR Repertoire analysis

TCR reconstruction was performed using TRACER (v0.6.0)[54] with settings --loci A B G D -p 16 -s Hsap on individual fastq files. Only cells harboring productive A and B locus were used for clonotype identification.

Mass spectrometry

1. Sample preparation

T cells from three DADA2 patients and healthy donors were cultured as previously described and nucleofected on day 5 of the platform, where 1M cells/sample were mock nucleofected or

ADA2-edited. Mock edited cells were treated with DMSO and edited cells with 0.5µM KU0060648, 0.6µM IDT Alt-R enhancer V2 or DMSO for the first 24h after nucleofection. Cells were collected seven days after editing on day 12 of the platform, washed 2X with ice-cold PBS, pelleted and snap-frozen in liquid nitrogen. Pellets were stored at -80°C until mass spectrometry sample preparation.

The samples were lysed in 8M Urea (#U5378-500G, Sigma Aldrich) in 100 mM ammonium bicarbonate (NH4HCO3 containing benzonase nuclease (415 units/ml, sc-202391, Santa Cruz Biotechnology). Total protein concentration was measured with Bio-Rad Protein Assay Dye (#5000006, Bio-Rad Laboratories). 50 µg of total protein was taken from each sample for reduction (5 mM dithiothreitol (#D9779, Sigma-Aldrich), alkylation (15 mM iodoacetamide (#122271000, Acros Organics), and overnight digestion with 2µg Trypsin/Lys-c Mix (V507A, Promega) at 37°C. After digestion, samples were acidified with 10% trifluoroacetic acid (TFA, #85049.051, VWR) and desalted with BioPureSPN PROTO 300 C18 Mini columns (#HUM S18V, Nest Group) according to manufacturer's instructions. After desalting the samples were dried in a centrifuge concentrator (Concentrator Plus, Eppendorf). The dried peptides were reconstituted in 40 µl buffer A (0.1% (vol/vol) TFA, 1% (vol/vol) acetonitrile (#83640.320, VWR) in HPLC grade water (#10505904, Fisher Scientific)).

For the DIA analysis the resuspended peptides were further diluted 1:60 in buffer A1 (1% formic acid in HPLC water). 20 µl was loaded into an Evotip (Evosep, Denmark) following manufacturer's instructions.

2. Mass spectrometry and analysis

The desalted samples were analyzed using the Evosep One liquid chromatography system coupled to a hybrid trapped ion mobility quadrupole TOF mass spectrometer (Bruker timsTOF Pro, Bruker Daltonics) (Meier, Brunner et al., 2018) via a CaptiveSpray nano-electrospray ion source (Bruker Daltonics). An 8 cm × 150 µm column with 1.5 µm C18 beads (EV1109, Evosep) was used for peptide separation with the 60 samples per day methods (21 min gradient time). Mobile phases A and B were 0.1% formic acid in water and 0.1% formic acid in acetonitrile, respectively. The MS analysis was performed in the positive-ion mode with dia-PASEF method[55] with sample optimized data independent analysis (dia) scan parameters. We performed DDA in PASEF mode from a pooled sample to be able to adjust dia-PASEF parameters optimally to these specific samples. To perform sample specific dia-PASEF parameter adjustment the default dia-short-gradient acquisition methods was adjusted based on

the sample specific DDA-PASEF run with the software "tims Control" (Bruker Daltonics). The following parameters were modified for each sample type: m/z range; 429.2 – 1204.2, mass steps per cycle; 31 mean cycle time; 1.48 s. The ion mobility windows were set to best match the ion cloud density from the sample type specific DDA-runs.

To analyze diaPASEF data, the raw data (.d) were processed with DIA-NN v1.8.1[56,57] utilizing spectral library generated from the UniProt human proteome. During library generation following settings were used, fixed modifications: carbamidomethyl (C); variable modifications: acetyl (protein N-term), oxidation (M); enzyme:Trypsin/P; maximum missed cleavages:1; mass accuracy fixed to 1.5e-05 (MS2) and 1.5e-05 (MS1); Fragment m/z set to 100-1700; peptide length set to 7-30; precursor m/z set to 300-1600; Precursor changes set to 2-4; protein inference not performed. All other settings were left to default. Information about mass spectrometry data in healthy controls and patients can be found in Supplemental Tables S19-21 (separate Excel files).

3. Statistical analysis of the proteomics data

The input file to further DIA data analysis was the DIA-NN Report.pg_matrix. For data pre-processing an in-house R-script was utilized. Raw intensity values were log2 transformed and median-normalized. Afterwards, missing values were imputed using QRILC imputation.[58] For sample group comparison, p-values were calculated with student's t-test using python package scipy,[59] and adjusted using benhamini-hockberg method via statsmodels package.[60] Volcano plots were generated with bioinfokit[61] using q-value threshold of 0.01 and log2 intensity fold change thresholds of 1 and -1.

Statistics:

The following softwares were used for data analysis: QuantaSoft (Bio-Rad), FlowJo, Cutadapt 3.2, STAR 2.7.7a, HTseq 0.9.0, Picard 2.22.0, Seurat 5.0.1, FGSEA 1.20.1, STAR-Fusion V1.11.0., cellsnp-lite 1.2.3, bcftools 1.14. All of the statistics in the study were performed using GraphPad Prism 9.

References

1. Fox, T.A., Houghton, B.C., Petersone, L., Waters, E., Edner, N.M., McKenna, A., Preham, O., Hinze, C., Williams, C., de Albuquerque, A.S., et al. (2022). Therapeutic gene editing of T cells to correct CTLA-4 insufficiency. Sci Transl Med *14*, eabn5811. 10.1126/scitranslmed.abn5811.
2. Li, X., Wirtz, T., Weber, T., Lebedin, M., Lowenstein, E.D., Sommermann, T., Zach, A., Yasuda, T., de la Rosa, K., Chu, V.T., et al. (2024). Precise CRISPR-Cas9 gene repair in autologous memory T

cells to treat familial hemophagocytic lymphohistiocytosis. Sci Immunol *9*, eadi0042. 10.1126/sciimmunol.adi0042.

3. Roth, T.L., Puig-Saus, C., Yu, R., Shifrut, E., Carnevale, J., Li, P.J., Hiatt, J., Saco, J., Krystofinski, P., Li, H., et al. (2018). Reprogramming human T cell function and specificity with non-viral genome targeting. Nature *559*, 405-409. 10.1038/s41586-018-0326-5.
4. Goodwin, M., Lee, E., Lakshmanan, U., Shipp, S., Froessl, L., Barzaghi, F., Passerini, L., Narula, M., Sheikali, A., Lee, C.M., et al. (2020). CRISPR-based gene editing enables FOXP3 gene repair in IPEX patient cells. Sci Adv *6*, eaaz0571. 10.1126/sciadv.aaz0571.
5. Vavassori, V., Mercuri, E., Marcovecchio, G.E., Castiello, M.C., Schiroli, G., Albano, L., Margulies, C., Buquicchio, F., Fontana, E., Beretta, S., et al. (2021). Modeling, optimization, and comparable efficacy of T cell and hematopoietic stem cell gene editing for treating hyper-IgM syndrome. EMBO Mol Med *13*, e13545. 10.15252/emmm.202013545.
6. Asperti, C., Canarutto, D., Porcellini, S., Sanvito, F., Cecere, F., Vavassori, V., Ferrari, S., Rovelli, E., Albano, L., Jacob, A., et al. (2023). Scalable GMP-compliant gene correction of CD4+ T cells with IDLV template functionally validated in vitro and in vivo. Mol Ther Methods Clin Dev *30*, 546-557. 10.1016/j.omtm.2023.08.020.
7. Houghton, B.C., Panchal, N., Haas, S.A., Chmielewski, K.O., Hildenbeutel, M., Whittaker, T., Mussolino, C., Cathomen, T., Thrasher, A.J., and Booth, C. (2022). Genome Editing With TALEN, CRISPR-Cas9 and CRISPR-Cas12a in Combination With AAV6 Homology Donor Restores T Cell Function for XLP. Front Genome Ed *4*, 828489. 10.3389/fgeed.2022.828489.
8. Ayoub, P.G., Gensheimer, J., Lathrop, L., Juett, C., Quintos, J., Tam, K., Reid, J., Ma, F., Tam, C., McAuley, G.E., et al. (2024). Lentiviral vectors for precise expression to treat X-linked lymphoproliferative disease. Mol Ther Methods Clin Dev *32*, 101323. 10.1016/j.omtm.2024.101323.
9. Li, X.L., Li, G.H., Fu, J., Fu, Y.W., Zhang, L., Chen, W., Arakaki, C., Zhang, J.P., Wen, W., Zhao, M., et al. (2018). Highly efficient genome editing via CRISPR-Cas9 in human pluripotent stem cells is achieved by transient BCL-XL overexpression. Nucleic Acids Res *46*, 10195-10215. 10.1093/nar/gky804.
10. Riesenberg, S., and Maricic, T. (2018). Targeting repair pathways with small molecules increases precise genome editing in pluripotent stem cells. Nat Commun *9*, 2164. 10.1038/s41467-018-04609-7.
11. Yu, C., Liu, Y., Ma, T., Liu, K., Xu, S., Zhang, Y., Liu, H., La Russa, M., Xie, M., Ding, S., and Qi, L.S. (2015). Small molecules enhance CRISPR genome editing in pluripotent stem cells. Cell Stem Cell *16*, 142-147. 10.1016/j.stem.2015.01.003.
12. Liu, B., Chen, S., Rose, A., Chen, D., Cao, F., Zwinderman, M., Kiemel, D., Aïssi, M., Dekker, F.J., and Haisma, H.J. (2020). Inhibition of histone deacetylase 1 (HDAC1) and HDAC2 enhances CRISPR/Cas9 genome editing. Nucleic Acids Res *48*, 517-532. 10.1093/nar/gkz1136.
13. Stein, E.M., Garcia-Manero, G., Rizzieri, D.A., Tibes, R., Berdeja, J.G., Jongen-Lavrencic, M., Altman, J.K., Dohner, H., Thomson, B., Blakemore, S.J., et al. (2015). A Phase 1 Study of the DOT1L Inhibitor, Pinometostat (EPZ-5676), in Adults with Relapsed or Refractory Leukemia: Safety, Clinical Activity, Exposure and Target Inhibition. Blood *126*, 2547-2547. 10.1182/blood.V126.23.2547.2547.
14. Neal, J.A., Dang, V., Douglas, P., Wold, M.S., Lees-Miller, S.P., and Meek, K. (2011). Inhibition of homologous recombination by DNA-dependent protein kinase requires kinase activity, is titratable, and is modulated by autophosphorylation. Mol Cell Biol *31*, 1719-1733. 10.1128/mcb.01298-10.
15. Munck, J.M., Batey, M.A., Zhao, Y., Jenkins, H., Richardson, C.J., Cano, C., Tavecchio, M., Barbeau, J., Bardos, J., Cornell, L., et al. (2012). Chemosensitization of cancer cells by KU-0060648, a dual inhibitor of DNA-PK and PI-3K. Mol Cancer Ther *11*, 1789-1798. 10.1158/1535-7163.Mct-11-0535.
16. Robert, F., Barbeau, M., Éthier, S., Dostie, J., and Pelletier, J. (2015). Pharmacological inhibition of DNA-PK stimulates Cas9-mediated genome editing. Genome Med *7*, 93. 10.1186/s13073-015-0215-6.
17. Maurissen, T.L., and Woltjen, K. (2020). Synergistic gene editing in human iPS cells via cell cycle and DNA repair modulation. Nat Commun *11*, 2876. 10.1038/s41467-020-16643-5.
18. Hickson, I., Zhao, Y., Richardson, C.J., Green, S.J., Martin, N.M., Orr, A.I., Reaper, P.M., Jackson, S.P., Curtin, N.J., and Smith, G.C. (2004). Identification and characterization of a novel and specific inhibitor of the ataxia-telangiectasia mutated kinase ATM. Cancer Res *64*, 9152-9159. 10.1158/0008-5472.Can-04-2727.
19. Riesenberg, S., Chintalapati, M., Macak, D., Kanis, P., Maricic, T., and Pääbo, S. (2019). Simultaneous precise editing of multiple genes in human cells. Nucleic Acids Res *47*, e116. 10.1093/nar/gkz669.
20. Zhao, T., Li, Q., Zhou, C., Lv, X., Liu, H., Tu, T., Tang, N., Cheng, Y., Liu, X., Liu, C., et al. (2021). Small-molecule compounds boost genome-editing efficiency of cytosine base editor. Nucleic Acids Res *49*, 8974-8986. 10.1093/nar/gkab645.

21. Li, G., Zhang, X., Zhong, C., Mo, J., Quan, R., Yang, J., Liu, D., Li, Z., Yang, H., and Wu, Z. (2017). Small molecules enhance CRISPR/Cas9-mediated homology-directed genome editing in primary cells. Sci Rep *7*, 8943. 10.1038/s41598-017-09306-x.
22. Karthik, S., Sankar, R., Varunkumar, K., and Ravikumar, V. (2014). Romidepsin induces cell cycle arrest, apoptosis, histone hyperacetylation and reduces matrix metalloproteinases 2 and 9 expression in bortezomib sensitized non-small cell lung cancer cells. Biomed Pharmacother *68*, 327-334. 10.1016/j.biopha.2014.01.002.
23. Park, H., Shin, J., Choi, H., Cho, B., and Kim, J. (2020). Valproic Acid Significantly Improves CRISPR/Cas9-Mediated Gene Editing. Cells *9*. 10.3390/cells9061447.
24. Pinder, J., Salsman, J., and Dellaire, G. (2015). Nuclear domain 'knock-in' screen for the evaluation and identification of small molecule enhancers of CRISPR-based genome editing. Nucleic Acids Res *43*, 9379-9392. 10.1093/nar/gkv993.
25. Dutta, A., Eckelmann, B., Adhikari, S., Ahmed, K.M., Sengupta, S., Pandey, A., Hegde, P.M., Tsai, M.S., Tainer, J.A., Weinfeld, M., et al. (2017). Microhomology-mediated end joining is activated in irradiated human cells due to phosphorylation-dependent formation of the XRCC1 repair complex. Nucleic Acids Res *45*, 2585-2599. 10.1093/nar/gkw1262.
26. Iyer, S., Suresh, S., Guo, D., Daman, K., Chen, J.C.J., Liu, P., Zieger, M., Luk, K., Roscoe, B.P., Mueller, C., et al. (2019). Precise therapeutic gene correction by a simple nuclease-induced double-stranded break. Nature *568*, 561-565. 10.1038/s41586-019-1076-8.
27. Singh, P., Schimenti, J.C., and Bolcun-Filas, E. (2015). A mouse geneticist's practical guide to CRISPR applications. Genetics *199*, 1-15. 10.1534/genetics.114.169771.
28. Lee, J.S. (2007). Activation of ATM-dependent DNA damage signal pathway by a histone deacetylase inhibitor, trichostatin A. Cancer Res Treat *39*, 125-130. 10.4143/crt.2007.39.3.125.
29. Takayama, K., Igai, K., Hagihara, Y., Hashimoto, R., Hanawa, M., Sakuma, T., Tachibana, M., Sakurai, F., Yamamoto, T., and Mizuguchi, H. (2017). Highly efficient biallelic genome editing of human ES/iPS cells using a CRISPR/Cas9 or TALEN system. Nucleic Acids Res *45*, 5198-5207. 10.1093/nar/gkx130.
30. Delacôte, F., Han, M., Stamato, T.D., Jasin, M., and Lopez, B.S. (2002). An xrcc4 defect or Wortmannin stimulates homologous recombination specifically induced by double-strand breaks in mammalian cells. Nucleic Acids Res *30*, 3454-3463. 10.1093/nar/gkf452.
31. Yang, D., Scavuzzo, M.A., Chmielowiec, J., Sharp, R., Bajic, A., and Borowiak, M. (2016). Enrichment of G2/M cell cycle phase in human pluripotent stem cells enhances HDR-mediated gene repair with customizable endonucleases. Sci Rep *6*, 21264. 10.1038/srep21264.
32. Lin, S., Staahl, B.T., Alla, R.K., and Doudna, J.A. (2014). Enhanced homology-directed human genome engineering by controlled timing of CRISPR/Cas9 delivery. Elife *3*, e04766. 10.7554/eLife.04766.
33. Ma, X., Chen, X., Jin, Y., Ge, W., Wang, W., Kong, L., Ji, J., Guo, X., Huang, J., Feng, X.H., et al. (2018). Small molecules promote CRISPR-Cpf1-mediated genome editing in human pluripotent stem cells. Nat Commun *9*, 1303. 10.1038/s41467-018-03760-5.
34. Wienert, B., Nguyen, D.N., Guenther, A., Feng, S.J., Locke, M.N., Wyman, S.K., Shin, J., Kazane, K.R., Gregory, G.L., Carter, M.A.M., et al. (2020). Timed inhibition of CDC7 increases CRISPR-Cas9 mediated templated repair. Nat Commun *11*, 2109. 10.1038/s41467-020-15845-1.
35. Reint, G., Li, Z., Labun, K., Keskitalo, S., Soppa, I., Mamia, K., Tolo, E., Szymanska, M., Meza-Zepeda, L.A., Lorenz, S., et al. (2021). Rapid genome editing by CRISPR-Cas9-POLD3 fusion. Elife *10*. 10.7554/eLife.75415.
36. Labun, K., Guo, X., Chavez, A., Church, G., Gagnon, J.A., and Valen, E. (2019). Accurate analysis of genuine CRISPR editing events with ampliCan. Genome Res *29*, 843-847. 10.1101/gr.244293.118.
37. Tsai, S.Q., Zheng, Z., Nguyen, N.T., Liebers, M., Topkar, V.V., Thapar, V., Wyvekens, N., Khayter, C., Iafrate, A.J., Le, L.P., et al. (2015). GUIDE-seq enables genome-wide profiling of off-target cleavage by CRISPR-Cas nucleases. Nat Biotechnol *33*, 187-197. 10.1038/nbt.3117.
38. Zhu, L.J., Lawrence, M., Gupta, A., Pagès, H., Kucukural, A., Garber, M., and Wolfe, S.A. (2017). GUIDEseq: a bioconductor package to analyze GUIDE-Seq datasets for CRISPR-Cas nucleases. BMC Genomics *18*, 379. 10.1186/s12864-017-3746-y.
39. Savić, N., Ringnalda, F.C., Berk, C., Bargsten, K., Hall, J., Jinek, M., and Schwank, G. (2019). In vitro Generation of CRISPR-Cas9 Complexes with Covalently Bound Repair Templates for Genome Editing in Mammalian Cells. Bio Protoc *9*. 10.21769/BioProtoc.3136.
40. Savic, N., Ringnalda, F.C., Lindsay, H., Berk, C., Bargsten, K., Li, Y., Neri, D., Robinson, M.D., Ciaudo, C., Hall, J., et al. (2018). Covalent linkage of the DNA repair template to the CRISPR-Cas9 nuclease enhances homology-directed repair. Elife *7*. 10.7554/eLife.33761.

41. Picelli, S., Faridani, O.R., Björklund, A.K., Winberg, G., Sagasser, S., and Sandberg, R. (2014). Full-length RNA-seq from single cells using Smart-seq2. Nat Protoc *9*, 171-181. 10.1038/nprot.2014.006.
42. Zachariadis, V., Cheng, H., Andrews, N., and Enge, M. (2020). A Highly Scalable Method for Joint Whole-Genome Sequencing and Gene-Expression Profiling of Single Cells. Mol Cell *80*, 541-553.e545. 10.1016/j.molcel.2020.09.025.
43. Martin, M. (2011). Cutadapt Removes Adapter Sequences From High-Throughput Sequencing Reads. EMBnet.journal. https://doi.org/10.14806/ej.17.1.200.
44. Dobin, A., Davis, C.A., Schlesinger, F., Drenkow, J., Zaleski, C., Jha, S., Batut, P., Chaisson, M., and Gingeras, T.R. (2013). STAR: ultrafast universal RNA-seq aligner. Bioinformatics *29*, 15-21. 10.1093/bioinformatics/bts635.
45. McKenna, A., Hanna, M., Banks, E., Sivachenko, A., Cibulskis, K., Kernytsky, A., Garimella, K., Altshuler, D., Gabriel, S., Daly, M., and DePristo, M.A. (2010). The Genome Analysis Toolkit: a MapReduce framework for analyzing next-generation DNA sequencing data. Genome Res *20*, 1297-1303. 10.1101/gr.107524.110.
46. Anders, S., Pyl, P.T., and Huber, W. (2015). HTSeq--a Python framework to work with high-throughput sequencing data. Bioinformatics *31*, 166-169. 10.1093/bioinformatics/btu638.
47. Satija, R., Farrell, J.A., Gennert, D., Schier, A.F., and Regev, A. (2015). Spatial reconstruction of single-cell gene expression data. Nat Biotechnol *33*, 495-502. 10.1038/nbt.3192.
48. Korotkevich, G., Sukhov, V., Budin, N., Shpak, B., Artyomov, M.N., and Sergushichev, A. (2021). Fast gene set enrichment analysis. bioRxiv, 060012. 10.1101/060012.
49. Haas, B.J., Dobin, A., Li, B., Stransky, N., Pochet, N., and Regev, A. (2019). Accuracy assessment of fusion transcript detection via read-mapping and de novo fusion transcript assembly-based methods. Genome Biol *20*, 213. 10.1186/s13059-019-1842-9.
50. Danecek, P., Bonfield, J.K., Liddle, J., Marshall, J., Ohan, V., Pollard, M.O., Whitwham, A., Keane, T., McCarthy, S.A., Davies, R.M., and Li, H. (2021). Twelve years of SAMtools and BCFtools. GigaScience *10*. 10.1093/gigascience/giab008.
51. Huang, X., and Huang, Y. (2021). Cellsnp-lite: an efficient tool for genotyping single cells. Bioinformatics *37*, 4569-4571. 10.1093/bioinformatics/btab358.
52. Li, H. (2011). A statistical framework for SNP calling, mutation discovery, association mapping and population genetical parameter estimation from sequencing data. Bioinformatics *27*, 2987-2993. 10.1093/bioinformatics/btr509.
53. Das, S., Forer, L., Schönherr, S., Sidore, C., Locke, A.E., Kwong, A., Vrieze, S.I., Chew, E.Y., Levy, S., McGue, M., et al. (2016). Next-generation genotype imputation service and methods. Nat Genet *48*, 1284-1287. 10.1038/ng.3656.
54. Stubbington, M.J.T., Lönnberg, T., Proserpio, V., Clare, S., Speak, A.O., Dougan, G., and Teichmann, S.A. (2016). T cell fate and clonality inference from single-cell transcriptomes. Nat Methods *13*, 329-332. 10.1038/nmeth.3800.
55. Meier, F., Brunner, A.D., Frank, M., Ha, A., Bludau, I., Voytik, E., Kaspar-Schoenefeld, S., Lubeck, M., Raether, O., Bache, N., et al. (2020). diaPASEF: parallel accumulation-serial fragmentation combined with data-independent acquisition. Nat Methods *17*, 1229-1236. 10.1038/s41592-020-00998-0.
56. Demichev, V., Messner, C.B., Vernardis, S.I., Lilley, K.S., and Ralser, M. (2020). DIA-NN: neural networks and interference correction enable deep proteome coverage in high throughput. Nat Methods *17*, 41-44. 10.1038/s41592-019-0638-x.
57. Demichev, V., Szyrwiel, L., Yu, F., Teo, G.C., Rosenberger, G., Niewienda, A., Ludwig, D., Decker, J., Kaspar-Schoenefeld, S., Lilley, K.S., et al. (2022). dia-PASEF data analysis using FragPipe and DIA-NN for deep proteomics of low sample amounts. Nat Commun *13*, 3944. 10.1038/s41467-022-31492-0.
58. Lazar, C. (2015). imputeLCMD: a collection of methods for left-censored missing data imputation. R package, version 2.1.
59. Virtanen, P., Gommers, R., Oliphant, T.E., Haberland, M., Reddy, T., Cournapeau, D., Burovski, E., Peterson, P., Weckesser, W., Bright, J., et al. (2020). SciPy 1.0: fundamental algorithms for scientific computing in Python. Nat Methods *17*, 261-272. 10.1038/s41592-019-0686-2.
60. Seabold, S., and Perktold, J. (2010). Statsmodels: Econometric and Statistical Modeling with Python. SciPy.
61. Renesh, B. (2020). Reneshbedre/Bioinfokit: Bioinformatics Data Analysis and Visualization Toolkit| Zenodo.
