# Supplementary material for: Precision T cell correction platform for inborn errors of immunity
Source: Mol Ther. 2025 Aug 12;33(11):5624–43. doi: 10.1016/j.ymthe.2025.08.018 (PMC12628183; doi:10.1016/j.ymthe.2025.08.018)
Supplement: Document S2. Article plus supplemental information [file mmc9.pdf]

Molecular Therapy
Original Article

Precision T cell correction platform for inborn errors of immunity

Katariina Mamia,[1,2,3] Solrun Kolbeinsdottir,[4,22] Kornel Labun,[5,22] Zhuokun Li,[1] Anna Komisarczuk,[1,3] Salla Keskitalo,[6,7] Ganna Reint,[1] Frida Loe Haugen,[1,3] Britt Olaug Lindestad,[1,3] Siv Skundberg Jensen,[1,3] Thea Johanne Gjerdingen,[3,8] Antti Tuhkala,[7] Carolina Wieczorek Ervik,[1,3] Pavel Kopcil,[1,3] Nail Fatkhutdinov,[1] Karen Helene Bronken Martinsen,[9,10] Hans Christian Erichsen,[9,10] Monika Szymanska,[1,3] Eero Tölö,[11] Virpi Glumoff,[12,13] Janna Saarela,[1,14,15] Trond Melbye Michelsen,[16] Camilla Schalin-Jäntti,[17,18] Johanna Olweus,[3,8] Eira Leinonen,[19] Markku Varjosalo,[6,7] Eivind Valen,[5,20] Timo Hautala,[12,13] Martin Enge,[4] Timi Martelius,[21] Shiva Dahal-Koirala,[1,3,23] and Emma Haapaniemi[1,2,3,23]

[1]Centre for Molecular Medicine Norway, University of Oslo, 0318 Oslo, Norway; [2]Department of Pediatrics, Oslo University Hospital, 0372 Oslo, Norway; [3]Precision Immunotherapy Alliance, University of Oslo, 0379 Oslo, Norway; [4]Department of Oncology-Pathology, Karolinska Institutet, 17177 Stockholm, Sweden; [5]Computational Biology Unit, Department of Informatics, University of Bergen, 5008 Bergen, Norway; [6]Systems Biology/Pathology Research Group, University of Helsinki, 00014 Helsinki, Finland; [7]Institute of Biotechnology, HiLIFE, University of Helsinki, 00014 Helsinki, Finland; [8]Department of Cancer Immunology, Institute for Cancer Research, Oslo University Hospital Radiumhospitalet, 0310 Oslo, Norway; [9]Division of Pediatric and Adolescent Medicine, Oslo University Hospital and Institute of Clinical Medicine, University of Oslo, 0424 Oslo, Norway; [10]Faculty of Medicine, Institute of Clinical Medicine, University of Oslo, 0318 Oslo, Norway; [11]Ministry of Finance, 0030 Oslo, Norway; [12]Research Unit of Internal Medicine and Biomedicine, University of Oulu, 90014 Oulu, Finland; [13]ERN-RITA Core Center Member, RITAFIN Consortium, Infectious Diseases Clinic, Oulu University Hospital, 90220 Oulu, Finland; [14]Institute for Molecular Medicine Finland, HiLIFE, 00290 Helsinki, Finland; [15]Department of Medical Genetics, Oslo University Hospital, 0450 Oslo, Norway; [16]Department of Obstetrics, Division of Obstetrics and Gynecology, Oslo University Hospital, 0424 Oslo, Norway; [17]Endocrinology, Abdominal Center, Helsinki University Hospital, 00029 Helsinki, Finland; [18]University of Helsinki, ENDO-ERN (European Reference Network on Rare Endocrine Conditions), 00290 Helsinki, Finland; [19]Folkhälsan Institute of Genetics, and Stem Cells and Metabolism Research Program, University of Helsinki, 00014 Helsinki, Finland; [20]Department of Biosciences, University of Oslo, 0371 Oslo, Norway; [21]Inflammation Center, Department of Infectious Disease, Helsinki University Hospital and University of Helsinki, 00029 Helsinki, Finland

CRISPR-Cas9 gene editing is a promising tool to correct pathogenic variants for autologous cell therapies targeting inborn errors of immunity (IEI). Current strategies, such as gene knockout or cDNA knockin, address many single-gene defects but can disrupt gene expression, highlighting the need for precise correction platforms. While transplanting corrected autologous hematopoietic stem cells is a curative approach, it is unsuitable for patients with advanced disease, inflammation, or acute infections. As correcting T cells is an alternative therapeutic strategy for lymphoid IEIs, we present an efficient T cell single-nucleotide variant (SNV) correction platform based on homology-directed repair (HDR). By using STAT1 gain-of-function, cartilage hair hypoplasia, deficiency of ADA2, and autoimmune polyendocrinopathy-candidiasis-ectodermal dystrophy as IEI models, we demonstrate that our platform achieves up to 80% correction, with resultant functional correction of the disease phenotype in the selected models. Furthermore, we performed safety profiling using GUIDE-seq, single-cell RNA sequencing, long-read genome sequencing, and proteomics analysis and detected no genomic, transcriptomic, or proteomic aberrations. This study establishes HDR-based SNV editing as a portable method for developing clinical autologous T cell therapies and represents a promising step toward a broad-spectrum gene correction platform for treating diverse monogenic immune disorders.

INTRODUCTION

Inborn errors of immunity (IEI) encompass ∼555 single-gene defects that affect multiple cell types of the immune system, leading to diverse clinical presentations, including infection susceptibility, autoimmunity and inflammation, cancer predisposition, and allergies.[1,2] IEIs are popular targets for CRISPR-Cas9 gene correction as routine clinical protocols exist for immune cell transplantation. While hematopoietic stem and progenitor cells (HSPCs) are considered the prime target for full IEI correction,[3,4] the strategy is not always suitable due to severe clinical status, acute infections, or ongoing inflammation.[5,6]

Therapeutic benefit can also come from correcting patient T cells in IEIs that affect the T cell lymphoid compartment, such as CTLA-4

Received 14 February 2025; accepted 8 August 2025;
https://doi.org/10.1016/j.ymthe.2025.08.018.

[22]These authors contributed equally
[23]These authors contributed equally
Correspondence: Emma Haapaniemi, Centre for Molecular Medicine Norway, University of Oslo, 0318 Oslo, Norway.
E-mail: e.m.haapaniemi@ncmm.uio.no

 © 2025 The Authors.
Published by Elsevier Inc. on behalf of The American Society of Gene and Cell Therapy.
This is an open access article under the CC BY license (http://creativecommons.org/licenses/by/4.0/).

insufficiency.[7–13] The corrected cells can be infused to the patient as an adoptive T cell therapy to control infections, inflammation, and other pathology that stems from faulty T cell functions.[4] Autologous T cell transplantation offers advantages over hematopoietic stem cell (HSC) transplantation, including easier protocols for cell collection and reduced toxicity from lymphodepletion compared to the intensive chemotherapy required for HSC engraftment.[14] Furthermore, T cell editing does not pose the same safety concerns as editing of HSCs as they are terminally differentiated and carry a lower risk of insertional mutagenesis.[15,16] The efficacy of T cell editing for IEIs using viral delivery has previously been demonstrated for selected diseases both *in vitro* and *in vivo*,[7,10] highlighting the translational potential in targeting T cells for gene therapy (Table S1).

For some IEIs, knockout of the disease gene can restore normal cell function.[17,18] However, the main CRISPR-Cas9 correction strategy is to knockin the therapeutic cDNA under endogenous promoter of the diseased gene.[19,20] Although this strategy can treat most defects caused by a single gene, it may result in suboptimal expression of the cDNA construct due to a lack of endogenous regulatory sequences.[21,22] Furthermore, this strategy is slow to adapt to large genes, novel disease gene discoveries, or ultra-rare IEIs, which might feature only <10 patients globally. To overcome these issues, precise gene correction of the pathogenic variant is a therapeutic alternative.

Precise correction of monogenic mutations is typically attributed to base[23,24] and prime editing[24,25] as these methods do not induce double-stranded DNA (dsDNA) breaks and are thus considered safer alternatives.[26,27] However, identifying safe and efficient guides are a limiting factor for both. Large screens are necessary to identify an effective prime editing guide RNA (gRNA),[28–30] and guide options are limited for base editing, with a risk for bystander editing of the nearby coding bases.[31,32] CRISPR reagent design is better defined for "standard" CRISPR-Cas9, where availability of protospacer adjacent motifs (PAMs) define the number of available gRNAs per target site. Together with a repair template, homology-directed repair (HDR) occurs at the dsDNA break, which can be used to correct virtually all single-nucleotide variants (SNVs) and small indels in the human genome.

In this study, we have developed a T cell editing platform that utilizes CRISPR-Cas9-mediated HDR and can correct SNV mutations in diverse IEIs with up to 80% efficiency. We have used the following model IEIs for this proof-of-concept and platform development study: STAT-1 gain-of function (STAT1-GOF) (*STAT1*, c.1163A>G, NM_007315.3, p.K388R), ADA2 deficiency (*ADA2, c.506G>A, NM_001282225.2,* p.R169Q), autoimmune polyendocrinopathy-candidiasis-ectodermal dystrophy (APECED) (*AIRE, c.769C>T, NM_000383.4,* p.R257X), and cartilage hair hypoplasia (CHH) (*RMRP, NR_003051.3,* c.A71G). During platform development, we investigated several strategies for HDR enhancement to obtain high mutation correction levels and functional improvement in the disease phenotype.

RESULTS

gRNA design and repair strategy

gRNA design is crucial for the success of CRISPR experiments.[33] As *ADA2* p.R169Q has no available base editing guides, and AIRE and RMRP guides can induce bystander base editing (Figures S1A–S1C), we designed "standard" CRISPR guides for these model loci. We included guides with cut sites located within the 100-bp repair template (7–18 guides per locus) (Figure 1A). To prevent CRISPR re-cutting[34,35] and enable identical repair templates for healthy and patient cells, we designed single-stranded oligodeoxynucleotide (ssODN) repair templates, where 3–4 silent SNPs were added close to the mutation site (Figures 1B and S1D–S1F). This repair strategy also enabled rapid HDR detection in the edited samples by droplet digital PCR (ddPCR), with no differences between using an internal or external reference probe (Figures 1C, S2A, and S2B). Since *RMRP* encodes a non-coding RNA, we could not design silent SNPs to the locus and thus knocked in two variants of unknown function during the early optimization experiments (Figures S1F and S1G). In later studies, we corrected only the pathogenic variant (Figure S1H).

We first tested the correction strategy in the *ADA2* locus in healthy control T cells, fibroblasts, and CD34$^+$ HSPCs isolated from umbilical cord blood and compared the results to similar screens in deficiency of ADA2 (DADA2) patient T cells and fibroblasts (Figures 1D and 1E; all patients are homozygous for the *ADA2* p.R169Q mutation). We identified gRNA number 3 as the best guide for *ADA2* correction across cell types, with ~30% maximum HDR efficiency (Figure 1E). We then screened guides for *AIRE* and *RMRP* loci in homozygous patient T cells and fibroblasts and identified AIRE gRNA number 11 and RMRP gRNA number 9 as the best guides (Figures 1F and 1G, 10%–20% HDR). We assessed HDR in the samples also by deep amplicon sequencing with near-identical results (Figures S2C–S2E), confirming ddPCR as a reliable method for rapid HDR assessment. We did not observe a clear correlation between the HDR frequency and guide cutting distance from the mutation site (Figures S2F–S2H), possibly due to the sequence context and structural or thermodynamic properties of the tested gRNAs.[33,36] *In silico* gRNA design tools showed poor accuracy with this correction strategy, likely as they are built on datasets adapted for non-homologous end joining (NHEJ)-based gene knockout (Figures S2I–S2K).

Optimized T cell culture for editing enhancement

HDR-dependent correction happens in the S/G2 phases of the cell cycle.[37] Therefore, optimal T cell expansion and viability can further increase gene correction.[38] As a baseline, we used common T cell editing protocols and our previous work,[39–41] where peripheral blood mononuclear cells (PBMCs) are first stimulated for 3 days, then nucleofected with CRISPR-Cas9 ribonucleoprotein complexes (RNPs), and collected for DNA extraction 3–5 days post-nucleofection (Figure 2A). We noted ~15%–30% *ADA2* and ~5%–8% *AIRE* HDR editing in healthy controls, with editing levels plateauing 3

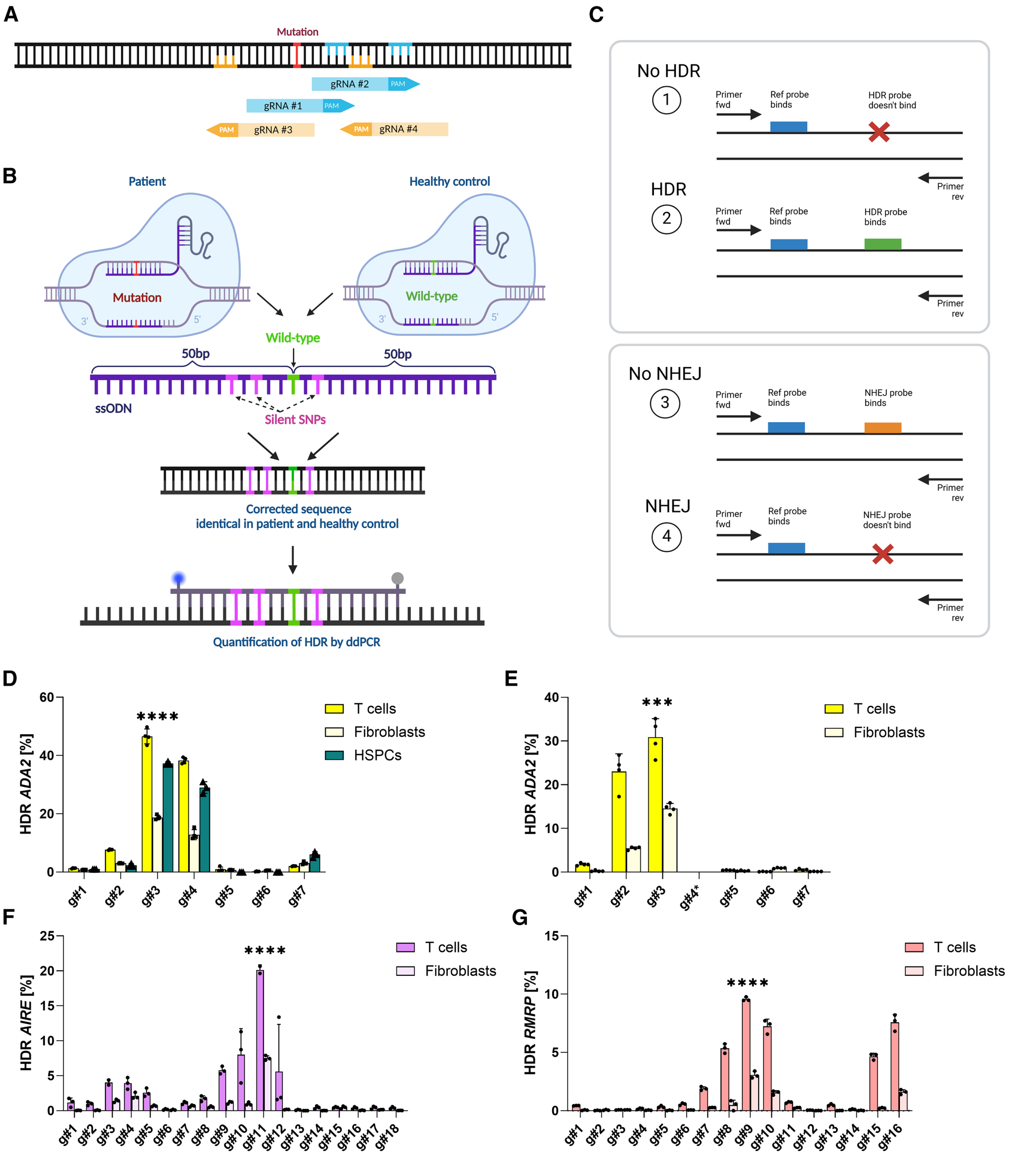

Figure 1. Repair strategy and gRNA screening in patient cells

(A) Schematic representation of the gRNA screening strategy, where multiple gRNAs were assessed based on available PAM sites within the 100-bp ssODN area. Forward gRNAs and their PAMs are marked in blue and reverse in yellow. (B) Schematic representation of the repair strategy used in the study, where 100-bp ssODNs with ±50-bp homology arms from the mutation site (red) were used. ssODN design includes correction of the mutation (green) and 3–4 silent SNVs (pink), enabling identical editing strategy in patients and healthy controls and HDR detection by ddPCR. (C) Schematic representation of the ddPCR assay design for HDR and NHEJ detection. (D) ADA2 gRNA screening in HD T cells, fibroblasts and $CD34^+$ HSPCs, assessed by ddPCR ($n = 4$ technical replicates for T cells and fibroblasts, $n = 3$ for HSPCs). (E) ADA2 gRNA screening in DADA2 patient T cells and fibroblasts, assessed by ddPCR ($n = 3$ technical replicates). ADA2 gRNA number 4 (asterisk) was not tested in patients due to PAM loss caused by the mutation. (F) AIRE gRNA screening in APECED patient T cells and fibroblasts, assessed by ddPCR ($n = 3$ technical replicates). (G) RMRP gRNA screening in CHH patient T cells and fibroblasts, assessed by ddPCR ($n = 3$ technical replicates). One independent experiment was performed for all sets of data. Statistical significance of best-performing gRNAs was assessed by one-way ANOVA with Fisher's least significant difference (LSD) test, where ***$p < 0.0002$ and ****$p < 0.0001$. Bar denotes mean value, error bars represent ±SD.

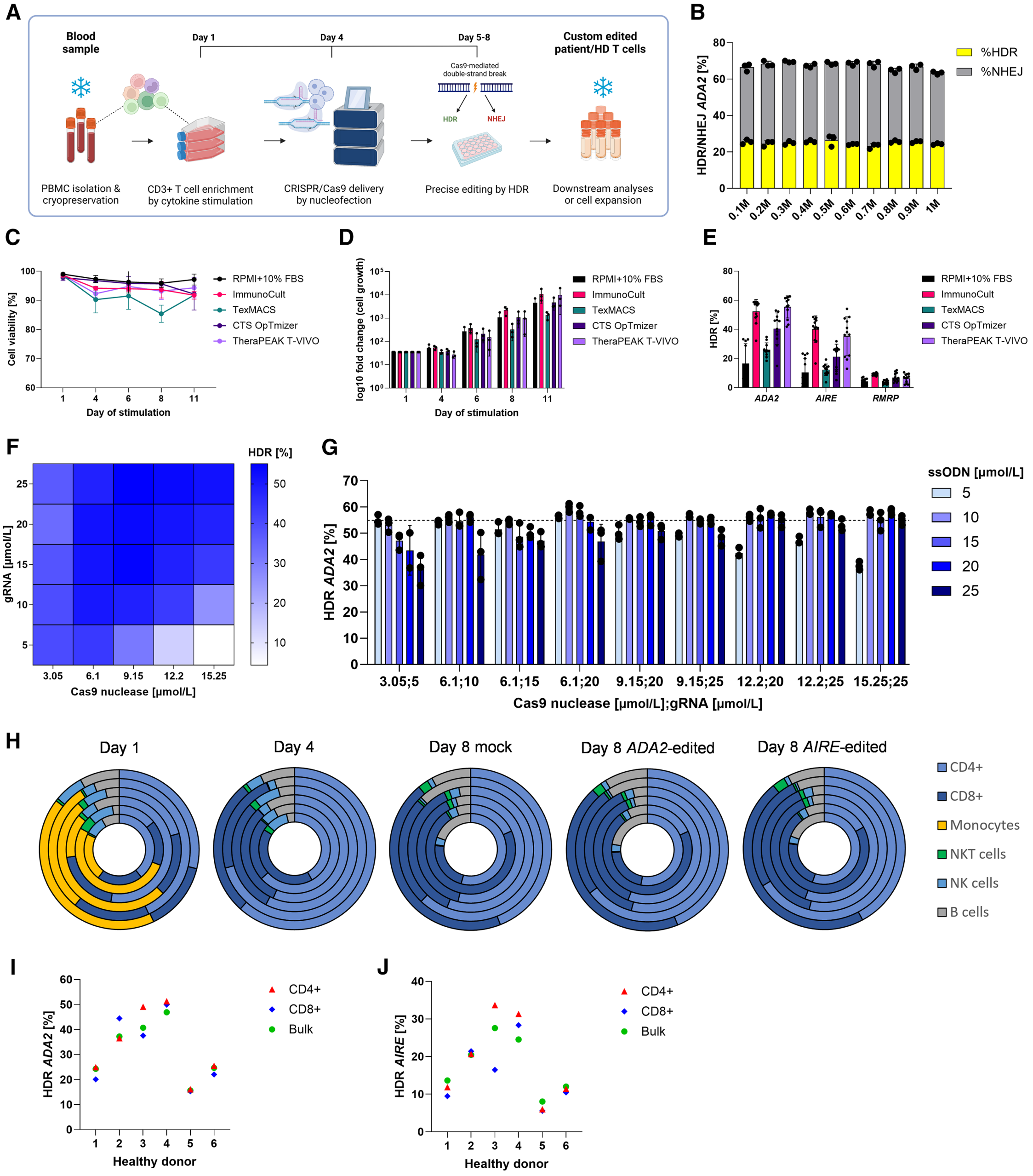

Figure 2. Establishment and assessment of CRISPR-Cas9 T cell editing platform

(A) Schematic representation of the CRISPR-Cas9 T cell editing platform. PBMCs from patient and HD blood samples are first isolated and cryopreserved. PBMCs are thawed on day 1 and stimulated for 3 days with interleukins: IL-2 (120 U/mL), IL-7 (3 ng/μL), and IL-15 (3 ng/μL) and soluble CD3/CD28 (15 μL/mL), which activate and induce expansion of CD3^{+} T cells. Cells are nucleofected on day 4 with custom CRISPR reagents (gRNA, Cas9 nuclease, ssODN). Afterward, cells are cultured for 4 days in IL-2 (250 U/mL), during which Cas9-mediated double-stranded breaks are repaired by HDR/NHEJ. On day 8, cells are harvested for downstream assays, expanded further, or cryopreserved. (B) *ADA2* HDR and NHEJ editing in HD T cells with 0.1–1 M nucleofected cells/sample, measured by ddPCR (*n* = 3 technical replicates). Comparison of different T cell culture media during 11-day cytokine stimulation, assessed by (C) T cell viability (dots represent mean of *n* = 3 biological replicates), (D) T cell fold change (*n* = 3 biological replicates), and (E) *ADA2*, *AIRE,* and *RMRP* HDR editing on day 8 (*n* = 3 technical ddPCR replicates from *n* = 3 biological replicates). (F) *ADA2* HDR editing in HD T cells with Cas9 nuclease at 3.05–15.25 μmol/L/sample, gRNA at 5–25 μmol/L/sample, and ssODN at 5 μmol/L/sample/sample, measured by ddPCR (*n* = 3 technical replicates). (G) *ADA2* HDR editing in HD T cells with selected Cas9-gRNA concentrations and ssODN at 5–25 μmol/L/sample, measured by ddPCR (*n* = 3 technical replicates). Dashed line indicates mean of Cas9 nuclease at 3.05 μmol/L/sample, gRNA at 5 μmol/L/sample, and ssODN at 5 μmol/L/sample. (H) Frequency of immune cells

(legend continued on next page)

(*ADA2*) and 2 (*AIRE*) days after nucleofection and staying consistent for up to 14 days after nucleofection (Figures S3A and S3B). The number of cells used for nucleofection had no effect on the final editing level, allowing us to work with less material when necessary (Figure 2B). We thus settled for 0.5–1 million cells per nucleofection and standardized sample collection on day 4 post-nucleofection.

To improve T cell proliferation, viability, and HDR, we compared several GMP-compatible T cell media while editing *ADA2*, *AIRE*, and *RMRP* loci (Figures 2C–2E). Based on the results across tested donors, Immunocult and TheraPEAK T-VIVO performed similarly. Since Immunocult supported cell proliferation earlier, we selected it as the basal medium and supplemented it with 120 U/mL interleukin-2 (IL-2), 3 ng/μL IL-7, 3 ng/μL IL-15, and 15 μL/mL soluble CD3/CD28 T cell activator to obtain the T cell stimulation cocktail for selective $CD3^+$ T cell expansion from PBMCs. We also titrated the concentrations of Cas9 nuclease, gRNA, and repair template, with the goal of reaching optimal reagent concentration in the nucleus without excessive toxicity (Figures 2F, 2G, S3C, and S3D). Based on the results, we standardized Cas9 nuclease at 3.05, gRNA at 5, and ssODN at 5 μmol/L per nucleofected sample.

To verify selective $CD3^+$ T cell expansion from PBMCs, we quantified the immune cell populations on culture days 1, 4, and 8 from 6 healthy controls by flow cytometry (Figure 2H). While PBMC population diversity is considerable on day 1, it gradually disappears during cytokine stimulation. By day 8, $CD4^+$ and $CD8^+$ T cells make up ∼80% of all cells. On day 8, it is possible to sort, cryopreserve, or further expand the T cells. Although we noted interindividual and locus-specific variation in editing efficiency, also described by others,[38,41–43] HDR editing levels in $CD4^+$ and $CD8^+$ subsets were similar within each donor (Figures 2I and 2J).

Refined repair template design for HDR enhancement

Positioning and format of the repair template affects HDR editing.[35,44–46] For optimal repair template positioning, we designed asymmetric 100-bp templates for *ADA2*, *AIRE*, and *RMRP* loci, with 10- to 90-bp homology arms on either side (9 templates per locus, all reverse complementary to the guide; Figure 3A).[44] The symmetric templates with 50-bp homology arms proved best for *ADA2* and *RMRP*; however, *AIRE* locus edited most optimally with an asymmetric template (30 bp left homology arm, 70 bp right homology arm, Figures 3B–3D).

Coupling the repair template to Cas9 improves HDR editing, presumably by enhancing nuclear import and template positioning at the cut site.[47,48] To test this, we synthesized 5′ benzylguanine (BG)-coupled repair templates that can bind covalently to Cas9-SNAP fusion protein.[48,49] BG templates led to 2-fold *ADA2* HDR enhancement in fibroblasts and T cells with both Cas9-wild type (WT) and Cas9-SNAP RNPs (Figures S4A–S4C), suggesting alternative HDR enhancing mechanisms independent of the Cas9-SNAP coupling. The commercially available 3′ phosphorothioate (PT) and locked nucleic acid (LNA) modifications led to similar HDR improvements in T cells, fibroblasts, and $CD34^+$ HSPCs (Figures 3E–3G and S4D–S4F). 3′ PT and LNA modifications likely protect the ssODN from 3′ endonucleases such as TREX1,[50] resulting in increased stability of the oligo and enhanced HDR. We chose 2PT 3′ modified repair templates for further experiments due to their universal effectiveness and ease of synthesis (Figures S4G–S4I).

Inhibition of DNA-PKcs further improves HDR

A substantial number of HDR-enhancing chemicals have been published. We reviewed 33 compounds convincingly reported as HDR enhancers (Table S12) and tested them in the *ADA2* locus in healthy control T cells at three concentrations based on previously reported effective concentrations in cell lines and primary cells. Most compounds decreased HDR, likely due to cell toxicity. Three compounds led up to 80% efficiencies in screening conditions (Figure 3H): the DNA-dependent protein kinase catalytic subunit (DNA-PKcs) inhibitors NU7441[51] and KU0060648[51] and Integrated DNA Technologies (IDT) Alt-R enhancer V2 (hereafter referred to as IDT Alt-R; compound identity undisclosed). We validated these three compounds in six endogenous loci (*ADA2*, *AIRE*, *CTCF-1*, *Enh4-1*, *RMRP*, and *RNF2*), optimized their concentrations in healthy controls, and tested them further in DADA2, APECED, and CHH patient T cells, consistently achieving minimal toxicity, ∼2-fold improvement, and up to 80% mutation correction, depending on the target locus and individual (Figures 3I, 3J, S4J, and S4K). The compounds improved editing even in $CD34^+$ HSPCs derived from healthy donor umbilical cord blood (Figure 3K). High HDR levels were maintained when nucleofection was performed at passages 1–3, decreasing in later passages (Figure 3L). Increased HDR levels were also observed in patient and healthy control samples analyzed by amplicon sequencing (Figures S5 and S6).

As HDR is dependent on the S/G2 phases of the cell cycle,[37] we also tested a set of cell-cycle inhibitors (Table S13) for their ability to synchronize editing to S/G2 phases and consequently increase HDR. Hydroxyurea[52] emerged as an unexpected HDR enhancer when applied 24 h before nucleofection, but because the effect was suboptimal in comparison to IDT Alt-R, we did not explore the strategy further (Figures S4L and S4M).

Adapted GUIDE-seq off-target profiling for patient and healthy control T cells

Genome-wide, unbiased identification of double-strand breaks enabled by sequencing (GUIDE-seq) finds CRISPR off-target cuts

($CD4^+$, $CD8^+$, monocytes, NKT cells, NK cells, B cells) in six HDs on days 1, 4, and 8 (mock, *ADA2* edited, or *AIRE* edited) of the platform, assessed by flow cytometry. Each ring of the doughnut plot represents one HD. HDR editing levels in $CD4^+$ and $CD8^+$ and the bulk of cells for *ADA2* (I) and *AIRE* (J) on day 8, measured by ddPCR (n = 1 technical replicate). One independent experiment was performed for all sets of data except for (C)–(E), where data from three donors are shown in the graphs, and (F) and (G), where one out of three representative experiments is shown. Bar denotes mean value, error bars represent ±SD.

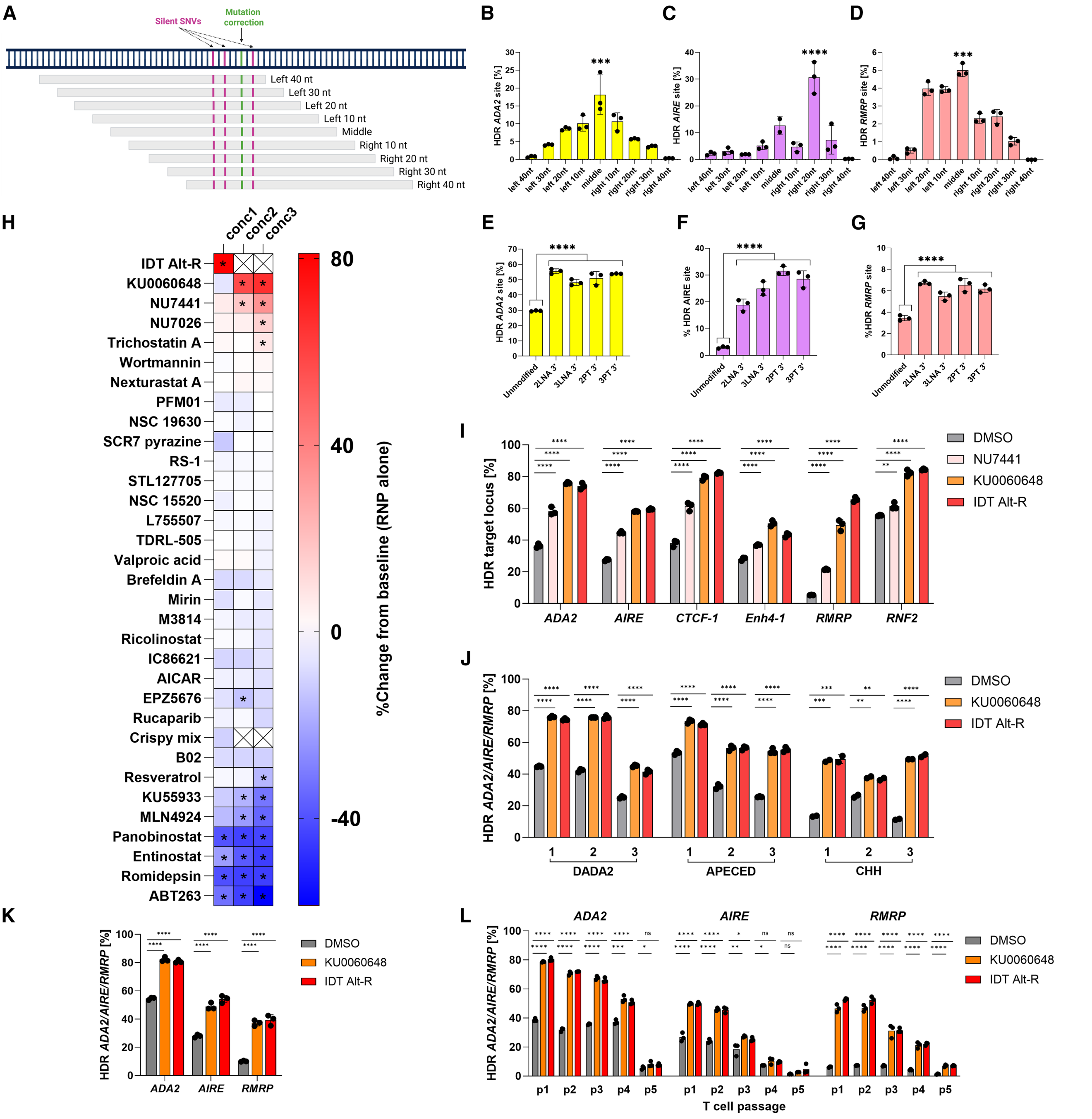

Figure 3. HDR enhancement in healthy control and patient T cells

(A) Schematic representation of asymmetric ssODN designs with 10- to 90-bp homology arms on either side from target site. HDR editing with asymmetric ssODNs in HD T cells for (B) *ADA2*, (C) *AIRE*, and (D) *RMRP*, measured by ddPCR ($n = 3$ technical replicates). HDR editing with 3′ LNA- or 3′ PT-modified, position-optimized ssODNs in HD T cells for (E) *ADA2*, (F) *AIRE*, and (G) *RMRP*, measured by ddPCR ($n = 3$ technical replicates). (H) Validation of HDR-enhancing compounds at three concentrations in increasing order (conc1–3) for *ADA2* editing in HD T cells, assessed by ddPCR ($n = 2$ technical replicates per condition). Compounds were assessed in three HDs, where mean of all donors per condition were compared to the mean of edited DMSO-treated baseline. Statistical significance was assessed by ANOVA. For the heatmap, percentage of HDR fold change from baseline was calculated for each compound concentration. Statistically significant concentrations are indicated with black asterisks. Conc2–3 are marked with a cross for those compounds that were assessed at one concentration. (I) HDR editing for *ADA2*, *AIRE*, *CTCF1*, *Enh4-1*, *RMRP*, and *RNF2*, measured by ddPCR ($n = 3$ technical replicates) with selected HDR-enhancing compounds (4 μM NU7441, 0.5 μM KU0060648, and 1 μM IDT Alt-R enhancer V2) or DMSO in HD T cells. (J) HDR in DADA2, APECED, and CHH patient T cells with concentration-optimized HDR enhancing compounds (0.5 μM KU0060648 and 0.6 μM IDT Alt-R

(legend continued on next page)

by transfecting cells with modified dsDNA oligos (dsODNs) along the CRISPR RNP complex, and then selectively amplifying and sequencing the oligo integration sites.[53] The existing GUIDE-seq data mainly come from cell lines.[53] There are reports for adaptations to T cells,[39,54] but since dsODNs can be particularly toxic to patient T cells, we started the off-target profiling by optimizing the dsODN concentration for improved cell viability. Experiments in healthy control T cells for guides targeting the *ADA2* and *HEK-site 4* loci (positive control guide with multiple off-targets[53]) showed acceptable cell viabilities and optimal dsODN integration with dsODN at 1–5 μmol/L per nucleofected sample (Figures S7A–S7F). Subsequent deep sequencing detected no off-targets for ADA2 guide but recovered several integrants for HEK-site 4, validating the sensitivity of the method (Figures S7C and S7F).

To account for increased dsODN toxicity in patient T cells, we refined the dsODN concentration further in DADA2 patient T cells, settling on dsODN at 1.5 μmol/L per nucleofected sample based on cell viability, dsODN integration, and cell yield (Figures 4A–4C). Finally, we performed GUIDE-seq in three patients and three healthy controls for each locus and confirmed the safety of ADA2 gRNA number 3, AIRE gRNA number 11, and RMRP gRNA number 9 with no off-targets, contrasting with multiple off-targets for *HEK-site 4* (Figures 4D–4H). To summarize, we present a refined GUIDE-seq protocol for T cell CRISPR-Cas9 off-target profiling and recommend lower dsODN concentrations for IEI patient samples to reach optimal cell viability and reliable sequencing results.

Long-read sequencing and single-cell transcriptomics reveal no aberrant changes in the karyotype, transcriptome, and T cell receptor repertoire of edited T cells

CRISPR-Cas9 can cause various chromosomal aberrations,[55,56] which increase the risk for malignant transformation and complicate clinical translation of genome editing. To evaluate the translational potential of our HDR enhancement strategies, we performed a comprehensive safety assessment using state-of-art technologies for genomic and transcriptomic analysis of the edited T cells.

We first performed PacBio long-read sequencing to map unintended edits in DADA2 and healthy control T cells. We edited the cells with or without KU0060648 and harvested cells 6 days post-nucleofection (Figures S8A and S8B; detailed visualization of the cut site is available in Figure S8C). We quantified a mean coverage of 25× across the genome, with 4/31 (∼47%, RNP only) and 12/18 (∼67%, RNP^+ KU0060648) reads containing the desired edit, respectively. We noted additional on-target indels between ∼3 and 300 bp, and a ∼1.2-kb on-target deletion in one HiFi read in the KU0060648-treated sample. Only one read per edited sample contained no on-target alterations, demonstrating that virtually all cells had been exposed to editing reagents. We did not find any chromosomal translocations or integrated repair template concatemers at the intended cut site or elsewhere in the genome (Figure S8C); however, increased sequencing depth might uncover additional low-frequency events. SNVs, small insertions or deletions outside the cut site, were shared between the experimental conditions and were not suggestive of aberrant mutational signatures or indicative of cancer (Figures S8D and S9A).[57]

Next, we performed single-cell RNA sequencing (scRNA-seq) of full-length mRNA to search for karyotypic and transcriptomic changes. DADA2 patient and matched healthy control T cells were nucleofected with or without ADA2 RNPs and treated with HDR enhancers (KU0060648, IDT-Alt-R) or DMSO (total of six treatment groups; Figures 5A and 5B). Four days post-nucleofection, we sorted equal amounts of single $CD4^+$ and $CD8^+$ cells in plates for library preparation (Figure 5A). Cultures exposed to HDR enhancers had a slight underrepresentation of $CD4^+$ cells and an overrepresentation of $CD8^+$ cells (Figures S10A and S10B). scRNA quantitative reverse transcription-PCR (RT-qPCR) detected the presence of the corrected RNA transcript in ∼80% of the RNP-treated and >98% of the HDR enhancer-treated cells, indicating that nearly all cells had been exposed to editing reagents and harbored at least one corrected allele (Figures 5C, S11A, and S11B).

The scRNA-seq data showed minimal effect of editing on the general transcriptomic profile as the edited cells clustered with unedited cells in both the control and DADA2 patient (Figures 5D–5K). The samples edited with the presence of DMSO and KU0060648 showed a slight downregulation of the p53 response, likely as an adaptation to the transient p53 upregulation[41] when the *ADA2* gene was cut. KU0060648 also affected metabolism slightly, likely due to the compound's bystander effect on phosphatidylinositol 3-kinase.[58] IDT Alt-R showed a downregulation of immune response pathways in the healthy control (Figure 5J). In addition, all samples recovered low-frequency non-recurring novel fusion transcripts (Table S18). Fusion transcripts that mapped to genes in chromosome 22 (where *ADA2* resides) were not found in >1 cell per condition. scRNA-seq data showed no loss of heterozygosity, indicative of no identifiable loss of chromosomal material. In addition, the T cell repertoire was polyclonal, and editing did not diminish the T cell receptor diversity (Figures S12A and S12B).

enhancer V2), where *ADA2*, *AIRE*, and *RMRP* loci, respectively, were corrected. HDR levels were assessed by ddPCR for *ADA2* and *AIRE* (*n* = 3 technical replicates) and by amplicon sequencing for *RMRP* (*n* = 2 technical replicates). (K) *ADA2*, *AIRE*, and *RMRP* HDR editing in HD $CD34^+$ HSPCs, measured by ddPCR (*n* = 3 technical replicates) with concentration-optimized HDR enhancing compounds (0.5 μM KU0060648 and 0.6 μM IDT Alt-R enhancer V2) or DMSO. (L) HDR editing in *ADA2*, *AIRE*, and *RMRP* in HD T cells at different cell passages (p1–p5), measured by ddPCR (*n* = 3 technical replicates) with concentration-optimized HDR enhancing compounds (0.5 μM KU0060648 and 0.6 μM IDT Alt-R enhancer V2) or DMSO. Three independent experiments were performed for all sets of data where representative experiment is shown, except for (H) where average measurements from three HDs is shown and (J) where all patients are shown in the graph. Bar denotes mean value, error bars represent ±SD. Statistical significance for all sets of data, except (H), was assessed by one-way ANOVA with Fisher's LSD test, where *$p < 0.01$, **$p < 0.001$, ***$p < 0.0002$, and ****$p < 0.0001$.

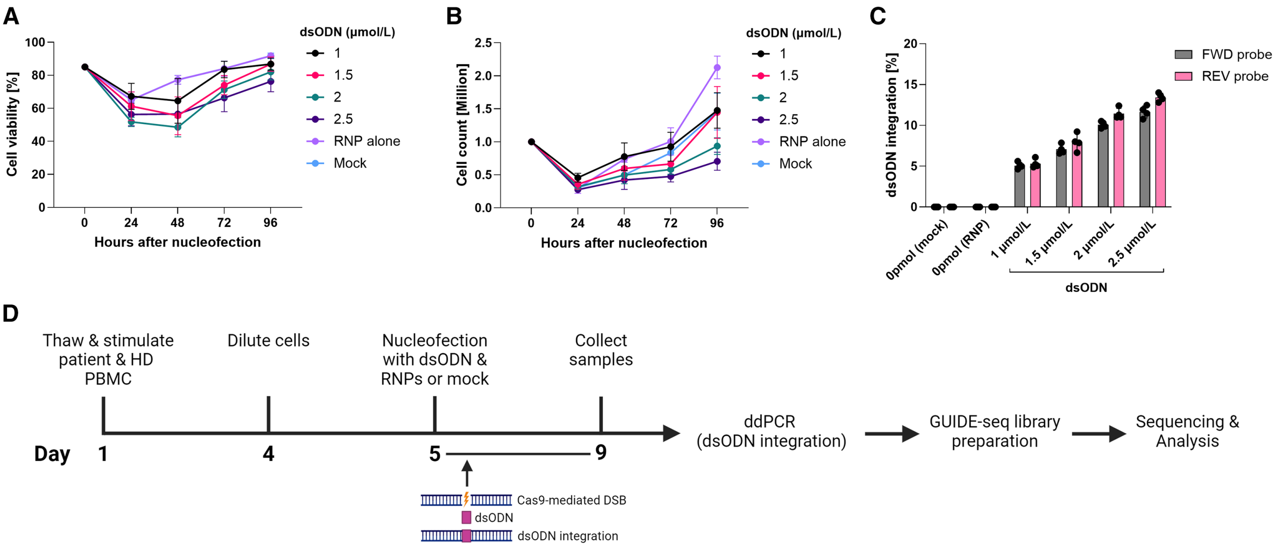

E

																								HD 1	HD 2	HD 3	DADA2 1	DADA2 2	DADA2 3
chr22:17207106:-	T	G	C	T	G	G	A	G	G	A	T	T	A	T	C	G	G	A	A	G	N	G	G	445	153	613	512	379	334

F

																								HD 1	HD 2	HD 3	APECED 1	APECED 2	APECED 3
chr21:44289770:-	G	C	T	C	C	C	T	T	G	G	C	T	C	G	A	A	C	C	A	G	N	G	G	339	142	414	189	389	272
chr13:63848783:-	-	|	|	|	|	|	|	|	|	|	|	-	|	|	|	|	|	|	|	|	|	|	|	17	NA	NA	NA	NA	NA
chr6:56394092:+	-	|	|	|	|	|	|	|	|	|	|	-	|	|	|	|	|	|	|	|	|	|	|	NA	NA	15	NA	NA	NA

G

																								HD 4	HD 5	HD 6	CHH 1	CHH 2	CHH 3
chr9:35657959:+	G	A	C	T	T	T	C	C	C	C	T	A	G	G	C	G	G	A	A	A	N	G	G	151	145	96	156	282	127

H

																								HD 1	HD 2	HD 3	HD 4	HD 5	HD 6	DADA2 1	DADA2 2	DADA2 3	APECED 1	APECED 2	APECED 3	CHH 1	CHH 2	CHH 3
chr20:32761966:+	G	G	C	A	C	T	G	C	G	G	C	T	G	G	A	G	G	T	G	G	N	G	G	158	182	282	119	78	26	204	213	224	208	306	251	88	85	30
chr4:55949032:+	|	|	|	|	A	|	|	|	|	|	|	|	|	|	|	|	|	C	|	|	|	|	|	60	45	87	20	23	21	79	55	72	77	93	86	60	50	14
chr10:125006305:+	|	|	|	|	|	-	|	|	|	|	|	|	|	|	|	|	|	|	|	|	|	|	|	37	73	93	18	18	NA	77	68	97	90	75	87	49	38	NA
chrX:105602037:-	|	-	|	T	|	|	|	|	|	|	|	|	|	|	|	|	|	A	|	|	|	|	|	51	30	45	27	NA	NA	91	73	83	74	128	104	35	60	NA
chr15:40752044:-	|	|	|	G	|	|	|	|	|	|	|	G	|	|	|	|	|	|	|	|	|	|	|	49	50	74	15	NA	NA	85	57	79	71	73	101	21	17	NA
chr6:160096849:-	|	|	|	|	|	|	|	|	T	|	|	|	|	|	G	|	|	|	|	|	|	|	|	19	24	40	NA	NA	NA	24	22	31	26	27	25	20	NA	NA
chr20:61435506:+	|	-	|	|	|	|	|	|	|	|	|	C	|	|	|	|	|	A	|	|	|	|	|	NA	20	37	NA	NA	NA	28	20	38	24	27	20	NA	NA	NA
chr16:50266435:+	|	-	|	|	|	|	|	T	|	|	|	|	|	|	G	|	|	A	|	|	|	|	|	19	NA	38	NA	NA	NA	20	NA	23	22	27	25	NA	NA	NA
chr7:54493744:+	|	|	-	|	|	|	|	|	|	|	|	|	|	|	G	|	|	|	|	|	|	|	|	NA	32	20	NA	NA	NA	28	NA	25	NA	23	23	NA	NA	NA
chr17:77433198:-	|	A	|	|	|	C	A	|	|	|	|	|	|	|	|	|	A	|	|	|	|	|	|	NA	NA	29	NA	NA	NA	26	NA	18	19	22	26	NA	NA	NA
chr13:27055273:-	|	|	|	|	|	|	|	G	|	|	T	|	|	|	|	|	|	|	|	|	|	|	|	NA	NA	28	NA	NA	NA	NA	NA	35	19	18	19	NA	NA	NA
chr10:75343361:+	|	|	|	|	-	|	A	|	|	|	|	|	|	|	|	|	|	|	|	|	|	|	|	NA	13	21	NA	NA	NA	22	NA	25	NA	19	NA	NA	NA	NA
chr13:38688791:+	|	-	|	|	G	|	|	|	|	|	|	|	A	|	|	|	|	|	|	|	|	|	|	NA	NA	21	NA	NA	NA	NA	NA	27	22	NA	16	NA	NA	NA
chr19:32891175:-	|	|	|	T	|	|	|	|	|	|	|	|	|	|	|	|	|	G	|	|	|	|	|	NA	20	28	NA	NA	NA	NA	NA	NA	NA	NA	NA	NA	NA	NA
chr19:38125546:-	|	|	|	|	|	|	|	A	|	A	|	|	|	|	G	|	|	|	|	|	|	|	|	NA	NA	NA	NA	NA	NA	NA	NA	19	NA	NA	NA	NA	NA	NA
chr20:1171210:-	|	|	|	|	|	|	|	T	|	|	|	|	|	C	|	|	|	|	|	|	|	|	|	NA	NA	17	NA	NA	NA	NA	NA	NA	NA	NA	NA	NA	NA	NA

Figure 4. gRNA off-target profiling by GUIDE-seq in patient and healthy control T cells

DADA2 patient T cell (A) viability ($n = 4$ technical replicates) and (B) count ($n = 4$ technical replicates) 24–96 h after nucleofection with 0–2.5 µmol/L/sample dsODN and ADA2 RNPs. (C) dsODN integration 96 h after nucleofection in DADA2 patient T cells, with 0–2.5 µmol/L/sample dsODN and ADA2 RNPs, assessed by ddPCR ($n = 3$ technical replicates) using forward (gray) and reverse (pink) dsODN probes for detection. (D) Schematic representation of the GUIDE-seq experiment. DADA2, APECED, and CHH patient and HD PBMCs were thawed and stimulated with IL-2 (120 U/mL), IL-7 (3 ng/µL), IL-15 (3 ng/µL), and soluble CD3/CD28 (15 µL/mL) on day 1, diluted on day 4, and nucleofected on day 5 with 1.5 µmol/L/sample dsODN and selected RNPs or mock. Cells were cultured in IL-2 (250 U/mL) until sample collection on day 9, followed by genomic DNA (gDNA) extraction, ddPCR for dsODN integration, and GUIDE-seq library preparation. GUIDE-seq in patient and HD T cells for (E) ADA2 gRNA number 3, (F) AIRE gRNA number 11, (G) RMRP gRNA number 9, and (H) HEK-site 4 gRNA, targeting the endogenous human embryonic kidney *HEK-site 4*. GUIDE-seq results are shown as mismatch plots, where the on-target sequence is depicted at the first line of the table with sequencing read counts per individual (right). The most abundant off-targets, if applicable, are listed under the target with their corresponding locations in the genome (left) and sequencing read counts (right). One independent experiment was performed for all sets of data. Bar denotes mean value, error bars represent ±SD.

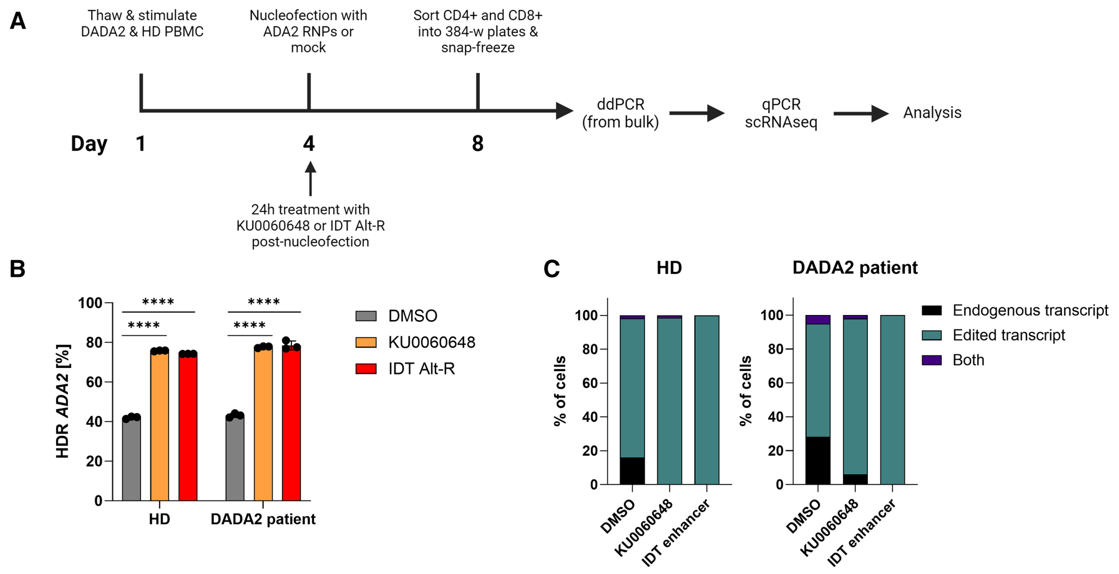

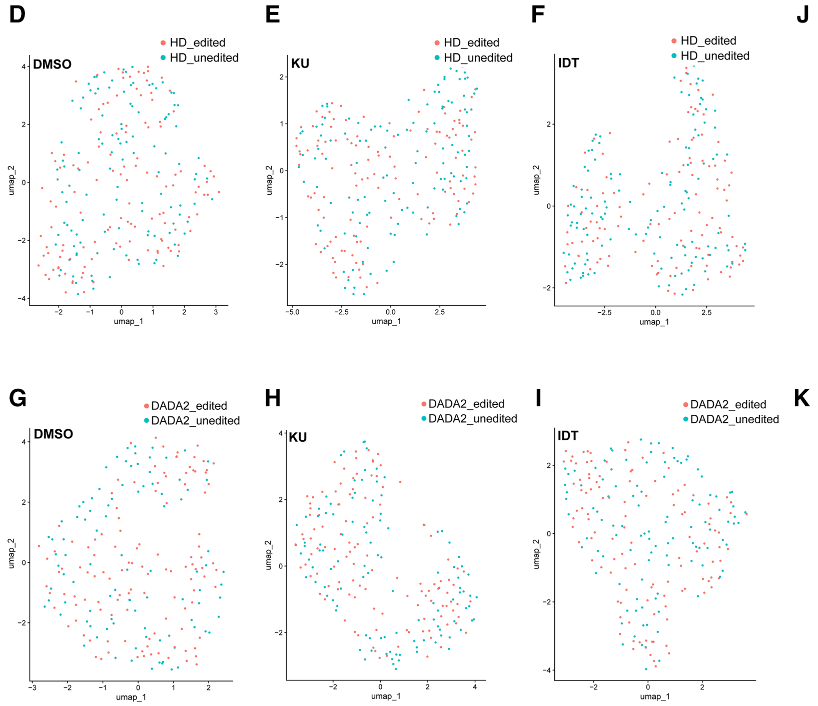

J

RNP + DMSO	P adjusted	NES
P53_PATHWAY	1.5e−02	−1,64
XENOBIOTIC_METABOLISM	4.6e−02	−1,57
**RNP + KU0060648**	**P adjusted**	**NES**
HYPOXIA	7.2e−06	−1.98
E2F_TARGETS	1.2e−04	1,74
GLYCOLYSIS	1.0e−03	−1.74
G2M_CHECKPOINT	1.1e−03	1,63
MYOGENESIS	8.7e−03	−1.67
P53_PATHWAY	3.4e−02	−1.50
**RNP + IDT Alt-R**	**P adjusted**	**NES**
INTERFERON_GAMMA_RESPONSE	1.2e−15	−2.51
INTERFERON_ALPHA_RESPONSE	4.6e−11	−2.55
TNFA_SIGNALING_VIA_NFKB	6.0e−03	−1.62
EPITHELIAL_MESENCHYMAL_TRANSITION	1.2e−02	−1.66
IL2_STAT5_SIGNALING	1.9e−02	−1.49
G2M_CHECKPOINT	3.8e−02	1,45
INFLAMMATORY_RESPONSE	4.7e−02	−1.51
MYOGENESIS	4.7e−02	−1.51

K

RNP + DMSO	P adjusted	NES
G2M_CHECKPOINT	1.7e−05	1,86
E2F_TARGETS	2.1e−05	1,78
TNFA_SIGNALING_VIA_NFKB	2.7e−04	-1,79
EPITHELIAL_MESENCHYMAL_TRANSITION	6.2e−03	-1,69
MYOGENESIS	1.2e−02	-1,65
P53_PATHWAY	1.5e−02	-1,54
MITOTIC_SPINDLE	2.7e−02	1,40
**RNP + KU0060648**	**P adjusted**	**NES**
P53_PATHWAY	9.5e−03	-1,61
HYPOXIA	9.5e−03	-1,63
TNFA_SIGNALING_VIA_NFKB	4.4e−03	-1,69
G2M_CHECKPOINT	2.2e−02	1,46
EPITHELIAL_MESENCHYMAL_TRANSITION	2.3e−02	-1,6
**RNP + IDT Alt-R**	**P adjusted**	**NES**
G2M_CHECKPOINT	1.5e−05	1,74
E2F_TARGETS	9.1e−05	1,61
MITOTIC_SPINDLE	1.1e−04	1,63

Figure 5. scRNA-seq assessment of CRISPR-Cas9 and HDR-enhancing compounds in DADA2 patient and HD T cells

(A) Outline of the experiment. HD and DADA2 patient PBMCs were thawed and stimulated with IL-2 (120 U/mL), IL-7 (3 ng/μL), IL-15 (3 ng/μL), and soluble CD3/CD28 (15 μL/mL) on day 1 and nucleofected on day 4 with ADA2 CRISPR RNPs or mock. Cells were cultured in IL-2 (250 U/mL) and HDR enhancers (0.5 μM KU0060648, 0.6 μM IDT Alt-R enhancer V2) or DMSO for 24 h after nucleofection and IL-2 alone afterward. On day 8, 64 $CD4^+$ and 64 $CD8^+$ T cells per condition (128 cells in total per condition) were sorted into 384-well plates, and gDNA was extracted from the bulk for ddPCR. Sorted cells were further analyzed with RT-qPCR and scRNA-seq. (B) *ADA2* HDR editing in HD and DADA2 patients on day 8, assessed by ddPCR ($n = 3$ technical replicates). (C) ADA2 editing in HD and DADA2 patients, assessed by RT-qPCR of the scRNA-seq libraries with probes to the corrected and uncorrected nucleotide sequence. For HD, 56, 75, and 77 cells were analyzed for DMSO, KU0060648, and IDT Alt-R enhancer V2-treated cells, respectively. For DADA2 patients, 39, 50, and 50 cells were analyzed for DMSO, KU0060648, and IDT Alt-R enhancer V2-treated cells, respectively. Uniform manifold approximation and projection (UMAP) plots generated from scRNA-seq for *ADA2*-edited HD treated with (D) DMSO, (E) KU0060648, and (F) IDT Alt-R enhancer V2, compared to unedited HD (DMSO). UMAP plots of corrected DADA2 patient treated with (G) DMSO, (H) KU0060648 and (I) IDT Alt-R enhancer V2,

(legend continued on next page)

To conclude, long-read sequencing and single-cell transcriptomics demonstrate that genome-edited healthy control and DADA2 patient T cells cultured with or without NHEJ inhibitors do not display identifiable structural variations, transcriptome, and T cell receptor repertoire.

Functional consequences of *ADA2* correction on the transcriptome and proteome

DADA2 is a complex autoinflammatory disease with multiple affected immune subsets, including T cells.[59] The disease hallmark is enhanced interferon-γ (IFN-γ) and tumor necrosis factor α (TNF-α) signaling. We compared T cell transcriptomes in unedited DADA2 patients and healthy controls. We found that they clustered separately and noted enhanced TNF-α signaling in the DADA2 patient (Figures S13A–S13D), suggesting that T cells can, with limitations, be used to model the disease pathology. Somewhat unexpectedly, patient T cell transcriptomes also indicated downregulation of IFN-α and IFN-γ responses.

As expected, 4 days after *ADA2* correction, we saw downregulation of TNF-α signaling in samples corrected in the presence of DMSO or KU0060648. The effects were not visible in cells corrected with IDT Alt-R, possibly due to the compound interfering with immune signaling pathways as seen in the healthy control (Figures 5J and 5K). The corrected DADA2 T cell transcriptomes continued to cluster with uncorrected cells. The corrected cells will likely need longer culture and re-stimulation with appropriate cytokines to show a noticeable shift toward a "healthy" T cell state.

To finalize safety and functional profiling, we analyzed the proteomes of edited and unedited DADA2 and healthy control T cells by mass spectrometry (MS) (Tables S18, S19, and S20). Cells were collected 7 days post-nucleofection, and genomic editing was confirmed with ddPCR (Figures 6A, 6B, and S14A). We saw low but detectable ADA2 expression in patients when all MS data-independent acquisition (DIA) runs were searched together (Figure 6C; Table S19); however, when searched alone, no ADA2 was detected, suggesting very low to no ADA2 expression in the patients. Editing increased ADA2 expression up to 2-fold in corrected DADA2 T cells (Figures 6C–6F). In healthy controls, ADA2 expression generally decreased upon editing, either due to on-target NHEJ deletions or the addition of silent SNVs (Figure S14B).

Other than the changes in ADA2 expression, we found no significant proteomic alterations in samples edited without enhancers (Figures 6D and S14C). In samples edited with HDR enhancers, gene set enrichment analysis[60,61] identified minor alterations without clear clustering to pathways (Figures 6E, 6F, S14D, and S14E). IDT Alt-R-treated, *ADA2*-edited healthy control cells showed more altered proteins (Figure S14E; Table S19), which we did not investigate further as the identity of the compound is undisclosed.

When comparing unedited DADA2 patients and healthy controls, DADA2 patients showed downregulation of several proteins implicated in inflammatory response, as well as decreased expression of the mRNA decapping enzyme NUTD16 (Figure 6G; Tables S20 and S21). Consequently, the proteins of the translational machinery were upregulated, along with several adaptive immune response proteins (Table S21). We also detected cytoplasmic immunoglobulins, which we attribute to residual B cells in the samples, as we saw no immunoglobulin transcripts in the scRNA-seq data where T cells were pre-sorted using flow cytometry.

To conclude, we observed ADA2 protein expression and downregulation of TNF-α signaling in corrected DADA2 patient proteomes and transcriptomes, with minimal persisting interference from the KU0060648 compound.

Gene correction improves T cell proliferation in CHH

Mutations in *RMRP* cause CHH, a syndromic immunodeficiency with defective T cell proliferation.[62] We thus evaluated the patient T cell proliferative capacity in response to mutation correction. We further hypothesized that corrected patient T cells would outgrow their uncorrected counterparts, and consequently the frequency of corrected alleles would increase in DNA samples taken during prolonged CHH T cell culture.

To test this, we first corrected *RMRP* in T cells from three CHH patients and measured HDR correction levels at 4, 7, and 14 days post-nucleofection by amplicon sequencing (Figure 7A). We noted an up to 50% correction at day 4, which increased to 70% at 14 days post-nucleofection, with individual variation and diverse representation of small indels in the samples (Figures S15–S17). Consequently, we chose to assess T cell proliferative capacity 14 days post-nucleofection and enhance *RMRP* correction by treating cells with KU0060648 for the first 24 h after nucleofection, as we observed no increased toxicity from NHEJ inhibition (Figure S4K). We performed carboxyfluorescein succinimidyl ester (CFSE)-based T cell proliferation assay in four corrected and uncorrected CHH patients 14 days after nucleofection (day 20 in cell culture) (Figure 7B). We also assessed a healthy control $CD4^+$ and $CD8^+$ T cells 14 days after mock nucleofection from the same experimental pipeline as a technical positive control for the assay (Figures S18A and S18B). Unstimulated PBMCs were used as a technical negative control for $CD4^+$ and $CD8^+$ T cell proliferation (Figures S18C and S18D). We saw significant improvement in proliferation of corrected $CD4^+$ T cells compared to that of uncorrected cells in all four patients (Figures 7C and S18E). Similarly, we saw significant improvement

compared to uncorrected DADA2 patient (DMSO). (J) Hallmark gene set enrichment results for *ADA2*-edited HD (DMSO, KU0060648, and IDT Alt-R enhancer V2) compared to unedited HD (DMSO). (K) Hallmark gene set enrichment results for corrected DADA2 patient (DMSO, KU0060648, and IDT Alt-R enhancer V2) compared to uncorrected DADA2 patient. One independent experiment was performed for all sets of data. Bar denotes mean value, error bars represent ±SD. Statistical significance for HDR editing in (B) was assessed by one-way ANOVA with Fisher's LSD test, where ****$p < 0.0001$. NES, normalized enrichment score.

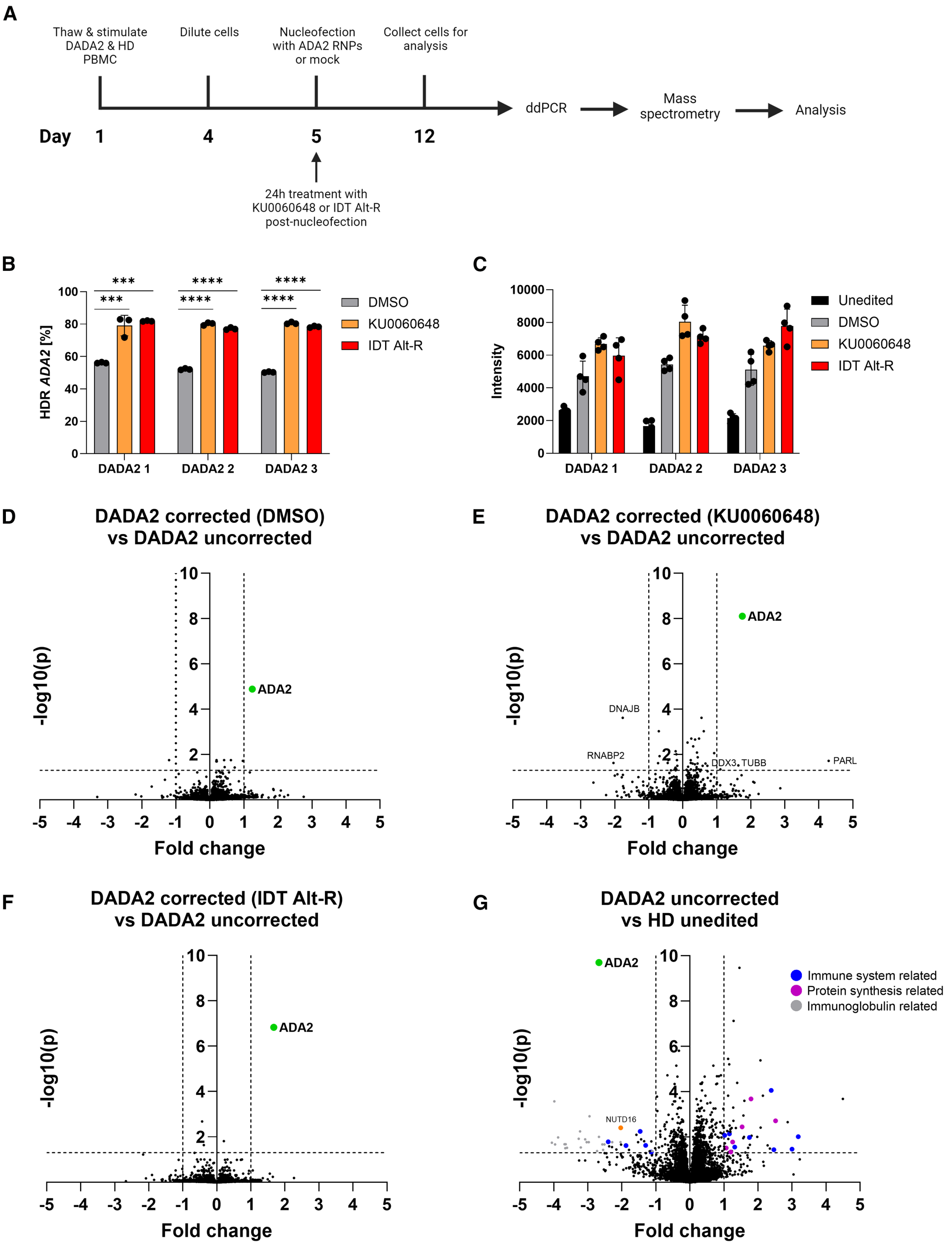

Figure 6. Mass spectrometry analysis of corrected and uncorrected DADA2 patient T cells

(A) Outline of the experiment. DADA2 patient and HD PBMCs were thawed and stimulated with IL-2 (120 U/mL), IL-7 (3 ng/μL), IL-15 (3 ng/μL), and soluble CD3/CD28 (15 μL/mL) on day 1 and diluted on day 4 for further expansion. Cells were nucleofected with ADA2 CRISPR RNPs or mock on day 5 and cultured in IL-2 (250 U/mL) and HDR enhancers (0.5 μM KU0060648 and 0.6 μM IDT Alt-R enhancer V2) or DMSO for 24 h. Afterward, cells were cultured in IL-2 (250 U/mL) until sample collection on day 12. (B) *ADA2* HDR editing in three DADA2 patients (DADA2 1–3) treated with HDR enhancers or DMSO, assessed by ddPCR ($n = 3$ technical replicates). (C) Abundance of

(legend continued on next page)

in CD8$^+$ T cell proliferation upon mutation correction in all but one patient (Figures 7D and S18F). In conclusion, genomic correction of *RMRP* enhances T cell proliferation, leading to selective growth advantage for the corrected cells.

Gene correction reduces STAT1 hyperphosphorylation in STAT1-GOF patients

Dominant activating *STAT1* mutations cause a defect in T cell function, which presents as increased susceptibility to fungal and viral infections and autoimmunity.[63–65] We thus hypothesized that correction of *STAT1* would reduce STAT1 hyperactivation in stimulated T cells. To correct an activating *STAT1* p.388R mutation, we first designed CRISPR reagents as described (Figures 1 and 3). Of the three available guides surrounding the mutation site (Figure 8A), we identified gRNA number 2 and symmetric repair template as the best combination because gRNA number 2 is mutation specific and does not cut the WT allele (Figures 8A–8C). When correcting patient cells with the optimized platform, we noted up to 40% total HDR, which translates to 80% diseased allele correction because the mutation is heterozygous (Figure 8D).

Activating STAT1 mutations lead to STAT1 hyperphosphorylation in stimulated cells.[66,67] Consistently, we saw increased pSTAT1 in patient T cells that were stimulated with IFN-α. The phosphorylation decreased in the patient upon gene correction (Figure 8E). Without stimulation, STAT1 phosphorylation was not observed in uncorrected patient T cells, and consequently, correction did not affect resting pSTAT1 levels in our patient. We conclude that gene correction can reduce excessive STAT1 activation, and that our platform is effective in correcting heterozygous mutations.

DISCUSSION

In this study, we developed a CRISPR-Cas9-based T cell gene correction platform for monogenic IEIs. We demonstrate up to 80% mutation correction efficiency and functional improvement in the model IEIs. The platform is suitable for correcting diverse SNVs and small indels in multiple genes and is portable for clinical translation. Corrected autologous T cell transplants can further be developed into a salvage therapy for IEI patients with isolated T cell defects.[4,7,10–13]

In this study, we have optimized the T cell editing platform in six endogenous loci (*ADA2*, *AIRE*, *CTCF-1*, *Enh4-1*, *RMRP*, and *RNF2*) in healthy controls and further assessed the functional impact of mutation correction in DADA2, CHH, and STAT1-GOF patients where the peripheral T cells contribute to disease phenotype. While gene editing of peripheral T cells is not expected to offer therapeutic benefits for APECED, which primarily affects the thymic medullary epithelial cells,[68] the T cells from these patients were used for method/platform development primarily due to practical considerations regarding access to sizable patient cohorts. In DADA2, we observed restored ADA2 protein expression and reduced TNF-α signaling in patient T cells following mutation correction. In CHH, the correction improved the proliferation defect observed in patient T cells. In STAT1-GOF patients, the correction reduced STAT1 hyperphosphorylation into a normal level. Successful SNV editing in six distinct loci along with observed functional impact of SNV correction in three IEIs highlights the versatility of this platform to serve as a universal approach for a wide range of monogenic T cell defects. However, thorough assessment of preclinical efficacy and safety of the strategies presented here are required before further clinical translation. Donor-to-donor variability among healthy controls limits threshold definition in this study. Future work will include larger cohorts to establish normal ranges and support clinical applications in diseases such as CHH and STAT1-GOF. Furthermore, we advise that each model be assessed separately for its potential clinical impact.

Our correction approach requires the presence of T cells that can proliferate, which excludes certain severe combined immunodeficiencies where T cells are absent or do not proliferate. In conditions where T cells exist but have little proliferation, cell-cycle-independent correction methods such as base and prime editing can be better alternatives. If poor proliferation is due to gene defects in cytokine signaling, then adjustments to the presented stimulation protocol can improve correction levels.

CRISPR-Cas9 cutting can lead to off-target cuts. We found no off-targets with GUIDE-seq profiling for the selected gRNAs. Alternative methods such as circularization for *in vitro* reporting of cleavage effects by sequencing, cellular indexing of transcriptomes and epitopes by sequencing, and circularization for high-throughput analysis of nuclease genome-wide effects by sequencing exist and all have their own advantages and limitations.[69] In addition, CRISPR can induce structural chromosomal changes at the target site.[56,70–73] The structural variants increase with rapid cell proliferation, and optimized culture conditions can decrease the events.[56] The use of DNA-PKcs inhibition[74,75] was recently reported to increase on-target chromosome loss.[76] Although we did not find persisting genomic aberrations, we cannot exclude the possibility that low-frequency on- and off-target structural variants remain undetected due to technology constraints. The cells with larger abnormalities can also become arrested and disappear below detection limit by the assay time point.[56,73] Time point experiments with extended cell

ADA2 protein in DADA2 patients, reported as intensities (n = 4 technical replicates). Comparison of protein expression levels in (D) *ADA2*-corrected (DMSO) DADA2 patients to uncorrected DADA2 patients, (E) *ADA2*-corrected (KU0060648) DADA2 patients to uncorrected DADA2 patients, (F) *ADA2*-corrected (IDT Alt-R enhancer V2) DADA2 patients to uncorrected DADA2 patients, and (G) uncorrected DADA2 patients to unedited HDs, assessed by mass spectrometry. For (D)–(G), volcano plots were created by reporting protein expression fold change from mean of three DADA2 patients and three HDs on the x axis and −log10 p value on the y axis. One independent experiment was performed for all sets of data. Statistical significance was assessed by one-way ANOVA with Fisher's LSD test, where *$p < 0.05$, **$p < 0.01$, ***$p < 0.0002$, and****$p < 0.0001$. Bar denotes mean value, error bars represent ±SD. DDX3, ATP-dependent RNA helicase; DNAJB, DnaJ homolog subfamily B; NUDT, U8 snoRNA-decapping enzyme; PARL, presenilin-associated rhomboid-like protein; RNABP2, E3 SUMO-protein ligase RanBP2; TUBB, tubulin beta.

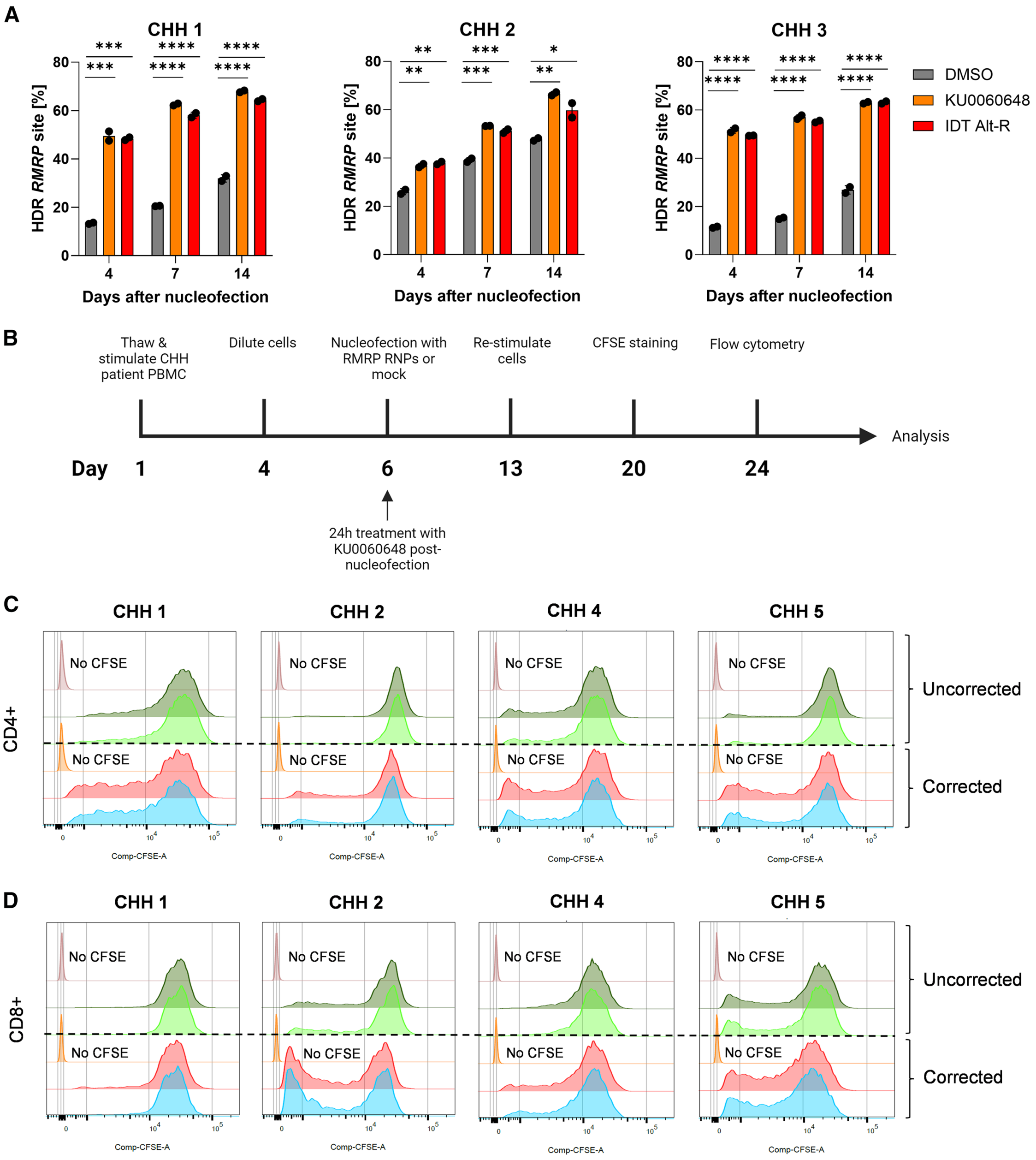

Figure 7. T cell proliferation assay in cartilage-hair hypoplasia patients

(A) *RMRP* HDR editing in three cartilage-hair hypoplasia (CHH) patients (CHH 1–3) 4, 7, and 14 days after nucleofection with concentration-optimized HDR enhancing compounds (0.5 μM KU0060648 and 0.6 μM IDT Alt-R enhancer V2) or DMSO. HDR was assessed by amplicon sequencing (n = 2 technical replicates). (B) Outline of the CFSE-based T cell proliferation experiment. CHH patient PBMCs were thawed and stimulated with IL-2 (120 U/mL), IL-7 (3 ng/μL), IL-15 (3 ng/μL), and soluble CD3/CD28 (15 μL/mL) on day 1 and diluted on day 4 for further expansion. Cells were nucleofected with CRISPR RNPs for RMRP correction or mock on day 6 and cultured in IL-2 (250 U/mL) and 0.5 μM KU0060648 for 24 h after nucleofection. Afterward, cells were cultured in IL-2 (250 U/mL) until re-stimulation on day 13 with the same setup as on day 1. Cells were stained with CFSE on day 20 and cultured in IL-2 (250 U/mL) for 4 days. On day 24, cells were stained for flow cytometry. T cell proliferation in corrected and uncorrected CHH patients for (C) $CD4^+$ and (D) $CD8^+$ T cells, assessed by flow cytometry. One independent experiment was performed for all sets of data. The patient number corresponds to patient information in Table S15. Bar denotes mean value, error bars represent ±SD. Statistical significance was assessed by one-way ANOVA with Fisher's LSD test, where $^*p < 0.05$, $^{**}p < 0.01$, $^{***}p < 0.0002$, and $^{****}p < 0.0001$.

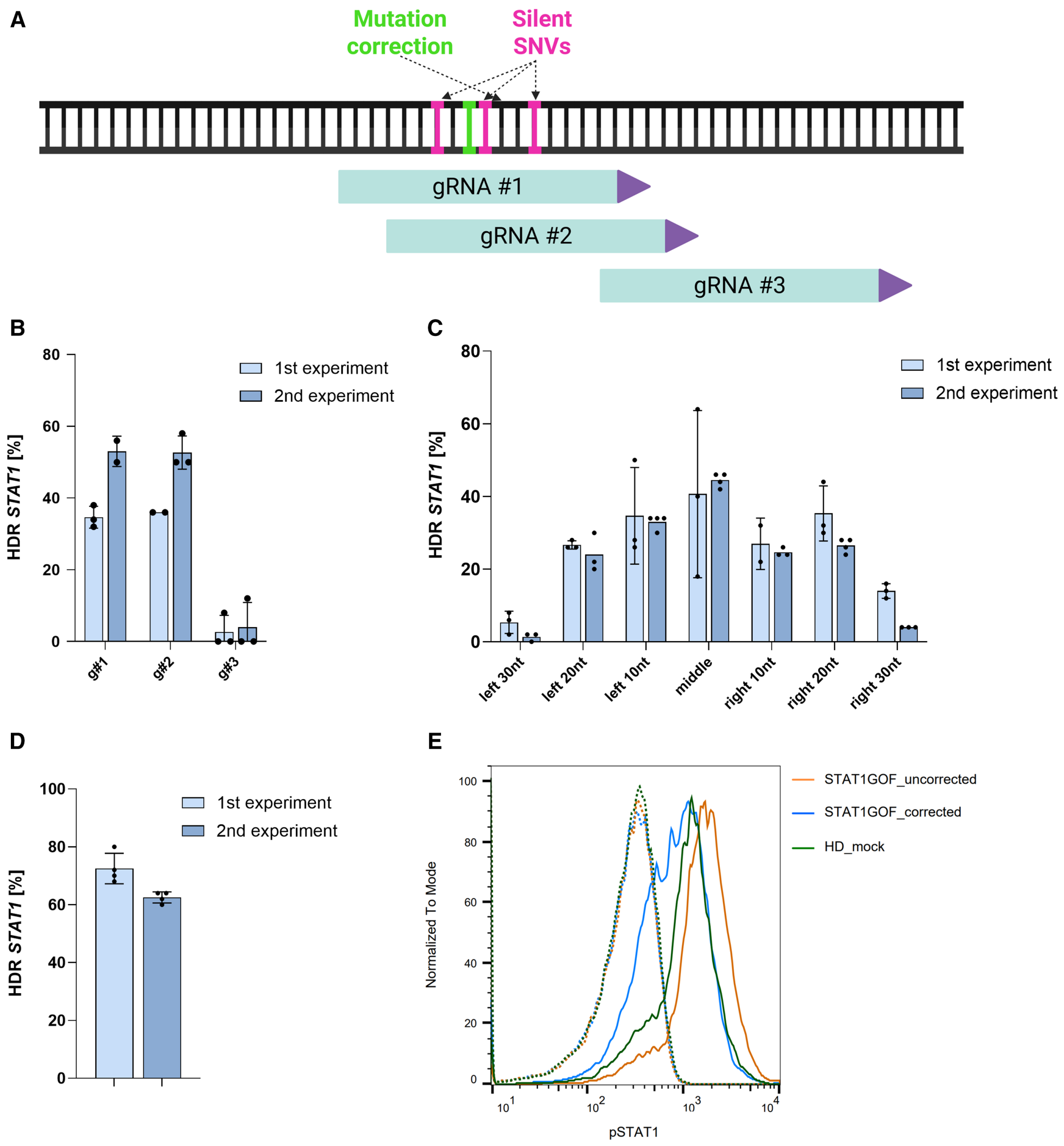

Figure 8. Assessment of STAT1 phosphorylation in corrected and uncorrected STAT1-GOF patients

(A) Schematic representation STAT1 gRNA design and repair strategy. Correction of pathogenic mutation is marked with green and silent SNVs in pink. Three gRNAs were designed (green), where the PAM site is represented as an arrow (purple). (B) STAT1 gRNA screening in STAT1-GOF patient T cells assessed by measuring HDR editing using ddPCR (n = 3 technical replicates) in two independent experiments, indicated in light and dark blue. (C) 3′ PT modified asymmetric ssODNs screening with best-performing guide (g number 2) in STAT1-GOF patient T cells assessed by measuring HDR editing using ddPCR (n = 3 technical replicates) in two independent experiments, indicated in light and dark blue. (D) *STAT1* HDR editing in T cells from STAT1-GOF patient 7 days after nucleofection with optimized RNP was assessed by ddPCR (n = 2 technical replicates) in two independent experiments, indicated in light and dark blue. The ddPCR readouts in (B)–(D) are reported as twice the measured value as the mutation is heterozygous and uncorrected allele is present at the time of assessment. (E) The cells obtained on 7 days post-nucleofection from the second experiment in (D) were also stimulated with IFN-α, followed by assessment of phosphorylated STAT1 levels in $CD3^{+}$ cells using flow cytometry. Mock electroporated patient T cells and healthy donor T cells that did not receive CRISPR RNPs were used as controls. The dotted line and the solid line show unstimulated and stimulated samples, respectively.

culture, along with *in vivo* xenotransplant studies, can complement the safety assessment and help to evaluate the long-term T cell survival and malignant transformation risk.

Our correction strategy introduces 2–4 silent SNVs along with correction of the pathogenic variant. The strategy prevents CRISPR re-cutting after successful HDR repair and improves precise

correction levels.[34,45] Additionally, it allows accurate and rapid editing quantification by droplet digital PCR. To ensure minimal interference with gene function and regulation, we advise prioritizing SNVs that are part of normal human variation and located in evolutionarily less conserved regions. We also advise that the functional effect of the SNVs be assessed case by case, as certain silent SNVs can disrupt mRNA transcription and protein translation.[77–79]

In conclusion, we present a non-viral T cell SNV correction platform that has the potential to be scaled up to a translationally relevant platform to correct diverse pathogenic SNVs, small deletions, and insertions in IEIs.

MATERIALS AND METHODS

The aim of this study was to develop a CRISPR-Cas9-based T cell platform for mutation correction in IEI patients. We used the following Finnish founder diseases as models: DADA2, APECED, and CHH. We obtained PB, cord blood, and skin biopsies from patients or healthy controls. Detailed information on the patients is provided in Table S15. The study was conducted in accordance with the principles of the Declaration of Helsinki and approved by the Helsinki University Central Hospital Ethics Committee and the Regional Committee for Medical and Health Research Ethics South-East Norway. Participants have signed written informed consent forms.

Isolation, culture, and nucleofection of T cells, $CD34^+$ HSPCs, and fibroblasts

PBMCs were isolated from PB using Ficoll gradient centrifugation and cryopreserved. Upon thawing, PBMCs were cultured in ImmunoCult-XF T Cell Expansion Medium supplemented with IL-2, IL-7, IL-15, and CD3/CD28 T cell activator. After 3 nights at 37°C/5% CO_2, cells were nucleofected or further cultured without the CD3/CD28 activator. $CD34^+$ HSPCs were isolated from cord blood using the CD34 MicroBead Kit and cryopreserved. Upon thawing, HSPCs were cultured in StemSpan SFEM II supplemented with GlutaMax, Flt3-L, thrombopoietin, stem cell factor, IL-6, StemRegenin-1, and UM729. After 3 nights at 37°C/5% CO_2, cells were nucleofected or further cultured. Fibroblasts isolated from skin biopsies were expanded in DMEM with low glucose, pyruvate, and FBS and cryopreserved. Upon thawing, fibroblasts were cultured until confluent, passaged every 3–4 days with TrypLE Express Enzyme, and nucleofected by passage 10.

T cells, $CD34^+$ HSPCs, and fibroblasts were nucleofected using a 4-D Nucleofector system and a 96-well unit (Lonza). gRNAs were prepared by annealing CRISPR RNA and *trans*-activating CRISPR RNA (IDT) and mixed with Cas9 nuclease and ssODN (IDT) to form RNPs. T cells (0.5 million or 1 million), HSPCs (0.3 million), and fibroblasts (1 million) were resuspended in 20 μL electroporation buffer and nucleofected using programs EO-115, DZ-100, and CA-137, respectively. Post-nucleofection, T cells, HSPCs and fibroblasts were incubated with their respective recovery media for 15 min, transferred to plates, and cultured until collection after 4–8 days. For details, see the supplemental methods.

Design and screening of CRISPR-Cas9 reagents

From 7 to 18 gRNAs were designed based on available PAM (NGG) centering the mutation site. ssODNs of 100 bp were designed with ±50-bp homology arms from the mutation site. Synonymous, silent SNVs were added in repair templates for *ADA2* (four SNVs) and *AIRE* (three SNVs) to prevent CRISPR re-cutting and ensure identical editing in donors and patients. As *RMRP* is a non-coding gene, SNVs were used in early experiments, and only mutation correction later in functional assessments. Asymmetric ssODNs with 10- to 40-bp homology arms were tested to enhance HDR. Details about gRNA (Table S2) and ssODN design (Table S3), BG-coupled ssODNs, and Cas9-SNAP protein production can be found in the supplemental methods.

On-target editing assessment

ddPCR assays were performed to assess HDR and NHEJ editing. Previously described oligos[41] were used to edit *Enh4-1*, *CTCF1*, and *RNF2*, while new oligos for *ADA2*, *AIRE*, and *RMRP* were designed. ddPCR was performed using the QX200 system (Bio-Rad) and analyzed with QuantaSoft software (Bio-Rad). The oligos are listed in Table S8.

Amplicon sequencing libraries were prepared from gDNA samples using a two-step PCR method.[41] Unique molecular identifiers were added to the primers to filter out PCR bias.[41] Libraries were sequenced using the Illumina MiSeq version 2 platform. Data analysis was done using the ampliCan software package.[80] Amplicon sequencing PCR and oligos are listed in Tables S9–S11.

Assessment of *in silico* gRNA design tools

We assessed the predictive power of *in silico* gRNA design tools against *in vitro* gRNA screening data using the following tools: Atum, Benchling, CHOPCHOP, CRISPOR, DeepSpCas9, EuPaGDT, and the IDT gRNA design tool. Using 100-bp mutant-specific sequences with 50-bp homology arms as input, we selected the three highest predicted efficiency gRNAs from each tool. These were then compared against the three best *in vitro*-validated gRNAs from patient T cells. Details of the *in silico* tools are listed in the supplemental methods.

Screening HDR enhancers and cell-cycle inhibitors in healthy control T cells

A total of 33 HDR enhancers and 10 cell-cycle inhibitors (Tables S12 and S13) were screened in healthy donor (HD) T cells at 3 concentrations against RNP-edited cells (DMSO). For HDR enhancers, 0.5 million T cells per sample were nucleofected and incubated, with the compounds in T cell recovery medium for 24 h, then split 1:1 in recovery medium without compounds 24 and 72 h after nucleofection. The toxicity of HDR enhancers was assessed using the CellTiter-Glo assay (Promega) according to the manufacturer's instructions (see details in the supplemental methods). For cell-cycle

inhibitors, cells were either pre-treated with the compounds for 24 h before or 24 h after nucleofection. In both cases, 0.5 million cells per sample were nucleofected and split 1:1 in recovery medium without compounds 24 and 72 h after nucleofection. Samples for both screens were collected 96 h after nucleofection for gDNA extraction and ddPCR.

Off-target editing assessment

The previously published GUIDE-seq method[53] was used to assess off-target editing (see details in the supplemental methods). In brief, 1 million T cells per sample were nucleofected on day 5 with RNPs (5 μmol/L/sample gRNA, 3.05 μmol/L/sample Cas9 nuclease, 1.5 μmol/L/sample dsODN). Samples were collected for library preparation, sequencing, and ddPCR 4 days later. Data analysis was performed following the GUIDE-seq analysis pipeline from Zhu et al.,[81] but adjusted for allowing bulges between single-guide RNA (sgRNA) and off-target sites with editing distance of 4 with the use of CHOP-OFF.[82] Final off-targets were normalized against control data (transfected with dsODN only). We used custom scripts available at https://git.app.uib.no/valenlab/t_cell_editing_pipeline/.

PacBio sequencing of CRISPR-edited healthy control T cells

Healthy control T cells were edited as described above and treated with 0.5 μM KU0060648 or DMSO. Six days post-editing, DNA was extracted from 5 million cells per sample using Qiagen kits. DNA quality was assessed using NanoDrop, Qubit, and agarose gel electrophoresis. Libraries for PacBio HiFi sequencing were prepared using the Revio HiFi Prep Kit and Sequencing Chemistry version 2.0. Sequencing data were demultiplexed with SMRT Link, and circular consensus sequence reads were generated and further demultiplexed using barcoded primers, with HiFi reads indexed by barcode IDs. The HiFi sequencing reads were aligned with pbmm2 version 1.13.0. Structural variants were called with pbsv version 2.9.0 and small variants with deepVariant version 1.6.0. All possible mismatches, deletions, and insertions were extracted from aligned reads using custom scripts (https://git.app.uib.no/valenlab/t_cell_editing_pipeline/-/tree/main/katariina_pacbio). We normalized data using two control samples and focused on sites that were potential sgRNA off-target within distance of 4, allowing for bulges.

Immunophenotyping by flow cytometry

PBMC samples from days 1, 4, and 8 of the platform were assessed using flow cytometry. Cells (0.5 million per sample) were washed with flow cytometry buffer, blocked with 10% human serum, and stained with an antibody cocktail (Table S4) to identify CD4 T cells, CD8 T cells, B cells, natural killer (NK) cells, monocytes, and dendritic cells. After washing, cells were resuspended in 250 μL flow cytometry buffer and stored at 4°C. Flow cytometry was done on LSRII and data analysis was done using FlowJo. For details, see the supplemental methods.

T cell proliferation assay in CHH patients

T cells from CHH patients from day 20 of the platform were collected, washed with PBS, and resuspended at 2 million cells/mL. Cells were stained with 1 μM CFSE and incubated in the dark at 37°C for 5 min. Cold human serum was added to quench the reaction. Cells were then washed and resuspended in Immunocult medium supplemented with 250 U/mL IL-2 at 0.2 million cells per well in a 96-well U-bottom plate. After 4 days, cells were stained with an antibody cocktail (Table S6) and analyzed by flow cytometry as described previously. For details, see the supplemental methods.

Assessment of STAT1 phosphorylation in STAT1-GOF patients

T cells from corrected and uncorrected STAT1-GOF patients were collected 4 days after nucleofection, washed with PBS, and resuspended at 1 million cells/sample. Cells were stained in the dark at 4°C for 30 min with Live/Dead dye and FcR Blocking Reagent, after which cells were stimulated with 250 μL of the 2×10^3 U/mL of the IFN-α in Immunocult medium and the cocktail of cell surface antibodies (Table S7). The unstimulated controls received only 250 μL medium and the same antibody cocktail. Cells were incubated at 37°C for 30 min in the dark, with shaking every 5 min. Immediately after, 2 mL freshly prepared 1:5 Phosflow Lyse/Fix Buffer was added to the samples, which were shortly vortexed before incubating at 37°C for 10 min, with shaking every 3 min. After incubation, samples were washed and centrifuged, and 500 μL cold Phosflow PermBuffer III was added, followed by incubation on ice for 30 min in the dark. Cells were washed with flow buffer and stained with 1:10 dilution of pSTAT1 antibody, followed by 30 min incubation at room temperature in the dark. Afterward, cells were washed two times with flow buffer, resuspended in flow buffer, and stored in a refrigerator overnight for flow cytometry analysis the day after. For details, see the supplemental methods.

DNRT scRNA-seq and RT-qPCR of control and DADA2 patient T cells

A previously published Smart-Seq2-based direct nuclear tagmentation and RNA-seq (DNRT) protocol was used.[83] For details, see the supplemental methods. In brief, on day 8, nucleofected HD and DADA2 patient T cells were collected, washed, and stained with Live/Dead dye and Fc blocking reagent. After washing, cells were stained with antibody cocktail (Table S5), washed and resuspended in flow buffer. Live CD4+ and CD8+ T cells were sorted into 384-well plates with lysis buffer. After sorting, plates were centrifuged, snap-frozen, and stored at −80°C. Using the Smart-Seq2 protocol,[83] cells were thawed, reverse transcribed, and cDNA pre-amplified, with cleanup using SPRI beads and concentration measured with the Qubit DNA HS kit (Table S14). Tagmentation of diluted cDNA was followed by SDS reaction stop, barcoding, and PCR. Libraries were cleaned with SPRI beads and sequenced on a Novaseq 6000.

For data analysis, the reads were trimmed with Cutadapt[84] and aligned to hg38 with STAR.[85] Picard[86] removed duplicates, and HTSeq[87] summarized counts. Cells with <20,000 reads, <500 features, or low ACTB expression were filtered out. Seurat[88] version 5.0.1 log-normalized data identified 2,000 variable features and scaled data per condition. FindMarkers in Seurat identified markers

between conditions, and fgsea[89] performed gene set enrichment analysis. Fusion gene detection was performed with STAR-Fusion. Loss of heterozygosity calculations were performed as described in the supplemental methods. For quantitative analysis of different alleles in single cells, 1 μL diluted cDNA was amplified with specific probes for WT and edited alleles (see details in the supplemental methods). RT-qPCR analysis used Bio-Rad software with a 200 relative fluorescence units as a threshold for determining which allele was being expressed.

MS

For details, see the supplemental methods. In brief, T cells from three DADA2 patients and HDs were cultured with 1 million cells per sample and nucleofected on day 5. Mock-nucleofected cells were treated with DMSO, and edited cells with 0.5 μM KU0060648, 0.6 μM IDT Alt-R enhancer V2, or DMSO for 24 h. Cells were collected on day 12, washed, pelleted, and snap-frozen on liquid nitrogen.

For MS, trypsin/LysC digested samples were diluted 1:60 in 0.1% formic acid in water, and 20 μL was loaded into an Evotip. Samples were analyzed using the Evosep One system with the Bruker timsTOF Pro mass spectrometer. Peptide separation used an 8 cm × 150 μm column with a 21-min gradient. Data were processed with DIA-NN version 1.8.1[90,91] using the UniProt human proteome spectral library, with fixed and variable modifications. Pre-processing involved log2 transformation, median-normalization, and QRILC imputation (https://cran.r-project.org/web/packages/imputeLCMD/imputeLCMD.pdf). Statistical analysis used Student's t test[92] and the Benjamini-Hochberg method[93] for *p* value adjustment. The volcano plots were generated using bioinfokit.

DATA AVAILABILITY

The data can be found in Tables S1–S14, Figures S1–S18, and Tables S15, S16, S17, S18, S19, S20, and S21. Raw GUIDE-seq, scRNA-seq, MS and PacBio whole-genome sequencing data will be deposited in a secure repository after publication.

ACKNOWLEDGMENTS

We thank all patients and families for their participation in the study. We thank Karolinska Institute Protein Science Facility for manufacturing the Cas9 protein and Riitta Lehtinen for her expert technical assistance. The Research Council of Norway, Health South-East Region, the Swedish Childhood Cancer Society (Barncancerfonden), and the Norwegian Cancer Society supported this work. This work was partially supported by the Research Council of Norway through its Centers of Excellence scheme (project number 332727).

AUTHOR CONTRIBUTIONS

K.M. performed most of the experiments and wrote the manuscript. S. Kolbeinsdottir and M.E. performed the scRNA-seq experiments. Z.L. designed the CRISPR reagents and performed the GUIDE-seq optimization. K.L., E.T., and E.V. performed the bioinformatics and data analysis. A.K. performed the GUIDE-seq and PacBio experiments. S. Keskitalo, A.T., and M.V. performed the MS experiments and data analysis. G.R. designed the CRISPR reagents and the amplicon sequencing panel and performed the experiments. F.H.H. performed the gRNA screening and BG-coupled ssODN experiments. B.O.L. performed the CRISPR optimization experiments in T cells. S.S.J. performed gRNA screening and the STAT1-GOF functional assessment. T.J.G. performed flow cytometry experiments. C.W.E. and P.K. performed gRNA screening in $CD34^+$ HSPCs. N.F. and M.S. performed library preparation for amplicon sequencing. T.M.M. obtained cord blood for HSPC isolation. J.S. and J.O. supervised the experiments. V.G., E.L., C.S.-J., T.H., K.H.B.M., H.C.E., and T.M. provided clinical care for the patients and obtained samples. S.D.-K. designed the scRNA-seq, flow cytometry and cell sorting experiments; performed and supervised the research; and wrote the manuscript. E.H. supervised the study and wrote the manuscript. All authors read and approved the manuscript.

DECLARATION OF INTERESTS

Authors declare no competing interests.

SUPPLEMENTAL INFORMATION

Supplemental information can be found online at https://doi.org/10.1016/j.ymthe.2025.08.018.

REFERENCES

1. Akalu, Y.T., and Bogunovic, D. (2024). Inborn errors of immunity: an expanding universe of disease and genetic architecture. Nat. Rev. Genet. *25*, 184–195. https://doi.org/10.1038/s41576-023-00656-z.
2. Tangye, S.G., Al-Herz, W., Bousfiha, A., Cunningham-Rundles, C., Franco, J.L., Holland, S.M., Klein, C., Morio, T., Oksenhendler, E., Picard, C., et al. (2022). Human Inborn Errors of Immunity: 2022 Update on the Classification from the International Union of Immunological Societies Expert Committee. J. Clin. Immunol. *42*, 1473–1507. https://doi.org/10.1007/s10875-022-01289-3.
3. Albert, M.H., Sirait, T., Eikema, D.J., Bakunina, K., Wehr, C., Suarez, F., Fox, M.L., Mahlaoui, N., Gennery, A.R., Lankester, A.C., et al. (2022). Hematopoietic stem cell transplantation for adolescents and adults with inborn errors of immunity: an EBMT IEWP study. Blood *140*, 1635–1649. https://doi.org/10.1182/blood.2022015506.
4. Harris, K.M., Davila, B.J., Bollard, C.M., and Keller, M.D. (2019). Virus-Specific T Cells: Current and Future Use in Primary Immunodeficiency Disorders. J. Allergy Clin. Immunol. Pract. *7*, 809–818. https://doi.org/10.1016/j.jaip.2018.10.049.
5. Burns, S.O., and Morris, E.C. (2021). How I use allogeneic HSCT for adults with inborn errors of immunity. Blood *138*, 1666–1676. https://doi.org/10.1182/blood.2020008187.
6. Lankester, A.C., Albert, M.H., Booth, C., Gennery, A.R., Güngör, T., Hönig, M., Morris, E.C., Moshous, D., Neven, B., Schulz, A., et al. (2021). EBMT/ESID inborn errors working party guidelines for hematopoietic stem cell transplantation for inborn errors of immunity. Bone Marrow Transplant. *56*, 2052–2062. https://doi.org/10.1038/s41409-021-01378-8.
7. Hubbard, N., Hagin, D., Sommer, K., Song, Y., Khan, I., Clough, C., Ochs, H.D., Rawlings, D.J., Scharenberg, A.M., and Torgerson, T.R. (2016). Targeted gene editing restores regulated CD40L function in X-linked hyper-IgM syndrome. Blood *127*, 2513–2522. https://doi.org/10.1182/blood-2015-11-683235.
8. Hou, T.Z., Qureshi, O.S., Wang, C.J., Baker, J., Young, S.P., Walker, L.S.K., and Sansom, D.M. (2015). A transendocytosis model of CTLA-4 function predicts its suppressive behavior on regulatory T cells. J. Immunol. *194*, 2148–2159. https://doi.org/10.4049/jimmunol.1401876.
9. Panchal, N., Houghton, B., Diez, B., Ghosh, S., Ricciardelli, I., Thrasher, A.J., Gaspar, H.B., and Booth, C. (2018). Transfer of gene-corrected T cells corrects humoral and cytotoxic defects in patients with X-linked lymphoproliferative disease. J. Allergy Clin. Immunol. *142*, 235–245.e6. https://doi.org/10.1016/j.jaci.2018.02.053.
10. Fox, T.A., Houghton, B.C., Petersone, L., Waters, E., Edner, N.M., McKenna, A., Preham, O., Hinze, C., Williams, C., de Albuquerque, A.S., et al. (2022). Therapeutic gene editing of T cells to correct CTLA-4 insufficiency. Sci. Transl. Med. *14*, eabn5811. https://doi.org/10.1126/scitranslmed.abn5811.
11. Goodwin, M., Lee, E., Lakshmanan, U., Shipp, S., Froessl, L., Barzaghi, F., Passerini, L., Narula, M., Sheikali, A., Lee, C.M., et al. (2020). CRISPR-based gene editing enables FOXP3 gene repair in IPEX patient cells. Sci. Adv. *6*, eaaz0571. https://doi.org/10.1126/sciadv.aaz0571.
12. Houghton, B.C., Panchal, N., Haas, S.A., Chmielewski, K.O., Hildenbeutel, M., Whittaker, T., Mussolino, C., Cathomen, T., Thrasher, A.J., and Booth, C. (2022). Genome Editing With TALEN, CRISPR-Cas9 and CRISPR-Cas12a in Combination With AAV6 Homology Donor Restores T Cell Function for XLP. Front. Genome Ed. *4*, 828489. https://doi.org/10.3389/fgeed.2022.828489.

13. Vavassori, V., Mercuri, E., Marcovecchio, G.E., Castiello, M.C., Schiroli, G., Albano, L., Margulies, C., Buquicchio, F., Fontana, E., Beretta, S., et al. (2021). Modeling, optimization, and comparable efficacy of T cell and hematopoietic stem cell gene editing for treating hyper-IgM syndrome. EMBO Mol. Med. *13*, e13545. https://doi.org/10.15252/emmm.202013545.

14. Fox, T.A., Houghton, B.C., and Booth, C. (2022). Gene Edited T Cell Therapies for Inborn Errors of Immunity. Front. Genome Ed. *4*, 899294. https://doi.org/10.3389/fgeed.2022.899294.

15. Chetty, K., Houghton, B.C., and Booth, C. (2022). Gene Therapy for Inborn Errors of Immunity: Severe Combined Immunodeficiencies. Hematol. Oncol. Clin. North Am. *36*, 813–827. https://doi.org/10.1016/j.hoc.2022.03.010.

16. Panchal, N., Ghosh, S., and Booth, C. (2021). T cell gene therapy to treat immunodeficiency. Br. J. Haematol. *192*, 433–443. https://doi.org/10.1111/bjh.17070.

17. Nasri, M., Ritter, M., Mir, P., Dannenmann, B., Aghaallaei, N., Amend, D., Makaryan, V., Xu, Y., Fletcher, B., Bernhard, R., et al. (2020). CRISPR/Cas9-mediated ELANE knockout enables neutrophilic maturation of primary hematopoietic stem and progenitor cells and induced pluripotent stem cells of severe congenital neutropenia patients. Haematologica *105*, 598–609. https://doi.org/10.3324/haematol.2019.221804.

18. Nasri, M., Ritter, M.U., Mir, P., Dannenmann, B., Kaufmann, M.M., Arreba-Tutusaus, P., Xu, Y., Borbaran-Bravo, N., Klimiankou, M., Lengerke, C., et al. (2024). CRISPR-Cas9n-mediated ELANE promoter editing for gene therapy of severe congenital neutropenia. Mol. Ther. *32*, 1628–1642. https://doi.org/10.1016/j.ymthe.2024.03.037.

19. Castiello, M.C., Ferrari, S., and Villa, A. (2023). Correcting inborn errors of immunity: From viral mediated gene addition to gene editing. Semin. Immunol. *66*, 101731. https://doi.org/10.1016/j.smim.2023.101731.

20. Somekh, I., Hendel, A., and Somech, R. (2024). Evolution of Gene Therapy for Inborn Errors of Immunity. JAMA Pediatr. *178*, 645–646. https://doi.org/10.1001/jamapediatrics.2024.1116.

21. Gray, D.H., Villegas, I., Long, J., Santos, J., Keir, A., Abele, A., Kuo, C.Y., and Kohn, D.B. (2021). Optimizing Integration and Expression of Transgenic Bruton's Tyrosine Kinase for CRISPR-Cas9-Mediated Gene Editing of X-Linked Agammaglobulinemia. CRISPR J. *4*, 191–206. https://doi.org/10.1089/crispr.2020.0080.

22. Rai, R., Romito, M., Rivers, E., Turchiano, G., Blattner, G., Vetharoy, W., Ladon, D., Andrieux, G., Zhang, F., Zinicola, M., et al. (2020). Targeted gene correction of human hematopoietic stem cells for the treatment of Wiskott - Aldrich Syndrome. Nat. Commun. *11*, 4034. https://doi.org/10.1038/s41467-020-17626-2.

23. McAuley, G.E., Yiu, G., Chang, P.C., Newby, G.A., Campo-Fernandez, B., Fitz-Gibbon, S.T., Wu, X., Kang, S.H.L., Garibay, A., Butler, J., et al. (2023). Human T cell generation is restored in CD3δ severe combined immunodeficiency through adenine base editing. Cell *186*, 1398–1416.e23. https://doi.org/10.1016/j.cell.2023.02.027.

24. Newby, G.A., and Liu, D.R. (2021). In vivo somatic cell base editing and prime editing. Mol. Ther. *29*, 3107–3124. https://doi.org/10.1016/j.ymthe.2021.09.002.

25. Nelson, J.W., Randolph, P.B., Shen, S.P., Everette, K.A., Chen, P.J., Anzalone, A.V., An, M., Newby, G.A., Chen, J.C., Hsu, A., and Liu, D.R. (2022). Engineered pegRNAs improve prime editing efficiency. Nat. Biotechnol. *40*, 402–410. https://doi.org/10.1038/s41587-021-01039-7.

26. Daliri, K., Hescheler, J., and Pfannkuche, K.P. (2024). Prime Editing and DNA Repair System: Balancing Efficiency with Safety. Cells *13*, 858. https://doi.org/10.3390/cells13100858.

27. Tao, J., Bauer, D.E., and Chiarle, R. (2023). Assessing and advancing the safety of CRISPR-Cas tools: from DNA to RNA editing. Nat. Commun. *14*, 212. https://doi.org/10.1038/s41467-023-35886-6.

28. Doman, J.L., Sousa, A.A., Randolph, P.B., Chen, P.J., and Liu, D.R. (2022). Designing and executing prime editing experiments in mammalian cells. Nat. Protoc. *17*, 2431–2468. https://doi.org/10.1038/s41596-022-00724-4.

29. Siegner, S.M., Karasu, M.E., Schröder, M.S., Kontarakis, Z., and Corn, J.E. (2021). PnB Designer: a web application to design prime and base editor guide RNAs for animals and plants. BMC Bioinformatics *22*, 101. https://doi.org/10.1186/s12859-021-04034-6.

30. Yu, G., Kim, H.K., Park, J., Kwak, H., Cheong, Y., Kim, D., Kim, J., Kim, J., and Kim, H.H. (2023). Prediction of efficiencies for diverse prime editing systems in multiple cell types. Cell *186*, 2256–2272.e23. https://doi.org/10.1016/j.cell.2023.03.034.

31. Jeong, Y.K., Song, B., and Bae, S. (2020). Current Status and Challenges of DNA Base Editing Tools. Mol. Ther. *28*, 1938–1952. https://doi.org/10.1016/j.ymthe.2020.07.021.

32. Lee, S.H., Wu, J., Im, D., Hwang, G.H., Jeong, Y.K., Jiang, H., Lee, S.J., Jo, D.H., Goddard, W.A., 3rd, Kim, J.H., and Bae, S. (2024). Bystander base editing interferes with visual function restoration in Leber congenital amaurosis. Preprint at bioRxiv. https://doi.org/10.1101/2024.10.23.619839.

33. Doench, J.G., Fusi, N., Sullender, M., Hegde, M., Vaimberg, E.W., Donovan, K.F., Smith, I., Tothova, Z., Wilen, C., Orchard, R., et al. (2016). Optimized sgRNA design to maximize activity and minimize off-target effects of CRISPR-Cas9. Nat. Biotechnol. *34*, 184–191. https://doi.org/10.1038/nbt.3437.

34. Paquet, D., Kwart, D., Chen, A., Sproul, A., Jacob, S., Teo, S., Olsen, K.M., Gregg, A., Noggle, S., and Tessier-Lavigne, M. (2016). Efficient introduction of specific homozygous and heterozygous mutations using CRISPR/Cas9. Nature *533*, 125–129. https://doi.org/10.1038/nature17664.

35. Okamoto, S., Amaishi, Y., Maki, I., Enoki, T., and Mineno, J. (2019). Highly efficient genome editing for single-base substitutions using optimized ssODNs with Cas9-RNPs. Sci. Rep. *9*, 4811. https://doi.org/10.1038/s41598-019-41121-4.

36. Corsi, G.I., Qu, K., Alkan, F., Pan, X., Luo, Y., and Gorodkin, J. (2022). CRISPR/Cas9 gRNA activity depends on free energy changes and on the target PAM context. Nat. Commun. *13*, 3006. https://doi.org/10.1038/s41467-022-30515-0.

37. Mao, Z., Bozzella, M., Seluanov, A., and Gorbunova, V. (2008). DNA repair by nonhomologous end joining and homologous recombination during cell cycle in human cells. Cell Cycle *7*, 2902–2906. https://doi.org/10.4161/cc.7.18.6679.

38. Foy, S.P., Jacoby, K., Bota, D.A., Hunter, T., Pan, Z., Stawiski, E., Ma, Y., Lu, W., Peng, S., Wang, C.L., et al. (2023). Non-viral precision T cell receptor replacement for personalized cell therapy. Nature *615*, 687–696. https://doi.org/10.1038/s41586-022-05531-1.

39. Webber, B.R., Lonetree, C.L., Kluesner, M.G., Johnson, M.J., Pomeroy, E.J., Diers, M.D., Lahr, W.S., Draper, G.M., Slipek, N.J., Smeester, B.A., et al. (2019). Highly efficient multiplex human T cell engineering without double-strand breaks using Cas9 base editors. Nat. Commun. *10*, 5222. https://doi.org/10.1038/s41467-019-13007-6.

40. Kaustio, M., Nayebzadeh, N., Hinttala, R., Tapiainen, T., Åström, P., Mamia, K., Pernaa, N., Lehtonen, J., Glumoff, V., Rahikkala, E., et al. (2021). Loss of DIAPH1 causes SCBMS, combined immunodeficiency, and mitochondrial dysfunction. J. Allergy Clin. Immunol. *148*, 599–611. https://doi.org/10.1016/j.jaci.2020.12.656.

41. Reint, G., Li, Z., Labun, K., Keskitalo, S., Soppa, I., Mamia, K., Tolo, E., Szymanska, M., Meza-Zepeda, L.A., Lorenz, S., et al. (2021). Rapid genome editing by CRISPR-Cas9-POLD3 fusion. Elife *10*, e75415. https://doi.org/10.7554/eLife.75415.

42. Frangoul, H., Locatelli, F., Sharma, A., Bhatia, M., Mapara, M., Molinari, L., Wall, D., Liem, R.I., Telfer, P., Shah, A.J., et al. (2024). Exagamglogene Autotemcel for Severe Sickle Cell Disease. N. Engl. J. Med. *390*, 1649–1662. https://doi.org/10.1056/NEJMoa2309676.

43. Locatelli, F., Lang, P., Wall, D., Meisel, R., Corbacioglu, S., Li, A.M., de la Fuente, J., Shah, A.J., Carpenter, B., Kwiatkowski, J.L., et al. (2024). Exagamglogene Autotemcel for Transfusion-Dependent β-Thalassemia. N. Engl. J. Med. *390*, 1663–1676. https://doi.org/10.1056/NEJMoa2309673.

44. Richardson, C.D., Ray, G.J., DeWitt, M.A., Curie, G.L., and Corn, J.E. (2016). Enhancing homology-directed genome editing by catalytically active and inactive CRISPR-Cas9 using asymmetric donor DNA. Nat. Biotechnol. *34*, 339–344. https://doi.org/10.1038/nbt.3481.

45. Schubert, M.S., Thommandru, B., Woodley, J., Turk, R., Yan, S., Kurgan, G., McNeill, M.S., and Rettig, G.R. (2021). Optimized design parameters for CRISPR Cas9 and Cas12a homology-directed repair. Sci. Rep. *11*, 19482. https://doi.org/10.1038/s41598-021-98965-y.

46. Ghasemi, H.I., Bacal, J., Yoon, A.C., Tavasoli, K.U., Cruz, C., Vu, J.T., Gardner, B.M., and Richardson, C.D. (2023). Interstrand crosslinking of homologous repair template DNA enhances gene editing in human cells. Nat. Biotechnol. *41*, 1398–1404. https://doi.org/10.1038/s41587-022-01654-y.

47. Aird, E.J., Lovendahl, K.N., St Martin, A., Harris, R.S., and Gordon, W.R. (2018). Increasing Cas9-mediated homology-directed repair efficiency through covalent tethering of DNA repair template. Commun. Biol. *1*, 54. https://doi.org/10.1038/s42003-018-0054-2.

48. Savic, N., Ringnalda, F.C., Lindsay, H., Berk, C., Bargsten, K., Li, Y., Neri, D., Robinson, M.D., Ciaudo, C., Hall, J., et al. (2018). Covalent linkage of the DNA repair template to the CRISPR-Cas9 nuclease enhances homology-directed repair. Elife *7*, e33761. https://doi.org/10.7554/eLife.33761.

49. Savić, N., Ringnalda, F.C., Berk, C., Bargsten, K., Hall, J., Jinek, M., and Schwank, G. (2019). In vitro Generation of CRISPR-Cas9 Complexes with Covalently Bound Repair Templates for Genome Editing in Mammalian Cells. Bio Protoc. *9*, e3136. https://doi.org/10.21769/BioProtoc.3136.

50. Karasu, M.E., Toufektchan, E., Chen, Y., Albertelli, A., Cullot, G., Maciejowski, J., and Corn, J.E. (2025). Removal of TREX1 activity enhances CRISPR-Cas9-mediated homologous recombination. Nat. Biotechnol. *43*, 1168–1176. https://doi.org/10.1038/s41587-024-02356-3.

51. Robert, F., Barbeau, M., Éthier, S., Dostie, J., and Pelletier, J. (2015). Pharmacological inhibition of DNA-PK stimulates Cas9-mediated genome editing. Genome Med. *7*, 93. https://doi.org/10.1186/s13073-015-0215-6.

52. Kwak, J.M., Lee, Y., Shin, S.W., and Lee, J.S. (2021). Hydroxyurea selection for enhancement of homology-directed targeted integration of transgenes in CHO cells. N. Biotechnol. *62*, 26–31. https://doi.org/10.1016/j.nbt.2021.01.007.

53. Tsai, S.Q., Zheng, Z., Nguyen, N.T., Liebers, M., Topkar, V.V., Thapar, V., Wyvekens, N., Khayter, C., Iafrate, A.J., Le, L.P., et al. (2015). GUIDE-seq enables genome-wide profiling of off-target cleavage by CRISPR-Cas nucleases. Nat. Biotechnol. *33*, 187–197. https://doi.org/10.1038/nbt.3117.

54. Malinin, N.L., Lee, G., Lazzarotto, C.R., Li, Y., Zheng, Z., Nguyen, N.T., Liebers, M., Topkar, V.V., Iafrate, A.J., Le, L.P., et al. (2021). Defining genome-wide CRISPR-Cas genome-editing nuclease activity with GUIDE-seq. Nat. Protoc. *16*, 5592–5615. https://doi.org/10.1038/s41596-021-00626-x.

55. Hunt, J.M.T., Samson, C.A., Rand, A.D., and Sheppard, H.M. (2023). Unintended CRISPR-Cas9 editing outcomes: a review of the detection and prevalence of structural variants generated by gene-editing in human cells. Hum. Genet. *142*, 705–720. https://doi.org/10.1007/s00439-023-02561-1.

56. Tsuchida, C.A., Brandes, N., Bueno, R., Trinidad, M., Mazumder, T., Yu, B., Hwang, B., Chang, C., Liu, J., Sun, Y., et al. (2023). Mitigation of chromosome loss in clinical CRISPR-Cas9-engineered T cells. Cell *186*, 4567–4582.e20. https://doi.org/10.1016/j.cell.2023.08.041.

57. Alexandrov, L.B., Kim, J., Haradhvala, N.J., Huang, M.N., Tian Ng, A.W., Wu, Y., Boot, A., Covington, K.R., Gordenin, D.A., Bergstrom, E.N., et al. (2020). The repertoire of mutational signatures in human cancer. Nature *578*, 94–101. https://doi.org/10.1038/s41586-020-1943-3.

58. Munck, J.M., Batey, M.A., Zhao, Y., Jenkins, H., Richardson, C.J., Cano, C., Tavecchio, M., Barbeau, J., Bardos, J., Cornell, L., et al. (2012). Chemosensitization of cancer cells by KU-0060648, a dual inhibitor of DNA-PK and PI-3K. Mol. Cancer Ther. *11*, 1789–1798. https://doi.org/10.1158/1535-7163.Mct-11-0535.

59. Wu, Z., Gao, S., Watanabe, N., Batchu, S., Kajigaya, S., Diamond, C., Alemu, L., Raffo, D.Q., Feng, X., Hoffmann, P., et al. (2022). Single-cell profiling of T lymphocytes in deficiency of adenosine deaminase 2. J. Leukoc. Biol. *111*, 301–312. https://doi.org/10.1002/jlb.5a0621-314r.

60. Huang, D.W., Sherman, B.T., and Lempicki, R.A. (2009). Systematic and integrative analysis of large gene lists using DAVID bioinformatics resources. Nat. Protoc. *4*, 44–57. https://doi.org/10.1038/nprot.2008.211.

61. Huang, D.W., Sherman, B.T., and Lempicki, R.A. (2009). Bioinformatics enrichment tools: paths toward the comprehensive functional analysis of large gene lists. Nucleic Acids Res. *37*, 1–13. https://doi.org/10.1093/nar/gkn923.

62. Pierce, G.F., and Polmar, S.H. (1982). Lymphocyte dysfunction in cartilage hair hypoplasia. II. Evidence for a cell cycle specific defect in T cell growth. Clin. Exp. Immunol. *50*, 621–628.

63. Largent, A.D., Lambert, K., Chiang, K., Shumlak, N., Liggitt, D., Oukka, M., Torgerson, T.R., Buckner, J.H., Allenspach, E.J., Rawlings, D.J., and Jackson, S.W. (2023). Dysregulated IFN-γ signals promote autoimmunity in STAT1 gain-of-function syndrome. Sci. Transl. Med. *15*, eade7028. https://doi.org/10.1126/scitranslmed.ade7028.

64. Okada, S., Asano, T., Moriya, K., Boisson-Dupuis, S., Kobayashi, M., Casanova, J.L., and Puel, A. (2020). Human STAT1 Gain-of-Function Heterozygous Mutations: Chronic Mucocutaneous Candidiasis and Type I Interferonopathy. J. Clin. Immunol. *40*, 1065–1081. https://doi.org/10.1007/s10875-020-00847-x.

65. Toubiana, J., Okada, S., Hiller, J., Oleastro, M., Lagos Gomez, M., Aldave Becerra, J. C., Ouachée-Chardin, M., Fouyssac, F., Girisha, K.M., Etzioni, A., et al. (2016). Heterozygous STAT1 gain-of-function mutations underlie an unexpectedly broad clinical phenotype. Blood *127*, 3154–3164. https://doi.org/10.1182/blood-2015-11-679902.

66. Liu, L., Okada, S., Kong, X.F., Kreins, A.Y., Cypowyj, S., Abhyankar, A., Toubiana, J., Itan, Y., Audry, M., Nitschke, P., et al. (2011). Gain-of-function human STAT1 mutations impair IL-17 immunity and underlie chronic mucocutaneous candidiasis. J. Exp. Med. *208*, 1635–1648. https://doi.org/10.1084/jem.20110958.

67. Zimmerman, O., Olbrich, P., Freeman, A.F., Rosen, L.B., Uzel, G., Zerbe, C.S., Rosenzweig, S.D., Kuehn, H.S., Holmes, K.L., Stephany, D., et al. (2019). STAT1 Gain-of-Function Mutations Cause High Total STAT1 Levels With Normal Dephosphorylation. Front. Immunol. *10*, 1433. https://doi.org/10.3389/fimmu.2019.01433.

68. Besnard, M., Padonou, F., Provin, N., Giraud, M., and Guillonneau, C. (2021). AIRE deficiency, from preclinical models to human APECED disease. Dis. Model. Mech. *14*, dmm046359. https://doi.org/10.1242/dmm.046359.

69. Cromer, M.K., Majeti, K.R., Rettig, G.R., Murugan, K., Kurgan, G.L., Bode, N.M., Hampton, J.P., Vakulskas, C.A., Behlke, M.A., and Porteus, M.H. (2023). Comparative analysis of CRISPR off-target discovery tools following ex vivo editing of CD34(+) hematopoietic stem and progenitor cells. Mol. Ther. *31*, 1074–1087. https://doi.org/10.1016/j.ymthe.2023.02.011.

70. Lazar, N.H., Celik, S., Chen, L., Fay, M.M., Irish, J.C., Jensen, J., Tillinghast, C.A., Urbanik, J., Bone, W.P., Gibson, C.C., and Haque, I.S. (2024). High-resolution genome-wide mapping of chromosome-arm-scale truncations induced by CRISPR-Cas9 editing. Nat. Genet. *56*, 1482–1493. https://doi.org/10.1038/s41588-024-01758-y.

71. Leibowitz, M.L., Papathanasiou, S., Doerfler, P.A., Blaine, L.J., Sun, L., Yao, Y., Zhang, C.Z., Weiss, M.J., and Pellman, D. (2021). Chromothripsis as an on-target consequence of CRISPR-Cas9 genome editing. Nat. Genet. *53*, 895–905. https://doi.org/10.1038/s41588-021-00838-7.

72. Alanis-Lobato, G., Zohren, J., McCarthy, A., Fogarty, N.M.E., Kubikova, N., Hardman, E., Greco, M., Wells, D., Turner, J.M.A., and Niakan, K.K. (2021). Frequent loss of heterozygosity in CRISPR-Cas9-edited early human embryos. Proc. Natl. Acad. Sci. USA *118*, e2004832117. https://doi.org/10.1073/pnas.2004832117.

73. Stadtmauer, E.A., Fraietta, J.A., Davis, M.M., Cohen, A.D., Weber, K.L., Lancaster, E., Mangan, P.A., Kulikovskaya, I., Gupta, M., Chen, F., et al. (2020). CRISPR-engineered T cells in patients with refractory cancer. Science *367*, eaba7365. https://doi.org/10.1126/science.aba7365.

74. Wimberger, S., Akrap, N., Firth, M., Brengdahl, J., Engberg, S., Schwinn, M.K., Slater, M.R., Lundin, A., Hsieh, P.P., Li, S., et al. (2023). Simultaneous inhibition of DNA-PK and PolΘ improves integration efficiency and precision of genome editing. Nat. Commun. *14*, 4761. https://doi.org/10.1038/s41467-023-40344-4.

75. Pugliano, C.M., Berger, M., Ray, R.M., Sapkos, K., Wu, B., Laird, A., Ye, Y., Thomson, D., DeGottardi, M.Q., Khan, I.F., et al. (2024). DNA-PK inhibition enhances gene editing efficiency in HSPCs for CRISPR-based treatment of X-linked hyper IgM syndrome. Mol. Ther. Methods Clin. Dev. *32*, 101297. https://doi.org/10.1016/j.omtm.2024.101297.

76. Cullot, G., Aird, E.J., Schlapansky, M.F., Yeh, C.D., van de Venn, L., Vykhlyantseva, I., Kreutzer, S., Mailänder, D., Lewków, B., Klermund, J., et al. (2024). Genome editing with the HDR-enhancing DNA-PKcs inhibitor AZD7648 causes large-scale genomic alterations. Nat. Biotechnol. https://doi.org/10.1038/s41587-024-02488-6.

77. Hunt, R.C., and Kimchi-Sarfaty, C. (2022). When Silence Disrupts. N. Engl. J. Med. *387*, 753–756. https://doi.org/10.1056/NEJMcibr2207405.

78. Rodriguez, A., Diehl, J.D., Wright, G.S., Bonar, C.D., Lundgren, T.J., Moss, M.J., Li, J., Milenkovic, T., Huber, P.W., Champion, M.M., et al. (2024). Synonymous codon

substitutions modulate transcription and translation of a divergent upstream gene by modulating antisense RNA production. Proc. Natl. Acad. Sci. USA *121*, e2405510121. https://doi.org/10.1073/pnas.2405510121.

79. Shen, X., Song, S., Li, C., and Zhang, J. (2022). Synonymous mutations in representative yeast genes are mostly strongly non-neutral. Nature *606*, 725–731. https://doi.org/10.1038/s41586-022-04823-w.

80. Labun, K., Guo, X., Chavez, A., Church, G., Gagnon, J.A., and Valen, E. (2019). Accurate analysis of genuine CRISPR editing events with ampliCan. Genome Res. *29*, 843–847. https://doi.org/10.1101/gr.244293.118.

81. Zhu, L.J., Lawrence, M., Gupta, A., Pagès, H., Kucukural, A., Garber, M., and Wolfe, S.A. (2017). GUIDEseq: a bioconductor package to analyze GUIDE-Seq datasets for CRISPR-Cas nucleases. BMC Genomics *18*, 379. https://doi.org/10.1186/s12864-017-3746-y.

82. Labun, K., Rio, O., Tjeldnes, H., Swirski, M., Komisarczuk, A.Z., Haapaniemi, E., and Valen, E. (2025). CHOPOFF: symbolic alignments enable fast and sensitive CRISPR off-target detection. Preprint at bioRxiv. https://doi.org/10.1101/2025.01.06.603201.

83. Picelli, S., Faridani, O.R., Björklund, A.K., Winberg, G., Sagasser, S., and Sandberg, R. (2014). Full-length RNA-seq from single cells using Smart-seq2. Nat. Protoc. *9*, 171–181. https://doi.org/10.1038/nprot.2014.006.

84. Martin, M. (2011). Cutadapt Removes Adapter Sequences From High-Throughput Sequencing Reads. EMBnet. J. *17*, 10. https://doi.org/10.14806/ej.17.1.200.

85. Dobin, A., Davis, C.A., Schlesinger, F., Drenkow, J., Zaleski, C., Jha, S., Batut, P., Chaisson, M., and Gingeras, T.R. (2013). STAR: ultrafast universal RNA-seq aligner. Bioinformatics *29*, 15–21. https://doi.org/10.1093/bioinformatics/bts635.

86. McKenna, A., Hanna, M., Banks, E., Sivachenko, A., Cibulskis, K., Kernytsky, A., Garimella, K., Altshuler, D., Gabriel, S., Daly, M., and DePristo, M.A. (2010). The Genome Analysis Toolkit: a MapReduce framework for analyzing next-generation DNA sequencing data. Genome Res. *20*, 1297–1303. https://doi.org/10.1101/gr.107524.110.

87. Anders, S., Pyl, P.T., and Huber, W. (2015). HTSeq–a Python framework to work with high-throughput sequencing data. Bioinformatics *31*, 166–169. https://doi.org/10.1093/bioinformatics/btu638.

88. Satija, R., Farrell, J.A., Gennert, D., Schier, A.F., and Regev, A. (2015). Spatial reconstruction of single-cell gene expression data. Nat. Biotechnol. *33*, 495–502. https://doi.org/10.1038/nbt.3192.

89. Korotkevich, G., Sukhov, V., Budin, N., Shpak, B., Artyomov, M.N., and Sergushichev, A. (2021). Fast gene set enrichment analysis. Preprint at bioRxiv. https://doi.org/10.1101/060012.

90. Demichev, V., Messner, C.B., Vernardis, S.I., Lilley, K.S., and Ralser, M. (2020). DIA-NN: neural networks and interference correction enable deep proteome coverage in high throughput. Nat. Methods *17*, 41–44. https://doi.org/10.1038/s41592-019-0638-x.

91. Demichev, V., Szyrwiel, L., Yu, F., Teo, G.C., Rosenberger, G., Niewienda, A., Ludwig, D., Decker, J., Kaspar-Schoenefeld, S., Lilley, K.S., et al. (2022). dia-PASEF data analysis using FragPipe and DIA-NN for deep proteomics of low sample amounts. Nat. Commun. *13*, 3944. https://doi.org/10.1038/s41467-022-31492-0.

92. Virtanen, P., Gommers, R., Oliphant, T.E., Haberland, M., Reddy, T., Cournapeau, D., Burovski, E., Peterson, P., Weckesser, W., Bright, J., et al. (2020). SciPy 1.0: fundamental algorithms for scientific computing in Python. Nat. Methods *17*, 261–272. https://doi.org/10.1038/s41592-019-0686-2.

93. Seabold, S., and Perktold, J. (2010). Statsmodels: econometric and statistical modeling with Python. In Proceedings of the 9th Python in Science Conference, https://doi.org/10.25080/Majora-92bf1922-011.

YMTHE, Volume *33*

Supplemental Information

Precision T cell correction platform for inborn errors of immunity

Katariina Mamia, Solrun Kolbeinsdottir, Kornel Labun, Zhuokun Li, Anna Komisarczuk, Salla Keskitalo, Ganna Reint, Frida Loe Haugen, Britt Olaug Lindestad, Siv Skundberg Jensen, Thea Johanne Gjerdingen, Antti Tuhkala, Carolina Wieczorek Ervik, Pavel Kopcil, Nail Fatkhutdinov, Karen Helene Bronken Martinsen, Hans Christian Erichsen, Monika Szymanska, Eero Tölö, Virpi Glumoff, Janna Saarela, Trond Melbye Michelsen, Camilla Schalin-Jäntti, Johanna Olweus, Eira Leinonen, Markku Varjosalo, Eivind Valen, Timo Hautala, Martin Enge, Timi Martelius, Shiva Dahal-Koirala, and Emma Haapaniemi

SUPPLEMENTAL FIGURES

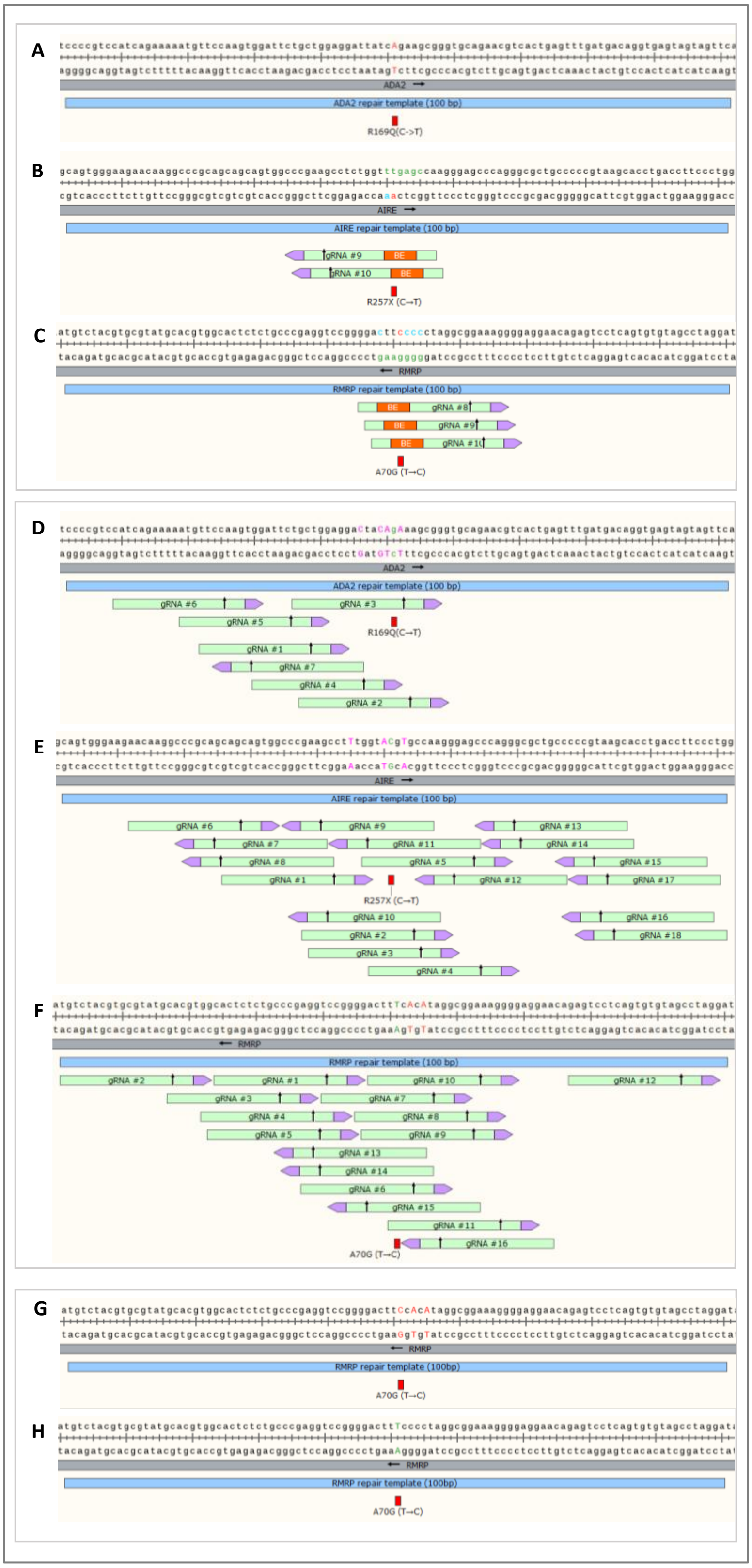

Figure S1. Repair template and gRNA design for *ADA2*, *AIRE* and *RMRP*

(A) Schematic representation of *ADA2* mutant site, marked in red, with no possible base editing gRNAs. (B) Schematic representation of *AIRE* mutant site, where the edited pathogenic mutation nucleotide is marked with red, showing possible A→G base editing gRNAs. The nucleotide positions which fall within the editing window span of BE guides are shown in green and the bystander edits are shown in blue. (C) Schematic representation of *RMRP* mutant site, where the edited pathogenic mutation nucleotide is marked with red, showing possible A→G base editing gRNAs. The nucleotide positions which fall within the editing window span of BE guides are shown in green and the bystander edits are shown in blue. (D) Schematic representation ADA2 gRNA design

and repair strategy. Correction of pathogenic mutation (red) is marked with green and silent SNVs as pink uppercase letters. (E) Schematic representation AIRE gRNA design and repair strategy, as explained in (d). (F) Schematic representation RMRP gRNA design and repair strategy, as explained in (d). As *RMRP* is noncoding, non-silent SNVs (red) were added in the repair strategy for early experiments. (G) Schematic representation of RMRP SVP strategy for editing wild-type cells, where non-silent SNVs (red) were added in the repair strategy for early experiments. (H) Schematic representation of *RMRP* repair strategy for mutation (red) correction (green). Abbreviations: gRNA (guide-RNA), BE (base editing), SNV (singe nucleotide variant), gRNA (guide-RNA).

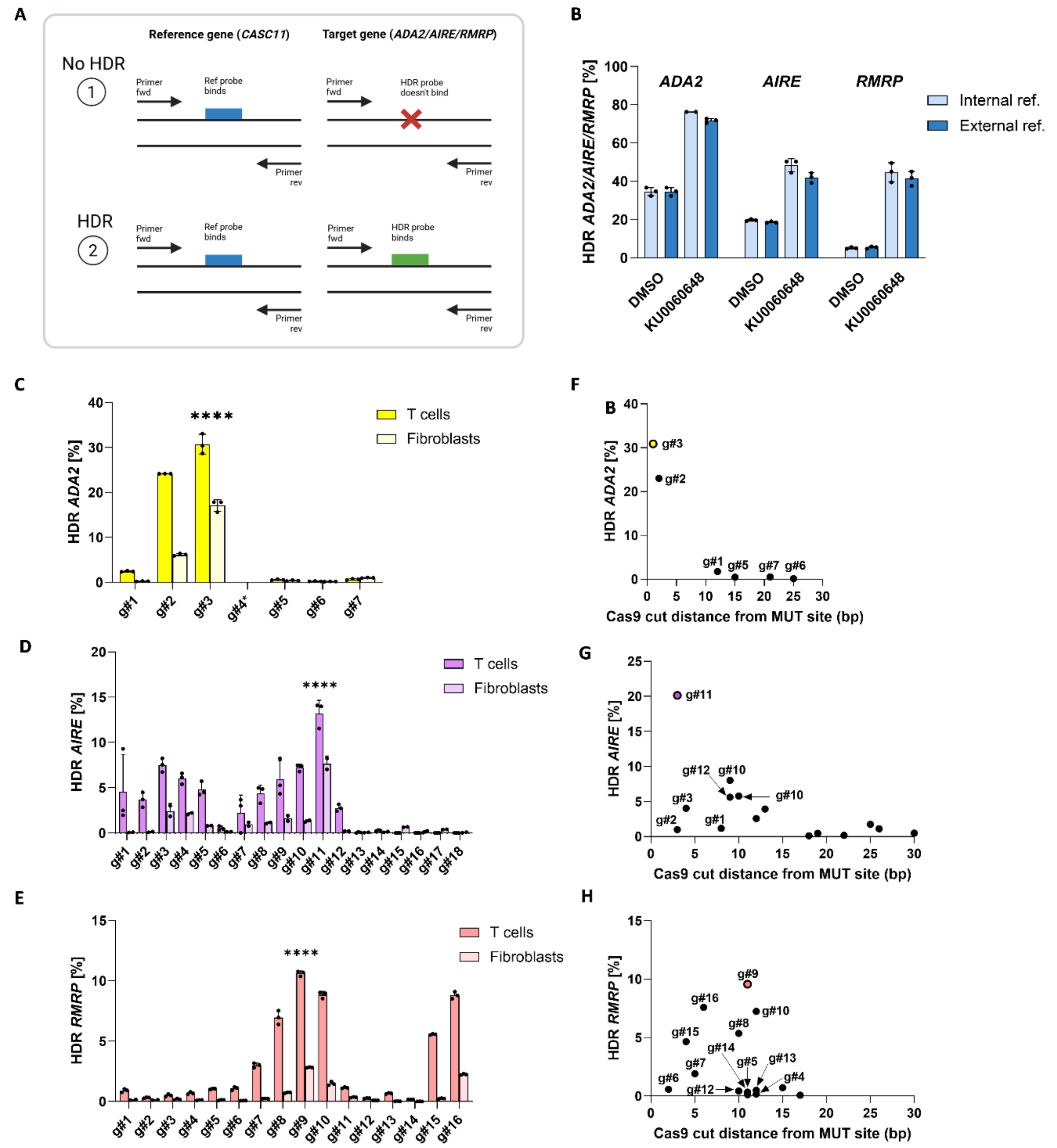

I

	Predicted #1	Predicted #2	Predicted #3
Atum	Not tested	g1	g5
Benchling	g6	Not tested	g2
CHOPCHOP	g6	g5	Not tested
CRISPROR	Not tested	Not tested	g5
DeepSpCas9	Not tested	g2	g5
EuPaGDT	g5	g2	Not tested
IDT design tool	Not tested	g5	g1

J

	Predicted #1	Predicted #2	Predicted #3
Atum	g15	g16	g13
Benchling	g11	g4	g9
CHOPCHOP	g11	g4	g2
CRISPROR	g15	g13	g16
DeepSpCas9	g4	g11	g5
EuPaGDT	g16	g17	g15
IDT design tool	g10	g4	g9

K

	Predicted #1	Predicted #2	Predicted #3
Atum	Not tested	Not tested	Not tested
Benchling	g7	g3	g11
CHOPCHOP	g7	g3	g11
CRISPROR	g2	Not tested	g16
DeepSpCas9	g11	g10	g1
EuPaGDT	g5	g10	g4
IDT design tool	g12	g3	g2

Figure S2. ddPCR assay design, gRNA screening results and *in silico* gRNA predictions for *ADA2*, *AIRE* and *RMRP*

(A) Schematic representation of an alternative ddPCR assay design for HDR detection, which uses an external reference probe binding to *CASC11* locus, while using an internal HDR probe that binds to the edited *ADA2*, *AIRE* or *RMRP* locus, respectively. Original ddPCR assay design schematic with an internal reference probe is shown in Fig. 1C. (B) Comparison of HDR frequencies reported by a ddPCR assay using an internal reference probe (light blue) and an external one (dark blue) in *ADA2*, *AIRE* and *RMRP* -edited HD T cells treated with 0.5 μM KU0060648 or DMSO. (C) ADA2 gRNA screening in DADA2 patient T cells and fibroblasts, HDR assessed by amplicon sequencing (n=3 technical replicates). (D) AIRE gRNA screening in APECED patient T cells and fibroblasts, HDR assessed by amplicon sequencing (n=3 technical replicates in T cells, n=2 in fibroblasts). (E) RMRP gRNA screening in CHH patient T cells and fibroblasts, HDR assessed by amplicon sequencing (n=3 technical replicates). Samples from (c-e) are the same samples assessed by ddPCR in Fig. 1E-G. Cas9 cut distance from mutation site plotted against HDR frequency for tested gRNAs in DADA2 (F), APECED (G) and CHH (H) patient T cells, where reported HDR frequencies are the ddPCR measurements reported in Fig. 1E-G. The best gRNA is shown as the coloured dot. (I) Comparison of three best ADA2 gRNAs identified by *in silico* gRNA design tools to *in vitro* validated gRNA screening results from DADA2 patient T cells, where accurate predictions are shown in green, incorrect predictions in red and gRNAs that were designed by the tools but not assessed *in vitro* in white. (J) Comparison of three best AIRE gRNAs identified by *in silico* gRNA design tools to *in vitro* validated gRNA screening results from APECED patient T cells, where accurate predictions are shown in green, incorrect predictions in red. (K) Comparison of three best RMRP gRNAs identified by *in silico* gRNA design tools to *in vitro* validated gRNA screening results from CHH patient T cells, where accurate predictions are shown in green, incorrect predictions in red and gRNAs that were designed by the tools but not assessed *in vitro* in white. One independent experiment was performed for all sets of data. Statistical

significance of highest HDR for a given gRNA was assessed by one-way ANOVA with Fisher's LSD test, where ****p<0.0001. Bar denotes mean value, error bars represent ± SD. Abbreviations: ddPCR (droplet digital PCR), gRNA (guide-RNA), DADA2 (Deficiency of adenosine deaminase 2), HDR (homology-directed repair), APECED (Autoimmune polyendocrinopathy-candidiasis-ectodermal dystrophy), CHH (Cartilage hair hypoplasia).

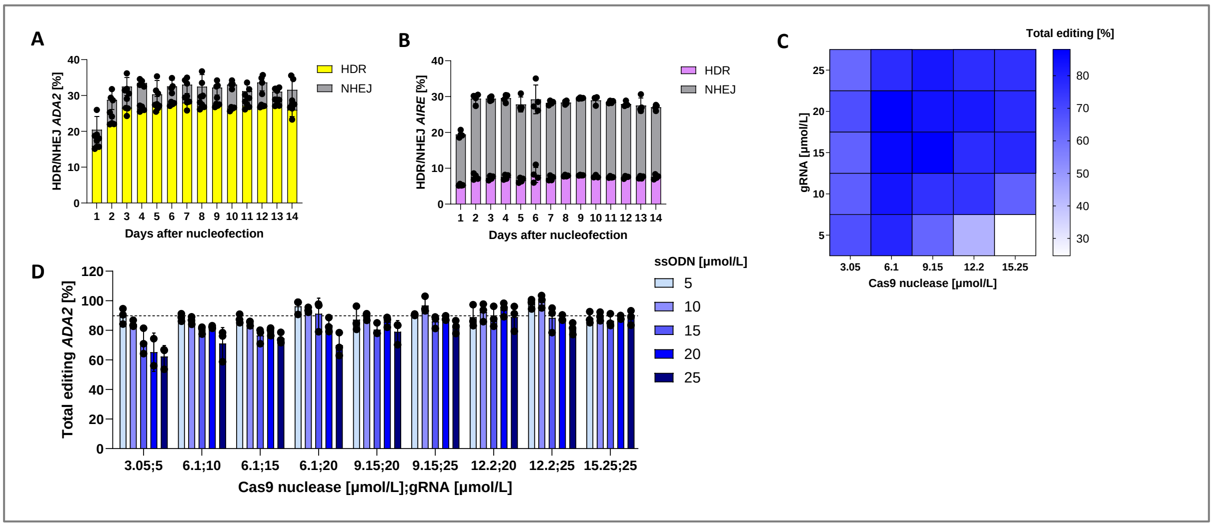

Figure S3. Optimization of CRISPR reagents and nucleofection in healthy control T cells

(A) *ADA2* HDR and NHEJ editing in HD T cells 1-14 days after nucleofection, measured by ddPCR (n=3 technical replicates). (B) *AIRE* HDR and NHEJ editing in HD T cells 1-14 days after nucleofection, measured by ddPCR (n=3 technical replicates). (C) *ADA2* total editing (reported as the sum of HDR and NHEJ) in HD T cells nucleofected with Cas9 nuclease at 3.05-15.25-, gRNA at 5-25- and ssODN at 5 µmol/L per nucleofected sample, measured by ddPCR (n=3 technical replicates). (D) *ADA2* total editing (reported as the sum of HDR and NHEJ) in HD T cells with selected combinations of RNPs with ssODN at 5-25 µmol/L per nucleofected sample, measured by ddPCR (n=3 technical replicates). Dashed line indicates mean value of Cas9 nuclease at 3.05 µmol/L-, gRNA at 5 µmol/L- and ssODN at 5 µmol/L per nucleofected sample. One independent experiment was performed for all sets of data except for (c)-(d) where one out of three representative experiments is shown. Bar denotes mean value,

error bars represent ± SD. Abbreviations: HDR (homology-directed repair), NHEJ (non-homologous end joining), HD (healthy donor), ddPCR (Droplet Digital PCR), gRNA (guide-RNA), ssODN (single-stranded oligodinucleotide), RNP (ribonucleoprotein).

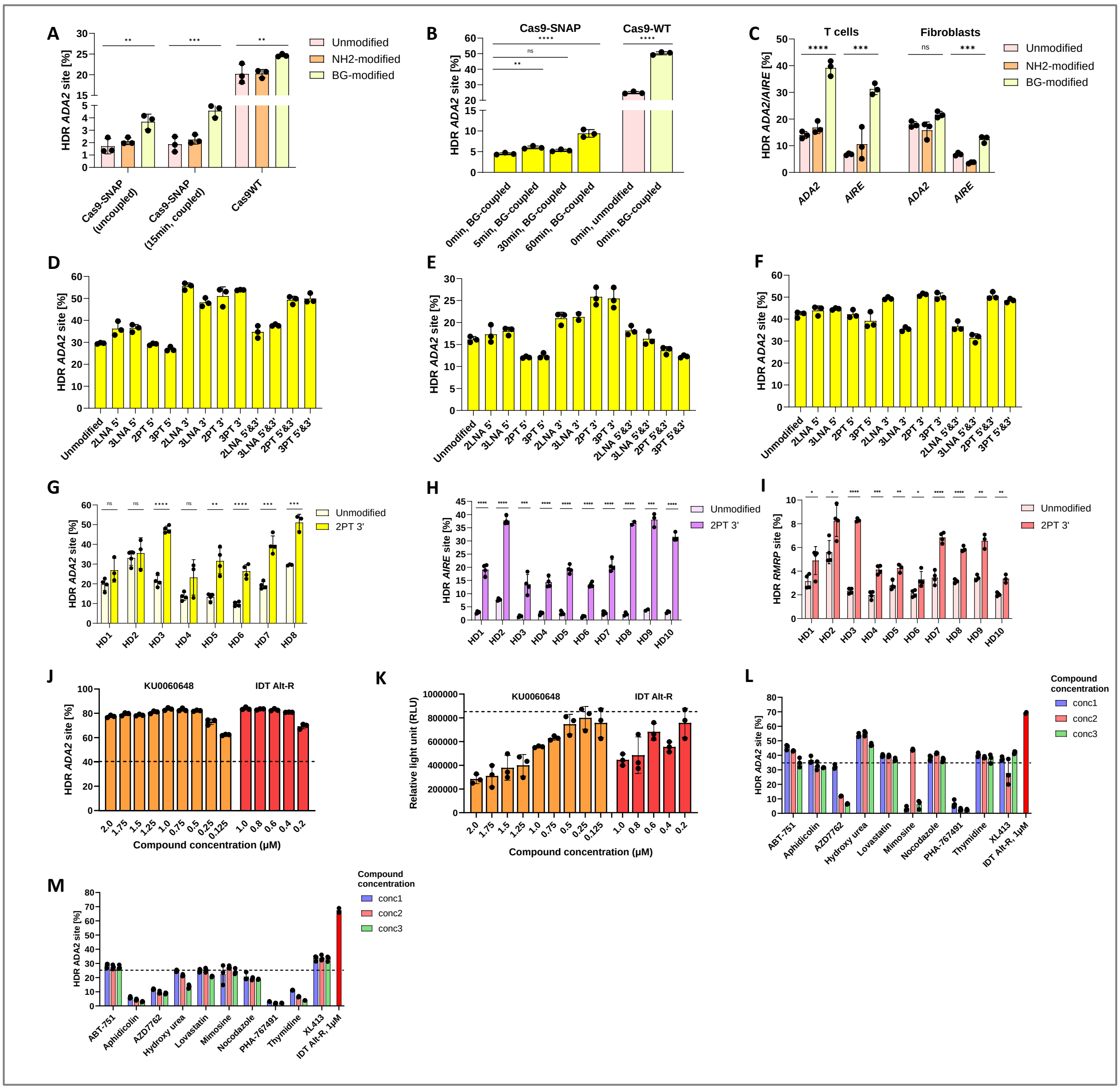

Figure S4. Assessing HDR improvement strategies in healthy control primary cells

(A) *ADA2* HDR editing in HD fibroblasts with unmodified, NH2- or BG-modified ssODNs with Cas9-SNAP (uncoupled or coupled with BG) or Cas9WT nuclease (uncoupled), measured by ddPCR (n=3 technical replicates). (B) *ADA2* HDR editing in HD T cells with unmodified (pink bar) or BG-modified (bright and pale-yellow bars) ssODNs with Cas9-SNAP or Cas9WT

nuclease, measured by ddPCR (n=3 technical replicates). (C) *ADA2* and *AIRE* HDR editing in HD T cells and fibroblasts with unmodified, NH2- or BG-modified ssODNs with Cas9WT nuclease, measured by ddPCR (n=3 technical replicates). *ADA2* HDR editing with LNA- and PT-modified ssODNs in HD (D) T cells (E), fibroblasts and (F) CD34+ HSPCs, measured by ddPCR (n=3 technical replicates). HDR editing in 8-10 healthy T cell donors with position-optimized ssODNs with unmodified or 2PT 3' modified ssODNs for (G) *ADA2*, (H) *AIRE* and (I) *RMRP*, measured by ddPCR (n=3-4 technical replicates depending on the donor). Effect of HDR enhancing compounds at selected concentrations (0.125-2 μM KU0060648, 0.2-1 μM IDT Alt-R enhancer V2) in HD T cells on (J) *ADA2* HDR editing, measured by ddPCR (n=3 technical replicates), where dashed line indicates the mean of RNP baseline (DMSO), and (K) cell viability 96h after nucleofection, measured by CellTiter-Glo (n=3 technical replicates). *ADA2* HDR editing in HD T cells with cell cycle inhibitors at three concentrations in increasing order (conc1-conc3) applied (L) 24h pre- and (M) 24h post nucleofection, measured by ddPCR (n=3 technical replicates). Dashed line indicates the mean value of RNP baseline (DMSO). A single experiment was performed for all sets of data except for (j) and (k) where three independent experiments were performed, and the representative experiment is shown. Statistical significance was assessed by one-way ANOVA with Fisher's LSD test, where ****$p<0.0001$, ***$p<0.0002$, **$p<0.001$ and *$p<0.01$. Bar denotes mean value, error bars represent ± SD. Abbreviations: HDR (homology-directed repair), HD (healthy donor), BG (benzylguanine), ssODN (single-stranded oligodinucleotide), ddPCR (Droplet Digital PCR), LNA (locked nucleic acid), PT (phosphorothioate), LNA (locked nucleic acid), HSPC (hematopoietic stem and progenitor cell), RNP (ribonucleoprotein).

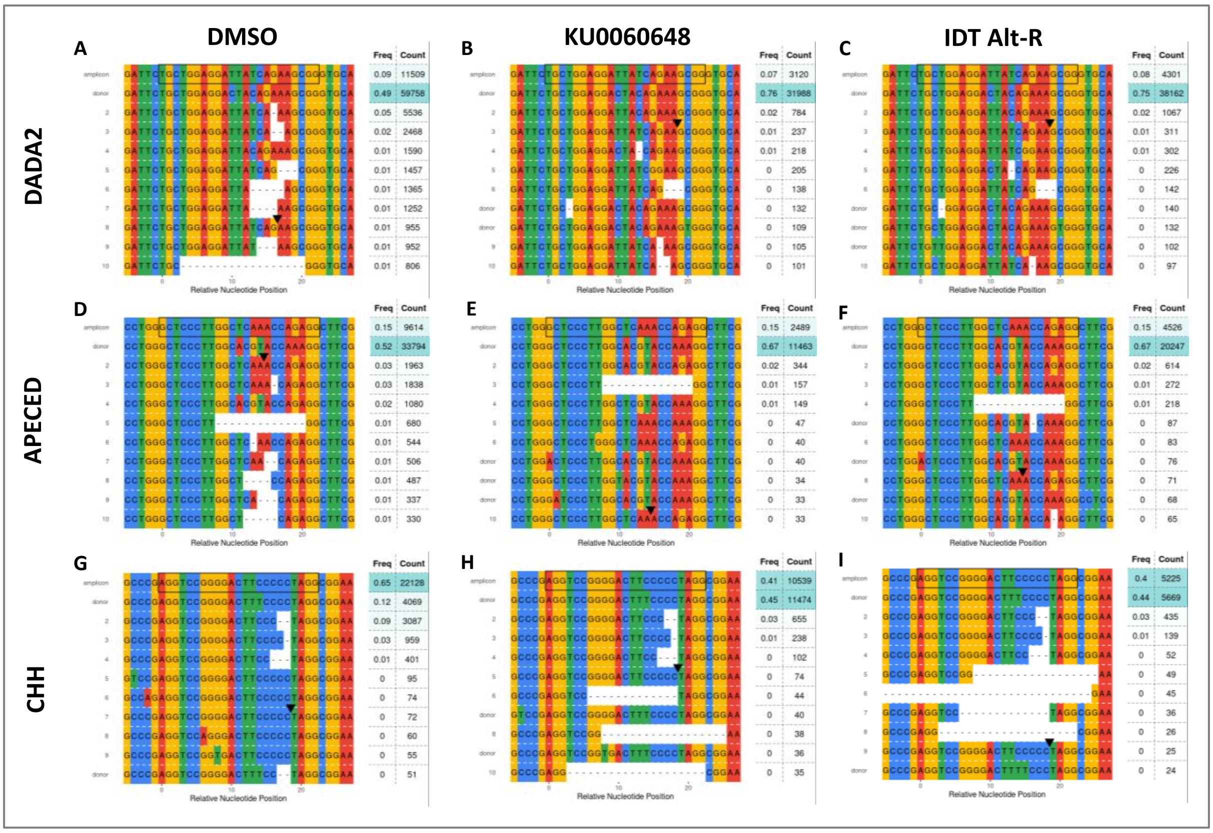

Figure S5. Amplicon sequencing variant plots in corrected DADA2, APECED and CHH patient T cells

(A-C) Amplicon sequencing variant plots in corrected DADA2 patient (DADA2 1) treated with HDR enhancing compounds (0.5 µM KU0060648 and 0.6 µM IDT Alt-R enhancer V2) or DMSO. (D-F) Amplicon sequencing variant plots in corrected APECED patient (APECED 1) treated with HDR enhancing compounds (0.5 µM KU0060648 and 0.6 µM IDT Alt-R enhancer V2) or DMSO. (G-I) Amplicon sequencing variant plots in corrected CHH patient (CHH 2) treated with HDR enhancing compounds (0.5 µM KU0060648 and 0.6 µM IDT Alt-R enhancer V2) or DMSO. For all patients, samples were collected four days after nucleofection. Edits are characterized on the left side of the plot, where "amplicon" is the unedited wild-type sequence, "donor" below the "amplicon" the perfect HDR and imperfect HDR or indels in the rows below "amplicon". Arrow indicates an insertion. Frequencies and counts are reported on the right side of the plots. HDR was assessed by amplicon sequencing, where one representative

measurement is shown (n=3 technical replicates). One independent experiment was performed for all sets of data. The patient number corresponds to patient information in Supplemental Table S15. Abbreviations: DADA2 (Deficiency of adenosine deaminase 2), APECED (Autoimmune polyendocrinopathy-candidiasis-ectodermal dystrophy), CHH (Cartilage Hair Hypoplasia).

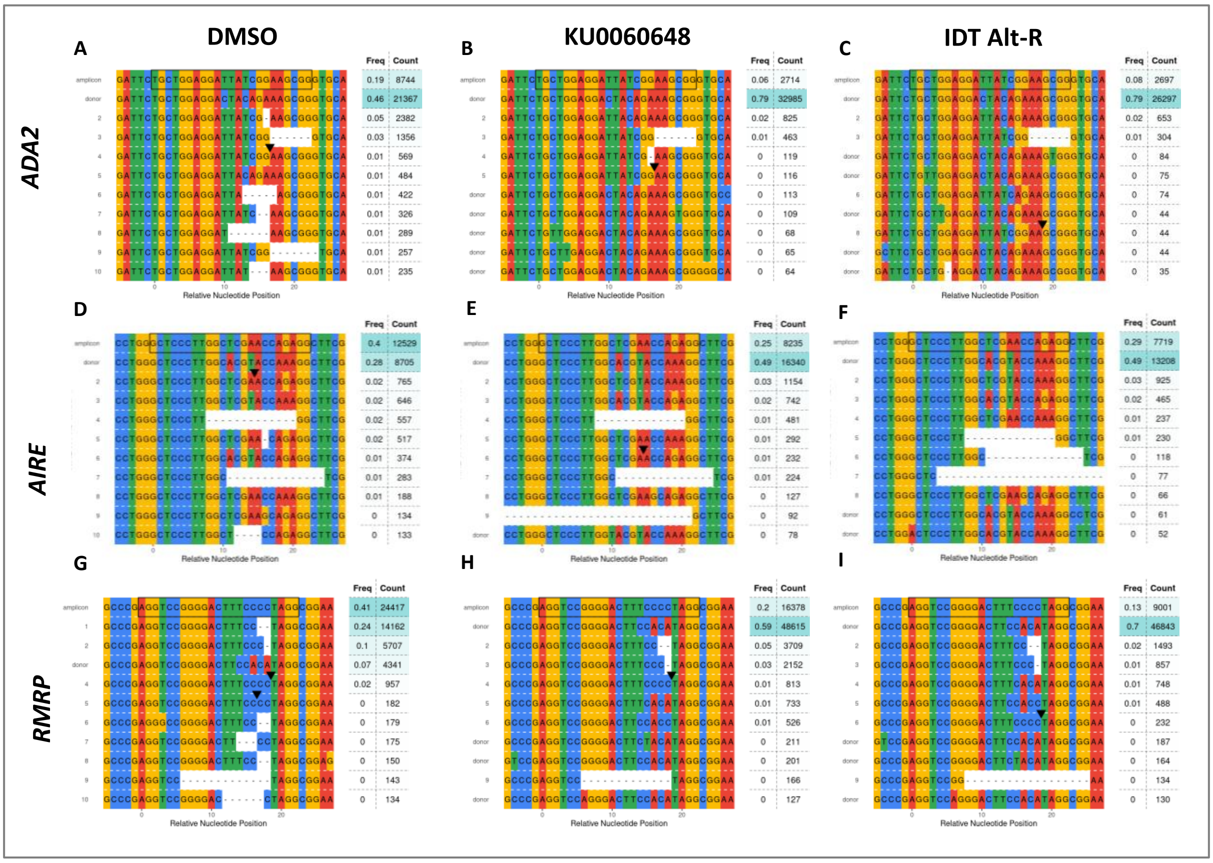

Figure S6. Amplicon sequencing variant plots in *ADA2*, *AIRE* and *RMRP* -edited healthy control T cells

(A-C) Amplicon sequencing variant plots in *ADA2-edited* HD treated with HDR enhancing compounds (0.5 µM KU0060648 and 0.6 µM IDT Alt-R enhancer V2) or DMSO. (D-F) Amplicon sequencing variant plots in *AIRE-edited* HD treated with HDR enhancing compounds (0.5 µM KU0060648 and 0.6 µM IDT Alt-R enhancer V2) or DMSO. (G-I) Amplicon sequencing variant plots in *RMRP*-edited HD treated with HDR enhancing compounds (0.5 µM KU0060648 and 0.6 µM IDT Alt-R enhancer V2) or DMSO. For all loci,

samples were collected four days after nucleofection. Edits are characterized on the left side of the plot, where “amplicon” is the unedited wild-type sequence, “donor” below the “amplicon” the perfect HDR and imperfect HDR or indels as the remaining rows below “amplicon”. Arrow indicates an insertion. Frequencies and counts are reported on the right side of the plots. HDR was assessed by amplicon sequencing, where one representative measurement is shown (n=3 technical replicates). One independent experiment was performed for all sets of data. Abbreviations: HD (healthy donor).

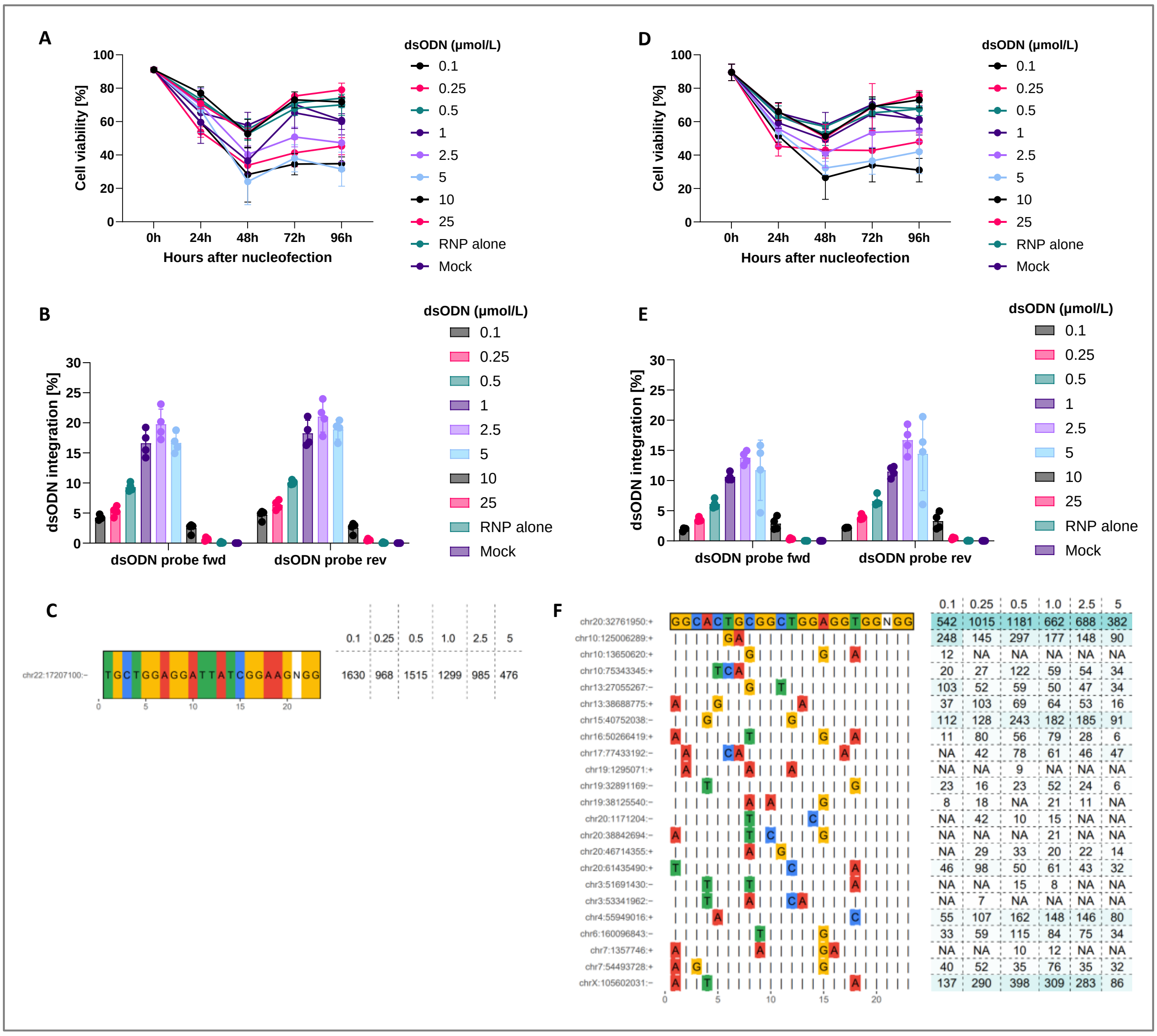

Figure S7. GUIDE-seq optimization in healthy control T cells

(A) HD T cell viability 24-96h after nucleofection with 0-25 µmol/L dsODN/nucleofected sample for *ADA2* locus (n=4 technical replicates). (B) dsODN integration in HD T cells with

0-25 μmol/L dsODN/nucleofected sample for *ADA2* locus, assessed by ddPCR (n=4 technical replicates). (C) GUIDE-seq mismatch plot for ADA2 gRNA #3 in HD T cells with dsODN at 0.1-5 μmol/L dsODN/nucleofected sample. On-target sequence is reported at the top of the table with sequencing reads for each dsODN concentration at the right. (D) HD T cell viability 24-96h after nucleofection with 0-25 μmol/L dsODN/nucleofected sample for *HEK-site4* locus (n=4 technical replicates). (E) dsODN integration in HD T cells with 0-25 μmol/L dsODN/nucleofected sample for *HEK-site4* locus, assessed by ddPCR (n=4 technical replicates). (F) GUIDE-seq mismatch plot for HEK-site4 gRNA, targeting the endogenous human embryonic kidney HEK site 4, in HD T cells with dsODN at 0.1-5 μmol/L dsODN/nucleofected sample. The most abundant off-targets are listed under the target site with their corresponding locations in the genome (left) and sequencing read counts (right). Coloured bases of off-targets indicate mismatches with the on-target site. One independent experiment was performed for all sets of data. Bar denotes mean value, error bars represent ± SD. Abbreviations: GUIDE-seq (Genome-wide, Unbiased Identification of DSBs Enabled by Sequencing), HD (healthy donor), dsODN (double-stranded oligodeoxynucleotide), ddPCR (Droplet Digital PCR), gRNA (guide-RNA), HDR (homology-directed repair), RNP (ribonucleoprotein).

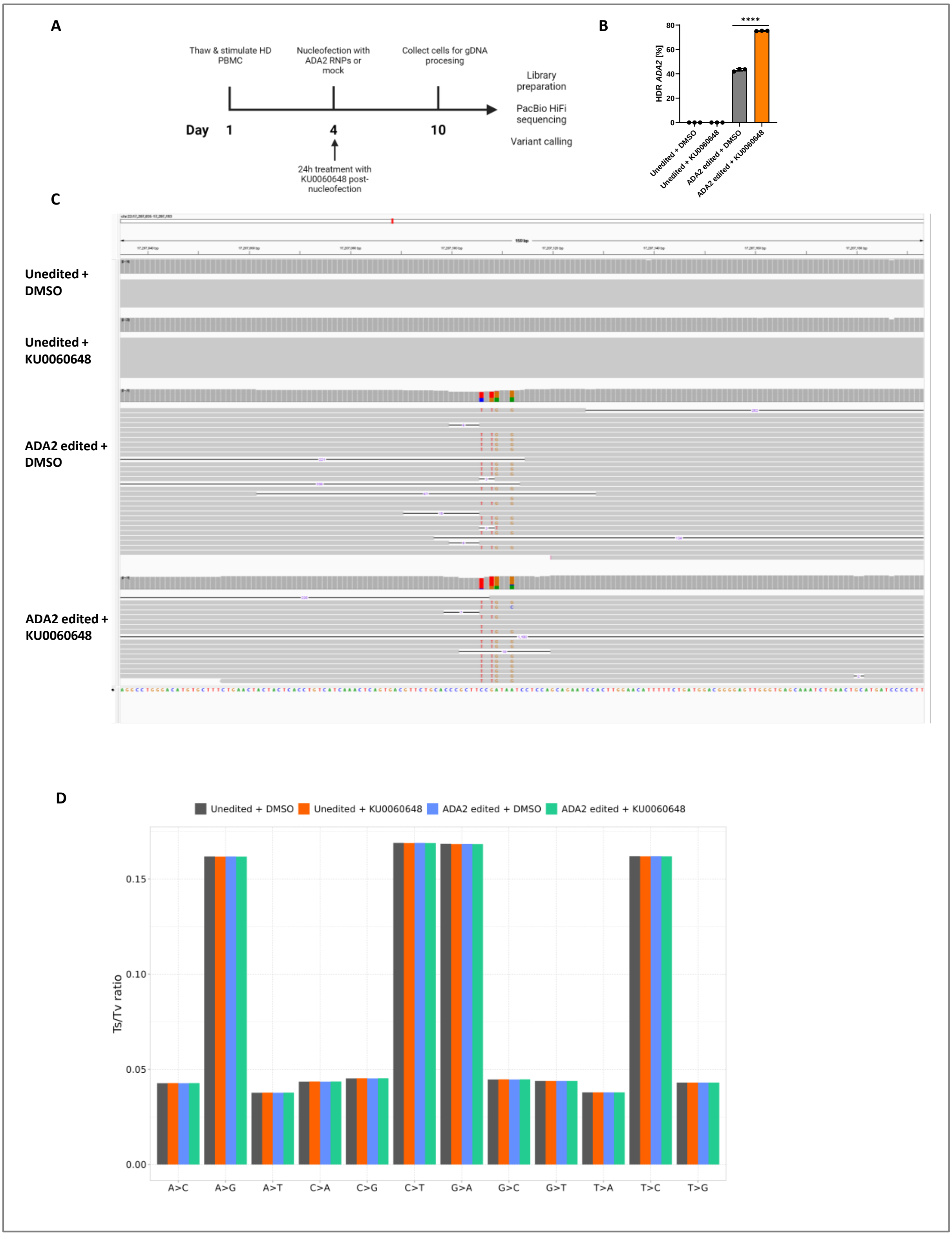

Figure S8. Whole genome sequencing of unedited and *ADA2*-edited healthy control T cells

(A) Outline of the WGS experiment, briefly discussed here: HD T cells were thawed and stimulated with IL-2 (120 U/mL), IL-7 (3 ng/µL), IL-15 (3 ng/µL) and soluble CD3/CD28 (15

μL/mL) on day 1 and nucleofected on day 4 with ADA2 RNPs or mock. Cells were cultured in IL-2 (250 U/mL) and 0.5 μM KU0060648 or DMSO for 24h after nucleofection and collected on day 10 of the platform. gDNA from samples was processed for ddPCR and PacBio sample preparation, followed by PacBio HiFi sequencing and analysis. (B) ADA2 HDR editing levels, assessed by ddPCR (measurements performed in triplicates. (C) IGV view of HiFi PacBio reads on the on-target ADA2 site. HDR reads contain four SNVs at the same time: C>T, G>T, A>G and A>G. No editing is present in the unedited samples. (D) Transition transversion ratio plot showing no difference between edited and unedited samples, showing no global CRISPR toxicity. One independent experiment was performed for all sets of data. Bar denotes mean value, error bars represent ± SD. Statistical significance was assessed by one-way ANOVA with Fisher's LSD test, where ****p<0.0001. Abbreviations: WGS (whole genome sequencing), HD (healthy donor), IL (interleukin), RNP (ribonucleoprotein), DMSO (dimethyl sulfoxide), gDNA (genomic DNA), ddPCR (Droplet Digital PCR), IGV (Integrative Genomics Viewer), HDR (homology-directed repair).

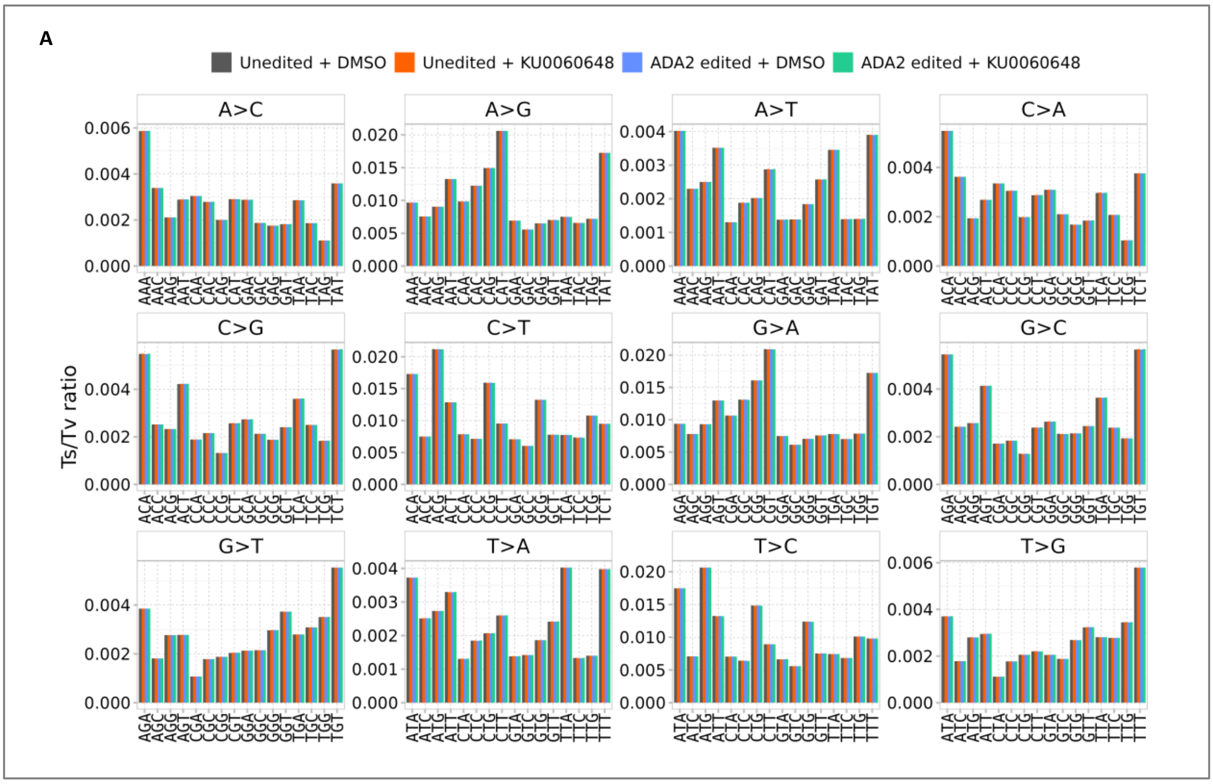

Figure S9. Whole genome sequencing of unedited and ADA2-edited healthy control T cells

(A) Mutational signature by codon shows no differences between edited and unedited samples. One independent experiment was performed for all sets of data. Abbreviations: WGS (whole genome sequencing), HD (healthy donor).

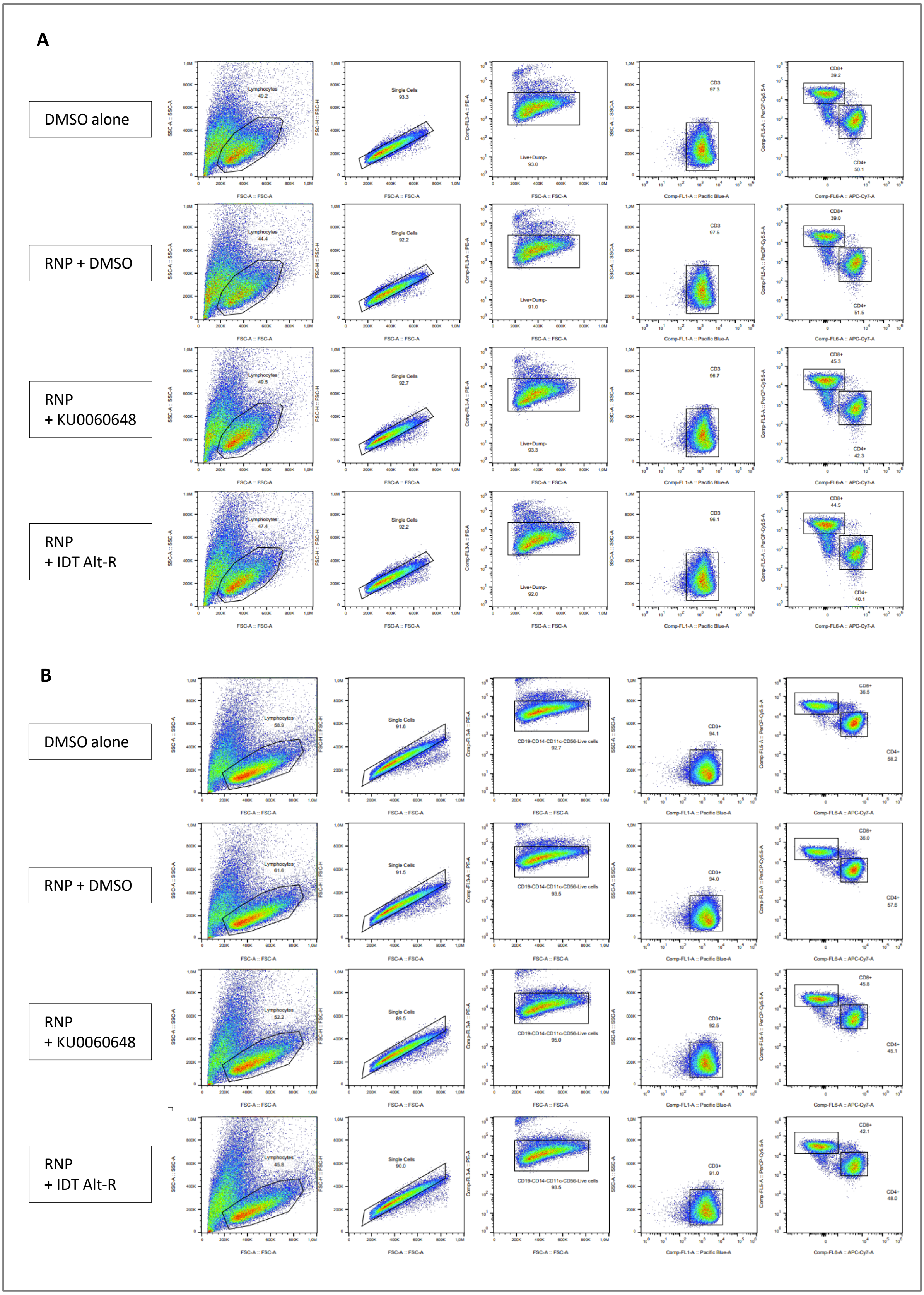

Figure S10. FACS sorting panel for scRNA-seq in DADA2 patient and healthy control

FACS gating strategy of unedited and ADA2-edited CD4+ and CD8+ T cells in (A) HD and (B) DADA2 patient. Cells were nucleofected on day 4 of the platform and treated with 0.5 μM KU0060648, 0.6 μM IDT Alt-R enhancer V2 or DMSO for 24h after nucleofection. Samples were collected for FACS on day 8 of the platform. One independent experiment was performed for all sets of data. Abbreviations: scRNA-seq (single-cell RNA sequencing), HD (healthy donor), RNP (ribonucleoprotein), DMSO (dimethyl sulfoxide), FACS (fluorescence-activated cell sorting), DADA2 (Deficiency of adenosine deaminase 2).

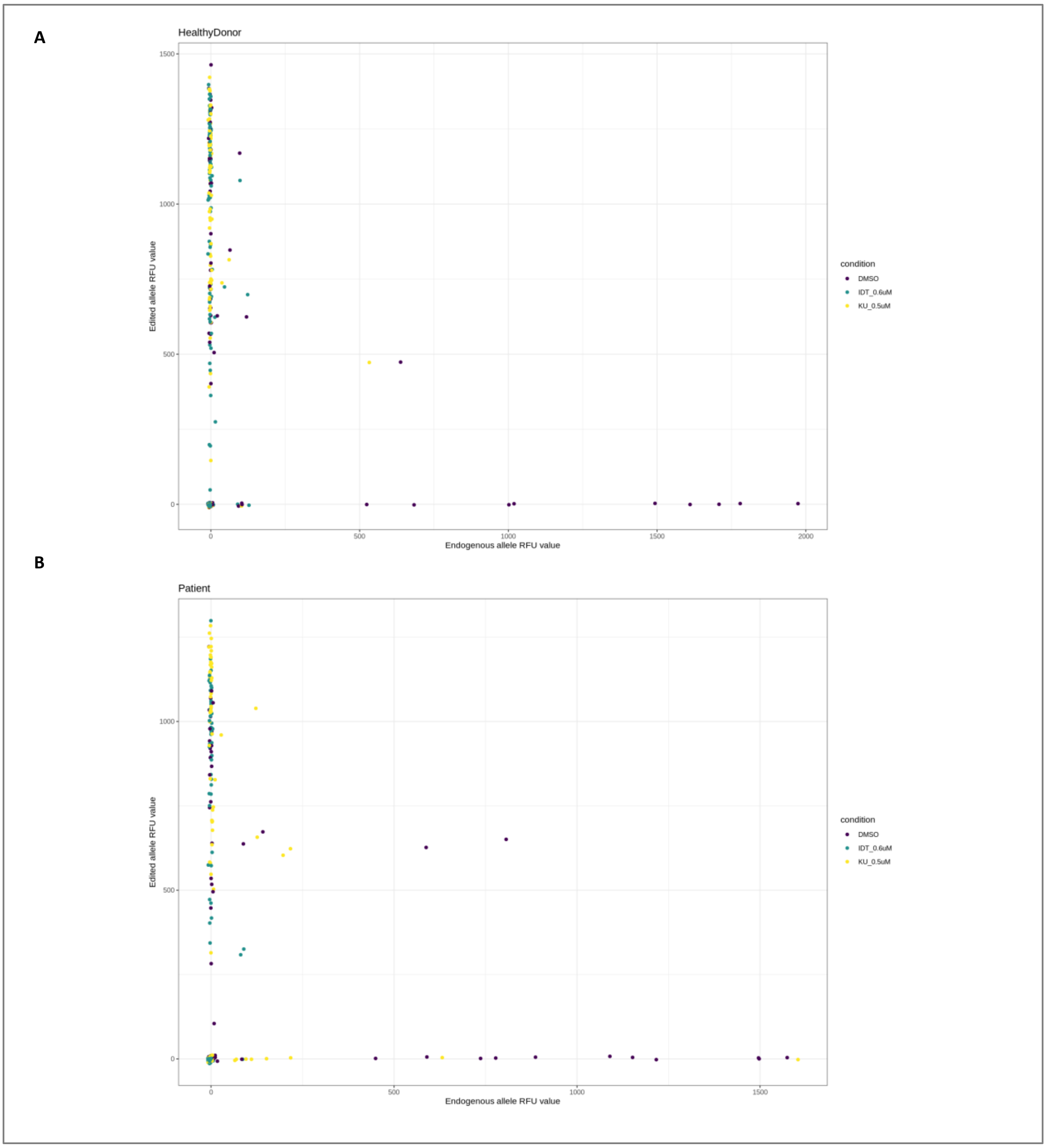

Figure S11. scRNA-seq RT-qPCR plots in healthy control and DADA2 patient

(A) RT-qPCR plots for edited HD and (B) DADA2 patient. Plots show RFU values of edited allele on y axis and endogenous allele on x axis. Each dot is measurement from a single cell. Dots are colored by which condition the cells underwent editing, DMSO in purple, 0.6 µM IDT Alt-R enhancer V2 in green and 0.5µM KU0060648 in yellow. Abbreviations: scRNA-seq (single-cell RNA sequencing), HD (healthy donor), DADA2 (Deficiency of adenosine deaminase 2), RT-qPCR (reverse transcription-qPCR), RFU (relative fluorescence units).

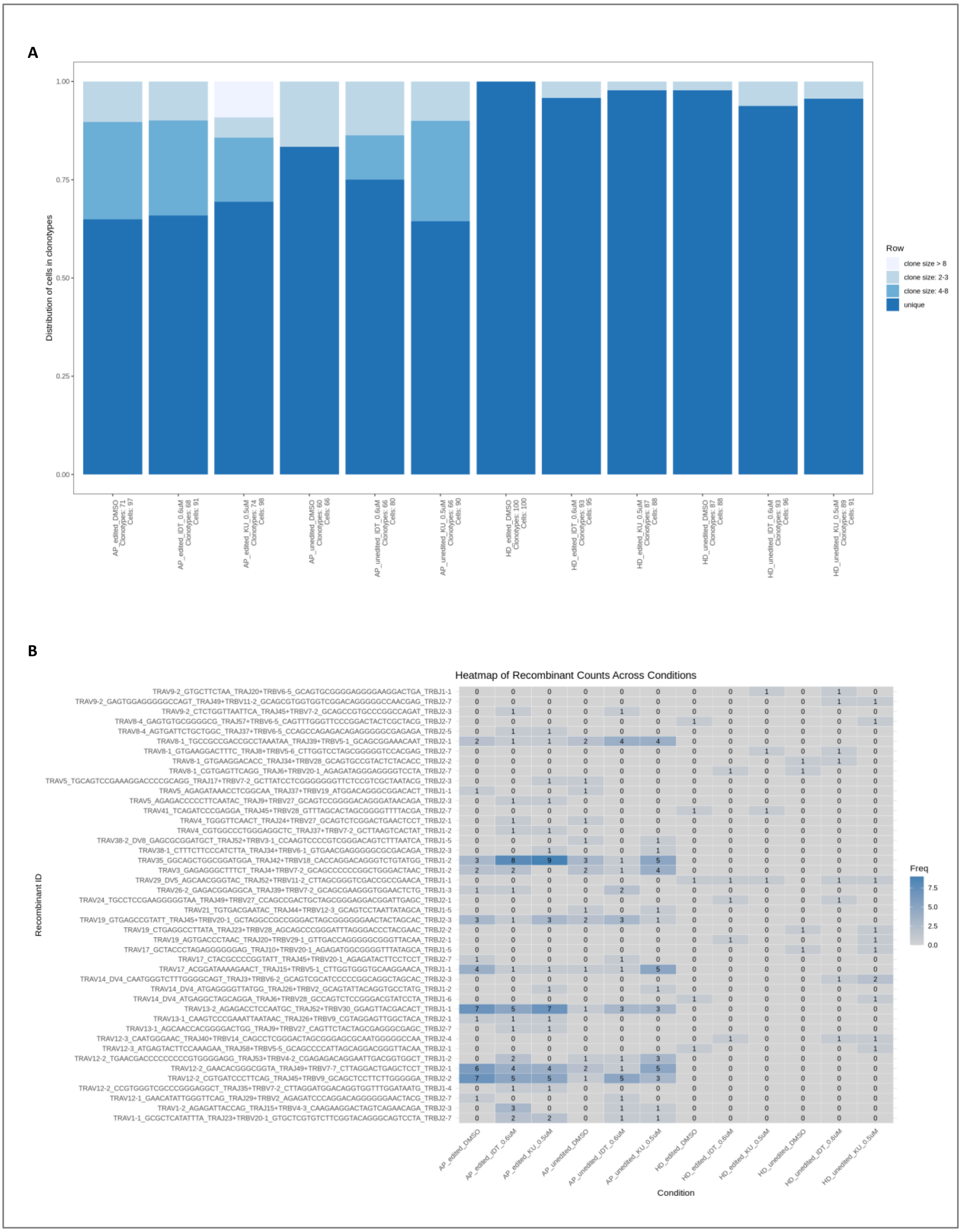

Figure S12. scRNA-seq TCR analysis in DADA2 patient and healthy control

(A) TCR repertoire analysis from scRNA-seq across experimental conditions for DADA2 patient ("AP") and healthy donor ("HD"). (B) Heatmap of recombinant counts across

conditions, reported as frequencies. One independent experiment was performed for all sets of data. Abbreviations: scRNA-seq (single-cell RNA sequencing), TCR (T cell receptor), HD (healthy donor), RNP (ribonucleoprotein), DMSO (dimethyl sulfoxide), DADA2 (Deficiency of adenosine deaminase 2).

A B C

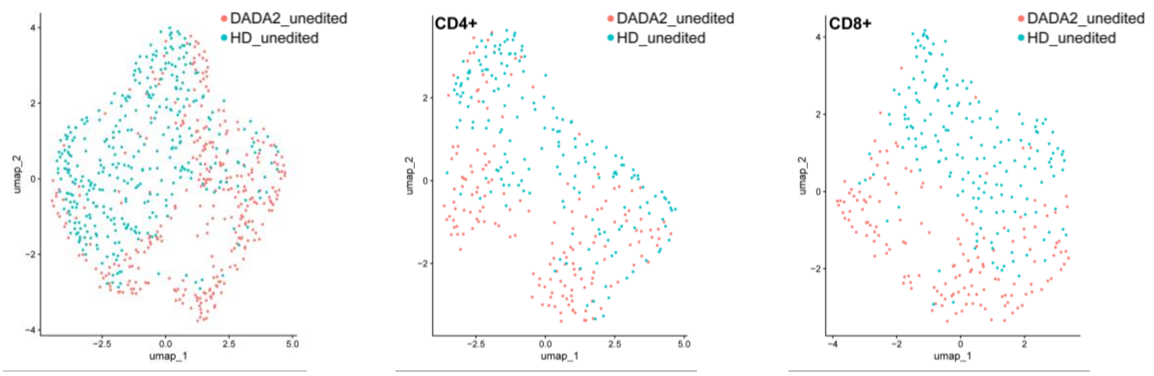

D

Total	P adjusted	NES
INTERFERON_ALPHA_RESPONSE	8.9e−06	-2,11
INTERFERON_GAMMA_RESPONSE	3.7e−03	-1,65
TNFA_SIGNALING_VIA_NFKB	3.7e−03	1,66
MITOTIC_SPINDLE	3.6e−02	-1,5

CD4+	P adjusted	NES
TNFA_SIGNALING_VIA_NFKB	2.1e−05	1,99
INTERFERON_ALPHA_RESPONSE	2.1e−05	-2,09
IL2_STAT5_SIGNALING	2.1e−03	1,70
ALLOGRAFT_REJECTION	2.1e−03	1,69
INTERFERON_GAMMA_RESPONSE	2.6e−03	-1,65
EPITHELIAL_MESENCHYMAL_TRANSITION	5.8e−03	1,7
COAGULATION	1.9e−02	1,66
ESTROGEN_RESPONSE_LATE	3.3e−02	1,51
ANGIOGENESIS	3.3e−02	1,75
KRAS_SIGNALING_UP	3.3e−02	1,49

CD8+	P adjusted	NES
INTERFERON_ALPHA_RESPONSE	4.0e−06	-2,12
INTERFERON_GAMMA_RESPONSE	1.7e−05	-1,86
KRAS_SIGNALING_UP	1.1e−04	-1,88
IL2_STAT5_SIGNALING	1.1e−04	-1,78
MYOGENESIS	1.4e−03	-1,76
MITOTIC_SPINDLE	3.6e−03	-1,59
XENOBIOTIC_METABOLISM	1.9e−02	-1,58
OXIDATIVE_PHOSPHORYLATION	2.5e−02	-1,45
UV_RESPONSE_DN	3.3e−02	1,48
COMPLEMENT	3.7e−02	-1,43

Figure S13. scRNA-seq UMAP plots comparing DADA2 patient to unedited healthy control

UMAP plots generated from scRNA-seq of unedited DADA2 patient for (A) total, (B) CD4+ and (C) CD8+ T cells, compared to unedited HD (DMSO). (D) Hallmark gene set enrichment results for unedited DADA2 patient (total, CD4+ and CD8+) compared to unedited HD

(DMSO). One independent experiment was performed for all sets of data. Abbreviations: scRNA-seq (single-cell RNA sequencing), HD (healthy donor), DADA2 (Deficiency of adenosine deaminase 2), UMAP (Uniform Manifold Approximation and Projection, NES (normalized enrichment score).

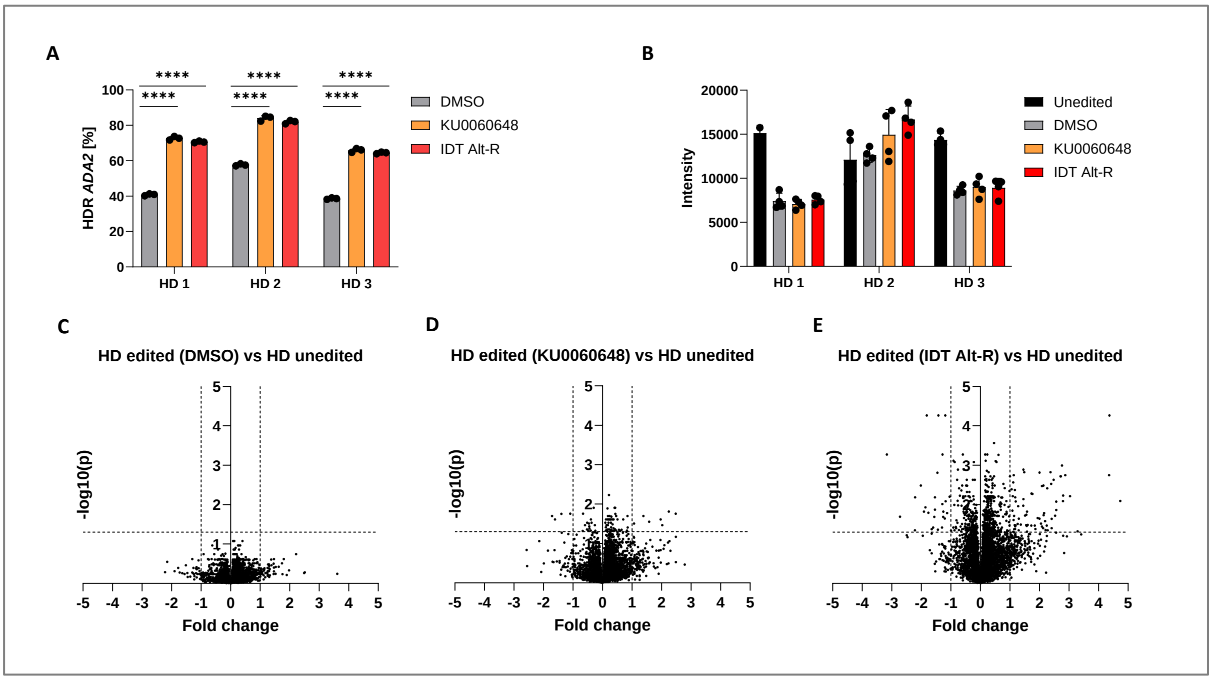

Figure S14. Mass spectrometry analysis of *ADA2*-edited and unedited healthy control T cells

(A) *ADA2* HDR editing in three HDs treated with HDR enhancers (0.5µM KU0060648, 0.6µM IDT Alt-R enhancer V2) or DMSO, assessed by ddPCR (n=3 technical replicates). (B) Abundance of ADA2 protein in three unedited and *ADA2*-edited HDs, reported as intensities (n=4 technical replicates). Comparison of protein expression levels in (C) *ADA2*-edited DMSO-treated HDs to unedited HDs, (D) *ADA2*-edited KU0060648-treated HDs to unedited HDs, (E) *ADA2*-edited IDT Alt-R enhancer V2 -treated HDs to unedited HDs, assessed by mass spectrometry. For (c)-(e), volcano plots were created by reporting protein expression fold change from mean of three HDs on the x axis and –log10 p value on the y axis. One independent experiment was performed for all sets of data. Statistical significance was assessed by one-way

ANOVA with Fisher's LSD test, where ****p<0.0001. Bar denotes mean value, error bars represent ± SD. Bar denotes mean value, error bars represent ± SD. Abbreviations: HDR (homology-directed repair), HD (healthy donor), DMSO (dimethyl sulfoxide), RNP (ribonucleoprotein), ddPCR (Droplet Digital PCR).

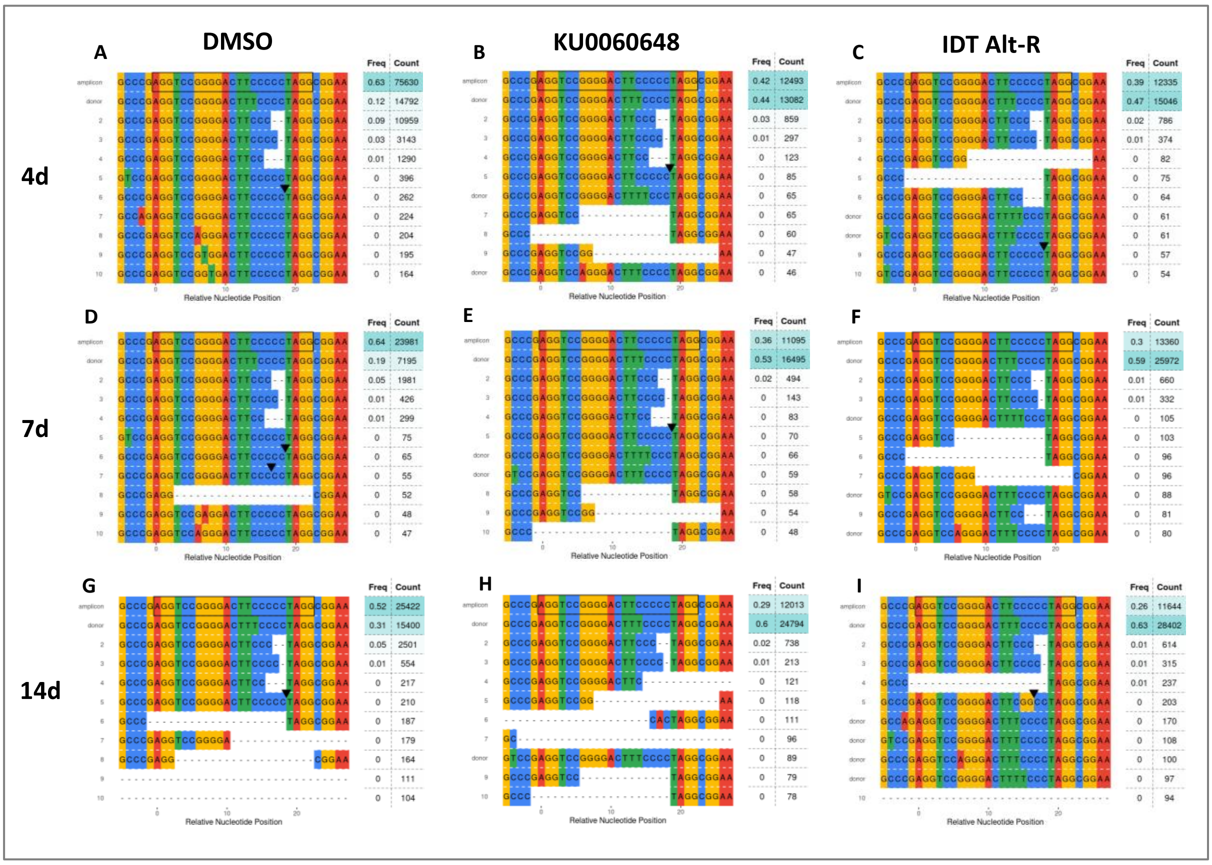

Figure S15. Amplicon sequencing variant plots in Cartilage hair hypoplasia patient CHH 1

Amplicon sequencing variant plots in *RMRP*-corrected CHH patient (CHH 1) treated with HDR enhancing compounds (0.5 µM KU0060648 and 0.6 µM IDT Alt-R enhancer V2) or DMSO, where samples were collected four (A-C), seven (D-F) and fourteen (G-I) days after nucleofection, shown in Fig. 7A. Edits are characterized on the left side of the plot, where "amplicon" is the unedited mutant sequence, "donor" below the "amplicon" is the perfect HDR (mutation correction) and imperfect HDR or indels in the rows below "amplicon". Arrow

indicates an insertion. Frequencies and sequencing counts are reported on the right side of the plot. HDR was assessed by amplicon sequencing, where one representative measurement is shown (n=2 technical replicates). One independent experiment was performed for all sets of data. The patient number corresponds to patient information in Supplemental Table S15. Abbreviations: CHH (Cartilage Hair Hypoplasia).

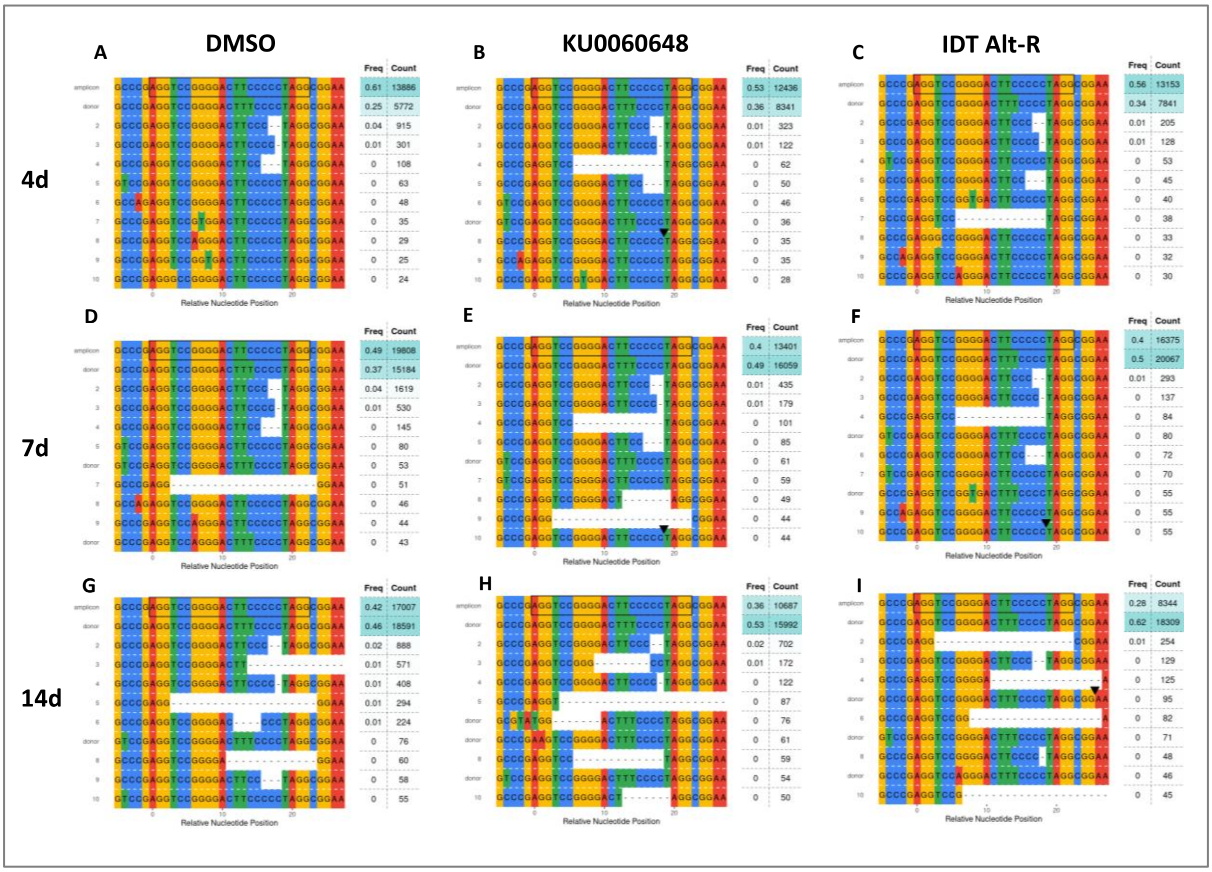

Figure S16. Amplicon sequencing variant plots in Cartilage hair hypoplasia patient CHH 2

Amplicon sequencing variant plots in *RMRP*-corrected CHH patient (CHH 2) treated with HDR enhancing compounds (0.5 µM KU0060648 and 0.6 µM IDT Alt-R enhancer V2) or DMSO, where samples were collected four (A-C), seven (D-F) and fourteen (G-I) days after nucleofection, shown in Fig. 7A. Edits are characterized on the left side of the plot, where "amplicon" is the unedited mutant sequence, "donor" below the "amplicon" is the perfect HDR

(mutation correction) and imperfect HDR or indels in the rows below "amplicon". Arrow indicates an insertion. Frequencies and sequencing counts are reported on the right side of the plot. HDR was assessed by amplicon sequencing, where one representative measurement is shown (n=2 technical replicates). One independent experiment was performed for all sets of data. The patient number corresponds to patient information in Supplemental Table S15. Abbreviations: CHH (Cartilage Hair Hypoplasia).

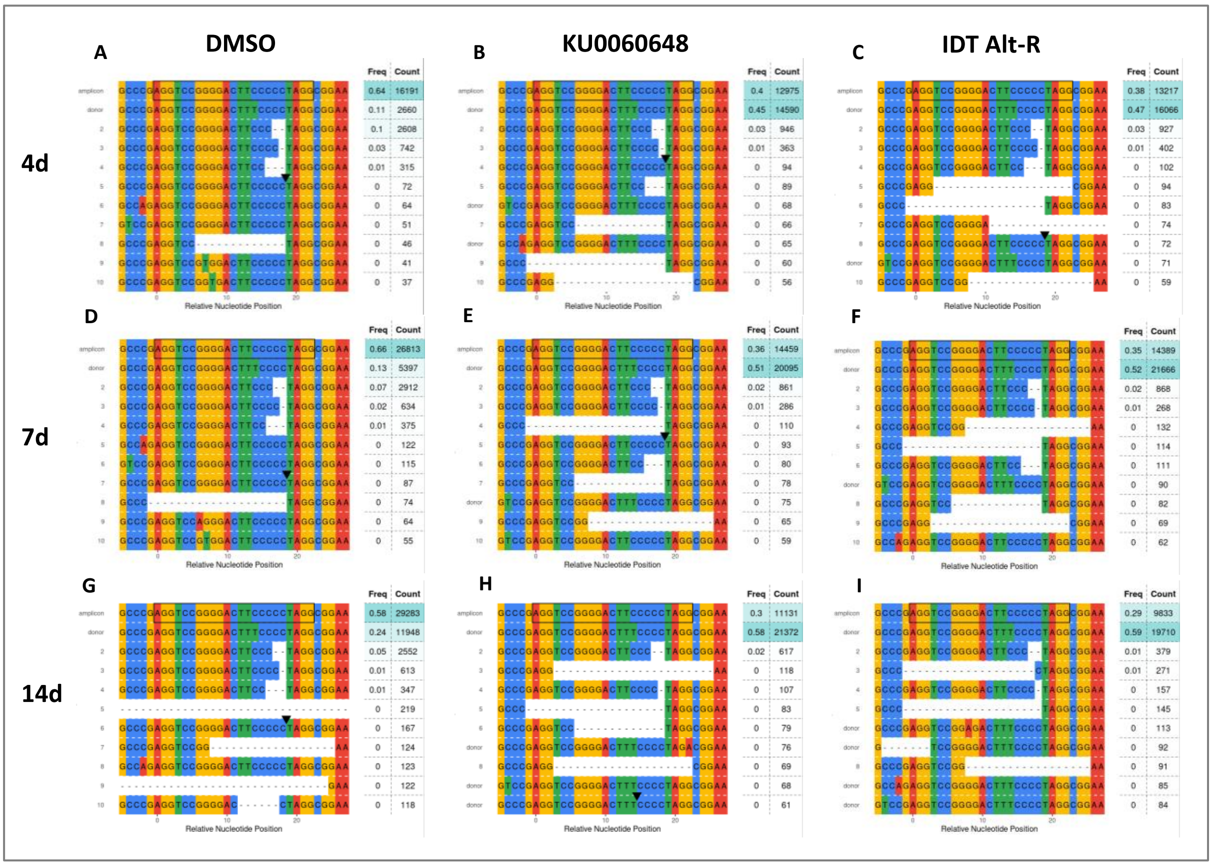

Figure S17. Amplicon sequencing variant plots in Cartilage hair hypoplasia patient CHH 3

Amplicon sequencing variant plots in *RMRP*-corrected CHH patient (CHH 3) treated with HDR enhancing compounds (0.5 µM KU0060648 and 0.6 µM IDT Alt-R enhancer V2) or DMSO, where samples were collected four (A-C), seven (D-F) and fourteen (G-I) days after nucleofection, shown in Fig. 7A. Edits are characterized on the left side of the plot, where

“amplicon” is the unedited mutant sequence, “donor” below the “amplicon” is the perfect HDR (mutation correction) and imperfect HDR or indels in the rows below “amplicon”. Arrow indicates an insertion. Frequencies and sequencing counts are reported on the right side of the plot. HDR was assessed by amplicon sequencing, where one representative measurement is shown (n=2 technical replicates). One independent experiment was performed for all sets of data. The patient number corresponds to patient information in Supplemental Table S15. Abbreviations: CHH (Cartilage Hair Hypoplasia).

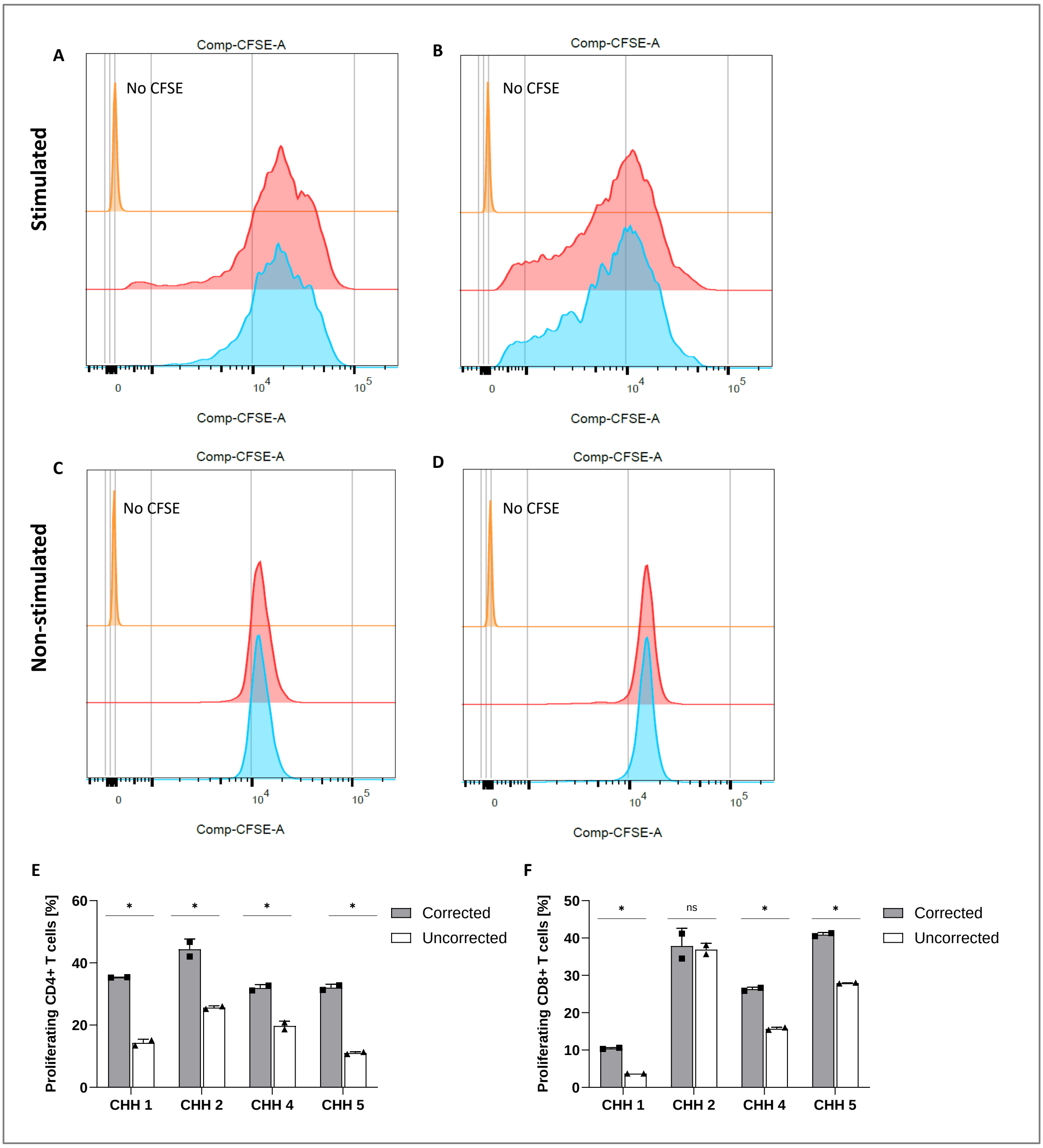

Figure S18. CFSE T cell proliferation assay in healthy control and Cartilage hair hypoplasia patient T cells

T cell proliferation assay in stimulated, mock electroporated (A) CD4+ and (B) CD8+ T cells, assessed by flow cytometry. T cell proliferation assay in unstimulated PBMCs in (C) CD4+ and (D) CD8+ T cells, assessed by flow cytometry. Frequency of proliferating (E) CD4+ and (F) CD8+ T cells in corrected and uncorrected CHH patients (CHH 1-5) shown in in Fig. 7C-

D were assessed by flow cytometry. CFSE signal in unstimulated PBMCs (c-d) were used as a gating control to assess frequency of proliferation in patients. The patient number corresponds to patient information in Supplemental Table S15. One independent experiment was performed for all sets of data. Bar denotes mean value, error bars represent ± SD. Statistical significance was assessed by performing unpaired t-test where * shows significance ($p<0.05$). Abbreviations: CHH (Cartilage hair hypoplasia).

SUPPLEMENTAL TABLES

Table S1: Preclinical evidence of T cell editing in IEI patients

IEI (gene)	Editing approach	Target cell type	Delivery strategy	Study highlights
CTLA4 Haploinsufficiency (*CTLA4*)[1]	cDNA knock-in to restore CTLA-4 expression	Patient CD4+ T cells and T_{regs}	Cas9 RNP + AAV6 HDR donor	Rescued CTLA-4 expression in CD4+ T cells; restoration of transendocytosis in T_{regs}. Engraftment and prevention of lymphoproliferation *in vivo* in a murine model of CTLA insufficiency.
Familial Hemophagocytic Lymphohistiocytosis (*PRF1, UNC13D*)[2]	HDR-mediated precise repair of cytotoxicity-related genes	Patient $CD8^+$ T cells	Cas9 RNP + AAV donor	Restored perforin or granule exocytosis; recovery of cytotoxic activity against target cells
IL-2Rα deficiency (*IL2RA/CD25*)[3]	Precise repair of pathogenic *IL2RA* variants	Patient CD4+ and CD8+ T cells	Cas9 RNP + ssDNA/dsDNA donor (non-viral)	Restored IL-2 expression; increased STAT5 phosphorylation
IPEX (*FOXP3*)[4]	cDNA knock-in to restore FOXP3 expression	Patient T_{eff} and T_{reg}	Cas9 RNP + rAAV6 HDR donor	Restored FOXP3 expression; regulation of T_{eff} proliferation; recovered suppressive T_{reg} function
X-linked Hyper-IgM Syndrome (*CD40LG*)[5]	cDNA knock-in to restore CD40LG expression	Patient $CD4^+$ T cells	Cas9 RNP + AAV6/IDLV donor	Restored CD40L expression; rescued B-cell help/class-switching
X-linked Hyper-IgM Syndrome (*CD40LG*)[6]	cDNA knock-in to restore CD40LG expression	Patient $CD4^+$ T cells	Cas9 RNP + IDLV donor	GMP-compatible & scalable manufacturing of edited T cells; restored CD40LG expression; engraftment upon xenotransplantation
X-linked Lymphoproliferative Disease (*SH2D1A*)[7]	cDNA knock-in to restore SAP expression	Patient $CD4^+$ and $CD8^+$ T cells	Cas9 RNP + AAV6 donor	Restored SAP expression; restored sensitivity to restimulation-induced

				cell death; restored T:B cell signalling; T_{FH} support; improved cytotoxic functions
X-linked Lymphoproliferative Disease (*SH2D1A*)[8]	Stable SAP expression using precise promoter-driven expression cassette	Patient $CD4^+$ and $CD8^+$ T cells	Lentiviral vectors	Restored SAP expression; restored sensitivity to restimulation-induced cell death

Table S2: gRNA sequences for *ADA2*, *AIRE*, *RMRP* and *STAT1*

Locus	gRNA	gRNA sequence 5'→3'	gRNA orientation	gRNA length (bp)
ADA2	g#1	ttccaagtggattctgctgg	FWD	20
ADA2	g#2 (WT)	gctggaggattatcggaagc	FWD	20
ADA2	g#2 (MUT)	gctggaggattatcagaagc	FWD	20
ADA2	g#3 (WT)	tgctggaggattatcggaag	FWD	20
ADA2	g#3 (MUT)	tgctggaggattatcagaag	FWD	20
ADA2	g#4	ggattctgctggaggattat	FWD	20
ADA2	g#5	atgttccaagtggattctgc	FWD	20
ADA2	g#6	catcagaaaaatgttccaag	FWD	20
ADA2	g#7	atcctccagcagaatccact	REV	20
AIRE	g#1	cagcagtggcccgaagcctc	FWD	20
AIRE	g#2 (MUT)	gaagcctctggtttgagcca	FWD	20
AIRE	g#3 (MUT)	aagcctctggtttgagccaa	FWD	20
AIRE	g#4 (MUT)	gtttgagccaagggagccca	FWD	20
AIRE	g#5 (MUT)	ggtttgagccaagggagccc	FWD	20
AIRE	g#6	aacaaggcccgcagcagcag	FWD	20
AIRE	g#7	cttcgggccactgctgctgc	REV	20
AIRE	g#8	gcttcgggccactgctgctg	REV	20
AIRE	g#9 (MUT)	tggctcaaaccagaggcttc	REV	20
AIRE	g#10 (MUT)	ttggctcaaaccagaggctt	REV	20
AIRE	g#11 (WT)	gctcccttggctcgaaccag	REV	20
AIRE	g#11 (MUT)	gctcccttggctcaaaccag	REV	20
AIRE	g#12 (MUT)	gctcccttggctcaaaccag	REV	20
AIRE	g#13	ggcagcgccctgggctccct	REV	20
AIRE	g#14	gcttacgggggcagcgccct	REV	20
AIRE	g#15	tgcttacgggggcagcgccc	REV	20
AIRE	g#16	ggaaggtcaggtgcttacgg	REV	20
AIRE	g#17	gggaaggtcaggtgcttacg	REV	20
AIRE	g#18	agggaaggtcaggtgcttac	REV	20

AIRE	g#19	cagggaaggtcaggtgctta	REV	20
RMRP	g#1	cactctctgcccgaggtccg	FWD	20
RMRP	g#2	tgtctacgtgcgtatgcacg	FWD	20
RMRP	g#3	cacgtggcactctctgcccg	FWD	20
RMRP	g#4	ggcactctctgcccgaggtc	FWD	20
RMRP	g#5	gcactctctgcccgaggtcc	FWD	20
RMRP	g#6 (MUT)	aggtccggggacttccccct	FWD	20
RMRP	g#7 (MUT)	tccggggacttcccccctagg	FWD	20
RMRP	g#8 (MUT)	ggacttcccccctaggcggaa	FWD	20
RMRP	g#9 (WT)	gactttcccctaggcggaaa	FWD	20
RMRP	g#9 (MUT)	gacttcccccctaggcggaaa	FWD	20
RMRP	g#10 (MUT)	acttcccccctaggcggaaag	FWD	20
RMRP	g#11 (MUT)	tcccccctaggcggaaagggg	FWD	20
RMRP	g#12	gagtcctcagtgtgtagcct	FWD	20
RMRP	g#13 (MUT)	gggggaagtccccggacctc	REV	20
RMRP	g#14 (MUT)	agggggaagtccccggacct	REV	20
RMRP	g#15 (MUT)	tccgcctagggggaagtccc	REV	20
RMRP	g#16	ttcctcccctttccgcctag	REV	20
STAT1	g#1 (MUT)	gcacacaagagtgatgaaca	FWD	20
STAT1	g#2 (MUT)	cacaagagtgatgaacatgg	FWD	20
STAT1	g#3	aacatggaggagtccaccaa	FWD	20

Table S3: ssODN sequences for *ADA2*, *AIRE*, *RMRP* and *STAT1*

**Locus**	**Repair strategy**	**ssODN symmetry**	**ssODN direction (5'→3')**	**ssODN sequence (100 bp)**
ADA2	WT/MUT →SNP	Left 40nt	fwd	cccaagggggatcatgcagttcagatttgctcacccaactccccgtccatcagaaaaatgttccaagtggattctgctggaggactacagaaagcgggtg
ADA2	WT/MUT →SNP	Left 30nt	fwd	atcatgcagttcagatttgctcacccaactccccgtccatcagaaaaatgttccaagtggattctgctggaggactacagaaagcgggtgcagaacgtca
ADA2	WT/MUT →SNP	Left 20nt	fwd	tcagatttgctcacccaactccccgtccatcagaaaaatgttccaagtggattctgctggaggactacagaaagcgggtgcagaacgtcactgagtttga
ADA2	WT/MUT →SNP	Left 10nt	fwd	tcacccaactccccgtccatcagaaaaatgttccaagtggattctgctggaggactacagaaagcgggtgcagaacgtcactgagtttgatgacaggtga
ADA2	WT/MUT →SNP	Middle	fwd	ccccgtccatcagaaaaatgttccaagtggattctgctggaggactacagaaagcgggtgcagaacgtcactgagtttgatgacaggtgagtagtagttc
ADA2	WT/MUT →SNP	Right 10nt	fwd	cagaaaaatgttccaagtggattctgctggaggactacagaaagcgggtgcagaacgtcactgagtttgatgacaggtgagtagtagttcagaaagcaca

ADA2	WT/MUT →SNP	Right 20nt	fwd	ttccaagtggattctgctggaggactacagaaagcgggtgcagaacgtcactgagtttgatgacaggtgagtagtagttcagaaagcacatgtcccaggc
ADA2	WT/MUT →SNP	Right 30nt	fwd	ttctgctggaggactacagaaagcgggtgcagaacgtcactgagtttgatgacaggtgagtagtagttcagaaagcacatgtcccaggcctgtcatgggg
ADA2	WT/MUT →SNP	Right 40nt	fwd	aggactacagaaagcgggtgcagaacgtcactgagtttgatgacaggtgagtagtagttcagaaagcacatgtcccaggcctgtcatggggtggcagtgg
AIRE	WT/MUT →SNP	Left 40nt	rev	cccttggcacgtaccaaaggcttcgggccactgctgctgcgggccttgttcttcccactgccggagtcttcgaacttgctgggagtgtagaactccccgc
AIRE	WT/MUT →SNP	Left 30nt	rev	gccctgggctcccttggcacgtaccaaaggcttcgggccactgctgctgcgggccttgttcttcccactgccggagtcttcgaacttgctgggagtgtag
AIRE	WT/MUT →SNP	Left 20nt	rev	cgggggcagcgccctgggctcccttggcacgtaccaaaggcttcgggccactgctgctgcgggccttgttcttcccactgccggagtcttcgaacttgct
AIRE	WT/MUT →SNP	Left 10nt	rev	caggtgcttacgggggcagcgccctgggctcccttggcacgtaccaaaggcttcgggccactgctgctgcgggccttgttcttcccactgccggagtctt
AIRE	WT/MUT →SNP	Middle	rev	cagggaaggtcaggtgcttacgggggcagcgccctgggctcccttggcacgtaccaaaggcttcgggccactgctgctgcgggccttgttcttcccactg
AIRE	WT/MUT →SNP	Right 10nt	rev	ccaggctccccagggaaggtcaggtgcttacgggggcagcgccctgggctcccttggcacgtaccaaaggcttcgggccactgctgctgcgggccttgtt
AIRE	WT/MUT →SNP	Right 20nt	rev	gcatcaagagccaggctccccagggaaggtcaggtgcttacgggggcagcgccctgggctcccttggcacgtaccaaaggcttcgggccactgctgctgc
AIRE	WT/MUT →SNP	Right 30nt	rev	ggggcgggggggcatcaagagccaggctccccagggaaggtcaggtgcttacgggggcagcgccctgggctcccttggcacgtaccaaaggcttcgggcca
AIRE	WT/MUT →SNP	Right 40nt	rev	cgctgttcctggggcgggggggcatcaagagccaggctccccagggaaggtcaggtgcttacgggggcagcgccctgggctcccttggcacgtaccaaagg
RMRP	WT→SNP	Left 40nt	fwd	gatacgcttcttggcggactttggagtgggaagcggggaatgtctacgtgcgtatgcacgtggcactctctgcccgaggtccggggacttccacataggc
RMRP	WT→SNP	Left 30nt	fwd	ttggcggactttggagtgggaagcggggaatgtctacgtgcgtatgcacgtggcactctctgcccgaggtccggggacttccacataggcggaaagggga
RMRP	WT→SNP	Left 20nt	fwd	ttggagtgggaagcggggaatgtctacgtgcgtatgcacgtggcactctctgcccgaggtccggggacttccacataggcggaaaggggaggaacagagt
RMRP	WT→SNP	Left 10nt	fwd	aagcggggaatgtctacgtgcgtatgcacgtggcactctctgcccgaggtccggggacttccacataggcggaaaggggaggaacagagtcctcagtgtg
RMRP	WT→SNP	Middle	fwd	tgtctacgtgcgtatgcacgtggcactctctgcccgaggtccggggacttccacataggcggaaaggggaggaacagagtcctcagtgtgtagcctagga
RMRP	WT→SNP	Right 10nt	fwd	cgtatgcacgtggcactctctgcccgaggtccggggacttccacataggcggaaaggggaggaacagagtcctcagtgtgtagcctaggatacaggcctt
RMRP	WT→SNP	Right 20nt	fwd	tggcactctctgcccgaggtccggggacttccacataggcggaaaggggaggaacagagtcctcagtgtgtagcctaggatacaggccttcagcacgaac
RMRP	WT→SNP	Right 30nt	fwd	tgcccgaggtccggggacttccacataggcggaaaggggaggaacagagtcctcagtgtgtagcctaggatacaggccttcagcacgaaccacgtcctca
RMRP	WT→SNP	Right 40nt	fwd	ccggggacttccacataggcggaaaggggaggaacagagtcctcagtgtgtagcctaggatacaggccttcagcacgaaccacgtcctcagcttcacaga
RMRP	MUT→SNP	Middle	fwd	tgtctacgtgcgtatgcacgtggcactctctgcccgaggtccggggactttcacataggcggaaaggggaggaacagagtcctcagtgtgtagcctagga
RMRP	MUT→WT	Middle	fwd	tgtctacgtgcgtatgcacgtggcactctctgcccgaggtccggggactttcccctaggcggaaaggggaggaacagagtcctcagtgtgtagcctagga
STAT1	MUT→ SNP	Left 30nt	fwd	taatagttggaagacttttcagcatttctttcctatattgtatagatttaggaagttcaacattttgggcacgcacacgaaggttatgaacatggaggag

STAT1	MUT→ SNP	Left 20nt	fwd	aagacttttcagcatttctttcctatattgtatagatttaggaagttcaacattttgggcacgcacacgaaggttatgaacatggaggagtccaccaatg
STAT1	MUT→ SNP	Left 10nt	fwd	agcatttctttcctatattgtatagatttaggaagttcaacattttgggcacgcacacgaaggttatgaacatggaggagtccaccaatggcagtctggc
STAT1	MUT→ SNP	Middle	fwd	tcctatattgtatagatttaggaagttcaacattttgggcacgcacacgaaggttatgaacatggaggagtccaccaatggcagtctggcggctgaattt
STAT1	MUT→ SNP	Right 10nt	fwd	tatagatttaggaagttcaacattttgggcacgcacacgaaggttatgaacatggaggagtccaccaatggcagtctggcggctgaatttcggcacctgg
STAT1	MUT→ SNP	Right 20nt	fwd	ggaagttcaacattttgggcacgcacacgaaggttatgaacatggaggagtccaccaatggcagtctggcggctgaatttcggcacctggtagggacatc
STAT1	MUT→ SNP	Right 30nt	fwd	cattttgggcacgcacacgaaggttatgaacatggaggagtccaccaatggcagtctggcggctgaatttcggcacctggtagggacatcagtttcctct

Table S4: Markers used for immune cell characterization

Marker	Color	Clone	Vendor	Catalog	Dilution
CD14	PerCP-Cy5.5	61D3	eBioscoence	45-0149-42	1:200
CD15	FITC	3G8	Biolegend	302001	1:200
CD56	FITC	NCAM16.2	BD	664524	1:200
CD4	AlexaFluor700	RPA-T4	BD Pharmingen	557922	1:200
CD3	BV421	UCHT1	BD Horizon	562426	1:200
CD20	BV786	2H7	Biolegend	302356	1:100
CD8	PE	4B9	eBioscience	12-0087-42	1:100
LiveDead near IR	N/A	N/A	Thermo Fisher	L34992	1:1000

Table S5: Markers used for CD4+ and CD8+ T cell sorting panel

Marker	Color	Clone	Vendor	Catalog	Dilution
CD19	PE	HIB19	BioLegend	302207	1:100
CD14	PE	HCD14	BioLegend	325605	1:100
CD11c	PE	3.9	BioLegend	301605	1:100
CD56	PE	HCD56	BioLegend	318305	1:100
CD3	Pacific Blue	SK7	BioLegend	344823	1:50
CD4	APC-Cy7	OKT4	BioLegend	317417	1:50
CD8a	PerCP-Cy5.5	RPA-T8	BioLegend	301031	1:100
LIVE/DEAD Fixable Red	N/A	N/A	Invitrogen	L34971	1:500

Table S6: Antibodies and reagents used for CFSE T cell proliferation assay

Marker	Color	Clone	Vendor	Catalog	Dilution
CFSE	CFSE	N/A	Invitrogen	C34554A	1µM
CD19	Pacific Blue	HIB19	Biolegend	302232	1:200
CD14	Pacific Blue	M5E2	Biolegend	301828	1:200
CD56	Pacific Blue	MEM-188	Biolegend	304629	1:200
CD11c	V450	B-ly6	BD Biosciences	560369	1:200
Live/Dead Fixable Violet	N/A	N/A	Invitrogen	L34963	1:500
CD8a	Alexa Fluor 594	RPA-T8	Biolegend	301056	1:100
CD4	APC-Cy7	OKT4	BioLegend	317417	1:100

Table S7. Antibodies used for assessment of phosphorylated STAT1

Marker	Color	Clone	Vendor	Catalog	Dilution
CD3	Alexa Flour 488	UCHT1	Biolegend	300415	1:500
CD19	Pacific Blue	HIB19	Biolegend	302232	1:1000
CD14	Pacific Blue	M5E2	Biolegend	301828	1:1000
CD56	Pacific Blue	MEM-188	Biolegend	304629	1:1000
CD11c	V450	B-ly6	BD Biosciences	560369	1:1000
Live/Dead Fixable Violet	N/A	N/A	Invitrogen	L34963	1:500
pSTAT1 (Y701)	Alexa Fluor 647		BD Biosciences	612597	1:10

Table S8: ddPCR oligos for ADA2, AIRE, RMRP and STAT-1

**Target gene**	**ddPCR primer fwd**	**ddPCR primer rev**	**ddPCR probe reference**	**ddPCR probe HDR**	**ddPCR probe NHEJ**
ADA2	GGTGAGGAATGTCACCTACA	GTACCAAGGGAGACACCTAC	**WT&MUT:**<br><br>/5'FAM/GCCACATCTGTTTCACCCCA/3'BHQ_1/	**WT/MUT→SNP:**<br>/5'HEX/CTGGAGGACTACAGAAAGCGG/3'BHQ_1/	**WT→SNP:**<br>/5'HEX/ATTATCGGAAGCGGGTGCAGA/3'BHQ_1/<br><br>**MUT→SNP:**<br>/5'HEX/ATTATCAGAAGCGGGTGCAGA/3'BHQ_1/

AIRE	TCTA CACT CCCA GCAA GTTC	GGAA GGTCA GGTGC TTACG	**WT&MUT:** /5'FAM/TCCGGCAGT GGGAAGAACAA/3'B HQ_1/	**WT/MUT→SNP:** /5'HEX/AAGCCTTTGG TACGTGCCAAG/3'BH Q_1/	**WT→SNP:** /5'HEX/CGAAGCCTC TGGTTCGAGC/3'BHQ _1/ **MUT→SNP:** /5'HEX/CGAAGCCTC TGGTTTGAGC/3'BHQ _1/
RMRP	GCTT CTTG GCGG ACTT TG	ATACT ACTCT GTGAA GCTGA GG	**WT&MUT:** /5'FAM/TGGGAAGCG GGGAATGTCTA/3'B HQ_1/	**WT→SNP:** /5'HEX/GGACTTCCAC ATAGGCGGAA/3'BHQ _1/ **MUT→SNP:** /5'HEX/GGACTTTCAC ATAGGCGGAA/3'BHQ _1/	**WT→SNP:** /5'HEX/ACTTTCCCCT AGGCGGAAAG/3'BH Q_1/ **MUT→SNP:** /5'HEX/ACTTCCCCCT AGGCGGAAAG/3'BH Q_1/
STAT1	ACGT GACG TACT TTAC GCTA T	GAAAC TGATG TCCCT ACCAG G	**WT & MUT:** /5’FAM/AGTTGGAAG ACTTTTCAGCATTTC T/3'BHQ_1/	**MUT→SNP:** /5’HEX/ACGCACACG AAGGTTATGAACA/3' BHQ_1/	**MUT→SNP:** /5’HEX/AAGAGTGAT GAACATGGAGGAGT /3'BHQ_1/
CASC11 (external reference gene)	AGGT GGCT GGAA ACTT GT	GGAGC AACCA ATCGC TATG	**All edited loci:** /5’FAM/CCTCGGACG CTCCTGCTCCT/3'BH Q_1/	N/A	N/A

Table S9: Amplicon-seq first PCR components

Reagent	Vendor	Final concentration
Nuclease-free water	Ambion	Add up to 20
5× Phusion GC Buffer	Thermo Fisher Scientific	1X
10 mM dNTPs	Thermo Fisher Scientific	200 µM
10 µM Primer fwd	IDT	0.5 µM
10 µM Primer rev	IDT	0.5 µM
Betaine	Sigma Aldrich	1 M
Phusion Hot Start II DNA Polymerase	Thermo Fisher Scientific	0.02 U/µl
Template DNA	N/A	100 ng

Table S10: Amplicon sequencing second PCR primers

i5-PCR Index 9	**AAT GAT ACG GCG ACC ACC GAG ATC TA**TTGCTTGC*AC ACT CTT TCC CTA CAC GAC GCT CTT CCG ATC* T*
i5-PCR Index 10	**AAT GAT ACG GCG ACC ACC GAG ATC TA**GAGAGGTT*AC ACT CTT TCC CTA CAC GAC GCT CTT CCG ATC* T*
i5-PCR Index 11	**AAT GAT ACG GCG ACC ACC GAG ATC TA**ACCTGGTT*AC ACT CTT TCC CTA CAC GAC GCT CTT CCG ATC* T*
i5-PCR Index 13	**AAT GAT ACG GCG ACC ACC GAG ATC TA**CGGAACAA*AC ACT CTT TCC CTA CAC GAC GCT CTT CCG ATC* T*
i5-PCR Index A505	**AAT GAT ACG GCG ACC ACC GAG ATC TA**CTAATCGA*AC ACT CTT TCC CTA CAC GAC GCT CTT CCG ATC* T*
i5-PCR Index A506	**AAT GAT ACG GCG ACC ACC GAG ATC TA**CTAGAACA*AC ACT CTT TCC CTA CAC GAC GCT CTT CCG ATC* T*
i5-PCR Index A507	**AAT GAT ACG GCG ACC ACC GAG ATC TA**TAAGTTCC*AC ACT CTT TCC CTA CAC GAC GCT CTT CCG ATC* T*
i5-PCR Index A508	**AAT GAT ACG GCG ACC ACC GAG ATC TA**TAGACCTA*AC ACT CTT TCC CTA CAC GAC GCT CTT CCG ATC* T*
i7-PCR Index 13	**CAA GCA GAA GAC GGC ATA CGA GAT**TTCCTCCT*G TGA CTG GAG TTC AGA CGT GTG CTC TTC CGA TC*T*
i7-PCR Index 14	**CAA GCA GAA GAC GGC ATA CGA GAT**TGCTTGCT*G TGA CTG GAG TTC AGA CGT GTG CTC TTC CGA TC*T*
i7-PCR Index 15	**CAA GCA GAA GAC GGC ATA CGA GAT**GGTGATGA*G TGA CTG GAG TTC AGA CGT GTG CTC TTC CGA TC*T*
i7-PCR Index 16	**CAA GCA GAA GAC GGC ATA CGA GAT**AACCTACG*G TGA CTG GAG TTC AGA CGT GTG CTC TTC CGA TC*T*
i7-PCR Index A705	**CAA GCA GAA GAC GGC ATA CGA GAT**ACCCAGCA*G TGA CTG GAG TTC AGA CGT GTG CTC TTC CGA TC*T*
i7-PCR Index A706	**CAA GCA GAA GAC GGC ATA CGA GAT**AACCCCTC*G TGA CTG GAG TTC AGA CGT GTG CTC TTC CGA TC*T*
i7-PCR Index A707	**CAA GCA GAA GAC GGC ATA CGA GAT**CCCAACCT*G TGA CTG GAG TTC AGA CGT GTG CTC TTC CGA TC*T*
i7-PCR Index A708	**CAA GCA GAA GAC GGC ATA CGA GAT**CACCACAC*G TGA CTG GAG TTC AGA CGT GTG CTC TTC CGA TC*T*
i7-PCR Index A709	**CAA GCA GAA GAC GGC ATA CGA GAT**GAAACCCA*G TGA CTG GAG TTC AGA CGT GTG CTC TTC CGA TC*T*
i7-PCR Index A710	**CAA GCA GAA GAC GGC ATA CGA GAT**TGTGACCA*G TGA CTG GAG TTC AGA CGT GTG CTC TTC CGA TC*T*
i7-PCR Index A711	**CAA GCA GAA GAC GGC ATA CGA GAT**AGGGTCAA*G TGA CTG GAG TTC AGA CGT GTG CTC TTC CGA TC*T*
i7-PCR Index A712	**CAA GCA GAA GAC GGC ATA CGA GAT**AGGAGTGG*G TGA CTG GAG TTC AGA CGT GTG CTC TTC CGA TC*T*

Table S11: Amplicon-seq second PCR components

**Reagent**	**Vendor**	**Final concentration**
Nuclease-free water	Ambion	Add up to 20

5× Phusion GC Buffer	Thermo Fisher Scientific	1X
10 mM dNTPs	Thermo Fisher Scientific	200 µM
10 µM Primer fwd	IDT	0.25 µM
10 µM Primer rev	IDT	0.25 µM
Betaine	Sigma Aldrich	1 M
Phusion Hot Start II DNA Polymerase	Thermo Fisher Scientific	0.02 U/µl
Template DNA	N/A	5 ng

Table S12: List of HDR enhancing compounds and tested concentrations

**Compound**	**Conc 1 (µM)**	**Conc 2 (µM)**	**Conc 3 (µM)**
ABT263 [9]	0,25	0,5	1
AICAR[10]	10	20	40
B02 [10]	10	20	40
Brefeldin A[11]	0,05	0,1	0,2
Entinostat[12]	2,5	5	10
EPZ5676[13]	0,05	0,1	0,2
IC86621[14]	100	200	400
KU0060648[15,16]	0,125	0,25	0,5
KU55933[17,18]	1,5	3	6
L755507[11]	2,5	5	10
Mirin[17]	1,5	3	6
MLN4924[10]	0,25	0,5	1
M3814[19]	1	2	4
Nexturastat A[20]	1,25	2,5	5
NSC 15520[10]	2,5	5	10
NSC 19630[10]	0,5	1	2
NU7026[10]	10	20	40
NU7441[16]	1	2	4
Panobinostat[12]	0,05	0,1	0,2

PFM01[17]	5	10	20
Resveratrol[21]	0,5	1	25
Ricolinostat[20]	1,25	2,5	5
Romidepsin[22,23]	0,01	0,025	0,1
RS-1[24]	5	10	20
Rucaparib[25,26]	2,5	5	10
SCR7 pyrazine	2,5	1	5
STL127705[10,27]	2,5	5	10
TDRL-505[17]	10	20	40
Trichostatin A[10,28]	0,005	0,01	0.1
Valproic acid[23,29]	5	10	20
Wortmannin[30]	0,01	0,02	0,04
Crispy mix[10]*	*	Not tested	Not tested
IDT ALT-R enhancer V2	1	Not tested	Not tested

*20 µM NU7026, 0.01 µM Trichostatin A, 0.5 µM MLN4924, 5uM NCS15520

Table S13: List of cell cycle inhibitors and tested concentrations

**Compound**	**Conc 1 (µM)**	**Conc 2 (µM)**	**Conc 3 (µM)**
ABT-751[31]	0,175*	0,35*	0,7*
Aphidicolin[32]	1*	2*	4*
AZD7762[33]	0,5	1	2
Hydroxy urea[32]	62,5	125	250
Lovastatin[32]	20	40	80
Mimosine[32]	100	200	400
Nocodazole[32]	0,1*	0,2*	0,4*
PHA-767491[34]	5	10	20
Thymidine[32]	1250	2500	5000
XL413[34]	5	10	20

***** Concentration reported as µg/mL instead of µM

Table S14: Reagents for scRNAseq

**Reagent**	**Vendor**

Maxima H Minus Reverse Transcriptase	Thermo Fisher
psfTn5	Addgene
KAPA HiFi HotStart ReadyMix	Roche
Lambda Exonuclease	BioNordika
Tween-20	Sigma Aldrich
10% SDS solution	Teknova
Magnesium Chloride (1 M)	Sigma Aldrich
Triton X-100	Sigma Aldrich
KAPA HiFi PCR kit with dNTPs	Roche
Betaine (5 M)	Sigma Aldrich
UltraPure DNase/RNase Free Distilled Water	Thermo Fisher
ERCC RNA Spike-In Mix	Thermo Fisher
USB Dithiothreitol (DTT, 0.1 M)	Thermo Fisher
RNase inhibitor	Takara Bio
dNTP Mix (dATP, dCTP, dGTP, and dTTP, each at 10 mM)	Thermo Fisher
SpeedBeads magnetic carboxylate modified particles	Merck
Peg8000	Sigma Aldrich
TAPS 0.2 M buffer soln., pH 8.5	Thermo Fisher
0.5 M EDTA, pH 8	Sigma Aldrich
Sodium Chloride solution 5 M	Invitrogen
Ultra Pure TrisHCl 1 M pH 8	Invitrogen
Qubit DNA HS	Thermo Fisher
Illumina compatible barcodes	IDT (see Table S16 for barcode sequences)

Table S15 (Excel file): Patient table for IEI patients used in the study

Table S16 (Excel file): CHOPOFF off-target predictions for selected gRNAs

Table S17 (Excel file): Barcodes used for scRNA-seq

Table S18 (Excel file): scRNA-seq fusion detection

Table S19 (Excel file): Mass spectrometry comparing all samples to unedited healthy controls

Table S20 (Excel file): Mass spectrometry comparing all samples to unedited DADA2 patients

Table S21 (Excel file): Mass spectrometry hits in DADA2 patients

SUPPLEMENTAL METHODS

Patient and healthy donor sample collection

We obtained peripheral blood, cord blood, and fibroblasts from human donors. The study was conducted per the principles of the Helsinki Declaration. It was approved by the Helsinki University Central Hospital Ethics Committee, and the Regional Committee for Medical and Health Research Ethics South-East Norway. The donors participating in the study have signed written informed consent. Information about patients used in the study is presented in Supplemental Table S15 (separate Excel file).

Isolation and culture of human primary T cells, CD34+ HSPCs and fibroblasts

To isolate human T cells, peripheral blood mononuclear cells (PBMCs) were isolated from human peripheral blood from healthy donors (HD) and patients using Ficoll (StemCell Technologies) gradient centrifugation. Isolated PBMCs were cryopreserved at -150°C. For experiments, PBMCs were thawed and cultured at 1 million cells/mL in ImmunoCult™-XF T Cell Expansion Medium (StemCell Technologies). The basal media was supplemented with 120 U/mL IL-2 (Peprotech), 3 ng/μL IL-7 (Peprotech), 3 ng/μL IL-15 (Peprotech) and 15 μL/mL ImmunoCult™ Human CD3/CD28 T Cell Activator (StemCell Technologies) to make T cell stimulation medium. After incubating cells at 37°C/5% CO_2 for three nights, cells were either nucleofected or diluted further with T cell stimulation medium without CD3/CD28 T Cell Activator.

CD34+ hematopoietic stem and progenitor cells (HSPCs) were isolated from cord blood collected during scheduled caesarean sections. Cells were isolated using CD34 MicroBead Kit UltraPure (Miltenyi), and cryopreserved at -150°C. For experiments, CD34+ HSPCs were thawed and cultured at 0.3 million cells/mL in StemSpan™ SFEM II (StemCell Technologies), supplemented with 1X GlutaMax (ThermoScientific), 100 ng/mL human recombinant Flt3-L (Peprotech), 20 ng/mL human recombinant TPO (Peprotech), 100 ng/mL human recombinant SCF (Peprotech), 20 ng/mL human recombinant IL-6 (Peprotech), 10 μM StemRegenin-1 (StemCell Technologies) and 50 μM UM729 (StemCell Technologies) to make HSPC stimulation medium. After incubating cells at 37°C/5% CO_2 for three nights, cells were either nucleofected or diluted further with HSPC stimulation medium.

Human fibroblasts were isolated from skin biopsies, expanded in DMEM medium supplemented with low glucose, 1 mM Puryvate and 10% FBS (Gibco) and cryopreserved at -150°C. For experiments, cells were thawed and cultured in same conditions until confluent.

Cells were passaged every 3-4 days by dissociating them with TrypLE™ Express Enzyme (Gibco), until cell expansion was complete and cells were nucleofected. All gene editing experiments with fibroblasts were carried out latest at passage 10.

CRISPR gRNA design for *ADA2, AIRE, RMRP* and *STAT1*

We designed 3-18 gRNAs per locus for *ADA2*, *AIRE*, *RMRP* and *STAT1* based on available PAM (NGG) sites within the 100 bp repair template region centering the mutation site. gRNA sequence information is presented in Table S2, where gRNAs overlapping the mutation sites are marked in red.

CRISPR repair template design for *ADA2, AIRE, RMRP* and *STAT1*

Single-stranded DNA repair templates (ssODN) of 100 bp length were designed for *ADA2*, *AIRE*, *RMRP* and *STAT1* with +/- 50 bp homology arms surrounding the mutation site. To prevent Cas9 from re-cutting the edited strand, silent SNPs were added in *ADA2*, *AIRE* and *STAT1* designs in addition to mutation correction (WT/MUT→SNP). Four silent SNPs were added for *ADA2* and three for *AIRE* and *STAT1*. As *RMRP* is non-coding, we used non-silent SNPs (WT→SNP, MUT→SNP) for early experiments and mutation correction (MUT→WT) later for functional assessments. ssODN sequence information is presented in Table S3. To further improve HDR, we designed asymmetric ssODNs for *ADA2*, *AIRE*, *RMRP* and *STAT1*, where we tested 10-40 nt homology arms surrounding the mutation sites. Sequences of asymmetric ssODNs and SNP strategy are presented in Table S3.

Nucleofection of human primary T cells, CD34+ HSPCs and fibroblasts

Human T cells, CD34+ HSPCs and fibroblasts were nucleofected using 4-D Nucleofector system and 96-well unit (Lonza). gRNAs were made by annealing crRNA (IDT) and tracrRNA (IDT) as according to the manufacturer's instructions. RNPs were prepared by mixing 61 pmol Alt-R™ S.p. Cas9 Nuclease V3 (IDT) with 100 pmol annealed gRNA per sample and incubating at 37°C for 15 min, after which 100 pmol ssODN (IDT) was added.

For nucleofection, 0.5 or 1 million T cells, 0.3 million HSPCs and 1 million fibroblasts per sample were resuspended in 20 uL Primary P3 electroporation buffer (Lonza) and mixed carefully with the RNPs, making the final concentrations of Cas9 nuclease at 3.05-, gRNA at 5- and ssODN at 5 μmol/L per nucleofected sample. Cells were nucleofected with the following programs: EO-115 (T cells), DZ-100 (HSPCs), CA-137 (fibroblasts).

After nucleofection of T cells, 85 µL T cell recovery medium (basal medium supplemented with 250U/mL IL-2) was added into the electroporation plate, followed by 15 min incubation at 37°C/5% CO_2. Afterwards, cells were transferred into 24-well (1 million cells/sample) or 48-well (0.5 million cells/sample) plates to grow and incubated at 37°C/5% CO_2. Cells were split 1:1 or as necessary with T cell recovery medium 24h and 72h after nucleofection. Cells were collected for downstream analyses 4 days, or alternatively 6-8 days, after nucleofection depending on the experiment.

After nucleofection of HSPCs, 85 µL HSPC stimulation medium (basal medium supplemented with aforementioned cytokines) was added into the electroporation plate, followed by 15 min incubation at 37°C/5% CO_2. Afterwards, cells were transferred into 48-well culture plates to grow and incubated at 37°C/5% CO_2. HSPC stimulation medium was added 24 and 72h after nucleofection if necessary. Cells were collected for downstream analyses 4 days after nucleofection.

After nucleofection of fibroblasts, 85 µL fibroblast culture medium (basal medium supplemented with aforementioned reagents) was added into the electroporation plate, followed by 15 min incubation at 37°C/5% CO_2. Afterwards, cells were transferred into 6-well culture plates to grow and incubated at 37°C/5% CO_2. Medium was changed 24h after nucleofection, and samples were trypsinized and collected for downstream analyses 4 days after nucleofection.

Flow cytometry

1. Characterization of immune cells in HD PBMCs

PBMC samples from day 1, 4 and 8 of the platform were prepared for flow cytometry analysis by washing 0.5 million cells per sample once with RT PBS, followed by blocking for 10 min at RT with 10% human serum in PBS. Cells were then stained in the dark for 30 min at 4°C by adding 50 µL antibody cocktail per sample, as presented in Table S4. After staining, cells were washed twice with 200 µL flow buffer (eBioscience) and resuspended in 250 µL flow buffer. Samples were stored in the dark at 4°C until flow cytometry. The flow analyses were performed on LSRII (BD Bioscience) at the Flow Cytometry Core Facility at Oslo University Hospital (Oslo, Norway). Data analysis was done with FlowJo software (FlowJo LLC, Ashland, OR).

2. CD4+ CD8+ T cell sorting panel for fluorescence-activated cell sorting

T cells from DADA2 patient and HD were collected on day 8 of the platform and prepared for flow cytometry analysis by collecting 2 million cells per sample and washing them once with ice-cold PBS. Cells were resuspended with 200 µL of 1:500 Live/Dead dye combined 1:10 FcR Blocking Reagent (Miltenyi) and samples were stained in the dark at 4°C for 30 min. Cells were then stained in the dark for 30 min at 4°C by adding 50 µL antibody cocktail per sample, as presented in Table S5. After staining, cells were washed once in cold flow buffer (eBioscience) and resuspended in cold flow buffer, followed by FACS (SONY SH800S) at Centre for Molecular Medicine Norway at University of Oslo, Norway. Data analysis was done with FlowJo software (FlowJo LLC, Ashland, OR).

3. T cell proliferation assay in CHH patients

CHH patient T cells from day 20 of the platform were collected and washed once with PBS. Cells were resuspended in PBS at 2 million cells/mL. CFSE working solution (2 µM) was prepared right before staining from CellTrace™ CFSE Cell Proliferation Kit (Invitrogen), where stock (5mM) was first diluted with PBS. To stain cells, equal volume of CFSE working solution and PBS were added to get a final concentration of 1 uM CFSE. Cells were immediately vortexed for 10 s, followed by incubation in the dark at 37°C, 5% CO2 for 5 min, including a brief vortexing step at 2.5 min of incubation. Immediately after incubation, equal volume of cold human serum (Sigma) was added on cells. Cells were centrifuged, washed twice with PBS and resuspended in Immunocult medium at 4 million cells/mL. Cell suspension (50 uL) was added per well on a 96-well U bottom plate (Thermo Fisher) containing 100 ul of Immunocult medium and IL2 to get 0.2 million cells per well with a final concentration of 250 U/mL IL-2 (Peprotech). The cells were then incubated for four days at 37°C, 5% CO_2. On day 24 of the platform, cells were stained for flow cytometry. Samples were washed once with PBS and resuspended in 50 uL Live/Dead staining with Fc blocking reagent (Miltenyi) per sample and incubated in the dark for 30 min at 4°C. Samples were centrifuged and resuspended in 50 uL antibody cocktail as presented in Table S6 and stained in the dark for 30 min at 4°C. Afterwards, cells were washed two times with flow buffer (eBioscience) and resuspended in flow buffer for flow cytometry analysis. The flow analyses were performed on LSRII (BD Bioscience) at the Flow Cytometry Core Facility at Oslo University Hospital (Oslo, Norway). Data analysis was done with FlowJo software (FlowJo LLC, Ashland, OR).

4. Assessment of phosphorylated STAT1 in STAT1-GOF patients

Patient T cells were harvested four days post electroporation. Cell suspension was washed twice in PBS by centrifuging at 400 g for 6 min and discarding the supernatant. One million cells per sample were used for each condition. Cells were resuspended with 100 µL of 1:500 Live/Dead dye combined 1:10 FcR Blocking Reagent (Miltenyi) and stained in the dark at 4°C for 30 min. After incubation, 5 mL ImmunoCult was added to the cells before centrifugation at 400 g for 6 min. The supernatant was discarded after centrifugation, and 250 µL of the 2 $x10^3$ U/ml of the IFNα in medium and the cocktail of cell surface antibodies (presented in Table S7) was added to the samples to be stimulated. The unstimulated controls received only 250 µL medium and the same antibody cocktail. The cells were incubated at 37 ºC for 30 min in the dark while being shaken every five minutes during incubation. Immediately after, 2 mL of freshly prepared 1:5 Phosflow Lyse/Fix Buffer was added to the sample tubes, which was then shortly vortexed before incubating in a 5 % CO2 incubator at 37 ºC for 10 min. The tubes were shaken every 3 minutes to ensure thorough cell lysis and fixation. After incubation, the tubes were centrifuged at 500 g for 8 min. The supernatant was discarded, and the cell pellet was shortly vortexed. 5 mL flow buffer was added to each tube and centrifuged at 500 g for 8 min. The supernatant was discarded, and the cell pellet was shortly vortexed. 500 µL of cold Phosflow PermBuffer III was added to each tube. The tubes were incubated on ice for 30 min in the dark. After incubation, 5 mL of eBioscience™ Flow Cytometry Staining Buffer was added to each tube, followed by centrifugation at 500 g for 8 min. Supernatant was discarded and cells were stained with 1:10 dilution of pSTAT1 antibody in a total staining volume of 50 µl. Cells were mixed and incubated for 30 min at RT in the dark. Afterwards, cells were washed two times with flow buffer, resuspended in flow buffer and stored in refrigerator overnight for flow cytometry analysis the day after.

Assessment of *in silico* gRNA design tools

To evaluate predictive power of available *in silico* gRNA design tools against *in vitro* gRNA screening data, we selected the following tools: Atum (https://www.atum.bio/eCommerce/cas9/input), Benchling (benchling.com), CHOPCHOP (chopchop.cbu.uib.no), CRISPOR (crispor.tefor.net/), DeepSpCas9 (deepcrispr.info/DeepSpCas9/), EuPaGDT (grna.ctegd.uga.edu/) and IDT gRNA design tool (eu.idtdna.com/site/order/designtool/index/CRISPR_SEQUENCE). We used 100 bp mutant-specific sequences with 50 bp homology arms from the mutation site as input (target sequence) for the tools. Three gRNAs with highest predicted efficiency were chosen from the tool output and compared against three best *in vitro* validated gRNAs from patient T cells.

On-target editing assessment by ddPCR

ddPCR assays were performed to assess HDR and NHEJ editing for *ADA2*, *AIRE*, *Enh4-1*, *CTCF1*, *RNF2*, *RMRP* and *STAT1*. We used previously published ddPCR oligos for Enh4-1, CTCF1, RNF2[35] and designed new ddPCR oligos for *ADA2*, *AIRE*, *RMRP* and *STAT1* (presented in Table S8). ddPCR was performed using the QX200 system (Bio-Rad) as previously described[35]. In short, 8 µl of DNA (concentration normalized to 8 ng/µl), primers (900 nM), reference probe (250 nM), and HDR or NHEJ probe (250 nM). The HDR and NHEJ detection occurred in two separate ddPCR reactions. Each reaction was then loaded into a sample well of an eight-well disposable cartridge (DG8; Bio-Rad Laboratories) along with 70 µl of droplet generation oil (Bio-Rad Laboratories). Droplets were formed using a QX200 Droplet Generator (Bio-Rad Laboratories). Droplets were transferred to a 96-well PCR plate, heat-sealed with foil, and amplified using a conventional thermal cycler. The thermocycling protocol was the following: (1) 95°C - 10 min, (2) 94°C – 30 s, 56°C – 3 min, step repeated 42 times (3) 98°C – 10 min, (4) 4°C – hold. The resulting PCR products were loaded on a QX200 Droplet Reader (Bio-Rad Laboratories), and the data was analyzed using QuantaSoft software (Bio-Rad Laboratories).

On-target editing assessment by amplicon sequencing

Amplicon sequencing libraries for assessing on-target editing for *ADA2*, *AIRE* and *RMRP* were prepared from gDNA samples as previously described.[35] In short, library preparation was performed using a two-step PCR method. For the first PCR, a pair of target-specific primers were designed to amplify a 150 bp area surrounding the cutting site. Each target primer additionally includes an extension at the 5' end: for forward primers, this contains the Illumina Read1 primer sequence (see below, nucleotides in bold) and an 8 bp UMI (nucleotides underlined), and for the reverse primers, this contains the Illumina Read2 primer sequence only (see below, nucleotides in bold):

ADA2 fwd 5'→3': **ACACTCTTTCCCTACACGACGCTCTTCCGATCT**NNNNNNNNTCATGCAGTTCAGATTTGCTCAC
ADA2 rev 5'→3': **GTGACTGGAGTTCAGACGTGTGCTCTTCCGATCT**GGCCTGGGACATGTGCTTTC

AIRE fwd 5'→3': **ACACTCTTTCCCTACACGACGCTCTTCCGATCT**NNNNNNNNactcccagcaagttcgaaga
AIRE rev 5'→3': **GTGACTGGAGTTCAGACGTGTGCTCTTCCGATCT**GGGGGCATCAAGAGCCAG

RMRP fwd 5'→3': **ACACTCTTTCCCTACACGACGCTCTTCCGATCT**NNNNNNNNgagtgggaagcggggaatg
RMRP rev 5'→3': **GTGACTGGAGTTCAGACGTGTGCTCTTCCGATCT**AGCTGAGGACGTGGTTCGT

First PCR was performed with reagents listed presented in Table S9. The thermocycling protocol was the following: (1) 98°C - 30 s, (2) 98°C – 10 s, 57°C (ADA2, RMRP)/58°C (AIRE) – 10 s, 72°C – 20 s, step repeated 30 times (3) 72°C – 5 min, (4) 4°C – hold.

For the second PCR, the amplified products were purified using AMPure XP magnetic (Beckman Coulter, #A63882) according to the manufacturer's instructions, pooled and annealed with i5 and i7 Illumina Index primers (presented in Table S10). Both primers contain flow-cell-binding region (highlighted in bold), index region (underlined) and Illumina Read1 or Read2 primer binding regions, correspondingly (italics).

Second PCR was performed with reagents listed in Table S10. The thermocycling protocol was the following: (1) 98°C - 30 s, (2) 98°C – 10 s, 58°C – 10 s, 72°C – 20 s, step repeated 10 times (3) 72°C – 5 min, (4) 4°C – hold.

PCR products were purified using AMPure XP magnetic beads (Beckman Coulter) according to the manufacturer's instructions and DNA concentrations were measured with Qubit HS kit (Thermo Fisher Scientific). Final sample libraries were sequenced using Illumine MiSeq v2 Micro flow cell, including 10% PhiX. Data analysis was performed using the ampliCan software package.[36]

Off-target assessment by GUIDE-seq

One million patient and healthy donor T cells/sample were nucleofected on day 5 of the platform, as previously described, with RNPs containing selected gRNAs 100 pmol, Cas9 nuclease at 61 pmol and dsODN at 30 pmol/nucleofected sample. Thus, final concentrations of CRISPR reagents were: Cas9 nuclease at 3.05-, gRNA at 5- and dsODN at 1.5 μmol/L per nucleofected sample.

Cells were transferred into 24w plates after nucleofection with 500 uL T cell recovery medium and split 1:1 with T cell recovery medium 24h and 72h after nucleofection. Samples were collected for GUIDE-seq sample processing and ddPCR 4 days after nucleofection.

The blunt-ended dsODN used in our GUIDE-seq experiments was the same as that was used in the original publication.[37] dsODN was prepared by annealing the two modified oligonucleotides of the following compositions:

5′- P-G*T**TTAATTGAGTTGTCATATGTTAATAACGGT**A*T -3′ and
5′- P-A*T**ACCGTTATTAACATATGACAACTCAATTAA**A*C -3′
P represents a 5′ phosphorylation and * indicates a phosphorothioate linkage.

The GUIDEseq protocol[37] was adapted from certain modifications described below. Briefly, gDNA was sheared with a Bioruptor® Pico Sonication System (Diagenode) to an average length of 500 bp. End-repair was done with Fast DNA End Repair Kit (Thermo Fisher Scientific), A-tailing with Taq DNA Polymerase, native (Thermo Fisher Scientific) and ligation of half-functional adapters, incorporating 8-nt random molecular index was done using T4 DNA Ligase (Thermo Fisher Scientific), all according to the manufacturer's instructions. Between each step, DNA was purified using AMPure XP SPRI beads (Backman Coulter) and eluted with TE buffer, pH 8.0 (Invitrogen). Two rounds of nested anchored PCR, with primers complementary to the oligo tag, were used for target enrichment. Before library pooling, the quality of the final products was tested using High Sensitivity DNA Kit (Agilent) on Bioanalyzer 2100 (Agilent) according to the manufacturer instructions and NanoDrop (Thermo Fisher Scientific). Based on the average size estimated from Bioanalyzer, equal number of particles from each sample were pulled to achieve the final volume 20µl containing $1{,}2 \times 10^{10}$.

Sample was delivered together with custom sequencing primer Index-1 and Read-2 and sequenced at The Department of Core Facilities, Oslo University Hospital, Norway. Denaturated library was loaded onto the Miseq according to Illumina's standard protocol for sequencing with an Illumina Miseq Reagent Kit V2 - 300 cycle (2 x 150 bp paired end).

Data analysis was performed following the GUIDE-Seq analysis pipeline[38] but adjusted for allowing bulges between sgRNA and off-target sites with editing distance of 4. We used custom scripts (https://git.app.uib.no/valenlab/t_cell_editing_pipeline/) with cutadapt v2.8 (TTGAGTTGTCATATGTTAATAACGGTAT and ACATATGACAACTCAATTAAAC). Afterward, the data was aligned to the human genome (hg38v34) using bwa v0.7.17-r1188. CHOPOFF (https://github.com/JokingHero/CHOPOFF.jl) was used to find all off-target sites with edit distance up to 4, allowing for mismatches, deletions and insertions. Final off-targets were normalized against control data (transfected with dsODN only). Control data was processed in the same pipeline as the modified Cas9 samples. Detailed analysis of predicted *in silico* off-targets can be found in Supplemental Table S16 (separate Excel file).

Cas9WT and Cas9-SNAP *in vitro* mRNA transcription

Cas9WT and Cas9-SNAP mRNA were prepared with HiScribe T7 ARCA mRNA Kit with tailing (NEB-Bionordika), according to the manufacturer's instructions. Total of 8000 ng stock plasmid was digested with 2 µl FastDigest MssI enzyme (Thermo Fisher Scientific) in the supplemented restriction-digestion buffer with a total reaction volume of 20 µl. Incubation was carried out at 37°C overnight. Length of the digested product was confirmed by gel electrophoresis. For the IVT reaction, 1000 ng of the linearized plasmid was mixed with 10 µl of 2xARCA/NTP mix and 2 µl of T7 RNA Polymerase mix, and the reaction was incubated for 30 min at 37°C. Sequentially, 2 µl of DNAse enzyme was added and the mixture was incubated at 37°C for 15 min. Poly(A) tailing step was performed by adding 20 µl of milliQ (RNAse free), 5 µl of 10× PolyA polymerase reaction buffer, and 5 µl of 10× PolyA polymerase directly to the IVT reaction, which was incubation at 37°C for 30 min. mRNA was purified using LiCl solution, as described in the manufacturer's protocol. Aliquots were frozen in -80°C for later use.

Synthesis of O^6-Benzylguanine coupled repair templates

BG-coupled repair template oligos for ADA2 and AIRE were prepared as previously described.[39,40] In short, a coupling reaction of BG-GLA-NHS (New England BioLabs) and NH2-oligo (IDT) in HEPES buffer pH 8.5 (Invitrogen) was performed. Following coupling reactions, the BG-oligos were purified with ethanol precipitation as and stored at -20°C until later use.

Cas9-SNAP nuclease production

To test the editing performance of BG-coupled repair oligo in combination with the Cas9-SNAP fusion protein, the Cas9-SNAP protein was produced as protein. The pTH24-Cas9-SNAP construct was transformed into *E. coli* BL21(DE3) T1R cells and cultivated in Terrific Broth (TB) medium. Protein expression was induced with isopropyl-D-1-thiogalactopyranoside, and protein purified by immobilized metal-ion chromatography, followed by size exclusion chromatography (SEC). The purified protein was stored in 20 mM HEPES supplemented with 300 mM NaCl, 10% glycerol and 2 mM TCEP to pH 7.5. Aliquots were flash-frozen in liquid nitrogen and stored at -80°C until experiments.

HDR enhancing compound screen in healthy donor T cells

We selected 33 previously published HDR enhancing compounds for the screen, described in Table S12. Compounds were dissolved in DMSO and each of them were assessed at three concentrations in HD T cells against DMSO vehicle control. As described previously, 0.5

million T cells/sample were nucleofected, transferred to 48-well cell culture plates and incubated in T cell recovery medium containing the compounds for 24h. Cells were split 1:1 24h and 72h after nucleofection in T cell recovery medium without the compounds. Samples were collected for gDNA extraction and ddPCR 96h after nucleofection.

Validating cell cycle inhibitors in healthy donor T cells

We selected 10 previously published HDR enhancing cell cycle inhibitors for validating in HD T cells and assessed at three concentrations against DMSO vehicle control, described in Table S13. Cells were either pre-treated with the compounds or vehicle for 24h before nucleofection, followed by nucleofection and incubation without compounds, or treated for 24h after nucleofection. For both conditions, 0.5 million cells per sample were nucleofected. For both groups, cells were split 1:1 in T cell recovery medium without compounds 24h and 72h after nucleofection. Samples were collected for gDNA extraction and ddPCR 96h after nucleofection.

PacBio sequencing and variant calling of CRISPR edited healthy donor T cells

T cells from a healthy donor with written consent for sequencing were cultured and edited as previously described. The cells were either unedited and treated with 0.5 µM KU0060648 or DMSO or *ADA2*-edited and treated with 0.5 µM KU0060648 or DMSO. Cells were collected six days after editing on day 10 of the platform and DNA was extracted from 5 million cells per sample using Blood & Cell Culture DNA Kits (Qiagen). All samples were extracted according to the manufacturer's instructions for Cell cultures described in “QIAGEN® Genomic DNA Handbook, June 2015”. The concentration, purity and size of the DNA was estimated using both NanoDrop (Thermo Fisher Scientific) and a Qubit fluorometer (Invitrogen) and checked on the agarose gel (0.5%, 35V, 16-18 hours runtime) containing 500 ng of each sample, with an appropriate ladder as a reference standard: Quick-Load 1 kb Extend DNA Ladder (New England Biolabs).

Library preparations for PacBio HiFi sequencing were done by the Norwegian sequencing Centre on 8M SMRT cells using Revio HiFi prep kit and Sequencing chemistry v2.0. The sequencing data was demultiplexed with the Demultiplexing pipeline on SMRT Link v10.2.0.1333434. Circular consensus sequencing (CCS) reads were then generated for demultiplexed polymerase reads and further demultiplexed using the barcoded primer sequences. The HiFi sequencing reads were separated and indexed with the provided barcode ID.

The HiFi sequencing reads were aligned with pbmm2 v1.13.0 with options "--preset HIFI --bam-index BAI --sort". Structural variants were called with pbsv v2.9.0, small variants with deepVariant v1.6.0. All possible mismatches, deletions and insertions were extracted from aligned reads using custom scripts (https://git.app.uib.no/valenlab/t_cell_editing_pipeline/-/tree/main/katariina_pacbio). We normalized data using two control samples and focused on sites that were potential sgRNA off-target within distance of 4, allowing for bulges. Additionally, transversion ratio plot and codon signature analysis showed no global effects of CRISPR activity.

CellTiter-Glo cell viability assay for HDR enhancing compound toxicity assessment

HDR enhancing compound toxicity was assessed by CellTiter-Glo viability assay (Promega) according to manufacturer's instructions. In short, 50 µL of T cell suspension per sample was transferred into while opaque 96-w plates (Thermo Fisher), followed by adding 100 µL RT CellTiter-Glo assay buffer per sample. Plate was covered with aluminum foil and placed on a plate shaker at 500 rpm for 5 min. Afterwards, plate was incubated for 10 min at RT while still covered. After incubation, foil was removed and luminescence values from the plate were assessed by BioTek Synergy Neo2 Instrument (Agilent). To analyze results, background values from medium alone were subtracted from sample values and data was analyzed according to manufacturer's instructions.

scRNAseq in HD and DADA2 patient T cells

1. Cell culture and processing

Cells were cultured and nucleofected as previously described and sorted with FACS on day 8 of the platform into 384 well plates containing 2 uL of lysis buffer [H_2O: 1.31 uL, RNase Inhibitor 0.05 uL, ERCC (1:30000) 0.05 uL, 10% Triton (0.04 uL), 10 mM dNTP (0.5 uL) and 100 uM oligo dT (0.05 uL)]. After sorting, the plates were spun down at 2000g, 4°C for 5 min and then the plate was snap frozen on dry ice and kept at -80 until further processing. Reagents for sample processing are described in Table S14.

Oligos:

Oligo-dT:	IDT	AAGCAGTGGTATCAACGCAGAGTACTTT<br>TTTTTTTTTTTTTTTTTTTTTTTTTTT<br>(N1:34333300)(N2:25252525)
IS_PCR	IDT	5′-AAGCAGTGGTATCAACGCAGAGT-3′

TSO	IDT	5′-AAGCAGTGGTATCAACGCA GAGTACATrGrG+G-3′
ME-A	IDT	5′-TCGTCGGCAGCGTCAGATGTG TATAAGAGACAG-3′
ME-B	IDT	5′-GTCTCGTGGGCTCGGAGATG TGTATAAGAGACAG-3′
ME-Rev	IDT	5′-/5Phos/CTGTCTCTTATACACATCT-3′

ADA2_WT IDT /56-FAM/TGGAGGATT/ZEN/ATCGGAAGCGGGTG/3IABkFQ/

ADA2_Mut/WT_Fixed IDT /5HEX/TGGAGGACT/ZEN/ACAGAAAGCGGGTG/3IABkFQ/

ADA2 fwd: GGTGAGGAATGTCACCTACA

ADA2 rev: CATCAAACTCAGTGACGTTC

2. RNA preparation

Full length mRNA-sequencing is based on Smart-Seq2 protocol.[41,42] Lysis plates containing cells were thawed and primer annealing was performed for 3 min at 72°C. 3 uL of reverse transcription mix (5x Reverse Transcriptase buffer (1 uL), Maxima H minus Reverse Transcriptase (0.05 uL), RNase Inhibitor (0.125uL), 100mM DTT (0.25 uL), 5M Betaine (1 uL), 1M $MgCl_2$ (0.03 uL), 100uM TSO (0.05 uL), H_2O 0.495) was added to each well, and reaction occurred at 42°C for 90 min, then heat inactivation at 70°C for 5 min. Next cDNA pre-amplification was performed by adding 7 uL of mastermix [2X Kapa HiFi HotStart ReadyMix (6 uL), 10 uM IS_PCR primer (0.12 uL), Lambda exonuclease (0.05625 uL) and H_2O (0.8237 uL)]. PCR program was 37°C for 30 min, 95°C for 3 min, 22 cycles of 98°C for 20s, 67°C for 15s, 72°C for 4 min, then final elongation at 72°C for 5 min.

At this stage primers are removed by SPRI bead cleanup (prepared as here https://openwetware.org/wiki/SPRI_bead_mix#Ingredients_for_50_mL_2) at a ratio of 0.7:1. Concentration of independent wells is measured with Qubit DNA HS kit, and wells are diluted to 0.15 ng/uL.

3. Library preparation

Tagmentation was performed on the diluted cDNA, by adding 1 uL cDNA to 1.5 uL tagmentation mix (Tn5 (2.6mg/mL purified psfTn5-c006, Addgene plasmid #79107, loaded with standard Illumina Tn5 adapters (Meds A, MedsB, MedsRev))) (0.250 uL), 5X TAPS-PEG (Buffer is is 8% PEG, 5mM MgCl2, 10mM TAPS) 0.5 uL), H2O (0.750 uL)) and incubate for

10 min at 55°C. Then the reaction was stopped and transposome stripped of cDNA by adding 0.1% SDS (1 uL) and incubating for 10 min at 55°C. 7 uL barcoding mix was added (5x buffer (2.5 uL), 10mM dNTP (0.3 uL), 10% Tween (0.15 uL), Kapa HiFi (0.2 uL), H2O (3.85 uL) and 2 uL primer mix at 3.75 uM/primer. PCR program was as follows: 72°C for 3 min, 95°C for 30s, then 12 cycles of 95°C for 15s, 55°C for 30s, 72°C 45s, then final elongation at 72°C for 5 min.

Library was pooled and cleaned up 2x with 0.9:1 ratio of SPRI beads.

Libraries were sequenced on a Novaseq 6000.

4. RT-qPCR

For quantitative analysis of the two different alleles, 1uL of the diluted cDNA is amplified with Ada2 specific primers in the presence of WT or mutated and edited probes that are attached to different fluorophores. Reaction conditions were as follows: 2X Kapa HiFi HotStart ReadyMix (2.5 uL), 10uM forward and reverse primers (0.05 + 0.05 uL), 10 uM WT probe (0.05 uL), 10 uM Edited probe (0.05 uL), H2O (1.3 uL). PCR program was 95°C for 1 min, 35 cycles of 95°C for 15 s, 63°C for 45 s.

5. Analysis of scRNA-seq data

Cutadapt[43] was used to trim RNA sequence reads from adapters and low-quality bases. STAR[44] was used to align to hg38, with ERCC reads added. Picard[45] was used to remove duplicate reads. HTSeq[46] was used to summarize read counts. Cells with less than 20000 reads or 500 features were filtered out, as well as those with ACTB expression less than 0.01 quantile of the normal distribution. Seurat[47] was used to process the count data. Shortly, data was log normalized, 2000 variable features were found and the data were scaled; this was done separately per condition. Pathway analysis on scRNA-data was done as follows. FindMarkers function of Seurat was used to find markers between the two conditions of interest, with logfc.threshold=0. The resulting table was arranged based on avg_log2FC. The fgsea[48] package was used to read in the C7 and hallmark pathways from the Molecular Signature Database /Subramanian, Tamayo, et al. (2005, PNAS) and one or more of the following as appropriate: Liberzon, et al. (2011, Bioinformatics) and to perform gene set enrichment analysis based on the ordered avg_log2FC from FindMarkers and minSize=15 and maxSize=500. Information about barcodes used for sequencing can be found in Supplemental Table S17 (separate Excel file).

STAR-Fusion[49] was used to predict fusion transcripts from the single cell data. Pseudobulk samples were prepared by merging fastq files per condition (unedited, edited +/- NHEJ inhibition, per individual), and run with default parameters. The deconvolved abridged fusion predictions were filtered by removing all fusions that were categorised as neighbouring by STAR-Fusion annotation. Information about fusion transcripts can be found in Supplemental Table S18 (separate Excel file).

To detect possible chromosomal loss due to editing all bam files from two individuals, a patient and a healthy donor were merged to create pseudobulk bam files using samtools.[50] cellsnp-lite[51] was used to genotype the samples, based on the 1000G phase 1 SNPs (https://www.internationalgenome.org/category/variants/), with settings minMAPQ=20, minLEN=30, UMItag=None, p=20, -I P, countORPHAN, exclFLAG=UNMAP, SECONDARY, QCFAIL, DUP. The output was filtered for GT="het" & INFO/AD[0]>1 using bcftools.[52] The vcf files were uploaded to Michigan Imputation Server,[53] where the 1000G phase3 30x panel was chosen as a reference for imputation and Eagle2.4 was chosen for phasing. The imputed files were filtered with bcftools for R2>0.3 and TYPE="snp. Then cellsnp-lite was run on all individual cells against the imputed file for each sample, with the same parameters as before, except minCOUNT=10 and minMAF=0.2. The haplotype ratios were calculated and cells with only one haplotype on the q arm of chr22 were considered to have lost one chromosome.

6. Analysis of RT-qPCR data

RT-qPCR analysis was performed on the RFU values per allele. A cutoff of an RFU value 200 was determined to decide which allele (wild type, mutated or edited) was being expressed in each cell.

7. TCR Repertoire analysis

TCR reconstruction was performed using TRACER (v0.6.0)[54] with settings --loci A B G D -p 16 -s Hsap on individual fastq files. Only cells harboring productive A and B locus were used for clonotype identification.

Mass spectrometry

1. Sample preparation

T cells from three DADA2 patients and healthy donors were cultured as previously described and nucleofected on day 5 of the platform, where 1M cells/sample were mock nucleofected or

ADA2-edited. Mock edited cells were treated with DMSO and edited cells with 0.5µM KU0060648, 0.6µM IDT Alt-R enhancer V2 or DMSO for the first 24h after nucleofection. Cells were collected seven days after editing on day 12 of the platform, washed 2X with ice-cold PBS, pelleted and snap-frozen in liquid nitrogen. Pellets were stored at -80°C until mass spectrometry sample preparation.

The samples were lysed in 8M Urea (#U5378-500G, Sigma Aldrich) in 100 mM ammonium bicarbonate (NH4HCO3 containing benzonase nuclease (415 units/ml, sc-202391, Santa Cruz Biotechnology). Total protein concentration was measured with Bio-Rad Protein Assay Dye (#5000006, Bio-Rad Laboratories). 50 µg of total protein was taken from each sample for reduction (5 mM dithiothreitol (#D9779, Sigma-Aldrich), alkylation (15 mM iodoacetamide (#122271000, Acros Organics), and overnight digestion with 2µg Trypsin/Lys-c Mix (V507A, Promega) at 37°C. After digestion, samples were acidified with 10% trifluoroacetic acid (TFA, #85049.051, VWR) and desalted with BioPureSPN PROTO 300 C18 Mini columns (#HUM S18V, Nest Group) according to manufacturer's instructions. After desalting the samples were dried in a centrifuge concentrator (Concentrator Plus, Eppendorf). The dried peptides were reconstituted in 40 µl buffer A (0.1% (vol/vol) TFA, 1% (vol/vol) acetonitrile (#83640.320, VWR) in HPLC grade water (#10505904, Fisher Scientific)).

For the DIA analysis the resuspended peptides were further diluted 1:60 in buffer A1 (1% formic acid in HPLC water). 20 µl was loaded into an Evotip (Evosep, Denmark) following manufacturer's instructions.

2. Mass spectrometry and analysis

The desalted samples were analyzed using the Evosep One liquid chromatography system coupled to a hybrid trapped ion mobility quadrupole TOF mass spectrometer (Bruker timsTOF Pro, Bruker Daltonics) (Meier, Brunner et al., 2018) via a CaptiveSpray nano-electrospray ion source (Bruker Daltonics). An 8 cm × 150 µm column with 1.5 µm C18 beads (EV1109, Evosep) was used for peptide separation with the 60 samples per day methods (21 min gradient time). Mobile phases A and B were 0.1% formic acid in water and 0.1% formic acid in acetonitrile, respectively. The MS analysis was performed in the positive-ion mode with dia-PASEF method[55] with sample optimized data independent analysis (dia) scan parameters. We performed DDA in PASEF mode from a pooled sample to be able to adjust dia-PASEF parameters optimally to these specific samples. To perform sample specific dia-PASEF parameter adjustment the default dia-short-gradient acquisition methods was adjusted based on

the sample specific DDA-PASEF run with the software "tims Control" (Bruker Daltonics). The following parameters were modified for each sample type: m/z range; 429.2 – 1204.2, mass steps per cycle; 31 mean cycle time; 1.48 s. The ion mobility windows were set to best match the ion cloud density from the sample type specific DDA-runs.

To analyze diaPASEF data, the raw data (.d) were processed with DIA-NN v1.8.1[56,57] utilizing spectral library generated from the UniProt human proteome. During library generation following settings were used, fixed modifications: carbamidomethyl (C); variable modifications: acetyl (protein N-term), oxidation (M); enzyme:Trypsin/P; maximum missed cleavages:1; mass accuracy fixed to 1.5e-05 (MS2) and 1.5e-05 (MS1); Fragment m/z set to 100-1700; peptide length set to 7-30; precursor m/z set to 300-1600; Precursor changes set to 2-4; protein inference not performed. All other settings were left to default. Information about mass spectrometry data in healthy controls and patients can be found in Supplemental Tables S19-21 (separate Excel files).

3. Statistical analysis of the proteomics data

The input file to further DIA data analysis was the DIA-NN Report.pg_matrix. For data pre-processing an in-house R-script was utilized. Raw intensity values were log2 transformed and median-normalized. Afterwards, missing values were imputed using QRILC imputation.[58] For sample group comparison, p-values were calculated with student's t-test using python package scipy,[59] and adjusted using benhamini-hockberg method via statsmodels package.[60] Volcano plots were generated with bioinfokit[61] using q-value threshold of 0.01 and log2 intensity fold change thresholds of 1 and -1.

Statistics:

The following softwares were used for data analysis: QuantaSoft (Bio-Rad), FlowJo, Cutadapt 3.2, STAR 2.7.7a, HTseq 0.9.0, Picard 2.22.0, Seurat 5.0.1, FGSEA 1.20.1, STAR-Fusion V1.11.0., cellsnp-lite 1.2.3, bcftools 1.14. All of the statistics in the study were performed using GraphPad Prism 9.

References

1. Fox, T.A., Houghton, B.C., Petersone, L., Waters, E., Edner, N.M., McKenna, A., Preham, O., Hinze, C., Williams, C., de Albuquerque, A.S., et al. (2022). Therapeutic gene editing of T cells to correct CTLA-4 insufficiency. Sci Transl Med *14*, eabn5811. 10.1126/scitranslmed.abn5811.
2. Li, X., Wirtz, T., Weber, T., Lebedin, M., Lowenstein, E.D., Sommermann, T., Zach, A., Yasuda, T., de la Rosa, K., Chu, V.T., et al. (2024). Precise CRISPR-Cas9 gene repair in autologous memory T

cells to treat familial hemophagocytic lymphohistiocytosis. Sci Immunol *9*, eadi0042. 10.1126/sciimmunol.adi0042.
3. Roth, T.L., Puig-Saus, C., Yu, R., Shifrut, E., Carnevale, J., Li, P.J., Hiatt, J., Saco, J., Krystofinski, P., Li, H., et al. (2018). Reprogramming human T cell function and specificity with non-viral genome targeting. Nature *559*, 405-409. 10.1038/s41586-018-0326-5.
4. Goodwin, M., Lee, E., Lakshmanan, U., Shipp, S., Froessl, L., Barzaghi, F., Passerini, L., Narula, M., Sheikali, A., Lee, C.M., et al. (2020). CRISPR-based gene editing enables FOXP3 gene repair in IPEX patient cells. Sci Adv *6*, eaaz0571. 10.1126/sciadv.aaz0571.
5. Vavassori, V., Mercuri, E., Marcovecchio, G.E., Castiello, M.C., Schiroli, G., Albano, L., Margulies, C., Buquicchio, F., Fontana, E., Beretta, S., et al. (2021). Modeling, optimization, and comparable efficacy of T cell and hematopoietic stem cell gene editing for treating hyper-IgM syndrome. EMBO Mol Med *13*, e13545. 10.15252/emmm.202013545.
6. Asperti, C., Canarutto, D., Porcellini, S., Sanvito, F., Cecere, F., Vavassori, V., Ferrari, S., Rovelli, E., Albano, L., Jacob, A., et al. (2023). Scalable GMP-compliant gene correction of CD4+ T cells with IDLV template functionally validated in vitro and in vivo. Mol Ther Methods Clin Dev *30*, 546-557. 10.1016/j.omtm.2023.08.020.
7. Houghton, B.C., Panchal, N., Haas, S.A., Chmielewski, K.O., Hildenbeutel, M., Whittaker, T., Mussolino, C., Cathomen, T., Thrasher, A.J., and Booth, C. (2022). Genome Editing With TALEN, CRISPR-Cas9 and CRISPR-Cas12a in Combination With AAV6 Homology Donor Restores T Cell Function for XLP. Front Genome Ed *4*, 828489. 10.3389/fgeed.2022.828489.
8. Ayoub, P.G., Gensheimer, J., Lathrop, L., Juett, C., Quintos, J., Tam, K., Reid, J., Ma, F., Tam, C., McAuley, G.E., et al. (2024). Lentiviral vectors for precise expression to treat X-linked lymphoproliferative disease. Mol Ther Methods Clin Dev *32*, 101323. 10.1016/j.omtm.2024.101323.
9. Li, X.L., Li, G.H., Fu, J., Fu, Y.W., Zhang, L., Chen, W., Arakaki, C., Zhang, J.P., Wen, W., Zhao, M., et al. (2018). Highly efficient genome editing via CRISPR-Cas9 in human pluripotent stem cells is achieved by transient BCL-XL overexpression. Nucleic Acids Res *46*, 10195-10215. 10.1093/nar/gky804.
10. Riesenberg, S., and Maricic, T. (2018). Targeting repair pathways with small molecules increases precise genome editing in pluripotent stem cells. Nat Commun *9*, 2164. 10.1038/s41467-018-04609-7.
11. Yu, C., Liu, Y., Ma, T., Liu, K., Xu, S., Zhang, Y., Liu, H., La Russa, M., Xie, M., Ding, S., and Qi, L.S. (2015). Small molecules enhance CRISPR genome editing in pluripotent stem cells. Cell Stem Cell *16*, 142-147. 10.1016/j.stem.2015.01.003.
12. Liu, B., Chen, S., Rose, A., Chen, D., Cao, F., Zwinderman, M., Kiemel, D., Aïssi, M., Dekker, F.J., and Haisma, H.J. (2020). Inhibition of histone deacetylase 1 (HDAC1) and HDAC2 enhances CRISPR/Cas9 genome editing. Nucleic Acids Res *48*, 517-532. 10.1093/nar/gkz1136.
13. Stein, E.M., Garcia-Manero, G., Rizzieri, D.A., Tibes, R., Berdeja, J.G., Jongen-Lavrencic, M., Altman, J.K., Dohner, H., Thomson, B., Blakemore, S.J., et al. (2015). A Phase 1 Study of the DOT1L Inhibitor, Pinometostat (EPZ-5676), in Adults with Relapsed or Refractory Leukemia: Safety, Clinical Activity, Exposure and Target Inhibition. Blood *126*, 2547-2547. 10.1182/blood.V126.23.2547.2547.
14. Neal, J.A., Dang, V., Douglas, P., Wold, M.S., Lees-Miller, S.P., and Meek, K. (2011). Inhibition of homologous recombination by DNA-dependent protein kinase requires kinase activity, is titratable, and is modulated by autophosphorylation. Mol Cell Biol *31*, 1719-1733. 10.1128/mcb.01298-10.
15. Munck, J.M., Batey, M.A., Zhao, Y., Jenkins, H., Richardson, C.J., Cano, C., Tavecchio, M., Barbeau, J., Bardos, J., Cornell, L., et al. (2012). Chemosensitization of cancer cells by KU-0060648, a dual inhibitor of DNA-PK and PI-3K. Mol Cancer Ther *11*, 1789-1798. 10.1158/1535-7163.Mct-11-0535.
16. Robert, F., Barbeau, M., Éthier, S., Dostie, J., and Pelletier, J. (2015). Pharmacological inhibition of DNA-PK stimulates Cas9-mediated genome editing. Genome Med *7*, 93. 10.1186/s13073-015-0215-6.
17. Maurissen, T.L., and Woltjen, K. (2020). Synergistic gene editing in human iPS cells via cell cycle and DNA repair modulation. Nat Commun *11*, 2876. 10.1038/s41467-020-16643-5.
18. Hickson, I., Zhao, Y., Richardson, C.J., Green, S.J., Martin, N.M., Orr, A.I., Reaper, P.M., Jackson, S.P., Curtin, N.J., and Smith, G.C. (2004). Identification and characterization of a novel and specific inhibitor of the ataxia-telangiectasia mutated kinase ATM. Cancer Res *64*, 9152-9159. 10.1158/0008-5472.Can-04-2727.
19. Riesenberg, S., Chintalapati, M., Macak, D., Kanis, P., Maricic, T., and Pääbo, S. (2019). Simultaneous precise editing of multiple genes in human cells. Nucleic Acids Res *47*, e116. 10.1093/nar/gkz669.
20. Zhao, T., Li, Q., Zhou, C., Lv, X., Liu, H., Tu, T., Tang, N., Cheng, Y., Liu, X., Liu, C., et al. (2021). Small-molecule compounds boost genome-editing efficiency of cytosine base editor. Nucleic Acids Res *49*, 8974-8986. 10.1093/nar/gkab645.

21. Li, G., Zhang, X., Zhong, C., Mo, J., Quan, R., Yang, J., Liu, D., Li, Z., Yang, H., and Wu, Z. (2017). Small molecules enhance CRISPR/Cas9-mediated homology-directed genome editing in primary cells. Sci Rep *7*, 8943. 10.1038/s41598-017-09306-x.
22. Karthik, S., Sankar, R., Varunkumar, K., and Ravikumar, V. (2014). Romidepsin induces cell cycle arrest, apoptosis, histone hyperacetylation and reduces matrix metalloproteinases 2 and 9 expression in bortezomib sensitized non-small cell lung cancer cells. Biomed Pharmacother *68*, 327-334. 10.1016/j.biopha.2014.01.002.
23. Park, H., Shin, J., Choi, H., Cho, B., and Kim, J. (2020). Valproic Acid Significantly Improves CRISPR/Cas9-Mediated Gene Editing. Cells *9*. 10.3390/cells9061447.
24. Pinder, J., Salsman, J., and Dellaire, G. (2015). Nuclear domain 'knock-in' screen for the evaluation and identification of small molecule enhancers of CRISPR-based genome editing. Nucleic Acids Res *43*, 9379-9392. 10.1093/nar/gkv993.
25. Dutta, A., Eckelmann, B., Adhikari, S., Ahmed, K.M., Sengupta, S., Pandey, A., Hegde, P.M., Tsai, M.S., Tainer, J.A., Weinfeld, M., et al. (2017). Microhomology-mediated end joining is activated in irradiated human cells due to phosphorylation-dependent formation of the XRCC1 repair complex. Nucleic Acids Res *45*, 2585-2599. 10.1093/nar/gkw1262.
26. Iyer, S., Suresh, S., Guo, D., Daman, K., Chen, J.C.J., Liu, P., Zieger, M., Luk, K., Roscoe, B.P., Mueller, C., et al. (2019). Precise therapeutic gene correction by a simple nuclease-induced double-stranded break. Nature *568*, 561-565. 10.1038/s41586-019-1076-8.
27. Singh, P., Schimenti, J.C., and Bolcun-Filas, E. (2015). A mouse geneticist's practical guide to CRISPR applications. Genetics *199*, 1-15. 10.1534/genetics.114.169771.
28. Lee, J.S. (2007). Activation of ATM-dependent DNA damage signal pathway by a histone deacetylase inhibitor, trichostatin A. Cancer Res Treat *39*, 125-130. 10.4143/crt.2007.39.3.125.
29. Takayama, K., Igai, K., Hagihara, Y., Hashimoto, R., Hanawa, M., Sakuma, T., Tachibana, M., Sakurai, F., Yamamoto, T., and Mizuguchi, H. (2017). Highly efficient biallelic genome editing of human ES/iPS cells using a CRISPR/Cas9 or TALEN system. Nucleic Acids Res *45*, 5198-5207. 10.1093/nar/gkx130.
30. Delacôte, F., Han, M., Stamato, T.D., Jasin, M., and Lopez, B.S. (2002). An xrcc4 defect or Wortmannin stimulates homologous recombination specifically induced by double-strand breaks in mammalian cells. Nucleic Acids Res *30*, 3454-3463. 10.1093/nar/gkf452.
31. Yang, D., Scavuzzo, M.A., Chmielowiec, J., Sharp, R., Bajic, A., and Borowiak, M. (2016). Enrichment of G2/M cell cycle phase in human pluripotent stem cells enhances HDR-mediated gene repair with customizable endonucleases. Sci Rep *6*, 21264. 10.1038/srep21264.
32. Lin, S., Staahl, B.T., Alla, R.K., and Doudna, J.A. (2014). Enhanced homology-directed human genome engineering by controlled timing of CRISPR/Cas9 delivery. Elife *3*, e04766. 10.7554/eLife.04766.
33. Ma, X., Chen, X., Jin, Y., Ge, W., Wang, W., Kong, L., Ji, J., Guo, X., Huang, J., Feng, X.H., et al. (2018). Small molecules promote CRISPR-Cpf1-mediated genome editing in human pluripotent stem cells. Nat Commun *9*, 1303. 10.1038/s41467-018-03760-5.
34. Wienert, B., Nguyen, D.N., Guenther, A., Feng, S.J., Locke, M.N., Wyman, S.K., Shin, J., Kazane, K.R., Gregory, G.L., Carter, M.A.M., et al. (2020). Timed inhibition of CDC7 increases CRISPR-Cas9 mediated templated repair. Nat Commun *11*, 2109. 10.1038/s41467-020-15845-1.
35. Reint, G., Li, Z., Labun, K., Keskitalo, S., Soppa, I., Mamia, K., Tolo, E., Szymanska, M., Meza-Zepeda, L.A., Lorenz, S., et al. (2021). Rapid genome editing by CRISPR-Cas9-POLD3 fusion. Elife *10*. 10.7554/eLife.75415.
36. Labun, K., Guo, X., Chavez, A., Church, G., Gagnon, J.A., and Valen, E. (2019). Accurate analysis of genuine CRISPR editing events with ampliCan. Genome Res *29*, 843-847. 10.1101/gr.244293.118.
37. Tsai, S.Q., Zheng, Z., Nguyen, N.T., Liebers, M., Topkar, V.V., Thapar, V., Wyvekens, N., Khayter, C., Iafrate, A.J., Le, L.P., et al. (2015). GUIDE-seq enables genome-wide profiling of off-target cleavage by CRISPR-Cas nucleases. Nat Biotechnol *33*, 187-197. 10.1038/nbt.3117.
38. Zhu, L.J., Lawrence, M., Gupta, A., Pagès, H., Kucukural, A., Garber, M., and Wolfe, S.A. (2017). GUIDEseq: a bioconductor package to analyze GUIDE-Seq datasets for CRISPR-Cas nucleases. BMC Genomics *18*, 379. 10.1186/s12864-017-3746-y.
39. Savić, N., Ringnalda, F.C., Berk, C., Bargsten, K., Hall, J., Jinek, M., and Schwank, G. (2019). In vitro Generation of CRISPR-Cas9 Complexes with Covalently Bound Repair Templates for Genome Editing in Mammalian Cells. Bio Protoc *9*. 10.21769/BioProtoc.3136.
40. Savic, N., Ringnalda, F.C., Lindsay, H., Berk, C., Bargsten, K., Li, Y., Neri, D., Robinson, M.D., Ciaudo, C., Hall, J., et al. (2018). Covalent linkage of the DNA repair template to the CRISPR-Cas9 nuclease enhances homology-directed repair. Elife *7*. 10.7554/eLife.33761.

41. Picelli, S., Faridani, O.R., Björklund, A.K., Winberg, G., Sagasser, S., and Sandberg, R. (2014). Full-length RNA-seq from single cells using Smart-seq2. Nat Protoc *9*, 171-181. 10.1038/nprot.2014.006.
42. Zachariadis, V., Cheng, H., Andrews, N., and Enge, M. (2020). A Highly Scalable Method for Joint Whole-Genome Sequencing and Gene-Expression Profiling of Single Cells. Mol Cell *80*, 541-553.e545. 10.1016/j.molcel.2020.09.025.
43. Martin, M. (2011). Cutadapt Removes Adapter Sequences From High-Throughput Sequencing Reads. EMBnet.journal. https://doi.org/10.14806/ej.17.1.200.
44. Dobin, A., Davis, C.A., Schlesinger, F., Drenkow, J., Zaleski, C., Jha, S., Batut, P., Chaisson, M., and Gingeras, T.R. (2013). STAR: ultrafast universal RNA-seq aligner. Bioinformatics *29*, 15-21. 10.1093/bioinformatics/bts635.
45. McKenna, A., Hanna, M., Banks, E., Sivachenko, A., Cibulskis, K., Kernytsky, A., Garimella, K., Altshuler, D., Gabriel, S., Daly, M., and DePristo, M.A. (2010). The Genome Analysis Toolkit: a MapReduce framework for analyzing next-generation DNA sequencing data. Genome Res *20*, 1297-1303. 10.1101/gr.107524.110.
46. Anders, S., Pyl, P.T., and Huber, W. (2015). HTSeq--a Python framework to work with high-throughput sequencing data. Bioinformatics *31*, 166-169. 10.1093/bioinformatics/btu638.
47. Satija, R., Farrell, J.A., Gennert, D., Schier, A.F., and Regev, A. (2015). Spatial reconstruction of single-cell gene expression data. Nat Biotechnol *33*, 495-502. 10.1038/nbt.3192.
48. Korotkevich, G., Sukhov, V., Budin, N., Shpak, B., Artyomov, M.N., and Sergushichev, A. (2021). Fast gene set enrichment analysis. bioRxiv, 060012. 10.1101/060012.
49. Haas, B.J., Dobin, A., Li, B., Stransky, N., Pochet, N., and Regev, A. (2019). Accuracy assessment of fusion transcript detection via read-mapping and de novo fusion transcript assembly-based methods. Genome Biol *20*, 213. 10.1186/s13059-019-1842-9.
50. Danecek, P., Bonfield, J.K., Liddle, J., Marshall, J., Ohan, V., Pollard, M.O., Whitwham, A., Keane, T., McCarthy, S.A., Davies, R.M., and Li, H. (2021). Twelve years of SAMtools and BCFtools. GigaScience *10*. 10.1093/gigascience/giab008.
51. Huang, X., and Huang, Y. (2021). Cellsnp-lite: an efficient tool for genotyping single cells. Bioinformatics *37*, 4569-4571. 10.1093/bioinformatics/btab358.
52. Li, H. (2011). A statistical framework for SNP calling, mutation discovery, association mapping and population genetical parameter estimation from sequencing data. Bioinformatics *27*, 2987-2993. 10.1093/bioinformatics/btr509.
53. Das, S., Forer, L., Schönherr, S., Sidore, C., Locke, A.E., Kwong, A., Vrieze, S.I., Chew, E.Y., Levy, S., McGue, M., et al. (2016). Next-generation genotype imputation service and methods. Nat Genet *48*, 1284-1287. 10.1038/ng.3656.
54. Stubbington, M.J.T., Lönnberg, T., Proserpio, V., Clare, S., Speak, A.O., Dougan, G., and Teichmann, S.A. (2016). T cell fate and clonality inference from single-cell transcriptomes. Nat Methods *13*, 329-332. 10.1038/nmeth.3800.
55. Meier, F., Brunner, A.D., Frank, M., Ha, A., Bludau, I., Voytik, E., Kaspar-Schoenefeld, S., Lubeck, M., Raether, O., Bache, N., et al. (2020). diaPASEF: parallel accumulation-serial fragmentation combined with data-independent acquisition. Nat Methods *17*, 1229-1236. 10.1038/s41592-020-00998-0.
56. Demichev, V., Messner, C.B., Vernardis, S.I., Lilley, K.S., and Ralser, M. (2020). DIA-NN: neural networks and interference correction enable deep proteome coverage in high throughput. Nat Methods *17*, 41-44. 10.1038/s41592-019-0638-x.
57. Demichev, V., Szyrwiel, L., Yu, F., Teo, G.C., Rosenberger, G., Niewienda, A., Ludwig, D., Decker, J., Kaspar-Schoenefeld, S., Lilley, K.S., et al. (2022). dia-PASEF data analysis using FragPipe and DIA-NN for deep proteomics of low sample amounts. Nat Commun *13*, 3944. 10.1038/s41467-022-31492-0.
58. Lazar, C. (2015). imputeLCMD: a collection of methods for left-censored missing data imputation. R package, version 2.1.
59. Virtanen, P., Gommers, R., Oliphant, T.E., Haberland, M., Reddy, T., Cournapeau, D., Burovski, E., Peterson, P., Weckesser, W., Bright, J., et al. (2020). SciPy 1.0: fundamental algorithms for scientific computing in Python. Nat Methods *17*, 261-272. 10.1038/s41592-019-0686-2.
60. Seabold, S., and Perktold, J. (2010). Statsmodels: Econometric and Statistical Modeling with Python. SciPy.
61. Renesh, B. (2020). Reneshbedre/Bioinfokit: Bioinformatics Data Analysis and Visualization Toolkit| Zenodo.
